# Access to Substituted Tricyclic Heteroarenes by an Oxidative Cyclization Reaction and Their Antifungal Performance

**DOI:** 10.3390/ph18020249

**Published:** 2025-02-12

**Authors:** Rehema Nakiwala, Noopur Dasgupta, Rebecca Wilson, Erika I. Lutter, Jeanne L. Bolliger

**Affiliations:** 1 Department of Chemistry, 107 Physical Sciences, Oklahoma State University, Stillwater, OK 74078, USA; 2 Department of Microbiology and Molecular Genetics, 307 Life Sciences East, Oklahoma State University, Stillwater, OK 74078, USA

**Keywords:** antifungal agents, organosulfur compounds, 1,2,4-triazoles, benzo[4,5]thiazolo[2,3-*c*][1,2,4]triazoles, oxidative cyclization, *Candida albicans*, *Trichosporon asahii*

## Abstract

**Background/Objectives**: Fungal pathogens are increasingly developing concerning resistance against the currently available antifungal drugs, which creates a constant demand for new antifungal agents. **Methods**: Here, we report the synthesis of C3,N4-substituted triazole derivatives containing a N4-(2-((4-methoxybenzyl)thio)phenyl) group. By selectively removing the 4-methoxybenzyl group, we were able to access the free thiol analogs which, under oxidative conditions, undergo a cyclization reaction yielding a C5-substituted benzo[4,5]thiazolo[2,3-*c*][1,2,4]triazole. We were able to show a broad functional group tolerance for the preparation of the triazole derivatives, as well as the tricyclic heteroarenes prepared thereof. Mechanistic investigations suggest that the oxidative cyclization reaction proceeds via an ionic pathway involving a disulfide intermediate. Isolation of the disulfide intermediate and resubjecting it to the reaction conditions shows that the presence of acid significantly increases its rate of conversion to the corresponding benzo[4,5]thiazolo[2,3-*c*][1,2,4]triazole. Antifungal testing of both the novel triazoles and the benzo[4,5]thiazolo[2,3-*c*][1,2,4]triazoles was carried out with *Candida albicans* (SC5314) and a clinical strain of *Trichosporon asahii* (OK01). **Results**: Most of the novel sulfur-containing triazoles and benzo[4,5]thiazolo[2,3-*c*][1,2,4]triazoles showed activity against *Candida albicans* (SC5314) and the emerging pathogen *Trichosporon asahii* (OK01). **Conclusions**: A series of new sulfur-containing triazoles and benzo[4,5]thiazolo[2,3-*c*][1,2,4]triazoles were synthesized. Antifungal testing revealed modest activity against *Candida albicans* (SC5314) and *Trichosporon asahii* (OK01).

## 1. Introduction

**Figure 1:**
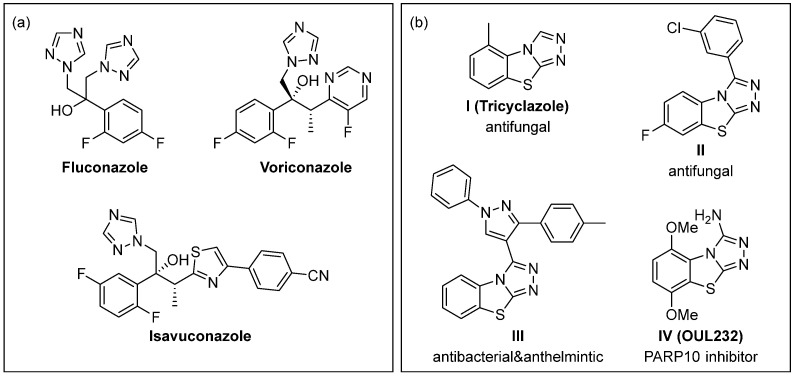
(**a**) FDA-approved antifungal drugs containing a triazole. (**b**) Selected examples of biologically active benzo[4,5]thiazolo[2,3-*c*][1,2,4]triazoles.

Fungal infections are increasingly a problem in people with a weakened immune system, such as patients undergoing chemotherapy, HIV-positive people, or patients with cystic fibrosis due to the emergence of resistant strains [1,2,3,4,5,6,7,8,9,10,11,12,13]. While *Candida albicans* and *Aspergillus fumigatus* are among the most frequently identified fungal pathogens in the lungs of individuals with cystic fibrosis, *Trichosporon* strains have more recently been identified as concerning novel respiratory pathogens in these patients [13,14,15]. The treatment of fungal infections currently relies on a limited number of antifungal medications belonging to just five classes of drugs, of which azoles (e.g., fluconazole and voriconazole, see Figure 1a) are most commonly prescribed to treat infections caused by different strains of *Candida* and *Trichosporon* [16,17]. To overcome solubility issues, recent developments have led to triazolium salts such as isavuconazonium sulfate being used as a prodrug which is converted by plasma esterase to the active antifungal agent isavuconazole (Figure 1a, right) [18].

In the past two decades, several groups have reported 1,2,4-triazole-containing antifungal candidates including 5-oxo-[1,2,4]triazole derivatives [19,20], 3,5-substituted 1,2,4-triazoles [21,22,23], and multicyclic triazole-containing compounds [24], of which many have demonstrated activity against fluconazole-resistant strains of *Candida albicans*. Meanwhile, compounds containing the tricyclic benzo[4,5]thiazolo[2,3-*c*][1,2,4]triazole scaffold have shown promising biological activity (Figure 1b) [25,26,27,28,29,30,31,32]. A member of that family, tricyclazole (Figure 1b, **I**) has been in use as a fungicide against rice blast for the past five decades [25]. Differently substituted benzo[4,5]thiazolo[2,3-*c*][1,2,4]triazoles, including **II** and **III**, have also shown activity against phytopathogenic fungi [26,27] and against bacteria and other microbes [28,29,30,31]. Just last year, OUL232 (Figure 1b, **IV**) has been reported to act as a nicotinamide mimic capable of selectively inhibiting the PARP10 enzyme over other PARP enzymes, even at low nanomolar concentrations [32]. Interestingly, the authors report that the benzo[4,5]thiazolo[2,3-*c*][1,2,4]triazole scaffold of OUL232 and related compounds proved to be not cytotoxic in cell viability assays, thus making this a promising scaffold for future drug development.

**Scheme 1:**
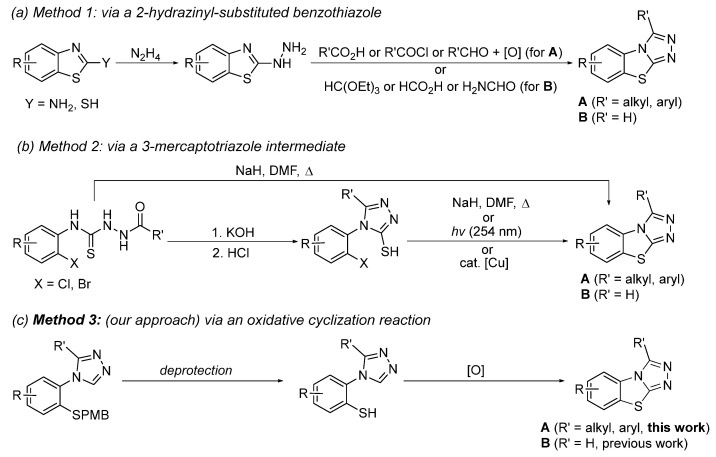
Synthetic pathways yielding the benzo[4,5]thiazolo[2,3-*c*][1,2,4]triazole scaffold.

The preparation of compounds containing the benzo[4,5]thiazolo[2,3-*c*][1,2,4]triazole scaffold is generally carried out by one of the three pathways shown in Scheme 1. The first method starts with either a 2-amino- or a 2-mercaptobenzothiazole (Scheme 1a). Treatment with hydrazine leads to the 2-hydrazinyl derivative which is then reacted with an electrophilic reagent such as formic acid or an acid chloride to form the triazole ring in the last step [26,33,34,35]. The second approach proceeds via a 3-mercaptotriazole intermediate (Scheme 1b). Upon treatment with a strong base, the central thiazole ring is formed at elevated temperatures [36]. Alternatively, UV irradiation or the presence of a catalytic amount of copper(II) has been observed to allow this ring closure [37,38]. More recently, we have reported a third pathway beginning with a protected 2-mercaptophenyl substituted triazole which, upon deprotection of the thiol, undergoes cyclization under oxidative conditions to yield the desired benzo[4,5]thiazolo[2,3-*c*][1,2,4]triazole **B** (R = H) [39,40]. Our past research has demonstrated a high functional group tolerance for substituents on the phenyl ring. In our current work, we expand the substrate scope to benzo[4,5]thiazolol[2,3-*c*][1,2,4]triazoles containing substituents on the triazole ring (**A**, R′ ≠ H), as well as benzo[4,5]thiazolol[2,3-*c*][1,2,4]triazoles containing both substituents on the phenyl ring and on the triazole ring.

Here, we report the synthesis of a new series of C3-substituted triazoles containing a N4-(2-((4-methoxybenzyl)thio)phenyl) substituent. Upon removal of the *p*-methoxybenzyl-protecting group from the sulfur, these substituted triazoles undergo an oxidative cyclization reaction in the presence of a mild oxidizing agent, thereby yielding the corresponding tricyclic benzo[4,5]thiazolo[2,3-*c*][1,2,4]triazoles. Both the novel triazoles and the tricyclic heteroarenes were evaluated for antifungal activity against the human pathogens *Candida albicans* and a clinical strain of *Trichosporon asahii*, and their activity was compared to the activity of fluconazole.

## 2. Results

### 2.1. Synthesis of 2-((4-Methoxybenzyl)thio)aniline Derivatives

**Scheme 2:**
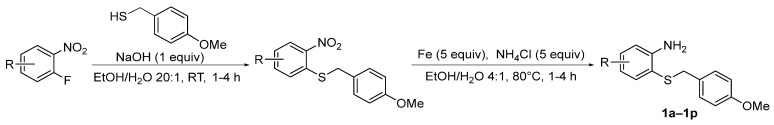
Preparation of substituted 2-((4-methoxybenzyl)thio)anilines.

The 2-((4-methoxybenzyl)thio)aniline derivatives **1a**–**1p** required for the 1,2,4-triazole were synthesized in two steps from the appropriate 2-fluoronitrobenzene (Scheme 2). In the first step, a nucleophilic aromatic substitution, the sulfur was introduced together with its protecting group, thus yielding the corresponding (4-methoxybenzyl)(2-nitrophenyl)sulfanes in excellent yields. The subsequent reduction in the nitrobenzene to the aniline afforded the desired substituted 2-((4-methoxybenzyl)thio)anilines in generally excellent yields (see Supporting Information).

### 2.2. Synthesis of 4-Aryl-3-R-1,2,4-triazoles

**Scheme 3:**
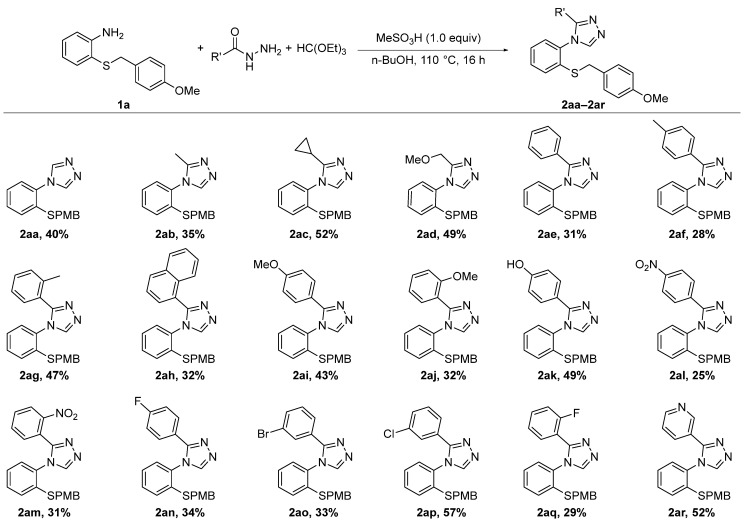
Preparation of substituted 2-((4-methoxybenzyl)thio)anilines.

4-Aryl-3-*R*-1,2,4-triazoles can be prepared from a hydrazone, an aniline and DMFDMA in the presence of an acid [41]. When we attempted this method, no product was observed in the case of **1a** as aniline and 3-chlorobenzohydrazide as hydrazone under a variety of reaction conditions. An alternative route is to first prepare an oxadiazole from a hydrazone and triethylorthoformate under acid catalysis, and then react this oxadiazole with the aniline to the corresponding 4-aryl-3-*R*-1,2,4-triazole [42,43,44,45]. Using this approach, we were able to carry out the triazole formation with **1a** as aniline and a range of hydrazides in 1-butanol in the presence of stoichiometric methanesulfonic acid (Scheme 3). Partial conversion was observed relative to the aniline **1a** within 16 h, after which no further increase in yield was seen by LCMS. Isolated yields were in the range of 25–57%, and in all cases, 90–95% of the unreacted aniline **1a** could be recovered during the purification step. Among the hydrazides used in the synthesis were molecules containing aliphatic substituents, aromatic substituents and even heteroaromatic substituents. With aliphatic (**2ab**–**2ad**) or no substitution (**2aa**) on C3, the triazoles were isolated in comparably good yields and the aniline **1a** could be recovered for reuse. However, it is worth mentioning that the triazole **2aa** which does not contain any carbon substituents can be obtained in significantly higher yields by reacting the aniline **1a** with *N,N*-dimethylformamide azine dihydrochloride, as we and others have previously reported [46,47]. Among the triazoles containing an aryl substituent on C3 (**2ae**–**2aq**), the highest yields were observed for compounds containing an electron-donating *para*-substituent (**2ai**, **2ak**), the *ortho*-tolyl derivative **2ag** and not surprisingly the *meta*-chlorobenzene compound **2ap**, since it was used in the reaction optimization. Additionally, triazole **2ar** with a 3-pyridyl substituent on C3 was obtained in good yields.

**Scheme 4:**
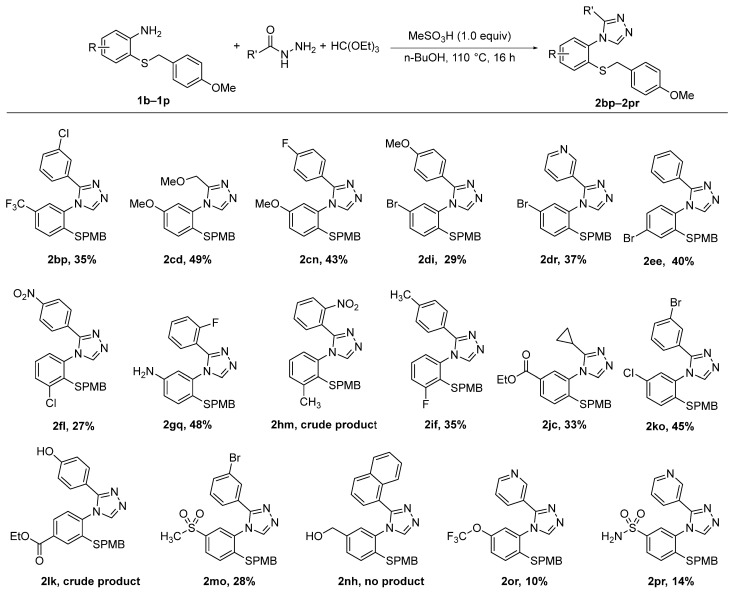
Triazole synthesis from substituted 2-((4-methoxybenzyl)thio)anilines.

Next, we prepared triazoles **2bp**–**2pr** containing a wide range of biologically relevant functional groups on the aromatic ring (Scheme 4) by replacing aniline **1a** with the substituted anilines **1b**–**1r**. Except for the benzyl alcohol **2nh**, all triazoles were formed under our reaction conditions. Purification by column chromatography generally afforded both the pure triazole product, as well as the unreacted aniline starting material. **2hm** and **2lk** were obtained in crude form. **2hm** was difficult to purify by column chromatography due to other compounds eluting simultaneously with the product. Meanwhile, the phenol containing compound **2lk** was strongly retained on silica and did not elute even with pure methanol, thus making purification by column chromatography impossible.

### 2.3. Synthesis of Benzo[4,5]thiazolo[2,3-c][1,2,4]triazoles

**Scheme 5:**
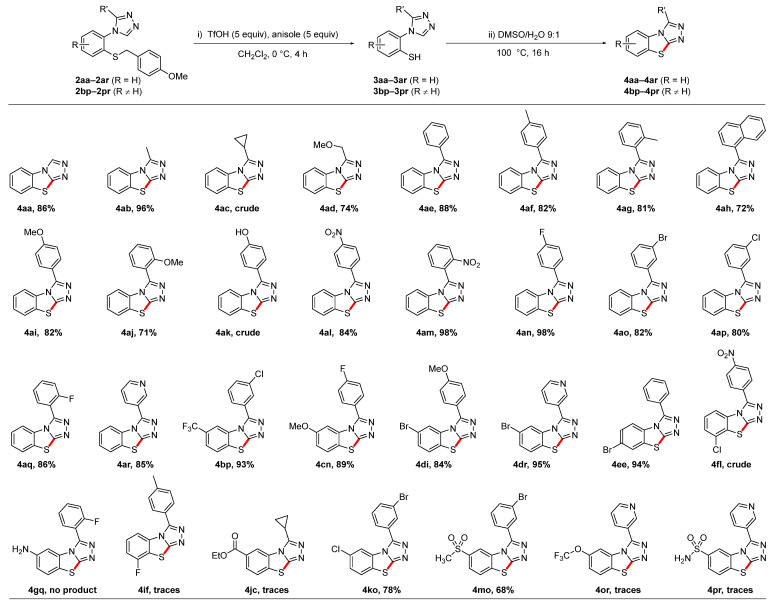
Deprotection and oxidative cyclization yielding substituted benzo[4,5]thiazolo[2,3-*c*][1,2,4]triazoles.

With the C3-substituted triazoles **2aa**–**2ar** and **2bp**–**2pr** in hand, we explored two-step synthesis of the tricyclic benzo[4,5]thiazolo[2,3-*c*][1,2,4]triazoles **4aa**–**4ar** and **4bp**–**4pr** by an oxidative cyclization reaction, as described in Scheme 1c. Compared to our previously reported procedure utilizing triazoles without C3 substituents [39,40], we found that both the deprotection step, as well as the subsequent cyclization, required prolonged reaction times (Scheme 5). The protected thiol of triazoles **2aa**–**2ar** and **2bp**–**2pr** was selectively deprotected with trifluoromethanesulfonic acid and anisole in dichloromethane under an argon atmosphere within 4 h yielding the free thiols **3aa**–**3ar** and **3bp**–**3pr**, as confirmed by LCMS. Upon completion of the deprotection, the reaction solvent was removed on a rotary evaporator, which yielded a red residue. It is worth noting that, contrary to the method reported previously by our group, we decided to employ dichloromethane as the deprotection solvent as it was easier to remove and did not corrode our pumps as the initial solvent (trifluoroacetic acid) did. By simply redissolving the red residue in a 9:1 DMSO/water mixture and heating in an oil bath at 100 °C overnight, a clean conversion to the corresponding C5-substituted benzo[4,5]thiazolo[2,3-*c*][1,2,4]triazoles **4aa**–**4ar** was observed in all cases. Isolated yields for these tricyclic heteroarenes containing a wide range of aliphatic substituents, electron-donating and electron-withdrawing aryl groups or a pyridine functionality ranged from 71 to 98%. However, both the cyclopropyl substituted benzo[4,5]thiazolo[2,3-*c*][1,2,4]triazole **4ac** and the phenol-substituted benzo[4,5]thiazolo[2,3-*c*][1,2,4]triazole **4ak** could only be isolated in crude form. The synthesis of the C5-substituted benzo[4,5]thiazolo[2,3-*c*][1,2,4]triazoles **4bp**–**4pr**, which also contained a range of substituents on the benzene ring of the tricyclic scaffold, was much less predictable. While in many cases, complete conversion was observed by LCMS and the tricyclic compound was isolated in excellent yields (examples: **4bp**, **4cn**, or **4dr**), the amino-substituted benzo[4,5]thiazolo[2,3-*c*][1,2,4]triazole **4gq** was not even detected by LCMS, and only traces were observed with the sulfonamide **4pr**. Additionally, the ester **4jc** was only detected in trace amounts, which was most likely due to the hydrolysis of the ester in the presence of acid and water. In the absence of water, excellent yields have been obtained in our group with related compounds [39].

**Scheme 6:**
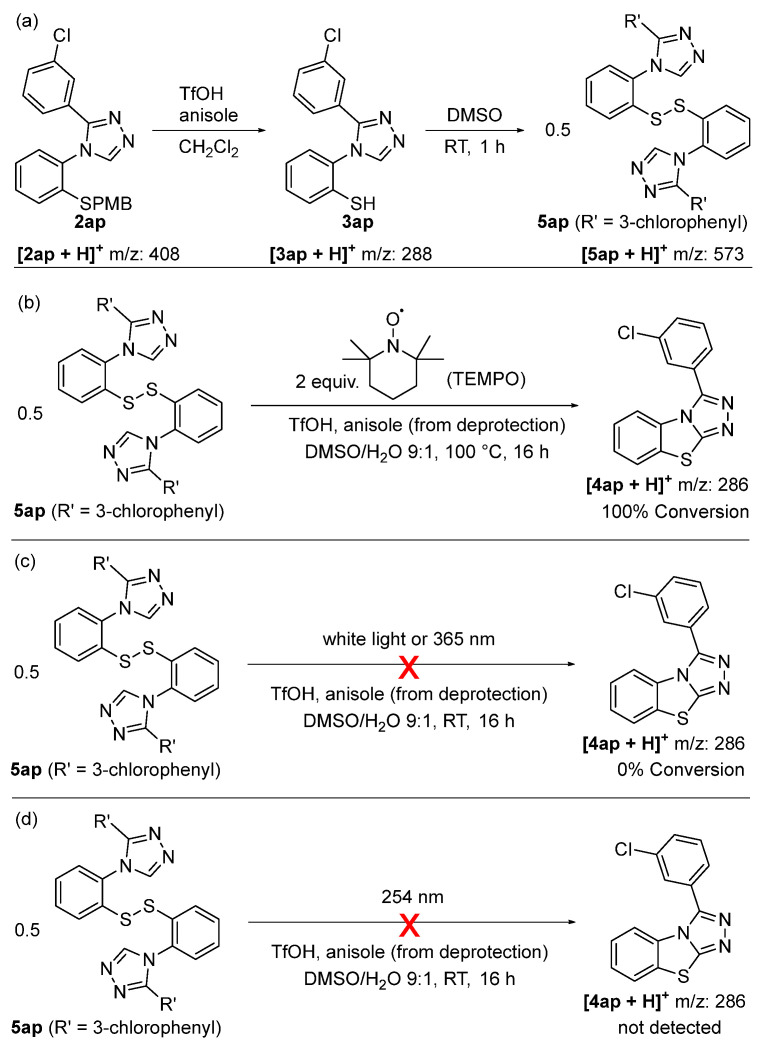
Mechanistic investigations. (**a**) Preparation of disulfide **5ap**. (**b**) Cyclization in the presence of TEMPO. (**c**) No cyclization under irradiation of white light or long-range UV light (365 nm). (**d**) No product formation by irradiation with short-wave UV light (254 nm).

Mechanistic investigations and close monitoring by LCMS indicate that the oxidative cyclization step proceeds via a disulfide intermediate which requires thermal energy (i.e., heat) to convert to the benzo[4,5]thiazolo[2,3-*c*][1,2,4]triazole (Scheme 6). Indeed, subjecting triazole **2ap** to our standard deprotection conditions yields the free thiol **3ap**, which upon addition of DMSO and stirring at room temperature (instead of 100 °C), forms the disulfide **5ap** as a sole product (Scheme 6a). The addition of two equivalents of TEMPO to the reaction mixture containing the disulfide **5ap**, followed by heating to 100 °C overnight, led to clean conversion to the benzo[4,5]thiazolo[2,3-*c*][1,2,4]triazole **4ap** without any TEMPO adducts being observed by LCMS, thus indicating that the cyclization step is unlikely to proceed via a radical mechanism (Scheme 6b). This is further supported by the fact that neither irradiation of the reaction mixture containing the disulfide **5ap** with white light nor with UV light at 365 nm led to the formation of the benzo[4,5]thiazolo[2,3-*c*][1,2,4]triazole **4ap** at room temperature (Scheme 6c). Furthermore, irradiation with UV light with a wavelength of 254 nm also failed to form the benzo[4,5]thiazolo[2,3-*c*][1,2,4]triazole **4ap**, but instead led to a messy mixture of other compounds (Scheme 6d). Because the disulfide bond is expected to absorb UV light at 254 nm, thiyl radicals are likely to be present under these reaction conditions [48]. The fact that conditions that favor radical formation are not suitable for our heterocycle formation and fail to give any product, while resulting in a multitude of other compounds, strongly suggests that the cyclization step is thermally activated.

**Scheme 7:**
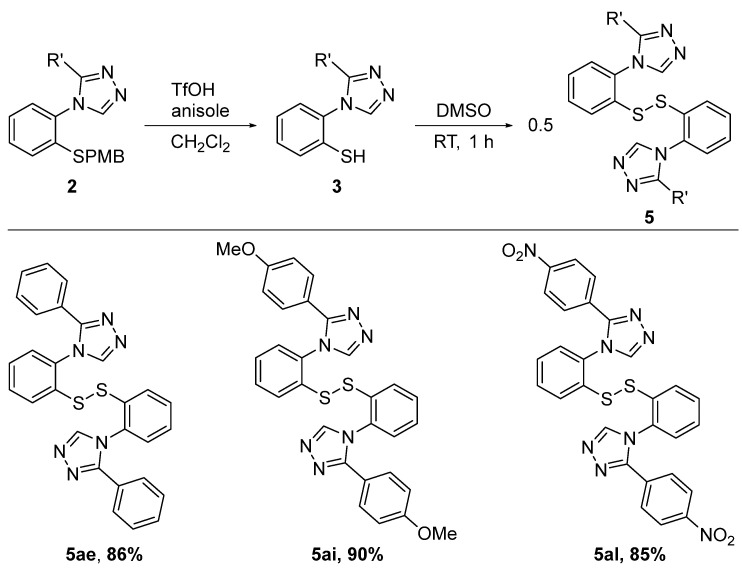
Isolation of disulfide intermediates.

We have previously investigated the electronic influence of substituents para to the disulfide group on the N-aryl ring on the relative rate of conversion of the disulfide to the tricyclic scaffold [40]. We have observed that electron-withdrawing groups (such as CF_3_) significantly increased the rate of this transformation, whereas electron-donating groups (such as OMe) decreased the rate compared to the unsubstituted analog which we assigned to the increase or decrease in the electrophilicity of the sulfur atoms in the disulfide bond. In contrast, the compounds in our current work offer the possibility to modulate and examine the effect of the electron density of the triazole ring in the cyclization step by substituting C3 with either electron-rich or electron-poor aryl groups. For this study, we selected three triazoles, namely **2ae** with a phenyl substituent on C3, **2ai** with the electron-donating 4-methoxyphenyl group on C3, and **2al** with the electron-withdrawing 4-nitrophenyl unit on C3. After the removal of the *para*-methoxybenzyl protecting group, the resulting thiols **3ae**, **3ai** and **3al** were subsequently oxidized at room temperature to the corresponding disulfides **5ae**, **5ai** and **5al**, which were isolated at over 85% yield (Scheme 7).

**Figure 2:**
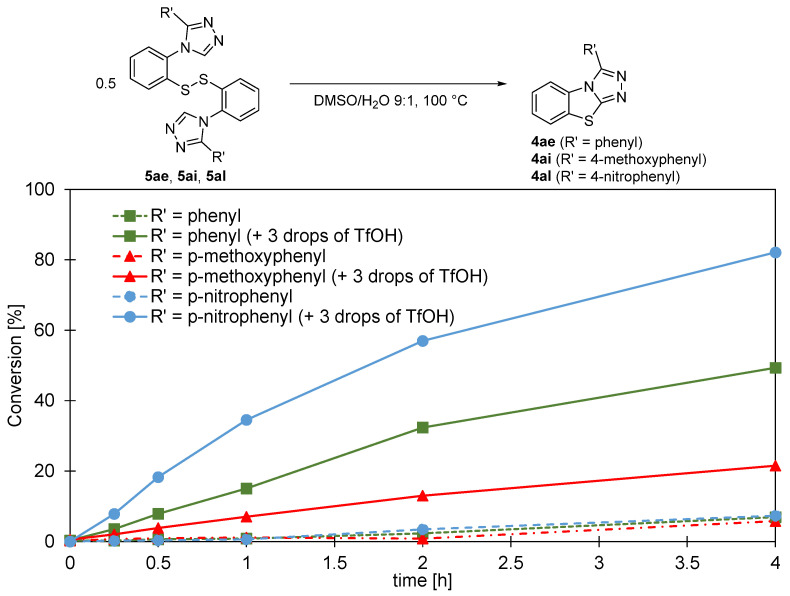
Substituent effects and the role of acid on the cyclization step.

Interestingly, when the isolated disulfides were dissolved in a 9:1 DMSO/water mixture and heated in an oil bath at 100 °C, the formation of the corresponding benzo[4,5]thiazolo[2,3-*c*][1,2,4]triazole was observed to occur at a drastically lower rate, as seen in Figure 2 (dashed curves), compared to the one-pot reactions performed in two steps from the protected thiols, which were typically complete within 6 to 16 h. Since the main difference between the two sets of conditions was the presence of triflic acid from the thiol deprotection, we added three drops of triflic acid to the disulfides prior to heating and, indeed, the conversions observed by LCMS for the transformation of the disulfides to the benzo[4,5]thiazolo[2,3-*c*][1,2,4]triazole (Figure 2, solid lines) then matched the ones for the one-pot procedure. As can be seen in Figure 2, the rates of formation of the benzo[4,5]thiazolo[2,3-*c*][1,2,4]triazole from the disulfides showed a strong correlation with the π donor/π acceptor properties of the aryl substituent R′ on the triazole C3 of the disulfide: **5al** containing the electron-withdrawing 4-nitrophenyl substituent shows over 80% conversion after 4 h which is a significantly higher rate than observed for **5ai** containing the electron-donating 4-methoxypenyl group, for which only approximately 20% conversion was found after 4 h. These observations, together with the lability of the triazole C5-H (proven by the rapid H-D exchange when dissolved in deuterated methanol), suggest that the deprotonation of C5-H of the disulfide is likely to play a key role in the formation of the benzo[4,5]thiazolo[2,3-*c*][1,2,4]triazole scaffold.

**Figure 3:**
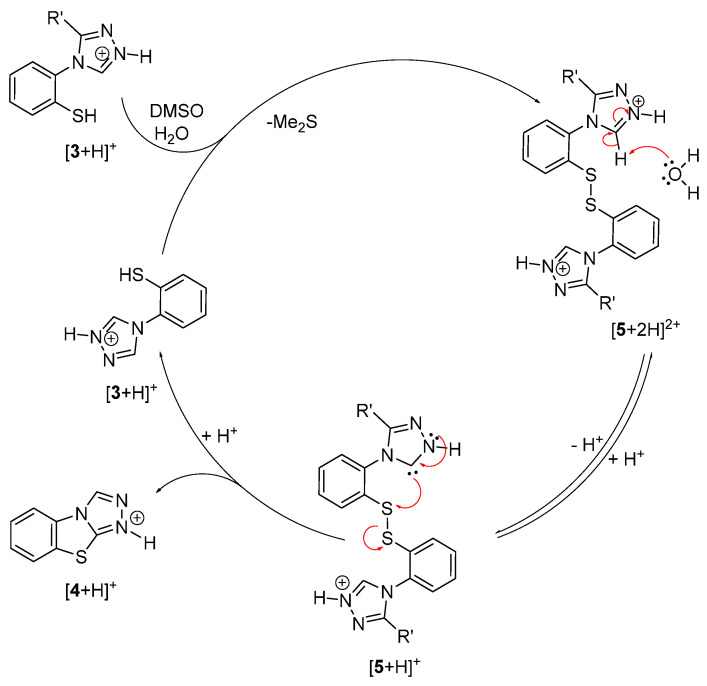
Proposed mechanistic cycle under acidic conditions.

The reaction mechanism displayed in Figure 3 is thought to be operative for oxidative cyclization under acidic conditions. Two deprotected thiol molecules (**3**) undergo rapid oxidation to the disulfide **5** (which can be isolated if the oxidation is carried out at room temperature, see above, Scheme 7). At elevated temperatures, this disulfide is seen to gradually convert to the benzo[4,5]thiazolo[2,3-*c*][1,2,4]triazole **4**. Since an excess of the acid from the deprotection step is present, the triazole will predominantly exist as a triazolium species which could undergo deprotonation of the C5-H, thus forming a highly nucleophilic carbene. As the equilibrium position between the triazolium and the carbene species will depend in this case on the π donor/π acceptor properties of the C3 substituent, electron-withdrawing groups (e.g., R′ = 4-nitrophenyl) are expected to increase and electron-donating groups (e.g., R′ = 4-methoxyphenyl) are expected to decrease the relative concentration of the carbene species. This carbene is thought to intramolecularly attack one of the sulfur atoms in the disulfide bond, thus forming the new C-S in the cyclization product **4**. The substituent effects observed in Figure 2 are in agreement with this proposed mechanism, and appear to indicate that the rate-determining step is the formation of the new C-S bond, which is dependent on the equilibrium position between the triazolium and the carbene species.

### 2.4. Antifungal Activity

**Figure 4:**
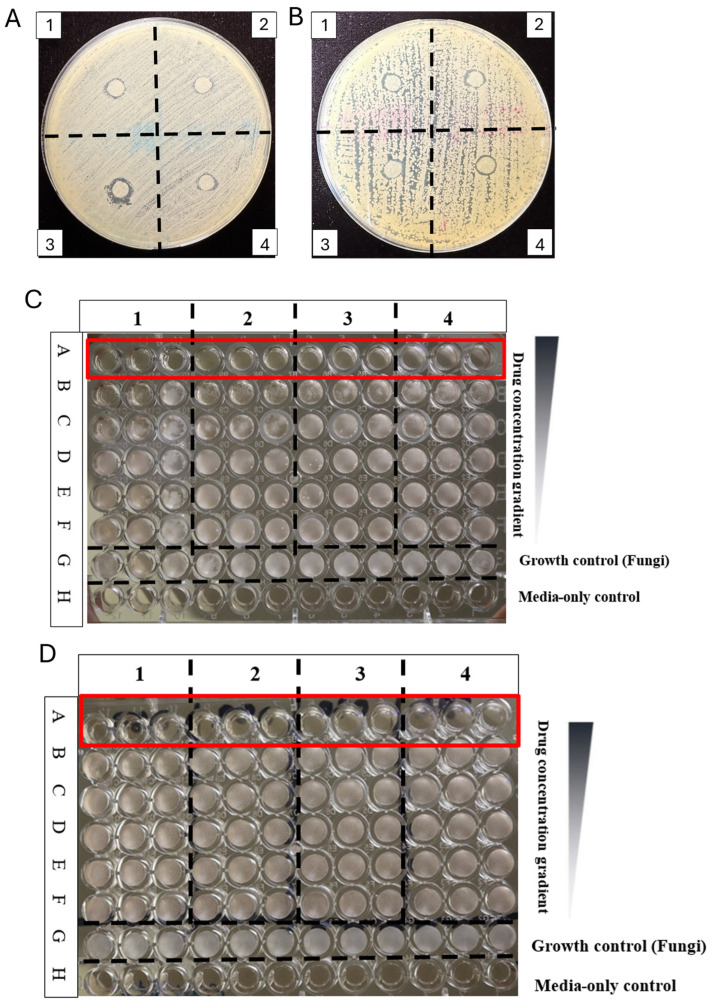
Disk diffusion assay and MICs of active compounds. Shown are **2if** (1), **2ar** (2), **4ah** (3) and **2af** (4), tested using a disk diffusion assay against *C. albicans* (**A**) and *T. asah* (**B**). MIC broth dilution against *C. albicans* (**C**) and *T. asah* (**D**) were tested with compounds **4ao** (1) **4an** (2) **4aq** (3) **2an** (4). Two-fold dilutions were made ranging from 256 µg/mL row A to 8 µg/mL row F; row G and H are fungal strains grown in media alone and media only control, respectively. All the compounds were tested in triplicates. Row A (in red) shows the inhibition of the fungi at the concentration of 256 µg/mL.

The novel triazoles **2ab**–**2ar** (Scheme 3) and **2bp**–**2pr** (Scheme 4), as well as the benzo[4,5]thiazolo[2,3-*c*][1,2,4]triazoles **4ab**–**4mo** (Scheme 5), were evaluated for their activity against *Candida albicans* and *Trichosporon asahii*, and compared to the activity of fluconazole, miconazole, voriconazole, amphotericin B, and caspofungin acetate. As seen in Table 1, all antifungal candidates were first tested using a disk diffusion plate inhibition assay to determine if there was inhibition. The minimum inhibitory concentration (MIC) in a broth dilution assay was then used to determine activity in liquid. DMSO was used as a vehicle control in both the disk diffusion assay (10 μL) and MIC (up to a solvent ratio of DMSO/broth = 1:3), with no effect on *C. albicans* and *T. asahii* growth. All antifungals that showed plate inhibition also resulted in inhibition in broth dilution assays (Table 1). Notably, *T. asahii* was susceptible to more antifungal candidates than *C. albicans*. Compounds **2if**, **2ar**, **4ah**, and **2af** are shown in Figure 4, as tested in the disk diffusion assay and the broth dilution MIC assay.

## 3. Discussion

We have prepared C3,N4-substituted triazole derivatives **2aa**–**2ar** (Scheme 3) and **2bp**–**2pr** (Scheme 4) containing a N4-(2-((4-methoxybenzyl)thio)phenyl) group. Selective removal of the 4-methoxybenzyl substituent from the sulfur atom followed by oxidation with DMSO at 100 °C yields the C5-substituted tricyclic benzo[4,5]thiazolo[2,3-*c*][1,2,4]triazoles **4bp**–**4pr** (Scheme 5). Detailed mechanistic investigations suggest that this oxidative cyclization reaction proceeds via an ionic pathway involving a disulfide intermediate which in the presence of a strong acid cyclizes to the corresponding tricyclic heteroarene (Scheme 6 and Scheme 7, Figure 2 and Figure 3).

Antifungal testing of both the novel sulfur-containing triazoles and the benzo[4,5]thiazolo[2,3-*c*][1,2,4]triazoles revealed that many of these compounds were moderately active against *C. albicans* and almost all molecules showed activity against the emerging fungal pathogen *T. asahii*. The structurally related antifungal drugs fluconazole, miconazole and voriconazole are known to bind to lanosterol 14α-demethylase (CYP51); however, it is unknown if our triazole-containing compounds have the same mechanism or not. [49] Interestingly, only some of the triazoles **2ab**–**2ar** and **2bp**–**2pr** produced zones inhibiting the fungal growth of *C. albicans*; however, apart from the pyridyl-substituted triazole **2ar**, all these compounds showed moderate activity against *T. asahii.* Meanwhile, all the C5-aryl substituted benzo[4,5]thiazolo[2,3-*c*][1,2,4]triazoles **4ae**–**4mo** exhibited moderate antifungal activity against *T. asahii* and were in most cases also active against *C. albicans* whereas the alkyl derivatives **4ab** and **4ad** were inactive against both fungal strains. In comparison to azole antifungal agents such as fluconazole, miconazole, voriconazole and other classes of antifungal agents, such as amphotericin B (a polyene antifungal medication) and caspofungin acetate (an echinocandin antifungal drug), our molecules showed a much lower activity against our test organisms.

Future work will involve cytotoxicity studies with HeLa and hepatic cells. We will also explore additional antifungal candidates.

## 4. Materials and Methods

### 4.1. General Information

Most reagents and solvents were purchased from Thermo Fisher Scientific (Waltham, MA, USA), Oakwood Chemical (Estill, SC, USA), TCI Chemicals (Montgomeryville, PA, USA) and VWR (Radnor, PA, USA), and used as supplied unless otherwise noted. Thermo Scientific™ Silica gel (Waltham, MA, USA; for column chromatography, 0.035–0.070 mm, 60 Å) was used for chromatographic separations. DMSO-*d*_6_ was dried over molecular sieves. For MIC assays, RPMI 1640 supplemented with 0.165 M morpholinepropanesulfonic acid (MOPS), pH 6.9–7 (filter-sterilized using a 0.22 µm filter) was used. Fungal strains were stored at −80 °C in 15% glycerol stocks and were plated on yeast extract peptone–dextrose (YPD) (BD Difco; Franklin Lakes, NJ, USA) agar plates.

### 4.2. Analyses

Melting points were obtained using an MEL-TEMP apparatus (Mel-Temp, Cambridge, MA, USA) and are uncorrected. ^1^H NMR, ^13^C{^1^H} NMR spectra and ^19^F{^1^H} NMR spectra were all recorded on a 400 MHz Bruker Avance III spectrometer with a 5 mm liquid-state smart probe. Chemical shifts (δ_H_, δ_C_) are expressed in parts per million (ppm) and reported relative to the resonance of the residual protons of the DMSO-*d*_6_ (δ_H_ = 2.50 ppm) or CDCl_3_ (δ_H_ = 7.26 ppm), or in ^13^C{^1^H} NMR spectra relative to the resonance of the deuterated solvent DMSO-*d*_6_ (δ_C_ = 39.52 ppm) or CDCl_3_ (δ_C_ = 77.16 ppm). Chemical shifts in ^19^F{^1^H} NMR spectra are reported relative to the internal standard fluorobenzene (δ_F_ = −113.15). Coupling constants (*J*) are given in Hz. All measurements were carried out at 298 K. Abbreviations used in the description of NMR data are as follows: s, singlet; d, duplet; t, triplet; q, quartet; sept, septet; m, multiplet. High resolution mass spectrometry (HRMS) data were obtained on an LTQ Orbitrap XL in FT Orbitrap Mode at a resolution of 100,000.

### 4.3. General Synthetic Procedures and Characterization of Compounds

#### 4.3.1. General Procedure 1 for the Preparation of 1,2,4-Triazoles

The procedure described below is for a 10 mmol scale reaction. The size of the round-bottomed flask and the solvent amounts were scaled accordingly for smaller scale reactions. A 25 mL round-bottomed flask was loaded with hydrazide (1.1 equiv., 11.0 mmol) aniline (1 equiv., 10.0 mmol), 1-butanol (10.0 mL), triethyl orthoformate (1.1 equiv., 11.0 mmol) and methanesulfonic acid (10 mmol, 1.0 equiv.). After sealing the flask with a septum, the reaction mixture was stirred at 110 °C for 16 h. The reaction mixture was cooled to room temperature and the solvent removed on a rotary evaporator. After adding 1 M NaOH, the aqueous phase was extracted three times with dichloromethane. The combined organic fractions were dried over MgSO_4_, filtered and concentrated on the rotary evaporator. The crude product was purified by column chromatography, as described below. The majority of the unreacted aniline was recovered during column chromatography from the initial fractions collected with dichloromethane as the eluent.

**4-(2-((4-Methoxybenzyl)thio)phenyl)-4*H*-1,2,4-triazole (2aa).** The title compound was prepared according to general procedure 1 on a 2.453 g (7.30 mmol) scale. Purification by column chromatography (silica gel, 1. dichloromethane, 2. dichloromethane/acetone 4:1, R_f_ = 0.20) gave the product as an off-white powder in 40% (0.72 g, 2.42 mmol) yield. The characterization data are in agreement with its characterization data reported in the literature [46,47,50].

**4-(2-((4-Methoxybenzyl)thio)phenyl)-3-methyl-4*H*-1,2,4-triazole (2ab).** The title compound was prepared according to general procedure 1 on a 2.453 g (10.00 mmol) scale. Purification by column chromatography (silica gel, 1. dichloromethane, 2. dichloromethane/acetone 4:1, R_f_ = 0.08) gave the product as a light-yellow powder in 35% (0.853 g, 2.74 mmol) yield; m.p. 149–150 °C. ^1^H NMR (400 MHz, DMSO-*d*_6_, 298 K): δ = 8.44 (s, 1H), 7.66 (dd, *J =* 8.1, 1.3 Hz, 1H), 7.54 (td, *J =* 7.5, 1.8 Hz, 1H), 7.44–7.32 (m, 2H), 7.21 (d, *J =* 8.7 Hz, 2H), 6.84 (d, *J =* 8.7 Hz, 2H), 4.17 (s, 2H), 3.71 (s, 3H), 2.05 (s, 3H); ^13^C{^1^H} NMR (100 MHz, DMSO-*d*_6_, 298 K): δ = 158.5, 154.0, 150.2, 149.6, 144.0, 135.3, 132.0, 130.4, 130.0, 128.8, 128.2, 128.1, 126.6, 113.9, 55.1, 35.3, 9.7. HRMS (ESI) *m*/*z* calculated for [M + H]^+^ = [C_17_H_18_N_3_OS]^+^ 312.1165; observed, 312.1167.

**3-Cyclopropyl-4-(2-((4-methoxybenzyl)thio)phenyl)-4*H*-1,2,4-triazole (2ac).** The title compound was prepared according to general procedure 1 on a 0.490 g (2.00 mmol) scale. Purification by column chromatography (silica gel, 1. dichloromethane, 2. dichloromethane/acetone 1:1, Rf = 0.18) gave the product as an off-white powder in 52% (0.349 g, 1.03 mmol) yield; m.p. 135–136 °C. ^1^H NMR (400 MHz, DMSO-*d*_6_, 298 K): δ = 8.38 (s, 1H), 7.67 (dd, *J =* 8.0, 1.4 Hz, 1H), 7.54 (td, *J =* 7.6, 1.7 Hz, 1H), 7.44 (dd, *J =* 7.8, 1.6 Hz, 1H), 7.38 (td, *J =* 7.5, 1.3 Hz, 1H), 7.22 (s, 2H), 6.84 (d, *J =* 8.8 Hz, 2H), 4.17 (s, 2H), 3.70 (s, 3H), 1.36 (ddd, *J =* 13.4, 8.3, 5.0 Hz, 1H), 0.88 (s, 2H), 0.81 (dd, *J =* 8.4, 2.4 Hz, 2H); ^13^C{^1^H} NMR (100 MHz, DMSO-*d*_6_, 298 K): δ = 158.5, 155.0, 143.8, 143.4, 14.6, 133.6, 132.8, 132.0, 130.4, 130.0, 128.9, 128.5, 128.1, 127.3, 127.1, 126.6, 113.9, 55.1, 36.3, 35.5, 7.3, 4.9. HRMS (ESI) *m*/*z* calculated for [M + H]^+^ = [C_19_H_20_N_3_OS]^+^ 338.1322; observed, 338.1325.

**4-(2-((4-Methoxybenzyl)thio)phenyl)-3-(methoxymethyl)-4*H*-1,2,4-triazole (2ad).** The title compound was prepared according to general procedure 1 on a 1.227 g (5.00 mmol) scale. Purification by column chromatography (silica gel, 1. dichloromethane, 2. dichloromethane/acetone 1:1, R_f_ = 0.19) gave the product as an off-white powder in 49% (0.839 g, 2.46 mmol) yield; m.p. 62–63 °C. ^1^H NMR (400 MHz, DMSO-*d*_6_, 298 K): δ = 8.55 (s, 1H), 7.66 (d, *J =* 7.9 Hz, 1H), 7.58–7.49 (m, 1H), 7.45–7.33 (m, 2H), 7.18 (d, *J =* 8.7 Hz, 2H), 6.84 (d, *J =* 8.7 Hz, 2H), 4.24 (s, 2H), 4.15 (s, 2H), 3.71 (s, 3H), 3.06 (s, 3H); ^13^C{^1^H} NMR (100 MHz, DMSO-*d*_6_, 298 K): δ = 158.5, 150.3, 145.2, 143.4, 134.9, 132.1, 130.4, 130.0, 129.4, 128.3, 128.2, 126.7, 62.70, 6.56, 55.1, 35.8. HRMS (ESI) *m*/*z* calculated for [M + H]^+^ = [C_18_H_20_N_3_O_2_S]^+^ 342.1271; observed, 342.1269.

**4-(2-((4-Methoxybenzyl)thio)phenyl)-3-phenyl-4*H*-1,2,4-triazole (2ae).** The title compound was prepared according to general procedure 1 on a 2.453 g (10.00 mmol) scale. Purification by column chromatography (silica gel, 1. dichloromethane, 2. dichloromethane/acetone 4:1, R_f_ = 0.11) gave the product as a colorless powder in 31% (0.953 g, 2.55 mmol) yield; m.p. 144–145 °C. ^1^H NMR (400 MHz, DMSO-*d*_6_, 298 K): δ = 8.63 (s, 1H), 7.61 (dd, *J =* 8.0, 1.3 Hz, 1H), 7.51 (td, *J =* 7.8, 1.5 Hz, 1H), 7.45 (dd, *J =* 7.8, 1.5 Hz, 1H), 7.42–7.36 (m, 1H), 7.35–7.29 (m, 5H), 7.11 (d, *J =* 8.7 Hz, 2H), 6.79 (d, *J =* 8.7 Hz, 2H), 4.10 (s, 2H), 3.72 (s, 3H); ^13^C{^1^H} NMR (100 MHz, DMSO-*d*_6_, 298 K): δ = 158.4, 152.3, 145.6, 135.3, 133.4, 132.6, 130.5, 130.0, 129.7, 128.9, 128.6, 128.0, 127.6, 126.6, 113.8, 55.0, 35.2. HRMS (ESI) *m*/*z* calculated for [M + H]^+^ = [C_22_H_20_N_3_OS]^+^ 374.1322; observed, 374.1323.

**4-(2-((4-Methoxybenzyl)thio)phenyl)-3-(p-tolyl)-4*H*-1,2,4-triazole (2af).** The title compound was prepared according to general procedure 1 on a 0.49 g (2.00 mmol) scale. Purification by column chromatography (silica gel, 1. dichloromethane, 2. dichloromethane/acetone 4:1, R_f_ = 0.08) gave the product as a white powder in 28% (0.329 g, 0.85 mmol) yield; m.p. 160–161 °C. ^1^H NMR (400 MHz, DMSO-*d*_6_, 298 K): δ = 8.59 (s, 1H), 7.61 (d, *J =* 7.0 Hz, 1H), 7.50 (t, *J =* 8.5 Hz, 1H), 7.45–7.39 (m, 1H), 7.32 (t, *J =* 7.0 Hz, 1H), 7.18 (d, *J =* 8.2 Hz, 2H), 7.14–7.07 (m, 4H), 6.79 (d, *J =* 8.7 Hz, 2H), 4.14–4.06 (m, 2H), 3.70 (s, 3H), 2.27 (s, 3H); ^13^C{^1^H} NMR (100 MHz, DMSO-*d*_6_, 298 K): δ = 158.4, 152.3, 145.4, 139.3, 135.3, 132.7, 130.4, 129.9, 129.1, 128.9, 128.6, 128.1, 127.5, 126.6, 123.8, 113.8, 55.0, 35.1, 20.8. HRMS (ESI) *m*/*z* calculated for [M + H]^+^ = [C_23_H_22_N_3_OS]^+^ 388.1478; observed, 388.1480.

**4-(2-((4-Methoxybenzyl)thio)phenyl)-3-(o-tolyl)-4*H*-1,2,4-triazole (2ag).** The title compound was prepared according to general procedure 1 on a 1.227 g (5.00 mmol) scale. Purification by column chromatography (silica gel, 1. dichloromethane, 2. dichloromethane/acetone 9:1, R_f_ = 0.09) gave the product as an off-white powder in 47% (0.904 g, 02.34 mmol) yield; m.p. 143–144 °C. ^1^H NMR (400 MHz, DMSO-*d*_6_, 298 K): δ = 8.65 (s, 1H), 7.51 (d, *J =* 8.4 Hz, 1H), 7.43–7.36 (m, 2H), 7.29–7.21 (m, 3H), 7.15 (d, *J =* 6.6 Hz, 2H), 7.04–6.95 (m, 2H), 6.83 (d, *J =* 8.8 Hz, 2H), 4.11 (s, 2H), 3.72 (s, 3H), 2.29 (s, 3H); ^13^C{^1^H} NMR (100 MHz, DMSO-*d*_6_, 298 K): δ = 158.5, 138.0, 135.1, 132.2, 130.5, 130.1, 130.0, 129.9, 129.7, 128.7, 128.6, 128.1, 126.3, 126.0, 125.3, 113.9, 55.1, 35.5, 20.0. HRMS (ESI) *m*/*z* calculated for [M + H]^+^ = [C_23_H_22_N_3_OS]^+^ 388.1478; observed, 388.1477.

**4-(2-((4-Methoxybenzyl)thio)phenyl)-3-(naphthalen-1-yl)-4*H*-1,2,4-triazole (2ah).** The title compound was prepared according to general procedure 1 on a 0.490 g (2.00 mmol) scale. Purification by column chromatography (silica gel, 1. dichloromethane, 2. dichloromethane/acetone 9:1, R_f_ = 0.18) gave the product as an off-white powder in 32% (0.273 g, 0.64 mmol) yield; m.p. 163–164 °C. ^1^H NMR (400 MHz, DMSO-*d*_6_, 298 K): δ = 8.78 (s, 1H), 8.10 (dd, *J =* 7.5, 2.3 Hz, 1H), 8.01–7.91 (m, 2H), 7.60–7.52 (m, 2H), 7.45 (d, *J =* 8.1 Hz, 1H), 7.41 (d, *J =* 7.8 Hz, 1H), 7.38–7.31 (m, 2H), 7.28 (d, *J =* 7.1 Hz, 1H), 7.17 (t, *J =* 8.4 Hz, 1H), 7.01 (d, *J =* 8.7 Hz, 2H), 6.77 (d, *J =* 8.7 Hz, 2H), 4.02 (s, 2H), 3.71 (s, 3H); ^13^C{^1^H} NMR (100 MHz, DMSO-*d*_6_, 298 K): δ = 158.4, 151.6, 145.2, 135.2, 133.1, 132.2, 131.4, 130.2, 130.1, 129.9, 128.6, 128.6, 128.5, 128.2, 127.9, 126.9, 126.4, 126.2, 125.7, 124.7, 123.7, 113.8, 55.0, 35.3. HRMS (ESI) *m*/*z* calculated for [M + H]^+^ = [C_26_H_22_N_3_OS]^+^ 424.1478; observed, 424.1481.

**4-(2-((4-Methoxybenzyl)thio)phenyl)-3-(4-methoxyphenyl)-4*H*-1,2,4-triazole (2ai).** The title compound was prepared according to general procedure 1 on a 2.453 g (10.00 mmol) scale. Purification by column chromatography (silica gel, 1. dichloromethane, 2. dichloromethane/acetone 4:1, R_f_ = 0.06) gave the product as a white powder in 43% (0.767 g, 1.9 mmol) yield; m.p. 180–181 °C. ^1^H NMR (400 MHz, DMSO-*d*_6_, 298 K): δ = 8.56 (s, 1H), 7.61 (d, *J =* 8.1 Hz, 1H), 7.51 (t, *J =* 7.6 Hz, 1H), 7.42 (d, *J =* 7.8 Hz, 1H), 7.32 (t, *J =* 7.6 Hz, 1H), 7.21 (d, *J =* 8.8 Hz, 2H), 7.11 (d, *J =* 8.6 Hz, 2H), 6.84 (d, *J =* 8.7 Hz, 2H), 6.79 (d, *J =* 8.7 Hz, 2H), 4.17–4.06 (m,, 2H), 3.73 (s, 3H), 3.70 (s, 3H); ^13^C{^1^H} NMR (100 MHz, DMSO-*d*_6_, 298 K): δ = 160.2, 158.4, 152.1, 145.2, 135.4, 132.7, 130.4, 129.9, 129.0, 128.8, 128.6, 128.1, 126.6, 118.9, 114.0, 113.8, 55.2, 55.0, 35.0. HRMS (ESI) *m*/*z* calculated for [M + H]^+^ = [C_23_H_22_N_3_O_2_S]^+^ 404.1427; observed, 404.1440.

**4-(2-((4-Methoxybenzyl)thio)phenyl)-3-(2-methoxyphenyl)-4*H*-1,2,4-triazole (2aj).** The title compound was prepared according to general procedure 1 on a 0.49 g (2.00 mmol) scale. Purification by column chromatography (silica gel, 1. dichloromethane, 2. dichloromethane/acetone 4:1, R_f_ = 0.03) gave the product as an off-white powder in 32% (0.259 g, 0.64 mmol) yield, m.p. 123–124 °C. ^1^H NMR (400 MHz, DMSO-*d*_6_, 298 K): δ = 8.54 (s, 1H), 7.50 (d, *J* = 7.8 Hz, 1H), 7.42–7.36 (m, 2H), 7.25–7.12 (m, 4H), 7.01–6.97 (m, 1H), 6.92 (d, *J* = 8.6 Hz, 1H), 6.84 (d, *J* = 8.7 Hz, 2H), 4.08 (s, 2H), 3.72 (s, 3H), 3.41 (s, 3H); ^13^C{^1^H} NMR (100 MHz, DMSO-*d*_6_, 298 K): δ = 158.5, 156.6, 144.7, 138.2, 134.5, 133.0, 131.9, 131.9, 130.0, 129.8, 129.1, 128.3, 127.8, 127.1, 126.1, 120.3, 115.8, 113.9, 111.8, 111.3, 55.1, 54.8, 36.0. HRMS (ESI) *m*/*z* calculated for [M + H]^+^ = [C_23_H_22_N_3_O_2_S]^+^ 404.1427; observed, 404.1428.

**4-(4-(2-((4-Methoxybenzyl)thio)phenyl)-4*H*-1,2,4-triazol-3-yl)phenol (2ak).** The title compound was prepared according to general procedure 1 on a 0.490 g (2.00 mmol) scale. Purification by column chromatography (silica gel, 1. dichloromethane, 2. dichloromethane/acetone 1:1, R_f_ = 0.18) and gave the product as an off-white powder in 49% (0.380 g, 0.98 mmol) yield; m.p. 229–230 °C. ^1^H NMR (400 MHz, DMSO-*d*_6_, 298 K): δ = 9.85 (s, 1H), 8.52 (s, 1H), 7.58 (d, *J =* 8.3 Hz, 1H), 7.50 (t, *J =* 7.8 Hz, 1H), 7.41 (d, *J =* 7.9 Hz, 1H), 7.31 (t, *J =* 7.2 Hz, 1H), 7.11 (d, *J =* 8.6 Hz, 4H), 6.79 (d, *J =* 8.6 Hz, 2H), 6.66 (d, *J =* 8.4 Hz, 2H), 4.14–4.06 (m, 2H), 3.70 (s, 3H); ^13^C{^1^H} NMR (100 MHz, DMSO-*d*_6_, 298 K): δ = 158.68, 158.42, 145.02, 135.39, 132.82, 130.35, 129.94, 129.15, 128.77, 128.58, 128.03, 126.54, 117.35, 115.33, 113.79, 55.02, 35.03, 30.70. HRMS (ESI) *m*/*z* calculated for [M + H]^+^ = [C_22_H_20_N_3_O_2_S]^+^ 390.1271; observed, 390.1274.

**4-(2-((4-Methoxybenzyl)thio)phenyl)-3-(4-nitrophenyl)-4*H*-1,2,4-triazole (2al).** The title compound was prepared according to general procedure 1 on a 2.453 g (10.00 mmol) scale. Purification by column chromatography (silica gel, 1. dichloromethane, 2. dichloromethane/acetone 4:1, R_f_ = 0.17) and gave the product as a light-brown powder in 25% (0.447 g, 1.01mmol) yield; m.p. 154–155 °C. ^1^H NMR (400 MHz, DMSO-*d*_6_, 298 K): δ = 8.78 (s, 1H), 8.13 (d, *J* = 8.9 Hz, 2H), 7.68 (d, *J* = 6.7 Hz, 1H), 7.59–7.48 (m, 4H), 7.39–7.35 (m, 1H), 7.07 (d, *J* = 8.7 Hz, 2H), 6.74 (d, *J* = 8.7 Hz, 2H), 4.17 (d, *J* = 13.0 Hz, 1H), 4.08 (d, *J* = 13.0 Hz, 1H), 3.68 (s, 3H); ^13^C{^1^H} NMR (100 MHz, DMSO-*d*_6_, 298 K): δ = 158.4, 147.8, 135.0, 132.6, 130.8, 129.9, 129.1, 128.5, 128.2, 126.9, 123.8, 113.7, 54.9, 35.0. HRMS (ESI) *m*/*z* calculated for [M + H]^+^ = [C_22_H_19_N_4_O_3_S]^+^ 419.1172; observed, 419.1171.

**4-(2-((4-Methoxybenzyl)thio)phenyl)-3-(2-nitrophenyl)-4*H*-1,2,4-triazole (2am).** The title compound was prepared according to general procedure 1 on a 1.227 g (5.00 mmol) scale. Purification by column chromatography (silica gel, 1. dichloromethane, 2. dichloromethane/acetone 4:1, R_f_ = 0.17) gave the product as a light-brown powder in 31% (0.656 g, 1.57 mmol) yield; m.p. 130–131 °C. ^1^H NMR (400 MHz, DMSO-*d*_6_, 298 K): δ = 8.78 (s, 1H), 8.04 (dd, *J =* 8.1, 1.3 Hz, 1H), 7.77–7.63 (m, 2H), 7.59 (dd, *J =* 8.1, 1.5 Hz, 1H), 7.46–7.44 (m, 1H), 7.34 (dd, *J =* 7.5, 1.7 Hz, 1H), 7.26–7.15 (m, 4H), 6.85 (d, *J =* 8.7 Hz, 2H), 4.16 (s, 2H), 3.72 (s, 3H); ^13^C{^1^H} NMR (100 MHz, DMSO-*d*_6_, 298 K): δ = 158.5, 148.6, 145.5, 135.0, 133.5, 132.0, 131.9, 131.1, 130.5, 130.0, 129.1, 128.2, 127.9, 126.6, 124.7, 120.9, 113.9, 55.1, 35.5 (one quaternary atom could not be identified with confidence). HRMS (ESI) *m*/*z* calculated for [M + H]^+^ = [C_22_H_19_N_4_O_3_S]^+^ 419.1172; observed, 419.1172.

**3-(4-Fluorophenyl)-4-(2-((4-methoxybenzyl)thio)phenyl)-4*H*-1,2,4-triazole (2an).** The title compound was prepared according to general procedure 1 on a 1.227 g (5.00 mmol) scale. Purification by column chromatography (silica gel, 1. dichloromethane, 2. dichloromethane/acetone 4:1, R_f_ = 0.07) and gave the product as an off-white powder in 34% (0.669 g, 1.70 mmol) yield; m.p. 118–119 °C. ^1^H NMR (400 MHz, DMSO-*d*_6_, 298 K): δ = 8.64 (s, 1H), 7.62 (dd, *J =* 8.1, 1.3 Hz, 1H), 7.52 (td, *J =* 7.6, 1.5 Hz, 1H), 7.48 (dd, *J =* 7.9, 1.5 Hz, 1H), 7.33 (dd, *J =* 9.0, 5.4 Hz, 3H), 7.16 (t, *J =* 9.0 Hz, 2H), 7.10 (d, *J =* 8.7 Hz, 2H), 6.79 (d, *J =* 8.7 Hz, 2H), 4.15–4.07 (m, 2H), 3.70 (s, 3H); ^13^C{^1^H} NMR (100 MHz, DMSO-*d*_6_, 298 K): δ = 162.8 (d, *J =* 250 Hz), 158.5, 151.6, 145.7, 135.3, 132.4, 130.6, 129.9 (d, *J =* 9Hz), 130.0, 128.9, 128.6, 128.1, 126.7, 123.2 (d, *J =* 3Hz), 115.7 (d, *J* = 20 Hz), 113.8, 55.0, 35.1. ^19^F{^1^H} NMR (376 MHz, DMSO-*d*_6_, 298 K, referenced to C_6_H_5_F): δ = −110.88. HRMS (ESI) *m*/*z* calculated for [M + H]^+^ = [C_22_H_19_FN_3_OS]^+^ 392.1227; observed, 392.1229.

**3-(3-Bromophenyl)-4-(2-((4-methoxybenzyl)thio)phenyl)-4*H*-1,2,4-triazole (2ao).** The title compound was prepared according to general procedure 1 on a 1.227 g (5.00 mmol) scale. Purification by column chromatography (silica gel, 1. dichloromethane, 2. dichloromethane/acetone 4:1, R_f_ = 0.19) gave the product as an off-white powder in 33% (0.756 g, 1.7 mmol) yield; m.p. 101–102 °C. ^1^H NMR (400 MHz, DMSO-*d*_6_, 298 K): δ = 8.69 (s, 1H), 7.67–7.57 (m, 2H), 7.57–7.49 (m, 3H), 7.37 (td, *J =* 7.4, 1.5 Hz, 1H), 7.29–7.20 (m, 2H), 7.09 (d, *J =* 8.7 Hz, 2H), 6.79 (d, *J =* 8.8 Hz, 2H), 4.09 (s, 2H), 3.70 (s, 3H); ^13^C{^1^H} NMR (100 MHz, DMSO-*d*_6_, 298 K): δ = 158.5, 151.0, 145.9, 135.3, 132.6, 132.3, 130.8, 130.8, 130.1, 129.9, 128.9, 128.8, 128.6, 127.9, 126.7, 126.3, 121.7, 113.9, 55.0, 35.2. HRMS (ESI) *m*/*z* calculated for [M + H]^+^ = [C_22_H_19_BrN_3_OS]^+^ 452.0427; observed, 452.0416.

**3-(3-Chlorophenyl)-4-(2-((4-methoxybenzyl)thio)phenyl)-4*H*-1,2,4-triazole (2ap).** The title compound was prepared according to general procedure 1 on a 2.453 g (10.00 mmol) scale. Purification by column chromatography (silica gel, 1. dichloromethane, 2. dichloromethane/acetone 4:1, R_f_ = 0.14) gave the product as an off-white powder in 57% (1702 g, 5.70 mmol) yield; m.p. 169–170 °C. ^1^H NMR (400 MHz, DMSO-*d*_6_, 298 K): δ = 8.70 (s, 1H), 7.61 (d, *J =* 8.8 Hz, 1H), 7.57–7.51 (m, 2H), 7.49 (d, *J =* 8.1 Hz, 1H), 7.40–7.30 (m, 3H), 7.20 (d, *J =* 7.9 Hz, 1H), 7.09 (d, *J =* 8.7 Hz, 2H), 6.78 (d, *J =* 8.6 Hz, 2H), 4.10 (s, 2H), 3.70 (s, 3H); ^13^C{^1^H} NMR (100 MHz, DMSO-*d*_6_, 298 K): δ = 158.5, 151.1, 145.9, 135.3, 133.3, 132.3, 130.7, 130.5, 129.9, 129.66, 128.9, 128.6, 127.9, 127.2, 126.7, 126.0, 113.8, 55.0, 35.2. HRMS (ESI) *m*/*z* calculated for [M + H]^+^ = [C_22_H_18_ClN_3_OS]^+^ 408.0932; observed, 408.0935.

**3-(2-Fluorophenyl)-4-(2-((4-methoxybenzyl)thio)phenyl)-4*H*-1,2,4-triazole (2aq).** The title compound was prepared according to general procedure 1 on a 1.227 g (5.00 mmol) scale. Purification by column chromatography (silica gel, 1. dichloromethane, 2. dichloromethane/acetone 4:1, R_f_ = 0.03) gave the product as an off-white powder in 29% (0.387 g, 0.94 mmol) yield; m.p. 143–144 °C. ^1^H NMR (400 MHz, DMSO-*d*_6_, 298 K): δ = 8.71 (s, 1H), 7.51 (d, *J =* 7.8 Hz, 2H), 7.44 (d, *J =* 7.3 Hz, 1H), 7.39 (d, *J =* 6.6 Hz, 1H), 7.36–7.31 (m, 1H), 7.30–7.25 (m, 1H), 7.23 (d, *J =* 9.2 Hz, 1H), 7.21–7.16 (m, 1H), 7.12 (d, *J =* 8.8 Hz, 2H), 6.82 (d, *J =* 8.7 Hz, 2H), 4.07 (s, 2H), 3.71 (s, 3H); ^13^C{^1^H} NMR (100 MHz, DMSO-*d*_6_, 298 K): δ = 159.4 (d, *J =* 250.5 Hz), 158.5, 148.7, 145.4, 134.8, 132.6 (d, *J =* 8.1 Hz), 132.2, 131.7, 130.3, 129.9, 129.1, 128.3, 128.0, 126.5, 124.5 (d, *J =* 3.5 Hz), 116.1 (d, *J =* 21.0 Hz), 114.8 (d, *J =* 14.4 Hz), 113.9, 55.1, 35.6; ^19^F{^1^H} NMR (376 MHz, DMSO-*d*_6_, 298 K, referenced to C_6_H_5_F): δ = −112.17. HRMS (ESI) *m*/*z* calculated for [M + H]^+^ = [C_22_H_19_FN_3_OS]^+^ 392.1227; observed, 392.1229.

**3-(4-(2-((4-Methoxybenzyl)thio)phenyl)-4*H*-1,2,4-triazol-3-yl)pyridine (2ar).** The title compound was prepared according to general procedure 1 on a 0.490 g (2.00 mmol) scale. Purification by column chromatography (silica gel, 1. dichloromethane, 2. dichloromethane/acetone 1:1, R_f_ = 0.28) gave the product as an off-white powder in 52% (0.392 g, 1.05 mmol) yield; m.p. 143–144 °C. ^1^H NMR (400 MHz, DMSO-*d*_6_, 298 K): δ = 8.74 (s, 1H), 8.59 (d, *J =* 4.9 Hz, 1H), 8.53 (s, 1H), 7.61 (d, *J =* 7.9 Hz, 2H), 7.58–7.48 (m, 2H), 7.42–7.29 (m, 2H), 7.10 (d, *J =* 8.7 Hz, 2H), 6.78 (d, *J =* 8.6 Hz, 2H), 4.11 (s, 2H), 3.70 (s, 3H); ^13^C{^1^H} NMR (100 MHz, DMSO-*d*_6_, 298 K): δ = 158.5, 150.6, 150.2, 147.9, 145.9, 135.3, 134.8, 132.0, 130.8, 129.9, 128.8, 128.6, 127.9, 126.7, 123.6, 123.0, 113.8, 55.0, 35.1. HRMS (ESI) *m*/*z* calculated for [M + H]^+^ = [C_21_H_19_N_4_OS]^+^ 375.1274; observed, 375.1278.

**3-(3-Chlorophenyl)-4-(2-((4-methoxybenzyl)thio)-5-(trifluoromethyl)phenyl)-4*H*-1,2,4-triazole (2bp).** The title compound was prepared according to general procedure 1 on a 1.567 g (5.00 mmol) scale. Purification by column chromatography (silica gel, 1. dichloromethane, 2. dichloromethane/methanol 19:1, R_f_ = 0.17) gave the product as an off-white powder in 35% (0.824 g, 1.73 mmol) yield, m.p. 153–154 °C. ^1^H NMR (400 MHz, DMSO-*d*_6_, 298 K): δ = 8.83 (s, 1H), 8.14 (d, *J =* 2.0 Hz, 1H), 7.88 (dd, *J =* 8.4, 2.7 Hz, 1H), 7.75 (d, *J =* 8.1 Hz, 1H), 7.52 (ddd, *J =* 8.1, 2.2, 1.1 Hz, 1H), 7.42 (t, *J =* 1.8 Hz, 1H), 7.37 (t, *J =* 7.9 Hz, 1H), 7.25–7.19 (m, 1H), 7.16–7.08 (m, 2H), 6.83–6.75 (m, 2H), 4.28–4.20 (m, 2H), 3.70 (s, 3H); ^13^C{^1^H} NMR (100 MHz, DMSO-*d*_6_, 298 K): δ = 159.0, 151.5, 146.2, 142.5, 133.7, 132.3, 131.1, 130.3, 130.3, 128.9, 128.5, 127.8, 127.7, 127.6, 126.8 (q, *J* = 33.4 Hz), 126.5, 126.3 (q, *J* = 3.9 Hz), 124.0 (q, *J* = 272.2 Hz), 114.4, 55.5, 34.7; ^19^F{^1^H} NMR (376 MHz, DMSO-*d*_6_, 298 K, referenced to C_6_H_5_F): δ = −60.92. HRMS (ESI) *m*/*z* calculated for [M + H]^+^ = [C_23_H_18_ClF_3_N_3_OS]^+^ 476.0806; observed, 476.0809.

**4-(5-Methoxy-2-((4-methoxybenzyl)thio)phenyl)-3-(methoxymethyl)-4*H*-1,2,4-triazole (2cd).** The title compound was prepared according to general procedure 1 on a 1.377 g (5.00 mmol) scale. Purification by column chromatography (silica gel, 1. dichloromethane, 2. dichloromethane/acetone 1:1, R_f_ = 0.25) gave the product as a white powder in 49% (0.903 g, 2.43 mmol) yield; m.p. 103–104 °C. ^1^H NMR (400 MHz, DMSO-*d*_6_, 298 K): δ = 8.43 (s, 1H), 7.57 (d, *J =* 7.9 Hz, 1H), 7.12 (d, *J =* 8.1 Hz, 2H), 7.04 (d, *J =* 8.7 Hz, 2H), 6.82 (d, *J =* 8.7 Hz, 2H), 4.24 (s, 2H), 3.94 (s, 2H), 3.78 (s, 3H), 3.71 (s, 3H), 3.09 (s, 3H); ^13^C{^1^H} NMR (100 MHz, DMSO-*d*_6_, 298 K): δ = 158.9, 158.4, 150.3, 145.2, 143.3, 135.2, 134.1, 129.9, 129.0, 123.7, 116.4, 113.8, 62.7, 57.6, 55.8, 55.1, 38.0. HRMS (ESI) *m*/*z* calculated for [M + H]^+^ = [C_19_H_22_N_3_O_3_S]^+^ 372.1376; observed, 372.1374.

**3-(4-Fluorophenyl)-4-(5-methoxy-2-((4-methoxybenzyl)thio)phenyl)-4*H*-1,2,4-triazole (2cn).** The title compound was prepared according to general procedure 1 on a 1.377 g (5.00 mmol) scale. Purification by column chromatography (silica gel, 1. dichloromethane, 2. dichloromethane/acetone 4:1, R_f_ = 0.20) gave the product as a brown powder in 43% (0.905 g, 2.2 mmol) yield; m.p. 143–144 °C. ^1^H NMR (400 MHz, DMSO-*d*_6_, 298 K): δ = 8.53 (s, 1H), 7.50 (d, *J =* 8.8 Hz, 1H), 7.40–7.33 (m, 2H), 7.24 (d, *J =* 2.8 Hz, 1H), 7.19 (t, *J =* 8.9 Hz, 2H), 7.12 (dd, *J =* 8.9, 2.9 Hz, 1H), 6.98 (d, *J =* 8.7 Hz, 2H), 6.78 (d, *J =* 8.8 Hz, 2H), 3.85 (s, 2H), 3.76 (s, 3H), 3.68 (s, 3H); ^13^C{^1^H} NMR (100 MHz, DMSO-*d*_6_, 298 K): δ = 162.7 (d, *J =* 247.6 Hz), 158.8, 158.4, 151.7, 145.6, 135.3, 133.3, 129.9 (d, *J* = 9.2 Hz), 129.9, 128.7, 124.3, 123.4 (d, *J* = 2.9 Hz), 116.7, 115.7 (d, *J* = 22.0 Hz), 114.30 113.7, 55.8, 55.0, 37.2; ^19^F{^1^H} NMR (376 MHz, DMSO-*d*_6_, 298 K, referenced to C_6_H_5_F): δ = −111.07. HRMS (ESI) *m*/*z* calculated for [M + H]^+^ = [C_23_H_21_FN_3_O_2_S]^+^ 422.1333; observed, 422.1336.

**4-(5-Bromo-2-((4-methoxybenzyl)thio)phenyl)-3-(4-methoxyphenyl)-4*H*-1,2,4-triazole (2di).** The title compound was prepared according to general procedure 1 on a 1.615 g (5.00 mmol) scale. Purification by column chromatography (silica gel, 1. dichloromethane, 2. dichloromethane/acetone 4:1; R_f_ = 0.11) gave the product as an off-white powder in 29% (0.691 g, 1.43 mmol) yield, m.p. 199–200 °C. ^1^H NMR (400 MHz, DMSO-*d*_6_, 298 K): δ = 8.60 (s, 1H), 7.84 (d, *J =* 2.2 Hz, 1H), 7.70 (dd, *J =* 8.5, 2.3 Hz, 1H), 7.52 (d, *J =* 8.6 Hz, 1H), 7.23 (d, *J =* 8.9 Hz, 2H), 7.09 (d, *J =* 8.7 Hz, 2H), 6.89 (d, *J =* 8.9 Hz, 2H), 6.79 (d, *J =* 8.8 Hz, 2H), 4.13 (s, 2H), 3.75 (s, 3H), 3.70 (s, 3H); ^13^C{^1^H} NMR (100 MHz, DMSO-*d*_6_, 298 K): δ = 160.3, 158.5, 152.0, 145.1, 135.3, 133.9, 133.2, 131.3, 130.2, 129.9, 129.1, 127.8, 118.7, 118.3, 114.1, 113.8, 55.2, 55.0, 34.9. HRMS (ESI) *m*/*z* calculated for [M + H]^+^ = [C_23_H_21_BrN_3_O_2_S]^+^ 482.0532; observed, 482.0538.

**3-(4-(5-Bromo-2-((4-methoxybenzyl)thio)phenyl)-4*H*-1,2,4-triazol-3-yl)pyridine (2dr).** The title compound was prepared according to general procedure 1 on a 1.615 g (5.00 mmol) scale. Purification by column chromatography (silica gel, 1. dichloromethane, 2. dichloromethane/acetone 1:1, R_f_ = 0.31) gave the product as a white powder in 37% (0.845 g, 1.86 mmol) yield; m.p. 199–200 °C. ^1^H NMR (400 MHz, DMSO-*d*_6_, 298 K): δ = 8.77 (s, 1H), 8.62 (dd, *J =* 4.8, 1.7 Hz, 1H), 8.56 (d, *J =* 2.3 Hz, 1H), 7.96 (d, *J =* 2.2 Hz, 1H), 7.73 (dd, *J =* 8.6, 2.3 Hz, 1H), 7.64 (dt, *J =* 7.9, 1.9 Hz, 1H), 7.52 (d, *J =* 8.7 Hz, 1H), 7.39 (dd, *J =* 8.0, 4.8 Hz, 1H), 7.07 (d, *J =* 8.8 Hz, 2H), 6.78 (d, *J =* 8.8 Hz, 2H), 4.10 (s, 2H), 3.70 (s, 3H); ^13^C{^1^H} NMR (100 MHz, DMSO-*d*_6_, 298 K): δ =159.0, 151.2, 150.6, 148.5, 146.3, 135.6, 135.4, 134.0, 133.6, 131.8, 130.8, 130.4, 128.1, 124.2, 123.3, 119.0, 114.4, 55.5, 35.5. HRMS (ESI) *m*/*z* calculated for [M + H]^+^ = [C_21_H_19_BrN_4_OS]^+^ 453.0379; observed, 453.0379.

**4-(4-Bromo-2-((4-methoxybenzyl)thio)phenyl)-3-phenyl-4*H*-1,2,4-triazole (2ee).** The title compound was prepared according to general procedure 1 on a 1.615 g (5.00 mmol) scale. Purification by column chromatography (silica gel, 1. dichloromethane, 2. dichloromethane/acetone 4:1, R_f_ = 0.11) gave the product as an off-white powder in 40% (0.905 g, 2.00 mmol) yield; m.p. 157–159 °C. ^1^H NMR (400 MHz, DMSO-*d*_6_, 298 K): δ = 8.66 (s, 1H), 7.75 (d, *J =* 2.1 Hz, 1H), 7.52 (dd, *J =* 8.4, 2.1 Hz, 1H), 7.46–7.40 (m, 2H), 7.38–7.29 (m, 4H), 7.12 (d, *J =* 8.7 Hz, 2H), 6.81 (d, *J =* 8.7 Hz, 2H), 4.22–4.14 (m, 2H), 3.71 (s, 3H); ^13^C{^1^H} NMR (100 MHz, DMSO-*d*_6_, 298 K): δ = 158.5, 152.3, 145.5, 138.2, 131.6, 130.7, 130.3, 130.2, 130.0, 129.8, 129.3, 128.7, 127.6, 127.5, 126.4, 123.6, 113.9, 55.0, 34.9. HRMS (ESI) *m*/*z* calculated for [M + H]^+^ = [C_22_H_19_BrN_3_OS]^+^ 452.0427; observed, 452.0432.

**4-(3-Chloro-2-((4-methoxybenzyl)thio)phenyl)-3-(4-nitrophenyl)-4*H*-1,2,4-triazole (2fl).** The title compound was prepared according to general procedure 1 on a 1.377 g (5.00 mmol) scale. Purification by column chromatography (silica gel, 1. dichloromethane, 2. dichloromethane/acetone 4:1, R_f_ = 0.15) gave the product as a brown powder in 27% (0.905 g, 2.1 mmol) yield; m.p. 183–184 °C. ^1^H NMR (400 MHz, DMSO-*d*_6_, 298 K): δ = 8.40 (s, 1H), 8.18 (d, *J =* 8.9 Hz, 2H), 7.87 (dd, *J =* 7.8, 1.7 Hz, 1H), 7.70–7.58 (m, 2H), 7.47 (d, *J =* 9.0 Hz, 2H), 6.86 (d, *J =* 8.7 Hz, 2H), 6.74 (d, *J =* 8.8 Hz, 2H), 3.91 (d, *J =* 12.3 Hz, 1H), 3.79 (d, *J =* 12.2 Hz, 1H), 3.67 (s, 3H); ^13^C{^1^H} NMR (100 MHz, DMSO-*d*_6_, 298 K): δ = 158.6, 151.1, 147.8, 146.4, 140.5, 138.8, 132.5, 132.1, 131.4, 130.8, 129.9, 128.7, 128.4, 127.7, 123.9, 113.9, 55.0, 37.6. HRMS (ESI) *m*/*z* calculated for [M + H]^+^ = [C_22_H_18_ClN_4_O_3_S]^+^ 453.0783; observed, 453.0785.

**3-(3-(2-Fluorophenyl)-4*H*-1,2,4-triazol-4-yl)-4-((4-methoxybenzyl)thio)aniline (2gq).** The title compound was prepared according to general procedure 1 on a 1.302 g (5.00 mmol) scale. Purification by column chromatography (silica gel, 1. dichloromethane, 2. dichloromethane/acetone 4:1, R_f_ = 0.05) gave the product as a brown powder in 48% (0.972 g, 2.39 mmol) yield; m.p. 78–79 °C. ^1^H NMR (400 MHz, DMSO-*d*_6_, 298 K): δ = 8.92 (s, 1H), 7.62–7.52 (m, 2H), 7.38–7.24 (m, 2H), 7.12–7.01 (m, 3H), 6.79 (d, *J =* 8.8 Hz, 2H), 6.59 (d, *J =* 2.3 Hz, 1H), 6.27 (dd, *J =* 8.1, 2.4 Hz, 1H), 5.57 (s, 2H), 3.91 (s, 2H), 3.71 (s, 3H); ^13^C{^1^H} NMR (100 MHz, DMSO-*d*_6_, 298 K): δ = 159.2 (d, *J* = 252.0 Hz), 158.2, 149.7, 148.1, 144.9, 135.0, 134.5, 132.7 (d, *J =* 8.4 Hz), 131.9 (d, *J =* 2.2 Hz), 130.0, 129.5, 124.8 (d, *J =* 3.7 Hz), 116.9, 116.0 (d, *J =* 20.9 Hz), 115.2 (d, *J =* 14.7 Hz), 113.6, 112.0, 109.6, 55.0, 36.7; ^19^F{^1^H} NMR (376 MHz, DMSO-*d*_6_, 298 K, referenced to C_6_H_5_F): δ = −112.66. HRMS (ESI) *m*/*z* calculated for [M + H]^+^ = [C_22_H_20_FN_4_OS]^+^ 407.1336; observed, 407.1347.

**4-(2-((4-Methoxybenzyl)thio)-3-methylphenyl)-3-(2-nitrophenyl)-4*H*-1,2,4-triazole (2hm).** The title compound was prepared according to general procedure 1 on a 1.296 g (5.00 mmol) scale. Purification by column chromatography (silica gel, 1. dichloromethane, 2. dichloromethane/acetone 4:1), gave the (crude) product as a brown powder of 0.115 g yield.

**4-(3-Fluoro-2-((4-methoxybenzyl)thio)phenyl)-3-(p-tolyl)-4*H*-1,2,4-triazole (2if).** The title compound was prepared according to general procedure 1 on a 1.317 g (5.00 mmol) scale. Purification by column chromatography (silica gel, 1. dichloromethane, 2. dichloromethane/acetone 4:1, R_f_ = 0.12) gave the product as an off-white powder in 35% (0.702 g, 1.73 mmol) yield; m.p. 114–116 °C. ^1^H NMR (400 MHz, DMSO-*d*_6_, 298 K): δ = 8.32 (s, 1H), 7.59–7.48 (m, 2H), 7.40–7.38 (m, 1H), 7.15–7.02 (m, 4H), 6.91 (d, *J =* 8.6 Hz, 2H), 6.76 (d, *J =* 8.2 Hz, 2H), 3.90–3,74 (m, 2H), 3.69 (s, 3H), 2.27 (s, 3H); ^13^C{^1^H} NMR (100 MHz, DMSO-*d*_6_, 298 K): δ = 162.8 (d, *J* = 246.9 Hz), 158.6, 152.7, 145.3, 139.4, 137.4 (d, *J* = 3.9 Hz), 130.9 (d, *J* = 10.6 Hz), 129.9, 129.2, 128.7, 127.6, 124.8, 123.6, 120.3 (d, *J* = 20.5 Hz), 117.6 (d, *J* = 23.8 Hz), 55.0, 37.3, 20.8; ^19^F{^1^H} NMR (376 MHz, DMSO-*d*_6_, 298 K, referenced to C_6_H_5_F): δ = −102.98. HRMS (ESI) *m*/*z* calculated for [M + H]^+^ = [C_23_H_21_FN_3_OS]^+^ 406.1384; observed, 406.1389.

**Ethyl 3-(3-Cyclopropyl-4*H*-1,2,4-triazol-4-yl)-4-((4-methoxybenzyl)thio)benzoate (2jc).** The title compound was prepared according to general procedure 1 on a 0.794 g (2.50 mmol) scale. Purification by column chromatography (silica gel, 1. dichloromethane, 2. dichloromethane/acetone 4:1, R_f_ = 0.14) gave the product as a yellow powder in 33% (0.338 g, 0.83 mmol) yield; m.p. 151–152 °C. ^1^H NMR (400 MHz, DMSO-*d*_6_, 298 K): δ = 8.48 (s, 1H), 8.06 (dd, *J =* 8.4, 1.9 Hz, 1H), 7.92 (d, *J =* 2.0 Hz, 1H), 7.79 (d, *J =* 8.6 Hz, 1H), 7.29 (d, *J =* 6.5 Hz, 2H), 6.86 (d, *J =* 8.8 Hz, 2H), 4.46–4.03 (m, 4H), 3.71 (s, 3H), 1.45–1.35 (m, 1H), 1.31 (t, *J =* 7.2 Hz, 3H), 0.90 (d, *J =* 2.9 Hz, 2H), 0.82 (dd, *J =* 8.4, 2.5 Hz, 2H); ^13^C{^1^H} NMR (100 MHz, DMSO-*d*_6_, 298 K): δ =164.6, 158.7, 143.1, 131.0, 130.6, 130.2, 130.2, 128.7, 127.3, 114.0, 114.0, 61.2, 55.1, 34.6, 14.1, 7.4, 4.8. HRMS (ESI) *m*/*z* calculated for [M + H]^+^ = [C_22_H_24_N_3_O_3_S]^+^ 410.1533; observed, 410.1536.

**3-(3-Bromophenyl)-4-(5-chloro-2-((4-methoxybenzyl)thio)phenyl)-4*H*-1,2,4-triazole (2ko).** The title compound was prepared according to general procedure 1 on a 0.699 g (2.50 mmol) scale. Purification by column chromatography (silica gel, 1. dichloromethane, 2. dichloromethane/acetone 4:1, R_f_ = 0.07) gave the product as a yellow powder in 45% (0.546 g, 1.12 mmol) yield; m.p. 179–180 °C. ^1^H NMR (400 MHz, DMSO-*d*_6_, 298 K): δ = 8.72 (s, 1H), 7.85 (d, *J =* 2.2 Hz, 1H), 7.68–7.51 (m, 4H), 7.29 (t, *J =* 7.9 Hz, 1H), 7.22 (d, *J =* 7.9 Hz, 1H), 7.06 (d, *J =* 8.7 Hz, 2H), 6.78 (d, *J =* 8.8 Hz, 2H), 4.09 (s, 2H), 3.70 (s, 3H); ^13^C{^1^H} NMR (100 MHz, DMSO-*d*_6_, 298 K): δ = 158.5, 150.9, 145.7, 134.6, 133.3, 132.7, 130.8, 130.6, 130.6, 130.3, 130.2, 129.8, 128.6, 128.6, 127.6, 126.3, 121.7, 113.9, 55.0, 35.2. HRMS (ESI) *m*/*z* calculated for [M + H]^+^ = [C_22_H_18_BrClN_3_OS]^+^ 486.0037; observed, 486.0043.

**Ethyl 4-(3-(4-hydroxyphenyl)-4*H*-1,2,4-triazol-4-yl)-3-((4-methoxybenzyl)thio)benzoate (2lk).** The title compound was prepared according to general procedure 1 on a 1.587 g (5.00 mmol) scale. Purification by column chromatography (silica gel, 1. dichloromethane, 2. dichloromethane/acetone 1:1, R_f_ = 0.31) gave the (crude) product as a brown powder in 0.150 g yield.

**3-(3-Bromophenyl)-4-(2-((4-methoxybenzyl)thio)-5-(methylsulfonyl)phenyl)-4*H*-1,2,4-triazole (2mo).** The title compound was prepared according to general procedure 1 on a 1.617 g (5.00 mmol) scale. Purification by column chromatography (silica gel, 1. dichloromethane, 2. dichloromethane/acetone 9:1, R_f_ = 0.11) gave the product as a white powder in 28% (0.762 g, 1.40 mmol) yield, m.p. 233–234 °C. ^1^H NMR (400 MHz, DMSO-*d*_6_, 298 K): δ = 8.83 (s, 1H), 8.22 (d, *J =* 2.1 Hz, 1H), 8.00 (dd, *J =* 8.4, 2.1 Hz, 1H), 7.78 (d, *J =* 8.4 Hz, 1H), 7.66 (dt, *J =* 7.6, 1.8 Hz, 1H), 7.61 (d, *J =* 1.7 Hz, 1H), 7.35–7.21 (m, 2H), 7.12 (d, *J =* 8.8 Hz, 2H), 6.80 (d, *J =* 8.8 Hz, 2H), 4.25 (s, 2H), 3.71 (s, 3H), 3.25 (s, 3H); ^13^C{^1^H} NMR (100 MHz, DMSO-*d*_6_, 298 K): δ = 158.6, 150.9, 145.6, 143.5, 137.9, 132.8, 130.9, 130.4, 129.9, 128.6, 128.5, 127.6, 127.3, 127.0, 126.4, 121.7, 114.0, 55.1, 43.4, 34.1. HRMS (ESI) *m*/*z* calculated for [M + H]^+^ = [C_23_H_21_BrN_3_O_3_S_2_]^+^ 530.0202; observed, 530.0208.

**(4-((4-Methoxybenzyl)thio)-3-(3-(naphthalen-1-yl)-4*H*-1,2,4-triazol-4-yl)phenyl)methanol. (2nh).** The title compound was prepared according to general procedure 1 on a 1.376 g (5.00 mmol) scale. While traces of the product were detected by LCMS and purification was attempted by column chromatography (silica gel, 1. dichloromethane, 2. dichloromethane/acetone 1:1), the product could not be isolated.

**3-(4-(2-((4-Methoxybenzyl)thio)-5-(trifluoromethoxy)phenyl)-4*H*-1,2,4-triazol-3-yl)pyridine (2or).** The title compound was prepared according to general procedure 1 on a 1.647 g (5.00 mmol) scale. Purification by column chromatography (silica gel, 1. dichloromethane, 2. dichloromethane/acetone 1:1, R_f_ = 0.13) gave the product as an off-white powder in 10% (0.231 g, 0.50 mmol) yield; m.p. 147–148 °C. ^1^H NMR (400 MHz, DMSO-*d*_6_, 298 K): δ = 8.83 (s, 1H), 8.61 (d, *J =* 4.9 Hz, 1H), 8.54 (s, 1H), 7.83 (d, *J =* 3.2 Hz, 1H), 7.70 (d, *J =* 8.9 Hz, 1H), 7.64–7.55 (m, 2H), 7.37 (dd, *J =* 8.0, 4.1 Hz, 1H), 7.10 (d, *J =* 8.8 Hz, 2H), 6.79 (d, *J =* 8.7 Hz, 2H), 4.15 (s, 2H), 3.70 (s, 3H); ^13^C{^1^H} NMR (100 MHz, DMSO-*d*_6_, 298 K): δ = 158.5, 150.7, 150.2, 148.1, 145.9 (q, *J* = 1.8 Hz), 145.8, 135.3, 135.1, 132.8, 130.1, 129.9, 127.6, 123.6, 123.5, 122.7, 122.2, 119.9 (q, *J* = 257.5 Hz), 113.9, 55.0, 35.2. ^19^F{^1^H} NMR (376 MHz, DMSO-*d*_6_, 298 K, referenced to C_6_H_5_F): δ = −57.00. HRMS (ESI) *m*/*z* calculated for [M + H]^+^ = [C_22_H_18_F_3_N_4_O_2_S]^+^ 459.1097; observed, 459.1102.

**4-((4-Methoxybenzyl)thio)-3-(3-(pyridin-2-yl)-4*H*-1,2,4-triazol-4-yl)benzenesulfonamide (2pr).** The title compound was prepared according to general procedure 1 on a 1.622 g (5.00 mmol) scale. Purification by column chromatography (silica gel, 1. dichloromethane, 2. dichloromethane/acetone 9:1, R_f_ = 0.04) yielded the product as a white powder in 14% (0.317 g, 0.70 mmol) yield; m.p. 220–221 °C. ^1^H NMR (400 MHz, DMSO-*d*_6_, 298 K): δ = 8.86 (s, 1H), 8.63 (d, *J =* 3.2 Hz, 1H), 8.55 (s, 1H), 7.97 (d, *J =* 2.1 Hz, 1H), 7.91 (dd, *J =* 8.4, 2.1 Hz, 1H), 7.78 (d, *J =* 8.6 Hz, 1H), 7.68 (dt, *J =* 8.1, 2.0 Hz, 1H), 7.51 (s, 2H), 7.38 (dd, *J =* 8.1, 4.9 Hz, 1H), 7.13 (d, *J =* 8.7 Hz, 2H), 6.79 (d, *J =* 8.8 Hz, 2H), 4.28–4.20 (m, 2H), 3.70 (s, 3H); ^13^C{^1^H} NMR (100 MHz, DMSO-*d*_6_, 298 K): δ = 158.6, 150.8, 150.1, 147.8, 145.8, 141.7, 140.9, 134.9, 131.1, 129.9, 127.9, 127.6, 127.2, 125.7, 123.7, 122.7, 113.9, 55.1, 34.3. HRMS (ESI) *m*/*z* calculated for [M + H]^+^ = [C_21_H_20_N_5_O_3_S_2_]^+^ 454.1002; observed, 454.1008.

#### 4.3.2. General Procedure 2 for the Preparation of the Tricyclic Heteroarenes

***Deprotection:*** A 15 mL round-bottomed flask fitted with a stir bar was loaded with the appropriate triazole (1 equiv.), dry dichloromethane (5–8 mL), and anisole (5 equiv.) and set under an argon atmosphere. After cooling to 0 °C in an ice bath, trifluoroacetic acid (5 equiv.) was added to the reaction mixture which was kept in an ice bath for 4 h or until the reaction was shown to be complete by LCMS analysis. Upon completion of the deprotection, the solvent was removed on a rotary evaporator and the reaction mixture was used without further purification in the next step. ***Oxidative Cyclization****:* DMSO (2 mL) was added to the crude thiol and the reaction mixture was stirred at 100 °C until of the cyclization reaction was complete (typically less than 4 h), as determined by samples taken and analyzed by LCMS. After cooling to room temperature, water was added to the reaction mixture and the aqueous phase was extracted three times with dichloromethane. The organic phases were combined and dried over MgSO_4_, filtered, and concentrated. The resulting crude product was purified by column chromatography as described below.

**Benzo[4,5]thiazolo[2,3-*c*][1,2,4]triazole (4aa).** This molecule has been previously synthesized, isolated and characterized by our research group [39].

**3-Methylbenzo[4,5]thiazolo[2,3-*c*][1,2,4]triazole (4ab).** The title compound was prepared according to general procedure 2 on a 0.311 g (0.50 mmol) scale. Purification by column chromatography (silica gel, 1. dichloromethane, 2. dichloromethane/methanol 1:1, R_f_ = 0.42) gave the product as an orange powder in 96% (0.091 g, 0.48 mmol) yield, m.p. 157–158 °C (Lit. 156 °C [51]). ^1^H NMR (400 MHz, DMSO-*d*_6_, 298 K): δ = 8.03 (dd, *J =* 8.0, 1.5 Hz, 1H), 7.96 (dd, *J =* 8.1, 1.7 Hz, 1H), 7.57 (td, *J =* 7.8, 1.3 Hz, 1H), 7.48 (td, *J =* 7.8, 1.2 Hz, 1H), 2.83 (s, 3H); ^13^C{^1^H} NMR (100 MHz, DMSO-*d*_6_, 298 K): δ = 154.3, 146.3, 131.4, 129.6, 126.9, 126.0, 125.4, 114.3, 11.8. HRMS (ESI) *m*/*z* calculated for [M + H]^+^ = [C_9_H_8_N_3_S]^+^ 190.0433; observed, 190.0435.

**3-Cyclopropylbenzo[4,5]thiazolo[2,3-*c*][1,2,4]triazole (4ac).** The title compound was prepared according to general procedure 2 on a 0.168 g (0.50 mmol) scale. Purification was attempted by column chromatography (silica gel, 1. dichloromethane, 2. gradient of 5–50% acetone in dichloromethane) but only yielded trace amounts of product.

**3-(Methoxymethyl)benzo[4,5]thiazolo[2,3-*c*][1,2,4]triazole (4ad).** The title compound was prepared according to general procedure 2 on a 0.166 g (0.50 mmol) scale. Purification by column chromatography (silica gel, 1. dichloromethane, 2. dichloromethane/acetone 9:1, R_f_ = 0.14) gave the product as a brown powder in 74% (0.081 g, 0.37 mmol) yield; m.p. 86–87 °C. ^1^H NMR (400 MHz, DMSO-*d*_6_, 298 K): δ = 8.07 (d, *J =* 8.1 Hz, 1H), 7.93 (d, *J =* 9.8 Hz, 1H), 7.61 (td, *J =* 7.8, 1.3 Hz, 1H), 7.56–7.48 (m, 1H), 4.99 (s, 2H), 3.36 (s, 3H); ^13^C{^1^H} NMR (100 MHz, DMSO-*d*_6_, 298 K): δ = 157.1, 149.1, 147.8, 133.5, 132.2, 131.0, 130.1, 127.4, 126.2, 124.7, 114.9. HRMS (ESI) *m*/*z* calculated for [M + H]^+^ = [C_10_H_10_N_3_OS]^+^ 220.0539; observed, 220.0537.

**3-Phenylbenzo[4,5]thiazolo[2,3-*c*][1,2,4]triazole (4ae).** The title compound was prepared according to general procedure 2 on a 0.373 g (1.00 mmol) scale. Purification by column chromatography (silica gel, 1. dichloromethane, 2. dichloromethane/acetone 19:1, R_f_ = 0.03) gave the product as an orange powder in 88% (0.221 g, 0.88 mmol) yield; m.p. 154–155 °C (Lit. 153 °C [51]). ^1^H NMR (400 MHz, DMSO-*d*_6_, 298 K): δ = 8.08 (dd, *J =* 7.5, 1.3 Hz, 1H), 7.84 (dd, *J =* 7.0, 2.6 Hz, 2H), 7.69–7.63 (m, 3H), 7.51–7.38 (m, 3H); ^13^C{^1^H} NMR (100 MHz, DMSO-*d*_6_, 298 K): δ = 155.6, 148.8, 131.8, 130.7, 129.7, 129.2, 129.1, 126.8, 126.7, 126.4, 113.9. HRMS (ESI) *m*/*z* calculated for [M + H]^+^ = [C_14_H_10_N_3_S]^+^ 252.0590; observed, 252.0591.

**3-(p-Tolyl)benzo[4,5]thiazolo[2,3-*c*][1,2,4]triazole (4af).** The title compound was prepared according to general procedure 2 on a 0.388 g (1.00 mmol) scale. Purification by column chromatography (silica gel, 1. dichloromethane, 2. dichloromethane/acetone 19:1, R_f_ = 0.20) gave the product as an orange powder in 82% (0.218 g, 0.82 mmol) yield; m.p. 150–151 °C (Lit. 154–156 °C [38]). ^1^H NMR (400 MHz, DMSO-*d*_6_,, 298 K): δ = 8.08 (dd, *J =* 6.2, 1.7 Hz, 1H), 7.72 (d, *J =* 8.1 Hz, 2H), 7.47 (d, *J =* 7.3 Hz, 3H), 7.45–7.41 (m, 2H), 2.46 (s, 3H); ^13^C{^1^H} NMR (100 MHz, DMSO-*d*_6_, 298 K): δ = 148.8, 140.5, 131.7, 129.7, 129.6, 129.0, 127.6, 126.7, 126.3, 125.7, 123.9, 113.8, 21.1. HRMS (ESI) *m*/*z* calculated for [M + H]^+^ = [C_15_H_12_N_3_S]^+^ 266.0746; observed, 266.0745.

**3-(o-Tolyl)benzo[4,5]thiazolo[2,3-*c*][1,2,4]triazole (4ag).** The title compound was prepared according to general procedure 2 on a 0.194 g (0.50 mmol) scale. Purification by column chromatography (silica gel, 1. dichloromethane, 2. dichloromethane/acetone 9:1, R_f_ = 0.16) gave the product as an orange powder in 81% (0.107 g, 0.40 mmol) yield; m.p. 112–113 °C (Lit. 110–112 °C [37]). ^1^H NMR (400 MHz, DMSO-*d*_6_, 298 K): δ = 8.08 (dd, *J =* 7.8, 1.6 Hz, 1H), 7.63–7.56 (m, 2H), 7.52 (d, *J =* 8.3 Hz, 1H), 7.46 (td, *J =* 7.8, 1.3 Hz, 2H), 7.39 (td, *J =* 7.8, 1.3 Hz, 1H), 6.88 (d, *J =* 7.2 Hz, 1H), 2.18 (s, 3H); ^13^C{^1^H} NMR (100 MHz, DMSO-*d*_6_, 298 K): δ = 155.6, 147.9, 138.3, 132.3, 131.5, 131.2, 130.9, 129.9, 127.4, 126.9, 126.8, 126.3, 113.6, 19.7. HRMS (ESI) *m*/*z* calculated for [M + H]^+^ = [C_15_H_12_N_3_S]^+^ 266.0746; observed, 266.0745.

**3-(Naphthalen-1-yl)benzo[4,5]thiazolo[2,3-*c*][1,2,4]triazole (4ah).** The title compound was prepared according to general procedure 2 on a 0.212 g (0.50 mmol) scale. Purification by column chromatography (silica gel, 1. dichloromethane, 2. dichloromethane/methanol 19:1, R_f_ = 0.43) gave the product as a yellow powder in 72% (0.108 g, 0.36 mmol) yield; m.p. 197–198 °C. ^1^H NMR (400 MHz, DMSO-*d*_6_, 298 K): δ = 8.29 (d, *J =* 8.2 Hz, 1H), 8.15 (d, *J =* 7.7 Hz, 1H), 8.08 (d, *J =* 8.1 Hz, 1H), 7.93 (d, *J =* 8.3 Hz, 1H), 7.82–7.73 (m, 1H), 7.64 (t, *J =* 7.6 Hz, 2H), 7.52 (t, *J =* 7.6 Hz, 1H), 7.40 (t, *J =* 7.2 Hz, 1H), 7.21 (t, *J =* 8.4 Hz, 1H), 6.56 (d, *J =* 8.2 Hz, 1H); ^13^C{^1^H} NMR (100 MHz, DMSO-*d*_6_, 298 K): δ = 156.2, 147.2, 133.6, 132.4, 131.8, 131.6, 129.9, 129.8, 129.2, 128.2, 127.4, 127.1, 126.8, 126.1, 126.0, 125.3, 124.3, 114.1. HRMS (ESI) *m*/*z* calculated for [M + H]^+^ = [C_18_H_12_N_3_S]^+^ 302.0746; observed, 302.0705.

**3-(4-Methoxyphenyl)benzo[4,5]thiazolo[2,3-*c*][1,2,4]triazole (4ai).** The title compound was prepared according to general procedure 2 on a 0.403 g (1.00 mmol) scale. Purification by column chromatography (silica gel, 1. dichloromethane, 2. dichloromethane/acetone 19:1, R_f_ = 0.07) gave the product as a pale-yellow powder in 82% (0.230 g, 0.82 mmol) yield; m.p. 145–147 °C (Lit. 145 °C [51] 145–146 °C [52]). ^1^ H NMR (400 MHz, DMSO-*d*_6_, 298 K): δ = 8.07 (dd, *J =* 6.7, 2.4 Hz, 1H), 7.76 (d, *J =* 8.9 Hz, 2H), 7.51–7.35 (m, 3H), 7.21 (d, *J =* 8.9 Hz, 2H), 3.89 (s, 3H); ^13^C{^1^H} NMR (100 MHz, DMSO-*d*_6_, 298 K): δ = 161.0, 155.3, 148.7, 131.7, 130.7, 129.8, 126.7, 126.3, 125.7, 118.8, 114.5, 113.8, 55.4. HRMS (ESI) *m*/*z* calculated for [M + H]^+^ = [C_15_H_12_N_3_OS]^+^ 282.0696; observed, 282.0697.

**3-(2-Methoxyphenyl)benzo[4,5]thiazolo[2,3-c][1,2,4]triazole (4aj).** The title compound was prepared according to general procedure 2 on a 0.202 g (0.500 mmol) scale. Purification by column chromatography (silica gel, 1. dichloromethane, 2. dichloromethane/acetone 19:1, R_f_ = 0.13) gave the product as an orange powder in 71% (0.100 g, 0.355 mmol) yield; m.p. 168–169 °C. ^1^H NMR (400 MHz, DMSO-*d*_6_, 298 K): δ = 8.09–8.02 (m, 1H), 7.69 (td, *J =* 8.0, 1.7 Hz, 1H), 7.60 (dd, *J =* 7.5, 1.7 Hz, 1H), 7.48–7.41 (m, 2H), 7.33 (d, *J =* 8.4 Hz, 1H), 7.20 (t, *J =* 7.5 Hz, 1H), 7.06–6.99 (m, 1H), 3.72 (s, 3H); ^13^C{^1^H} NMR (100 MHz, DMSO-*d*_6_, 298 K): δ = 157.2, 132.9, 131.7, 131.5, 129.6, 126.9, 126.3, 125.4, 121.0, 115.5, 114.1, 111.8, 55.5. HRMS (ESI) *m*/*z* calculated for [M + H]^+^ = [C_15_H_12_N_3_OS]^+^ 282.0696; observed, 282.0699.

**4-(Benzo[4,5]thiazolo[2,3-*c*][1,2,4]triazol-3-yl)phenol (4ak).** The title compound was prepared according to general procedure 2 on a 0.195 g (0.50 mmol) scale. Purification by column chromatography (silica gel, 1. dichloromethane, 2. A gradient of 5–50% acetone in dichloromethane) yielded the product in trace amounts, as confirmed by LCMS.

**3-(4-Nitrophenyl)benzo[4,5]thiazolo[2,3-*c*][1,2,4]triazole (4al).** The title compound was prepared according to general procedure 2 on a 0.418 g (1.00 mmol) scale. Purification by column chromatography (silica gel, 1. dichloromethane, 2. dichloromethane/acetone 19:1, R_f_ = 0.06) gave the product as a pale-yellow powder in 84% (0.250 g, 0.84 mmol) yield; m.p. 287–289 °C (Lit 288–290 °C [53]). ^1^H NMR (400 MHz, DMSO-*d*_6_, 298 K): δ = 8.49 (d, *J =* 8.9 Hz, 2H), 8.16 (d, *J =* 8.9 Hz, 2H), 8.13–8.06 (m, 1H), 7.80–7.07 (m, 3H); ^13^C{^1^H} NMR (100 MHz, DMSO-*d*_6_, 298 K): δ = 156.6, 148.6, 147.3, 133.1, 131.8, 130.6, 129.7, 126.9, 126.6, 125.8, 124.3, 114.4. HRMS (ESI) *m*/*z* calculated for [M + H]^+^ = [C_14_H_9_N_4_O_2_S]^+^ 297.0441; observed, 297.0440.

**3-(2-Nitrophenyl)benzo[4,5]thiazolo[2,3-*c*][1,2,4]triazole (4am).** The title compound was prepared according to general procedure 2 on a 0.130 g (0.50 mmol) scale. Purification by column chromatography (silica gel, 1. dichloromethane, 2. dichloromethane/acetone 19:1, R_f_ = 0.06) gave the product as a yellow powder in 98% (0.086 g, 0.291 mmol) yield; m.p. 217–218 °C. ^1^H NMR (400 MHz, DMSO-*d*_6_, 298 K): δ = 8.43 (dd, *J =* 8.3, 1.5 Hz, 1H), 8.12 (dd, *J =* 8.4, 1.5 Hz, 1H), 8.09–7.98 (m, 3H), 7.52–7.37 (m, 2H), 6.99 (dd, *J =* 8.1, 1.8 Hz, 1H); ^13^C{^1^H} NMR (100 MHz, DMSO-*d*_6_, 298 K): δ = 155.7, 148.1, 144.3, 134.8, 133.1, 132.9, 131.6, 129.2, 127.2, 126.6, 125.9, 125.6, 121.3, 113.3. HRMS (ESI) *m*/*z* calculated for [M + H]^+^ = [C_14_H_9_N_4_O_2_S]^+^ 297.0441; observed, 297.0440.

**3-(4-Fluorophenyl)benzo[4,5]thiazolo[2,3-*c*][1,2,4]triazole (4an).** The title compound was prepared according to general procedure 2 on a 0.196 g (0.50 mmol) scale. Purification by column chromatography (silica gel, 1. dichloromethane, 2. dichloromethane/acetone 19:1, R_f_ = 0.09) gave the product as an orange powder in 98% (0.131 g, 0.48 mmol) yield; m.p. 175–176 °C (Lit. 168–169 °C [37]). ^1^H NMR (400 MHz, DMSO-*d*_6_, 298 K): δ = 8.10–8.05 (m, 1H), 7.95–7.87 (m, 2H), 7.54–7.47 (m, 3H), 7.47–7.42 (m, 1H), 7.40–7.34 (m, 1H); ^13^C{^1^H} NMR (100 MHz, DMSO-*d*_6_, 298 K): δ = 163.4 (d, *J =* 247 Hz), 147.9, 131.8, 131.7, 129.7, 126.8, 126.3, 125.7, 123.3 (d, *J =* 3 Hz), 116.3 (d, *J =* 22 Hz), 113.9. ^19^F{^1^H} NMR (376 MHz, DMSO-*d*_6_, 298 K, referenced to C_6_H_5_F): δ = −109.80. HRMS (ESI) *m*/*z* calculated for [M + H]^+^ = [C_14_H_9_FN_3_S]^+^ 270.0496; observed, 270.0495.

**3-(3-Bromophenyl)benzo[4,5]thiazolo[2,3-*c*][1,2,4]triazole (4ao).** The title compound was prepared according to general procedure 2 on a 0.226 g (0.50 mmol) scale. Purification by column chromatography (silica gel, 1. dichloromethane, 2. dichloromethane/acetone 19:1, R_f_ = 0.13) gave the product as an orange powder in 82% (0.1350 g, 0.408 mmol) yield; m.p. 170–171 °C. ^1^H NMR (400 MHz, DMSO-*d*_6_, 298 K); δ = 8.13–8.07 (m, 1H), 8.06 (t, *J =* 1.8 Hz, 1H), 7.88 (ddd, *J =* 7.8, 2.9, 1.8 Hz, 2H), 7.63 (t, *J =* 7.9 Hz, 1H), 7.52–7.44 (m, 2H), 7.42–7.36 (m, 1H); ^13^C{^1^H} NMR (100 MHz, DMSO-*d*_6_, 298 K): δ = 155.9, 147.4, 133.5, 131.8, 131.7, 131.2, 129.7, 129.1, 128.2, 126.8, 126.4, 125.7, 122.1, 113.9. HRMS (ESI) *m*/*z* calculated for [M + H]^+^ = [C_14_H_9_BrN_3_S]^+^ 329.9695; observed, 329.9696.

**3-(3-Chlorophenyl)benzo[4,5]thiazolo[2,3-*c*][1,2,4]triazole. (4ap).** The title compound was prepared according to general procedure 2 on a 0.204 g (0.50 mmol) scale. Purification by column chromatography (silica gel, 1. dichloromethane, 2. dichloromethane/acetone 19:1, R_f_ = 0.19.) gave the product as an orange powder in 80% (0.104 g, 0.4 mmol) yield; m.p. 157–159 °C (Lit. 168–169 °C [38]) °C. ^1^H NMR (400 MHz, DMSO-*d*_6_, 298 K): δ = 8.12–8.06 (m, 1H), 7.93 (t, *J =* 1.8 Hz, 1H), 7.83 (dt, *J =* 7.4, 1.5 Hz, 1H), 7.75 (ddd, *J =* 8.2, 2.2, 1.3 Hz, 1H), 7.69 (t, *J =* 7.8 Hz, 1H), 7.52–7.44 (m, 2H), 7.42–7.36 (m, 1H); ^13^C{^1^H} NMR (100 MHz, DMSO-*d*_6_, 298 K): δ = 156.4, 147.9, 134.2, 132.2, 131.5, 131.1, 130.1, 129.4, 129.3, 128.3, 127.3, 126.9, 126.2, 114.9. HRMS (ESI) *m*/*z* calculated for [M + H]^+^ = [C_14_H_9_ClN_3_S]^+^ 286.0200; observed, 286.0202.

**3-(2-Fluorophenyl)benzo[4,5]thiazolo[2,3-*c*][1,2,4]triazole (4aq).** The title compound was prepared according to general procedure 2 on a 0.196 g (0.50 mmol) scale. Purification by column chromatography (silica gel, 1. dichloromethane, 2. dichloromethane/acetone 19:1, R_f_ = 0.10) gave the product as an orange powder in 86% (0.115 g, 0.433mmol) yield; m.p. 133–134 °C. ^1^H NMR (400 MHz, DMSO-*d*_6_, 298 K): δ = 8.13–8.09 (m, 1H), 7.86–7.75 (m, 2H), 7.58 (ddd, *J =* 10.0, 8.4, 1.1 Hz, 1H), 7.54–7.44 (m, 3H), 7.18 (dt, *J =* 6.8, 2.2 Hz, 1H); ^13^C{^1^H} NMR (100 MHz, DMSO-*d*_6_, 298 K): δ = 159.6 (d, *J =* 247 Hz), 156.02, 143.07, 133.7 (d, *J =* 8 Hz), 132.2 (d, *J =* 2 Hz), 131.7, 129.3, 127.0, 126.6, 125.7, 125.5 (d, *J =* 3 Hz), 116.5 (d, *J =* 20 Hz), 114.8 (d, *J =* 15 Hz), 113.7 (d, *J =* 2 Hz). ^19^F{^1^H} NMR (376 MHz, DMSO-*d*_6_, 298 K, referenced to C6H5F): δ = −112.90. HRMS (ESI) *m*/*z* calculated for [M + H]^+^ = [C_14_H_9_FN S]^+^ 270.0496; observed, 270.0496.

**3-(Pyridin-2-yl)benzo[4,5]thiazolo[2,3-*c*][1,2,4]triazole (4ar).** The title compound was prepared according to general procedure 2 on a 0.374 g (1.00 mmol) scale. Purification by column chromatography (silica gel, 1. dichloromethane, 2. dichloromethane/acetone 1:1, R_f_ = 0.20) gave the product as a brown powder in 85% (0.213 g, 0.84 mmol) yield; m.p. 238–239 °C. ^1^H NMR (400 MHz, DMSO-*d*_6_, 298 K): δ = 9.03 (s, 1H), 8.86 (d, *J =* 4.2 Hz, 1H), 8.37–8.27 (m, 1H), 8.14–8.06 (m, 1H), 7.71 (dd, *J =* 7.9, 4.0 Hz, 1H), 7.54–7.42 (m, 2H), 7.41–7.33 (m, 1H); ^13^C{^1^H} NMR (100 MHz, DMSO-*d*_6_, 298 K): δ = 156.2, 151.5, 149.4, 146.4, 136.9, 131.7, 129.6, 126.9, 126.5, 125.8, 124.0, 123.4, 114.0. HRMS (ESI) *m*/*z* calculated for [M + H]^+^ = [C_13_H_9_N_4_S]^+^ 253.0542; observed, 253.0545.

**3-(3-Chlorophenyl)-6-(trifluoromethyl)benzo[4,5]thiazolo[2,3-*c*][1,2,4]triazole (4bp).** The title compound was prepared according to general procedure 2 on a 0.476 g (1.00 mmol) scale. Purification by column chromatography (silica gel, 1. dichloromethane, 2. dichloromethane/acetone 19:1, R_f_ = 0.17) gave the product as an off-white powder in 93% (0.328 g, 0.93 mmol) yield; m.p. 203–204 °C. ^1^H NMR (400 MHz, DMSO-*d*_6_, 298 K): δ = 8.38 (d, *J =* 8.6 Hz, 1H), 8.00 (s, 1H), 7.91–7.85 (m, 2H), 7.81–7.67 (m, 2H), 7.60 (s, 1H); ^13^C{^1^H} NMR (100 MHz, DMSO-*d*_6_, 298 K): δ = 156.4, 147.7, 137.0, 133.7, 131.1, 130.9, 129.9, 128.9, 128.6, 127.8, 127.0, 126.7 (q, *J* = 32.6 Hz), 123.6 (q, *J* = 272.5 Hz), 122.9 (q, *J* = 3.7 Hz), 110.5 (q, *J* = 4.6 Hz). ^19^F{^1^H} NMR (376 MHz, DMSO-*d*_6_, 298 K, referenced to C_6_H_5_F): δ = −61.08 HRMS (ESI) *m*/*z* calculated for [M + H]^+^ = [C_15_H_8_ClF_3_N_3_S]^+^ 354.0074; observed, 354.0074.

**3-(4-Fluorophenyl)-6-methoxybenzo[4,5]thiazolo[2,3-*c*][1,2,4]triazole (4cn).** The title compound was prepared according to general procedure 2 on a 0.421 g (1.00 mmol) scale. Purification by column chromatography (silica gel, 1. dichloromethane, 2. dichloromethane/acetone 9:1, R_f_ = 0.21) gave the product as an orange power in 89% (0.267 g, 0.89 mmol) yield; m.p. 204–205 °C. ^1^H NMR (400 MHz, DMSO-*d*_6_, 298 K): δ = 7.98 (d, *J =* 8.9 Hz, 1H), 7.92 (dd, *J =* 8.8, 5.4 Hz, 1H), 7.52 (t, *J =* 8.9 Hz, 2H), 7.13 (dd, *J =* 9.0, 2.5 Hz, 1H), 6.85 (d, *J =* 2.6 Hz, 1H), 3.71 (s, 3H); ^13^C{^1^H} NMR (100 MHz, DMSO-*d*_6_, 298 K): δ = 163.9 (d, *J* = 248.7 Hz), 158.6, 132.3 (d, *J* = 8.8 Hz), 130.8, 126.8, 123.7 (d, *J* = 3.3 Hz), 123.0, 116.7 (d, *J* = 22.0 Hz),113.6, 100.6, 56.1. ^19^F{^1^H} NMR (376 MHz, DMSO-*d*_6_, 298 K, referenced to C_6_H_5_F): δ = −111.07. HRMS (ESI) *m*/*z* calculated for [M + H]^+^ = [C_15_H_10_OFN_3_S]^+^ 300.0601; observed, 300.0603.

**6-Bromo-3-(4-methoxyphenyl)benzo[4,5]thiazolo[2,3-*c*][1,2,4]triazole (4di).** The title compound was prepared according to general procedure 2 on a 0.241 g (0.50 mmol) scale. Purification by column chromatography (silica gel, 1. dichloromethane, 2. dichloromethane/acetone 9:1, R_f_ = 0.18) gave the product as a brown powder in 84% (0.152 g, 0.42 mmol) yield; m.p. 219–220 °C. ^1^H NMR (400 MHz, DMSO-*d*_6_, 298 K): δ = 8.06 (d, *J =* 8.6 Hz, 1H), 7.86–7.72 (m, 2H), 7.68 (dd, *J =* 8.7, 2.0 Hz, 1H), 7.46 (d, *J =* 2.0 Hz, 1H), 7.31–7.13 (m, 2H), 3.89 (s, 3H); ^13^C{^1^H} NMR (100 MHz, DMSO-*d*_6_, 298 K): δ = 161.6, 158.3, 145.4, 131.9, 131.3, 131.2, 129.7, 129.4, 127.9, 127.7, 119.2, 119.0, 117.0, 115.0, 56.0. HRMS (ESI) *m*/*z* calculated for [M + H]^+^ = [C_15_H_10_BrN_3_OS]^+^ 359.9801; observed, 359.9800.

**6-Bromo-3-(pyridin-3-yl)benzo[4,5]thiazolo[2,3-*c*][1,2,4]triazole (4dr).** The title compound was prepared according to general procedure 2 on a 0.530 g (1.00 mmol) scale. Purification by column chromatography (silica gel, 1. dichloromethane, 2. dichloromethane/acetone 9:1, R_f_ = 0.20) gave the product as an off-white powder in 69% (0.280 g, 1.68 mmol) yield; m.p. 223–224 °C. ^1^H NMR (400 MHz, DMSO-*d*_6_, 298 K): δ = 9.03 (s, 1H), 8.87 (d, *J =* 5.0 Hz, 1H), 8.32 (d, *J =* 7.9 Hz, 1H), 8.09 (d, *J =* 8.6 Hz, 1H), 7.82–7.62 (m, 2H), 7.39 (s, 1H); ^13^C{^1^H} NMR (100 MHz, DMSO-*d*_6_, 298 K): δ = 157.1, 152.2, 149.9, 146.9, 137.3, 131.8, 131.2, 129.6, 128.0, 124.5, 123.7, 119.3, 117.2. HRMS (ESI) *m*/*z* calculated for [M + H]^+^ = [C_15_H_11_BrN_3_O_2_S_2_]^+^ 407.947; observed, 407.9476.

**7-Bromo-3-phenylbenzo[4,5]thiazolo[2,3-*c*][1,2,4]triazole (4ee).** The title compound was prepared according to general procedure 2 on a 0.452 g (1.0 mmol) scale. Purification by column chromatography (silica gel, 1. dichloromethane, 2. dichloromethane/acetone 9:1, R_f_ = 0.17) gave the product as an orange powder in 94% (0.309 g, 0.94 mmol) yield; m.p. 229–230 °C (Lit. 224–225 °C [38]). ^1^H NMR (400 MHz, DMSO-*d*_6_, 298 K): δ =8.39 (d, *J =* 2.1 Hz, 1H), 7.88–7.78 (m, 2H), 7.71–7.59 (m, 4H), 7.32 (d, *J =* 8.8 Hz, 1H); ^13^C{^1^H} NMR (100 MHz, DMSO-*d*_6_, 298 K): δ = 155.7, 148.8, 134.1, 130.8, 129.6, 129.1, 129.1, 128.0, 126.5, 118.1, 115.4. HRMS (ESI) *m*/*z* calculated for [M + H]^+^ = [C_14_H_9_BrN_3_S]^+^ 329.9695; observed, 329.9695.

**8-Chloro-3-(3-nitrophenyl)benzo[4,5]thiazolo[2,3-*c*][1,2,4]triazole (4fl).** The title compound was prepared according to general procedure 2 on a 0.22 g (0.50 mmol) scale. Purification by column chromatography (silica gel, 1. dichloromethane, 2. dichloromethane/acetone 9:1) was attempted but no product could be isolated.

**3-(2-Fluorophenyl)benzo[4,5]thiazolo[2,3-*c*][1,2,4]triazol-6-amine (4gq).** The title compound was prepared according to general procedure 2 on a 0.203 g (0.50 mmol) scale. The crude material was analyzed by LCMS according to which no product was formed.

**8-Fluoro-3-(p-tolyl)benzo[4,5]thiazolo[2,3-*c*][1,2,4]triazole (4if).** The title compound was prepared according to general procedure 2 on a 0.203 g (0.50 mmol) scale. Purification by column chromatography (silica gel, 1. dichloromethane, 2. dichloromethane/acetone 19:1) gave trace amounts of the product as confirmed by LCMS.

**Ethyl 3-cyclopropylbenzo[4,5]thiazolo[2,3-*c*][1,2,4]triazole-6-carboxylate (4jc).** The title compound was prepared according to general procedure 2 on a 0.205 g (0.50 mmol) scale. The reaction mixture was separated by column chromatography (silica gel, 1. dichloromethane, 2. A gradient of 5–50% acetone in dichloromethane) but no product was isolated.

**3-(3-Bromophenyl)-6-chlorobenzo[4,5]thiazolo[2,3-*c*][1,2,4]triazole (4ko).** The title compound was prepared according to general procedure 2 on a 0.487 g (1.00 mmol) scale. Purification by column chromatography (silica gel, 1. dichloromethane, 2. dichloromethane/acetone 19:1, R_f_ = 0.10) gave the product as an off-white powder in 78% (0285 g, 0.78 mmol) yield; m.p. 238–239 °C. ^1^H NMR (400 MHz, DMSO-*d*_6_, 298 K): δ = 8.14 (d, *J =* 8.7 Hz, 1H), 8.08 (s, 1H), 7.90 (dd, *J =* 7.9, 2.2 Hz, 2H), 7.65 (t, *J =* 7.9 Hz, 1H), 7.58 (dd, *J =* 8.7, 2.1 Hz, 1H), 7.31 (d, *J =* 2.2 Hz, 1H); ^13^C{^1^H} NMR (100 MHz, DMSO-*d*_6_, 298 K): δ = 156.6, 147.5, 133.7, 131.8, 131.3, 130.9, 130.8, 130.5, 128.8, 128.2, 127.2, 126.3, 122.1, 114.0. HRMS (ESI) *m*/*z* calculated for [M + H]^+^ = [C_14_H_8_BrClN_3_S]^+^ 363.9305; observed, 363.9312.

**3-(3-Bromophenyl)-6-(methylsulfonyl)benzo[4,5]thiazolo[2,3-*c*][1,2,4]triazole (4mo).** The title compound was prepared according to general procedure 2 on a 0.530 g (1.00 mmol) scale. Purification by column chromatography (silica gel, 1. dichloromethane, 2. dichloromethane/acetone 9:1, R_f_ = 0.15) gave the product as an orange powder in 69% (0.280 g, 1.68 mmol) yield; m.p. 223–224 °C. ^1^H NMR (400 MHz, DMSO-*d*_6_, 298 K): δ = 8.41 (d, *J =* 8.6 Hz, 1H), 8.13 (t, *J =* 1.8 Hz, 1H), 8.05 (dd, *J =* 8.4, 1.7 Hz, 1H), 7.96–7.88 (m, 3H), 7.64 (t, *J =* 7.9 Hz, 1H), 3.23 (s, 3H); ^13^C{^1^H} NMR (100 MHz, DMSO-*d*_6_, 298 K): δ = 139.3, 138.5, 134.3, 132.1, 131.7, 130.2, 129.2, 128.5, 127.3, 126.0, 125.2, 122.7, 115.2, 113.1, 43.8. HRMS (ESI) *m*/*z* calculated for [M + H]^+^ = [C_15_H_11_BrN_3_O_2_S_2_]^+^ 407.9471; observed, 407.9476.

**3-(Pyridin-3-yl)-6-(trifluoromethoxy)benzo[4,5]thiazolo[2,3-*c*][1,2,4]triazole (4or).** The title compound was prepared according to general procedure 2 on a 0.229 g (0.50 mmol) scale. Purification by column chromatography (silica gel, 1. dichloromethane, 2. dichloromethane/acetone 1:1) yielded trace amount of product in crude form.

**3-(Pyridin-3-yl)benzo[4,5]thiazolo[2,3-c][1,2,4]triazole-6-sulfonamide (4pr).** The title compound was prepared according to general procedure 2 on a 0.227 g (0.50 mmol) scale. Purification by column chromatography (silica gel, 1. dichloromethane, 2. dichloromethane/methanol 9:1) gave no product.

#### 4.3.3. General Procedure 3 for the Preparation and Isolation of the Disulfide Intermediates

***Deprotection:*** A 15 mL round bottomed flask fitted with a stir bar was loaded with the appropriate triazole **2** (2.00 mmol, 1 equiv.), dry dichloromethane (3–4 mL) and anisole (10 mmol, 5 equiv.), and set under an argon atmosphere. After cooling to 0 °C in an ice bath, trifluormethanesulfonic acid (10 mmol, 5 equiv.) was added dropwise to the reaction mixture, which was kept in the ice bath for 4 h or until the reaction was shown to be complete by LCMS analysis. Upon completion of the deprotection, the solvent was removed under vacuum at ambient temperature and the reaction mixture containing the free thiol **3** was used without further purification for the disulfide formation. ***Disulfide formation:*** DMSO (3 mL) was added to the crude reaction mixture of thiol 3 and the reaction was stirred at room temperature until the oxidative disulfide coupling was complete as determined by samples taken and analyzed by LCMS (typically less than 1 h). After addition of a saturated aqueous solution of NaHCO_3_, the aqueous phase was extracted three times with dichloromethane. The combined organic phases were dried over MgSO_4_, filtered, and the solvent removed under vacuum at ambient temperature. The resulting crude disulfide **5** was purified by column chromatography as described below.

**1,2-Bis(2-(3-phenyl-4*H*-1,2,4-triazol-4-yl)phenyl)disulfane (5ae).** The title compound was prepared according to general procedure 3 from **2ae** on a 0.747 g (2.00 mmol) scale. Purification by column chromatography (silica gel, 1. dichloromethane, 2. dichloromethane/methanol 9:1, R_f_ = 0.58) gave the product as a pale-yellow powder in 86% (0.436 g, 0.864 mmol) yield; m.p. 181–182 °C. ^1^H NMR (400 MHz, DMSO-*d*_6_, 298 K): δ = 8.76 (s, 2H), 7.68–7.60 (m, 2H), 7.54–6.90 (m, 16H); ^13^C{^1^H} NMR (100 MHz, DMSO-*d*_6_, 298 K): δ = 152.5, 145.6, 131.3, 130.0, 129.2, 128.8, 127.7, 126.3 (due to the low solubility and the broadness of some carbon signals, quaternary carbons could not be assigned with confidence and have been omitted from the listed peaks). HRMS (ESI) *m*/*z* calculated for [M + H]^+^ = [C_28_H_20_N_6_S_2_]^+^ 505.1264; observed, 505.1272.

**1,2-Bis(2-(3-(4-methoxyphenyl)-4*H*-1,2,4-triazol-4-yl)phenyl)disulfane (5ai).** The title compound was prepared according to general procedure 3 from **2ai** on a 0.807 g (2.00 mmol) scale. Purification by column chromatography (silica gel, 1. dichloromethane, 2. dichloromethane/methanol 9:1, R_f_ = 0.59) gave the product as a pale-yellow powder in 90% (0.507 g, 0.898 mmol) yield; m.p. 194–195 °C. ^1^H NMR (400 MHz, DMSO-*d*_6_, 298 K): δ = 8.70 (s, 2H), 7.67–5.57 (m, 2H), 7.54–6.83 (m, 14H), 3.76 (s, 6H); the compound was not sufficiently soluble in DMSO-*d*_6_ to obtain a ^13^C{^1^H} NMR spectrum. HRMS (ESI) *m*/*z* calculated for [M + H]^+^ = [C_30_H_24_N_6_O_2_S_2_]^+^ 565.1475; observed, 565.1489.

**1,2-Bis(2-(3-(4-nitrophenyl)-4*H*-1,2,4-triazol-4-yl)phenyl)disulfane (5al).** The title compound was prepared according to general procedure 3 from **2al** on a 0.836 g (2.00 mmol) scale. Purification by column chromatography (silica gel, 1. dichloromethane, 2. dichloromethane/acetone 1:1, R_f_ = 0.5) gave the product as a white powder in 85% (0.508 g, 0.854 mmol) yield, m.p. 217–218 °C. ^1^H NMR (400 MHz, DMSO-*d*_6_, 298 K): δ = 8.86 (s, 2H), 8.24–8.12 (m, 4H), 7.71 (d, J = 7.8 Hz, 2H), 7.61–7.28 (m, 10H); ^13^C{^1^H} NMR (100 MHz, DMSO-*d*_6_, 298 K): δ = 151.0, 148.0, 146.5, 132.9, 132.3, 131.5, 130.1, 129.2, 128.8, 124.0 (one carbon signal could not be found; most likely due to its broadness). HRMS (ESI) *m*/*z* calculated for [M + H]^+^ = [C_28_H_18_N_8_O_4_S_2_]^+^ 595.0965; observed, 595.0977.

### 4.4. Antifungal Testing

Two methods were used for antifungal testing: disk diffusion assays and MICs [54]. For disk diffusion assays, fungal strains were grown in YPD, diluted 1:1 into YPD and a sterile cotton-tipped applicator was used to apply the fungal cultures to the surface of a YPD plate. After the culture had dried, sterile disks containing 10 µg of the antifungal candidates were applied to the plate. Following 24 h incubation at 30 °C, each disk was examined for the production of a zone of clearing around the disk. MIC assays were conducted according to CLSI guidelines (CLSI, 2017 #3098). Briefly, each antifungal candidate was resuspended in DMSO and then diluted in RPMI-MOPS and evaluated in a two-fold dilution range (256 µg/mL to 8 µg/mL). Fluconazole (Spectrum Chemical, New Brunswick, NJ, USA), miconazole (Thermo Fisher Scientific, Waltham, MA, USA), voriconazole (Thermo Fisher Scientific), amphotericin B (Gold Bio, St. Louis, MO, USA), and caspofungin acetate (APExBIO, Houston, TX, USA) were included as controls for antifungal testing. All dilutions were made in RPMI-MOPS in a 96-well microtiter plate. *C. albicans* or *T. asahii* were grown overnight, rinsed in phosphate-buffered saline and added to each well at an initial inoculum of a 0.5 × 10^3^/mL. The plates were incubated for 48 h at 30 °C in a humidified incubator. Positive growth controls included the fungal strains grown in media alone and negative growth controls were media only. Plates were inspected for turbidity. MICs were defined as the concentration that inhibited fungal growth.

## 5. Conclusions

In summary, we have prepared C3,N4-substituted triazole derivatives **2aa**–**2ar** and **2bp**–**2pr** containing a N4-(2-((4-methoxybenzyl)thio)phenyl) group. Selective removal of the 4-methoxybenzyl substituent from the sulfur atom followed by oxidation with DMSO at 100 °C yielded the C5-substituted tricyclic benzo[4,5]thiazolo[2,3-*c*][1,2,4]triazoles **4bp**–**4pr**. Mechanistic investigations suggest that this oxidative cyclization reaction proceeds via an ionic pathway involving a disulfide intermediate which, in the presence of a strong acid, cyclizes to the corresponding tricyclic heteroarene.

Antifungal testing of both the novel sulfur-containing triazoles and the benzo[4,5]thiazolo[2,3-*c*][1,2,4]triazoles revealed that many of these compounds showed some activity against *C. albicans*. Almost all our molecules showed some activity against the emerging fungal pathogen *T. asahii*; however, they were significantly less active than the antifungal drugs fluconazole, miconazole, voriconazole, amphotericin B, and caspofungin acetate.

## Figures and Tables

**Table 1:** Antifungal activity of triazoles and benzo[4,5]thiazolo[2,3-*c*][1,2,4]triazoles.

Compound	*C. albicans* (SC5314) Plate Inhibition *^a^*	*C. albicans* (SC5314) MIC (μg/mL) *^b^*	*T. asahii* (OK01) Plate Inhibition *^a^*	*T. asahii* (OK01) MIC (μg/mL) *^b^*
**2ab**	No	-	Yes	256
**2ac**	No	-	Yes	256
**2ad**	Yes	256	Yes	256
**2ae**	No	-	Yes	256
**2af**	Yes	256	Yes	>256
**2ag**	No	-	Yes	256
**2ah**	Yes	256	Yes	256
**2ai**	No	-	Yes	256
**2aj**	No	-	Yes	256
**2ak**	No	-	Yes	256
**2al**	Yes	256	Yes	256
**2am**	No	-	Yes	256
**2an**	Yes	256	Yes	256
**2ao**	No	-	Yes	256
**2ap**	No	-	Yes	256
**2aq**	No	-	Yes	256
**2ar**	Yes	256	No	-
**2bp**	No	-	Yes	>256
**2cd**	No	-	Yes	256
**2cn**	No	-	Yes	256
**2di**	No	-	Yes	>256
**2dr**	No	-	Yes	>256
**2ee**	No	-	Yes	256
**2fl**	No	-	Yes	>256
**2gq**	No	-	Yes	256
**2if**	Yes	>256	Yes	256
**2jc**	No	-	Yes	256
**2ko**	No	-	Yes	>256
**2lk**	No	-	Yes	>256
**2mo**	Yes	>256	Yes	>256
**2or**	Yes	256	Yes	256
**2pr**	No	-	Yes	256
**4ab**	No	-	No	-
**4ad**	No	-	No	-
**4ae**	No	-	Yes	256
**4af**	Yes	256	Yes	256
**4ag**	Yes	256	Yes	256
**4ah**	Yes	256	Yes	256
**4ai**	No	-	Yes	256
**4aj**	No	-	Yes	256
**4al**	No	-	Yes	>256
**4am**	Yes	256	Yes	256
**4an**	Yes	256	Yes	256
**4ao**	Yes	256	Yes	>256
**4ap**	No	-	Yes	256
**4aq**	Yes	256	Yes	256
**4ar**	No	-	Yes	256
**4bp**	Yes	>256	Yes	>256
**4cn**	Yes	256	Yes	256
**4di**	Yes	>256	Yes	>256
**4dr**	No	-	Yes	256
**4ee**	No	-	Yes	>256
**4ko**	Yes	>256	Yes	>256
**4mo**	Yes	>256	Yes	>256
Fluconazole	Yes	3.125	Yes	50–100
Miconazole	Yes	3.125	Yes	1.56
Voriconazole	Yes	1.56	Yes	1.56
Amphotericin B	Yes	0.24	Yes	1.56
Capsofungin acetate	Yes	0.19	Yes	25

*^a^* Disk assays after 24 h of incubation at 30 °C. *^b^* MIC assays after 48 h of incubation at 30 °C in a humidified incubator.

## Data Availability

The experimental procedures to reproduce this work and all characterization data for the compounds are included in the article itself or in the Supplementary Materials.

## Supplementary Materials

The following supporting information can be downloaded at: https://www.mdpi.com/article/10.3390/ph18020249/s1, Supporting Information containing the synthesis and characterization of anilines **1a**–**1p**; processed ^1^H, ^13^C, and ^19^F NMR spectra of all new compounds reported in this article.
