# Supplementary material for: Access to Substituted Tricyclic Heteroarenes by an Oxidative Cyclization Reaction and Their Antifungal Performance
Source: Pharmaceuticals (Basel). 2025 Feb 12;18(2):249. doi: 10.3390/ph18020249 (PMC11860112; doi:10.3390/ph18020249)
Supplement: Supplementary file 1 [file pharmaceuticals-18-00249-s001.zip › pharmaceuticals-3334395-supplementary.pdf]

Supporting Information

Access to Substituted Tricyclic Heteroarenes by an Oxidative Cyclization Reaction and Their Antifungal Performance

Rehema Nakiwala,[1] Noopur Dasgupta,[2] Rebecca Wilson,[2] Erika I. Lutter,[2] and Jeanne L. Bolliger[1,*]

[1]Department of Chemistry, 107 Physical Sciences, Oklahoma State University, Stillwater, OK, 74078, United States.

[2]Department of Microbiology and Molecular Genetics, 307 Life Sciences East, Oklahoma State University, Stillwater, OK, 74078, United States.

Contents

1. Synthesis of Anilines 1a-p *S3*
2. References *S6*
3. NMR Spectra *S7*

1. Synthesis of Anilines 1a-r

Anilines **1a-p** have been synthesized in two steps as shown in Scheme S1 and in the general procedure below. Anilines **1a-k** have been previously reported by our group.[1]

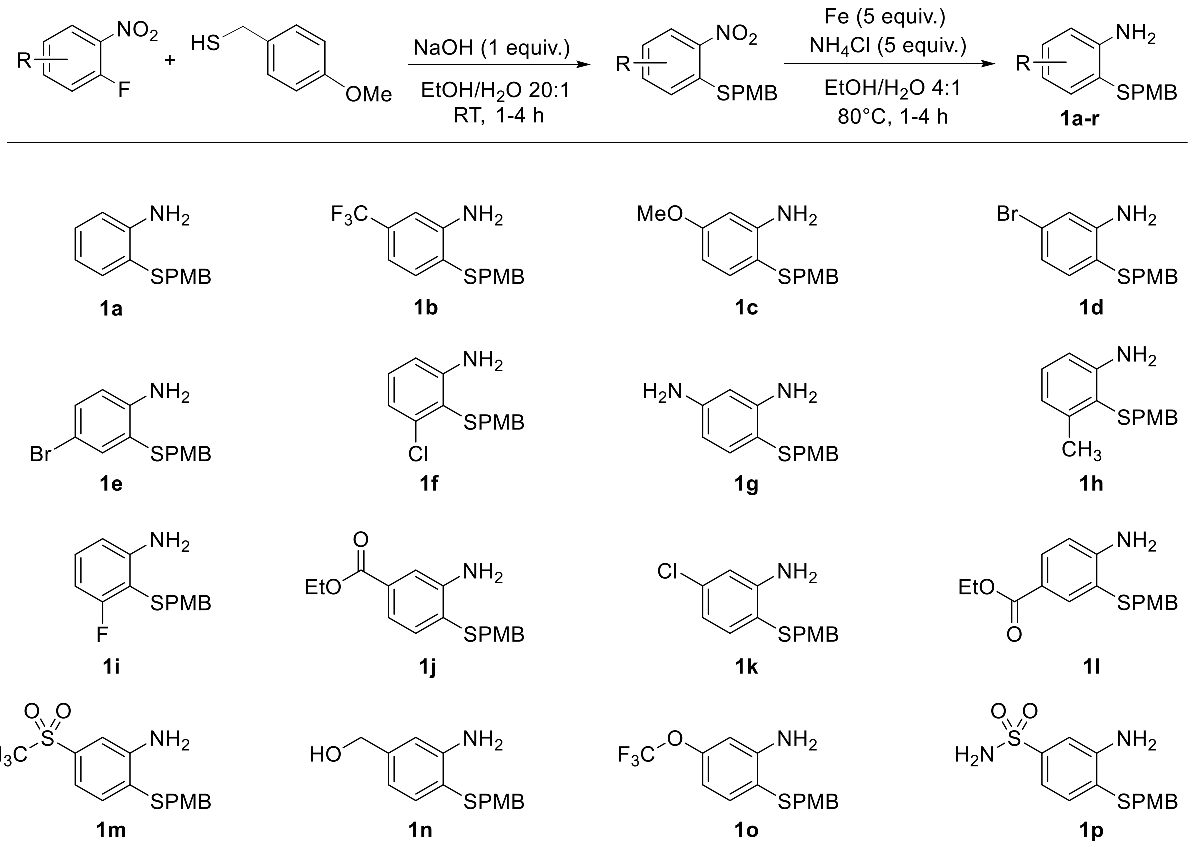

Scheme S1. Synthesis of Anilines 1a-p.

General Procedure for the Synthesis of 4-Methoxybenzyl)(2-nitrophenyl)sulfanes and Anilines 1a-p

4-Methoxybenzyl)(2-nitrophenyl)sulfanes: The following description is for a 30 mmol scale reaction. The solvent quantities and flask sizes were adjusted accordingly for smaller-scale reactions.

A 500 mL round-bottomed flask equipped with a stir bar was loaded with the 1-fluoro-2-nitrobenzene derivative (1 equiv) and 200 mL of ethanol and placed under an atmosphere of argon. (4-methoxyphenyl)methanethiol (1 equiv) was added with a syringe, followed by a dropwise addition of NaOH (1 equiv) dissolved in 10 mL of H_2O. The reaction mixture was stirred at room temperature until TLC indicated the completion of the reaction (typically within 2 h). If the product precipitated from the reaction mixture, it was filtered off and washed with H_2O, followed by ethanol and diethyl ether which generally gave the product in pure form. If the title compound did not precipitate from the reaction mixture, the solvent was removed under reduced pressure, the residue was diluted with 150 mL of H_2O and extracted twice with dichloromethane. The organic phases were combined, dried over $MgSO_4$, filtered, and concentrated. The resulting crude product was purified by recrystallization or column chromatography as described below.

Anilines (**1l-p**)*:* The following description is for a 20–25 mmol scale reaction. The solvent quantities and flask sizes were adjusted accordingly for smaller-scale reactions.

A 250 mL round-bottomed flask equipped with a stir bar was loaded with the (4-methoxybenzyl)(2-nitrophenyl)sulfane derivative (1 equiv), N$_{H}$4Cl (5.0 equiv), and 150 mL of EtOH/H_2O (4:1). The reaction flask was placed into an oil bath set to 80 ◦C and the iron power (5.0 equiv) was added while stirring. Then the reaction flask was fitted with a reflux condenser and the reaction was stirred under argon at 80 ◦C until TLC indicated a complete reduction (typically between 1 and 4 h). After cooling to room temperature, the reaction mixture was filtered through celite and concentrated under reduced pressure. The residue was then basified with 1M NaOH and extracted twice with dichloromethane, dried over $MgSO_4$, and evaporated. The crystalline solid was washed with hexanes or diethyl ether as described below.

Anilines **1a-k** have been previously reported by our group.[1]

Ethyl 4-amino-3-((4-methoxybenzyl)thio)benzoate (1l). The title compound was prepared in two steps from ethyl 3-fluoro-4-nitrobenzoate and purified as described below.

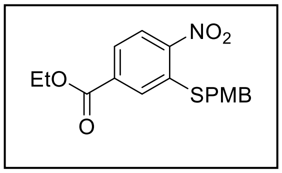

Ethyl 3-((4-methoxybenzyl)thio)-4-nitrobenzoate. The title compound has been prepared according to the general procedure on a 5.013 g (23.5 mmol) scale. Once the reaction was determined to be complete by TLC, the solid was filtered off, washed with H_2O, followed by ethanol and finally diethyl ether to yield the product as a yellow powder in 97% yield (7.943 g, 22.9 mmol), m.p. 86-87 °C.

^{1}H NMR (400 MHz, DMSO-d_6, 298 K): δ = 8.28 (d, J = 8.6 Hz, 1H), 8.16 (d, J = 1.5 Hz, 1H), 7.85 (dd, J = 8.5, 1.6 Hz, 1H), 7.38 (d, J = 8.7 Hz, 2H), 6.90 (d, J = 8.7 Hz, 2H), 4.42 – 4.32 (m, 4H), 3.73 (s, 3H), 1.36 (t, J = 7.1 Hz, 3H); ^{13}C{^{1}H} NMR (100 MHz, DMSO-d_6, 298 K): δ = 164.1, 158.7, 147.7, 137.0, 134.1, 130.4, 128.2, 127.0, 126.3, 125.5, 114.0, 61.8, 55.1, 35.6, 14.0.

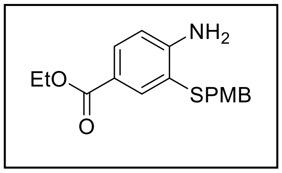

Ethyl 4-amino-3-((4-methoxybenzyl)thio)benzoate (1l). The title compound has been prepared according to the general procedure on a 7.857 g (22.62 mmol) scale. Purification by washing with hexanes followed by minimal amounts of diethyl ether yielded the product as a salmon pink powder in 96% yield (6.908 g, 21.77 mmol), m.p. 88-90 °C.

^{1}H NMR (400 MHz, DMSO-d_6, 298 K): δ = 7.74 – 7.65 (m, 1H), 7.62 (d, J = 8.5 Hz, 1H), 7.13 (d, J = 8.4 Hz, 2H), 6.81 (d, J = 8.4 Hz, 2H), 6.74 (d, J = 8.5 Hz, 1H), 6.16 (s, 2H), 4.18 (q, J = 7.0 Hz, 2H), 3.90 (s, 2H), 3.70 (s, 3H), 1.25 (t, J = 7.1 Hz, 3H); ^{13}C{^{1}H} NMR (100 MHz, DMSO-d_6, 298 K): δ = 165.3, 158.3, 153.6, 137.1, 130.9, 130.1, 129.6, 116.8, 114.9, 113.6, 113.2, 59.7, 55.0, 37.2, 14.3.

2-((4-Methoxybenzyl)thio)-5-(methylsulfonyl)aniline (1m). The title compound was prepared in two steps from 2-fluoro-5-(methylsulfonyl)aniline and purified as described below.

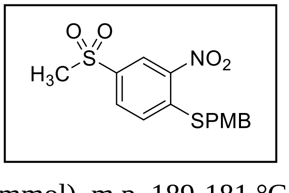

(4-Methoxybenzyl)(4-(methylsulfonyl)-2-nitrophenyl)sulfane. The title compound was prepared according to the general procedure on a 4.964 g (22.6 mmol) scale. Once the reaction was determined to be complete by TLC, the solid was filtered off, washed with H_2O, followed by ethanol and finally diethyl ether to yield the product as a yellow powder in 95% yield (7.574 g, 21.43 mmol), m.p. 189-181 °C.

^{1}H NMR (400 MHz, DMSO-d_6, 298 K): δ = 8.61 (d, J = 2.0 Hz, 1H), 8.16 (dd, J = 8.6, 2.0 Hz, 1H), 7.99 (d, J = 8.7 Hz, 1H), 7.40 (d, J = 8.6 Hz, 2H), 6.92 (d, J = 8.6 Hz, 2H), 4.42 (s, 2H), 3.74 (s, 3H), 3.33 (s,

3H); $^{13}C\{^{1}H\}$ NMR (100 MHz, DMSO-d_6, 298 K): δ = 158.8, 144.3, 143.8, 137.0, 131.4, 130.6, 128.6, 126.4, 124.9, 114.2, 55.1, 43.3, 35.7.

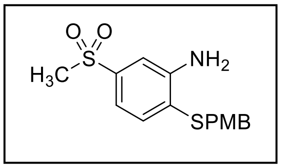

2-((4-Methoxybenzyl)thio)-5-(methylsulfonyl)aniline (1m). The title compound was prepared according to the general procedure on a 7.476 g (21.15 mmol) scale. After washing with hexanes and diethyl ether, the pure product was obtained in the form of a white powder in 83% yield (5.705 g, 17.64 mmol), m.p. 124-125 °C.

^{1}H NMR (400 MHz, DMSO-d_6, 298 K): δ = 7.34 (d, J = 8.1 Hz, 1H), 7.24 (d, J = 8.7 Hz, 2H), 7.18 (d, J = 2.0 Hz, 1H), 6.96 (dd, J = 8.1, 2.0 Hz, 1H), 6.88 – 6.81 (m, 2H), 5.70 (s, 2H), 4.10 (s, 2H), 3.72 (s, 3H), 3.10 (s, 3H); $^{13}C\{^{1}H\}$ NMR (100 MHz, DMSO-d_6, 298 K): δ = 158.4, 148.0, 139.7, 131.8, 130.1, 128.9, 123.5, 113.9, 113.8, 111.3, 55.0, 43.7, 35.9.

(3-Amino-4-((4-methoxybenzyl)thio)phenyl)methanol (1n) The title compound was prepared in two steps from (4-fluoro-3-nitrophenyl)methanol and purified as described below.

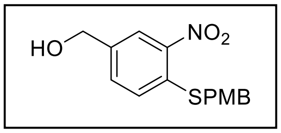

((4-((4-Methoxybenzyl)thio)-3-nitrophenyl)methanol. The title compound was prepared according to the general procedure on a 4.839 g (28.28 mmol) scale. Following extraction with dichloromethane, the crude product was purified by column chromatography (silica gel, 1. dichloromethane, 2. dichloromethane/methanol 19:1) which yielded the product as a yellow powder in 65% yield (5.618 g, 18.40 mmol), m.p. 127-128 °C.

^{1}H NMR (400 MHz, DMSO-d_6, 298 K): δ = 8.12 (s, 1H), 7.70 (d, J = 8.4 Hz, 1H), 7.63 (dd, J = 8.4, 1.5 Hz, 1H), 7.35 (d, J = 8.6 Hz, 2H), 6.89 (d, J = 8.6 Hz, 2H), 5.46 (t, J = 5.7 Hz, 1H), 4.55 (d, J = 5.7 Hz, 2H), 4.29 (s, 2H), 3.73 (s, 3H); $^{13}C\{^{1}H\}$ NMR (100 MHz, DMSO-d_6, 298 K): δ = 158.6, 145.2, 140.5, 134.5, 132.1, 130.4, 127.6, 127.3, 123.1, 114.0, 61.3, 55.1, 35.6.

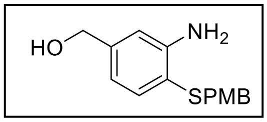

(3-amino-4-((4-methoxybenzyl)thio)phenyl)methanol (1n). The title compound was prepared according to the general procedure on a 4.658 g (15.25 mmol) scale. Upon washing with hexanes, the product was obtained quantitatively (4.203 g, 15.25 mmol) in the form of a brown powder, m.p. 82 °C.

^{1}H NMR (400 MHz, DMSO-d_6, 298 K): δ = 7.15 (d, J = 8.5 Hz, 2H), 7.05 (d, J = 7.8 Hz, 1H), 6.82 (d, J = 8.6 Hz, 2H), 6.72 (s, 1H), 6.42 (d, J = 7.8 Hz, 1H), 5.26 (s, 2H), 5.08 (t, J = 5.7 Hz, 1H), 4.36 (d, J = 5.7 Hz, 2H), 3.87 (s, 2H), 3.71 (s, 3H); $^{13}C\{^{1}H\}$ NMR (100 MHz, DMSO-d_6, 298 K): δ = 158.2, 149.1, 144.0, 134.8, 130.1, 130.0, 114.6, 113.9, 113.6, 112.2, 62.8, 55.0, 37.4.

2-((4-Methoxybenzyl)thio)-5-(trifluoromethoxy)aniline (1o). The title compound was prepared in two steps from 1-fluoro-2-nitro-4-(trifluoromethoxy)benzene and purified as described below.

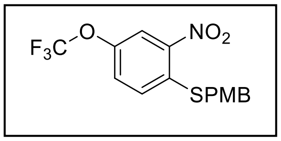

(4-Methoxybenzyl)(2-nitro-4-(trifluoromethoxy)phenyl)sulfane. The title compound was prepared according to the general procedure on a 5.832 g (25.9 mmol) scale. Once the reaction was determined to be complete by TLC, the solid was filtered off, washed with H_2O, followed by ethanol and finally diethyl ether to yield the product as a yellow powder in 96% yield (8.965 g, 24.94 mmol), m.p. 104-105 °C.

^{1}H NMR (400 MHz, DMSO-d_6, 298 K): δ = 8.20 (d, J = 2.2 Hz, 1H), 7.85 (d, J = 9.0 Hz, 1H), 7.78 (dd, J = 8.9, 1.8 Hz, 1H), 7.36 (d, J = 8.7 Hz, 2H), 6.95 – 6.87 (m, 2H), 4.35 (s, 2H), 3.74 (s, 3H); $^{13}C\{^{1}H\}$ NMR (100 MHz, DMSO-d_6, 298 K): δ = 158.8, 145.4, 144.6, 136.3, 130.5, 129.6, 127.0, 126.8, 119.9 (q,

J = 257.8 Hz), 118.7, 114.1; $^{19}F\{^1H\}$ NMR (376 MHz, DMSO-d_6, 298 K, referenced to C_6H_5F): δ = -57.44.

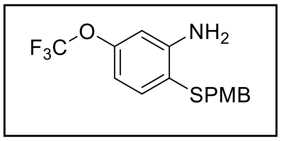

2-((4-Methoxybenzyl)thio)-5-(trifluoromethoxy)aniline (1o). The title compound was prepared according to the general procedure on a 8.805 g (24.5 mmol) scale. Purification by washing with hexanes followed by minimal amounts of diethyl ether yielded the product as a salmon pink powder in 95% yield (7.656 g, 23.25 mmol), m.p. 70-71 °C.

^1H NMR (400 MHz, DMSO-d_6, 298 K): δ = 7.16-7.13 (m, 3H), 6.81 (d, J = 8.6 Hz, 2H), 6.70-6.63 (m, 1H), 6.41-6.32 (m, 1H), 5.70 (s, 2H), 3.91 (s, 2H), 3.71 (s, 3H); $^{13}C\{^1H\}$ NMR (100 MHz, DMSO-d_6, 298 K): δ = 158.3, 150.7, 149.4, 136.2, 130.0, 129.6, 120.1 (q, J = 255.9 Hz), 114.8, 113.6, 107.7, 105.4, 55.0, 37.0; $^{19}F\{^1H\}$ NMR (376 MHz, DMSO-d_6, 298 K, referenced to C_6H_5F): δ = -56.57.

3-Amino-4-((4-methoxybenzyl)thio)benzenesulfonamide (1p). The title compound was prepared in two steps from 4-fluoro-3-nitrobenzenesulfonamide and purified as described below.

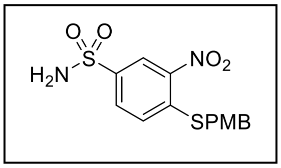

4-((4-Methoxybenzyl)thio)-3-nitrobenzenesulfonamide. The title compound was prepared according to the general procedure on a 4.942 g (22.45 mmol) scale. Once the reaction was determined to be complete by TLC, the solid was filtered off, washed with dilute aqueous HCl (approx 0.05 M), followed by ethanol and finally diethyl ether to yield the product quantitatively (7.957 g, 22.45 mmol) as a yellow powder, m.p. 214-215 °C.

^1H NMR (400 MHz, DMSO-d_6, 298 K): δ = 8.55 (d, J = 2.0 Hz, 1H), 8.03 (dd, J = 8.6, 2.0 Hz, 1H), 7.95 (d, J = 8.7 Hz, 1H), 7.58 (s, 2H), 7.39 (d, J = 8.7 Hz, 2H), 6.92 (d, J = 8.7 Hz, 2H), 4.39 (s, 2H), 3.74 (s, 3H); $^{13}C\{^1H\}$ NMR (100 MHz, DMSO-d_6, 298 K): δ = 158.8, 144.2, 141.5, 140.8, 130.6, 130.3, 128.4, 126.6, 123.1, 114.1, 55.1, 35.6.

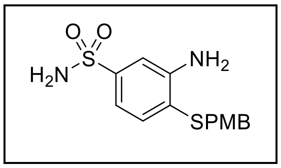

3-Amino-4-((4-methoxybenzyl)thio)benzenesulfonamide (1p). The title compound was prepared according to the general procedure on an 8.321 g (23.48 mmol) scale. Due to the poor solubility of the product in ethanol, filtration through celite was performed with hot ethanol. After concentration of the filtrate, water was added, and the reaction mixture extracted with dichloromethane which yielded some product upon evaporation. Additional compoun was recovered as solid from the aqueous phase upon acidification. The combined solid product fractions were washed with diethyl ether to yield the pure product as a white powder in 70% yield (5.333 g, 16.44 mmol), m.p. 167 °C.

^1H NMR (400 MHz, DMSO-d_6, 298 K): δ = 7.27 (d, J = 8.1 Hz, 1H), 7.24-7.17 (m, 4H), 7.15 (d, J = 1.7 Hz, 1H), 6.90 (dd, J = 8.1, 1.7 Hz, 1H), 6.84 (d, J = 8.5 Hz, 2H), 5.62 (s, 2H), 4.03 (s, 2H), 3.72 (s, 3H); $^{13}C\{^1H\}$ NMR (100 MHz, DMSO-d_6, 298 K): δ = 158.3, 148.2, 143.7, 132.7, 130.1, 129.3, 120.6, 113.7, 113.0, 110.7, 55.0, 36.3.

References

(1) Ardón-Muñoz, L. G.; Bolliger, J. L. Synthesis of Benzo[4,5]Thiazolo[2,3-c][1,2,4]Triazole Derivatives via C-H Bond Functionalization of Disulfide Intermediates. *Molecules* **2022**, *27* (5), 1464. https://doi.org/10.3390/molecules27051464.

3. NMR Spectra

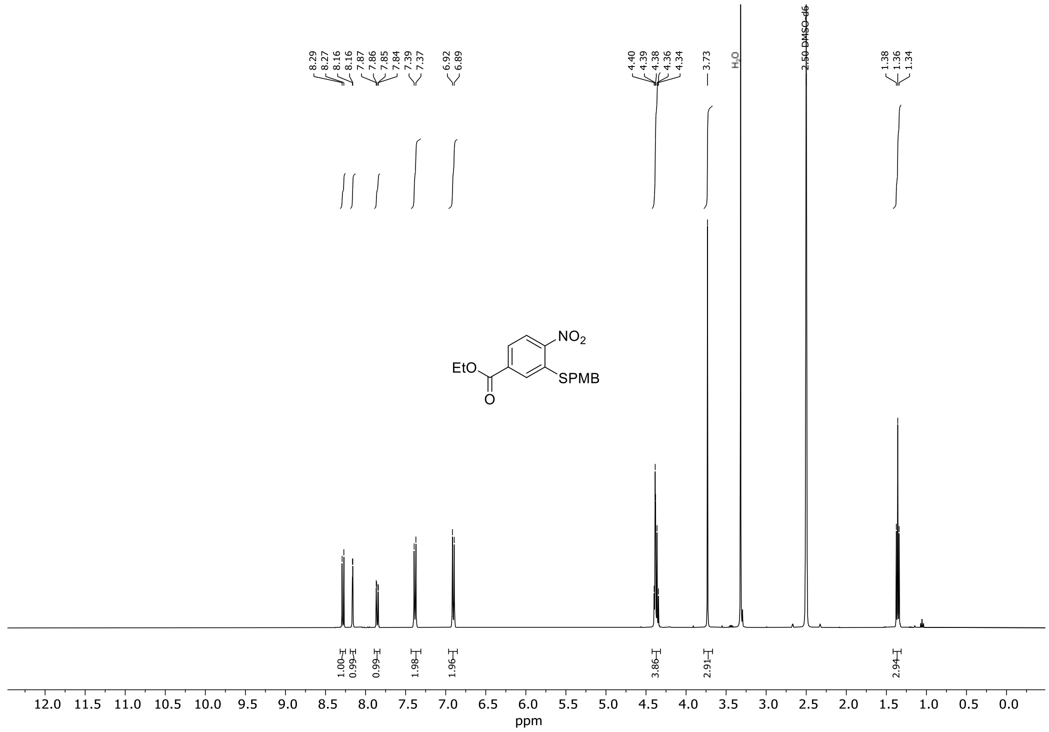

Figure S001: ^{1}H NMR spectrum of ethyl 3-((4-methoxybenzyl)thio)-4-nitrobenzoate (400 MHz, DMSO-d_6, 298 K).

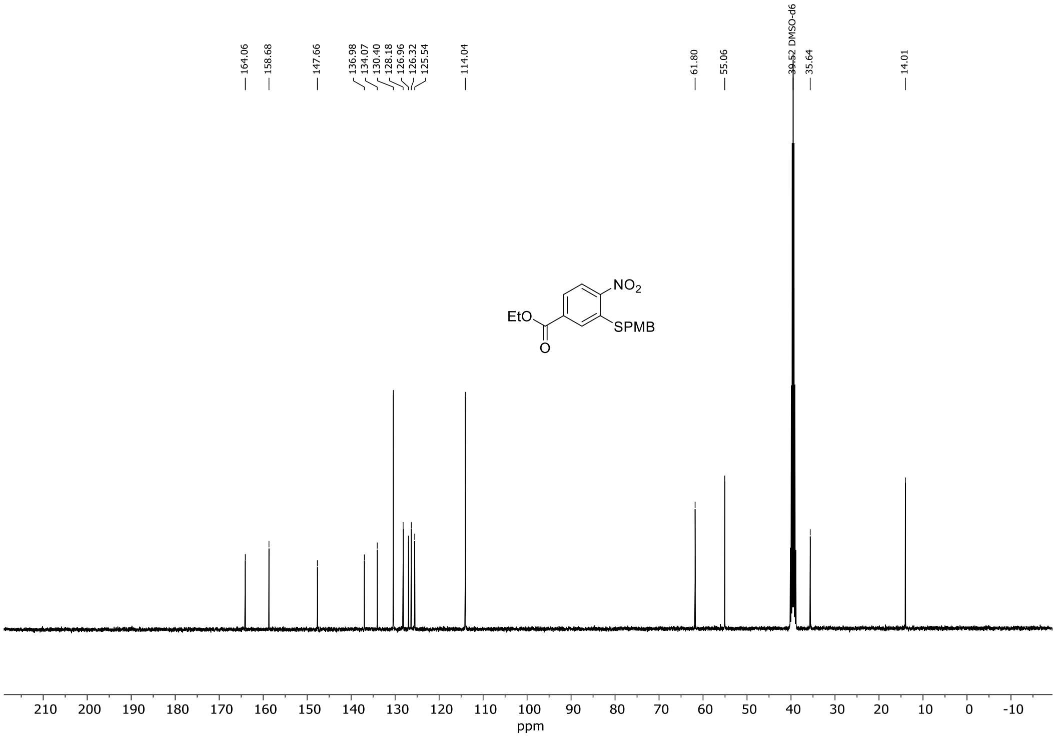

Figure S002: $^{13}C\{^{1}H\}$ NMR spectrum of ethyl 3-((4-methoxybenzyl)thio)-4-nitrobenzoate (100 MHz, DMSO-d_6, 298 K).

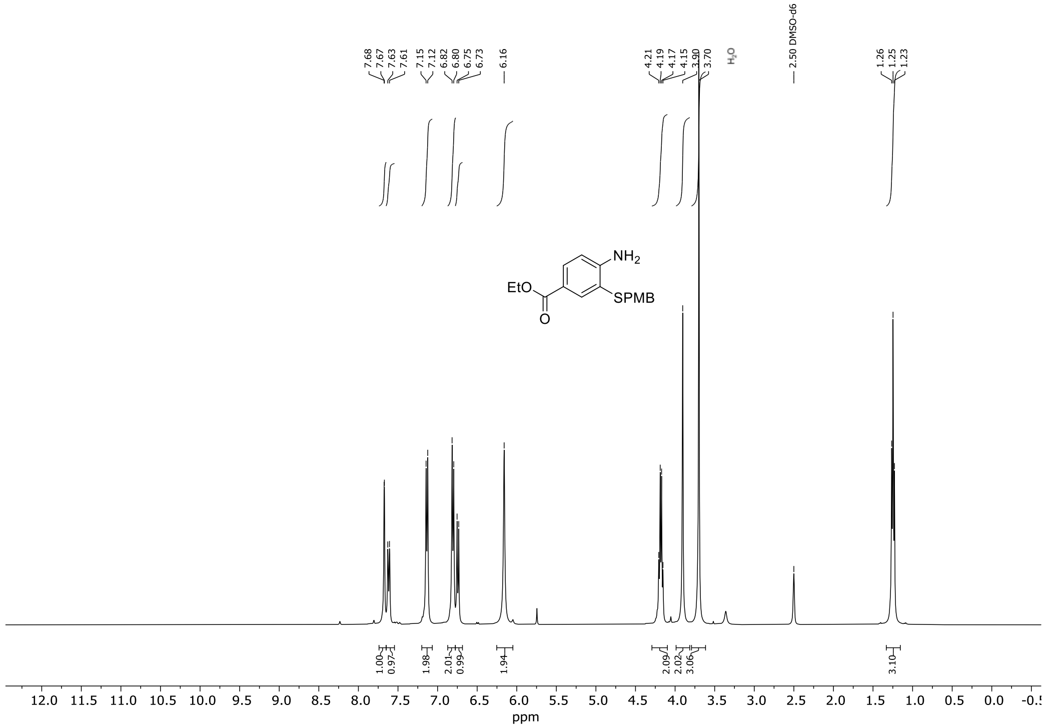

Figure S003: ^{1}H NMR spectrum of ethyl 4-amino-3-((4-methoxybenzyl)thio)benzoate (**1l)** (400 MHz, DMSO-d_6, 298 K).

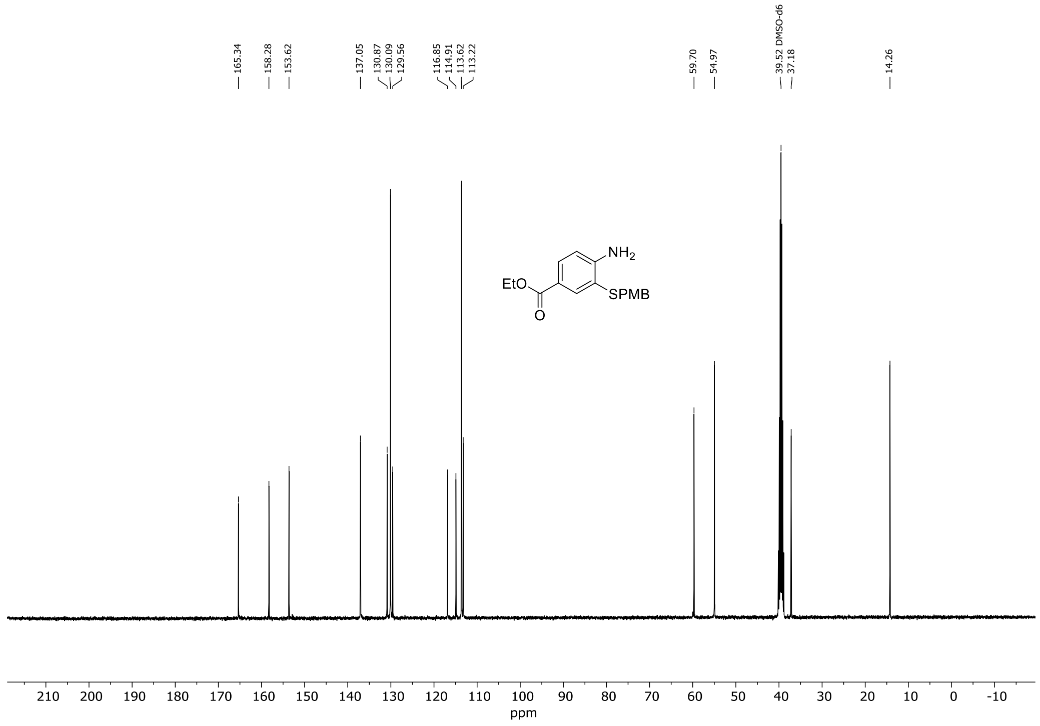

Figure S004: ^{13}C{^{1}H} NMR spectrum of ethyl 4-amino-3-((4-methoxybenzyl)thio)benzoate (**1l)** (100 MHz, DMSO-d_6, 298 K).

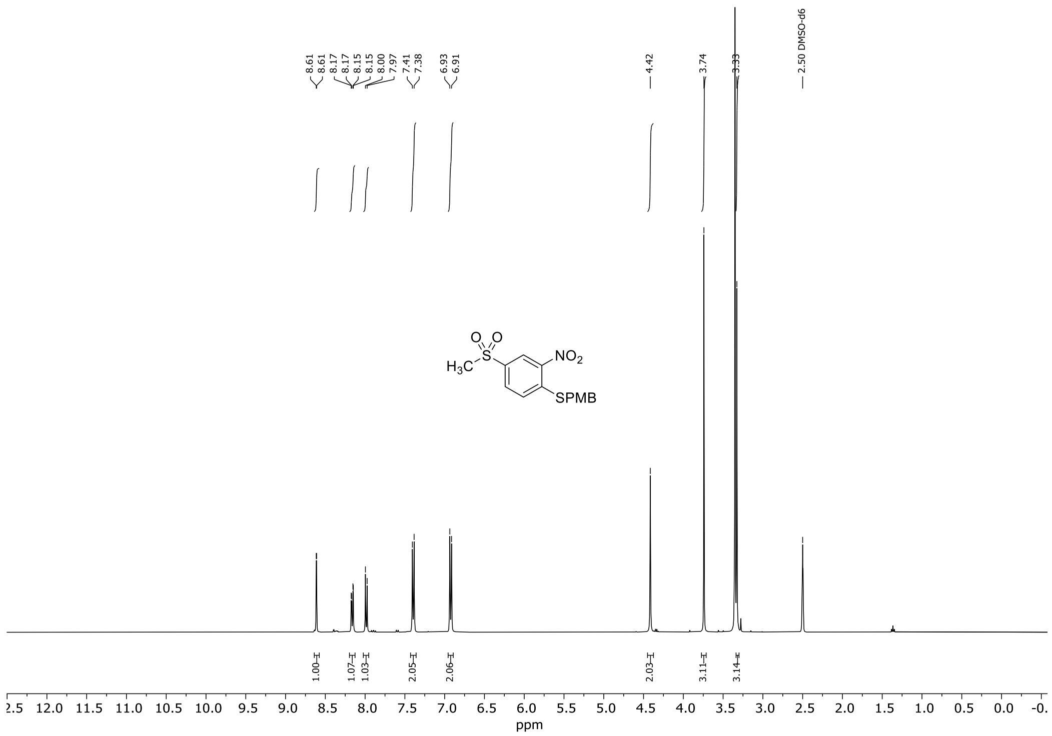

Figure S005: ^{1}H NMR spectrum of (4-methoxybenzyl)(4-(methylsulfonyl)-2-nitrophenyl)sulfane (400 MHz, DMSO-d_6, 298 K).

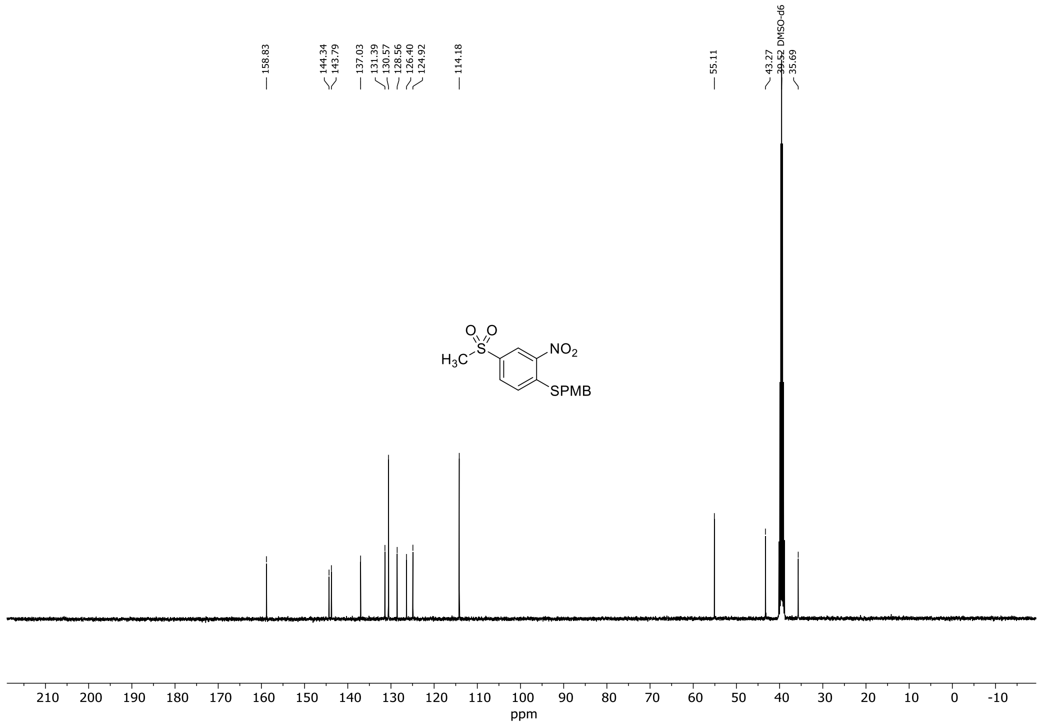

Figure S006: $^{13}C\{^{1}H\}$ NMR spectrum of (4-methoxybenzyl)(4-(methylsulfonyl)-2-nitrophenyl)sulfane (100 MHz, DMSO-d_6, 298 K).

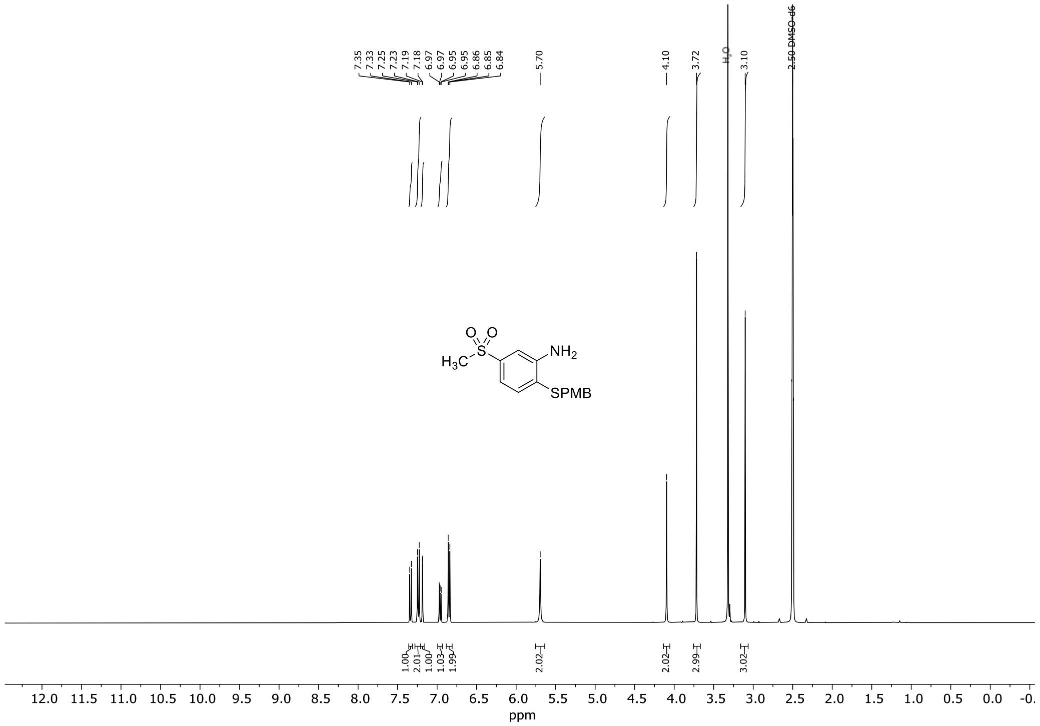

Figure S007: ^{1}H NMR spectrum of 2-((4-methoxybenzyl)thio)-5-(methylsulfonyl)aniline (**1m**) (400 MHz, DMSO-d_6, 298 K).

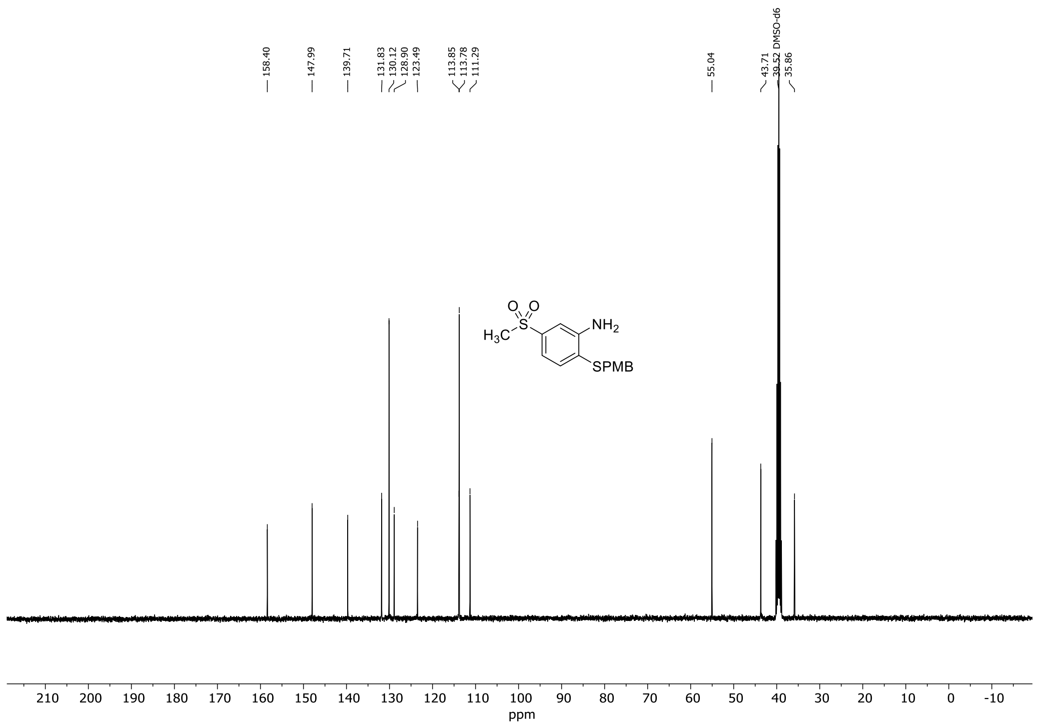

Figure S008: ^{13}C{^{1}H} NMR spectrum of 2-((4-methoxybenzyl)thio)-5-(methylsulfonyl)aniline (**1m**) (100 MHz, DMSO-d_6, 298 K).

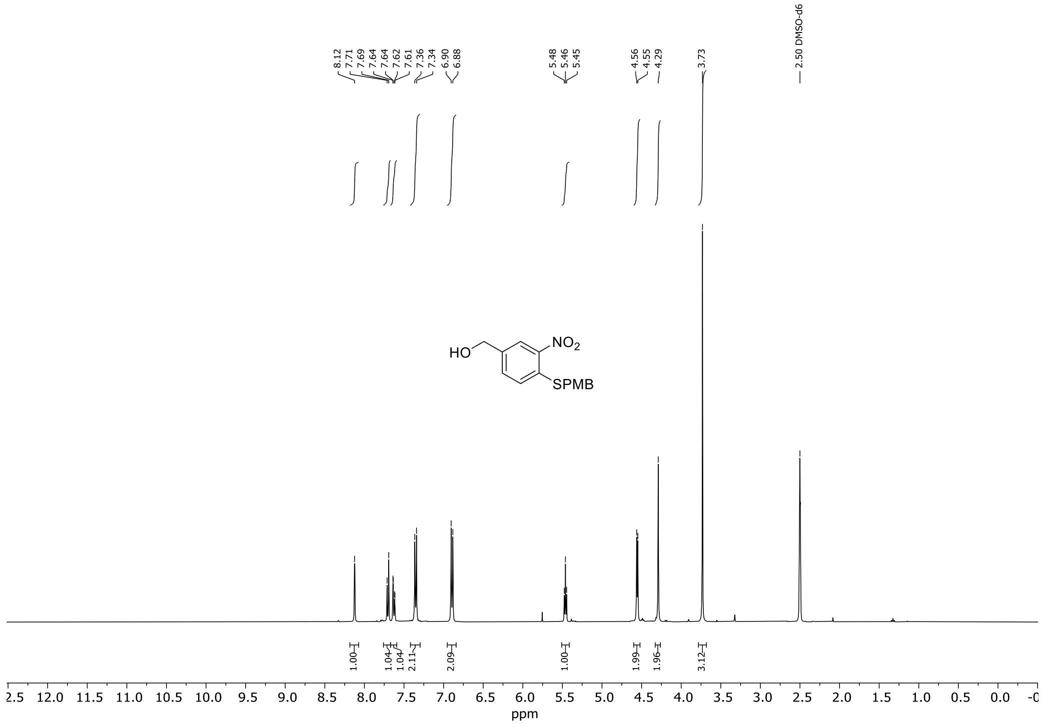

Figure S009: ^{1}H NMR spectrum of ((4-((4-methoxybenzyl)thio)-3-nitrophenyl)methanol (400 MHz, DMSO-d_6, 298 K).

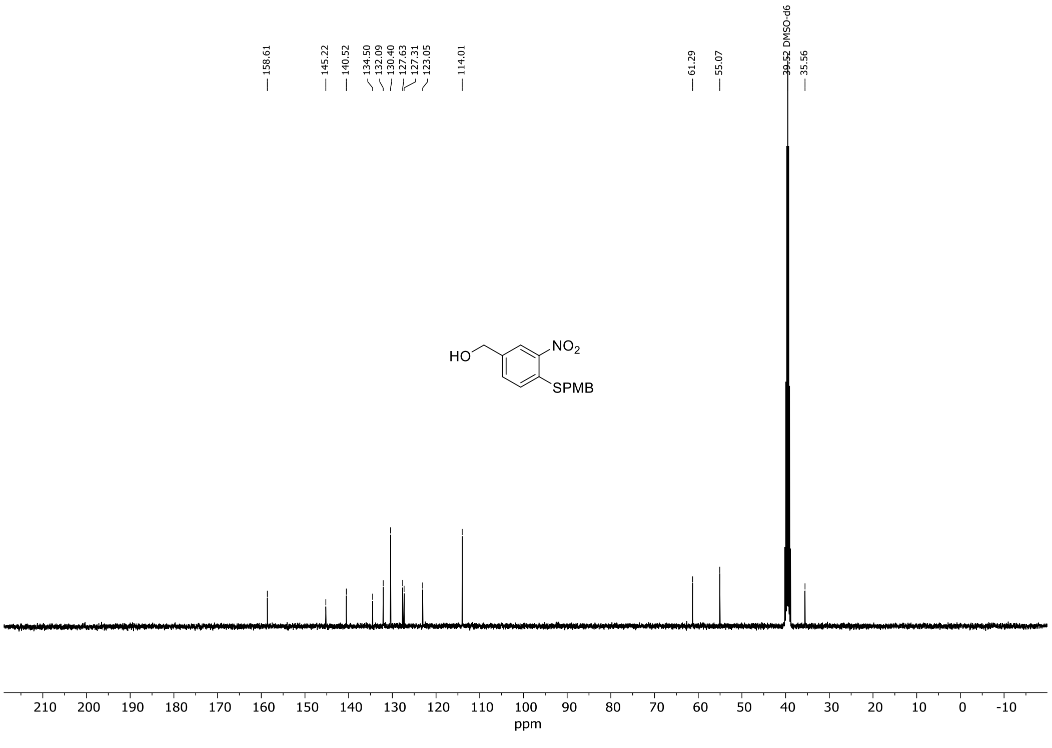

Figure S010: ^{13}C{^{1}H} NMR spectrum of ((4-((4-methoxybenzyl)thio)-3-nitrophenyl)methanol (100 MHz, DMSO-d_6, 298 K).

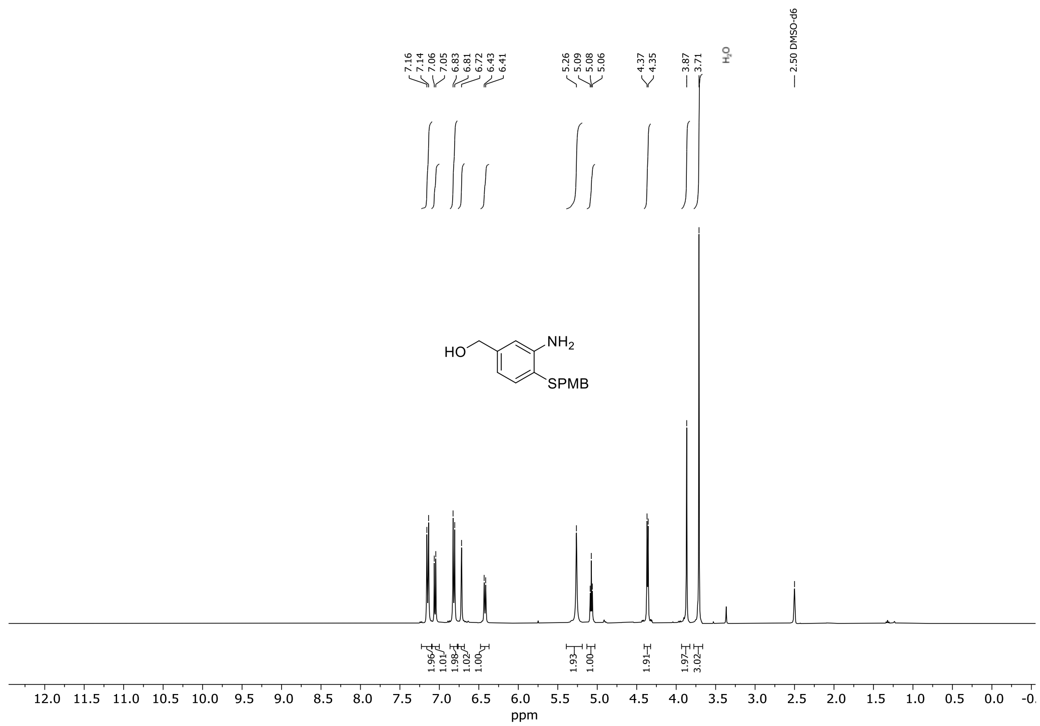

Figure S011: ^{1}H NMR spectrum of (3-amino-4-((4-methoxybenzyl)thio)phenyl)methanol (**1n**) (400 MHz, DMSO-d_6, 298 K).

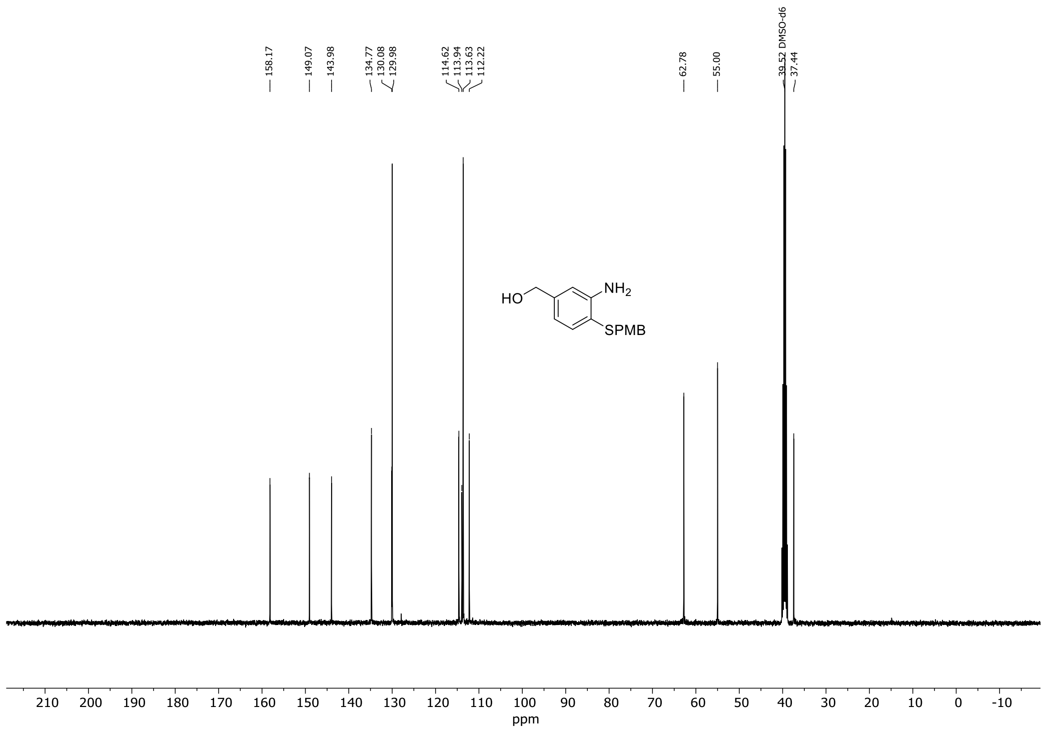

Figure S012: $^{13}C\{^{1}H\}$ NMR spectrum of (3-amino-4-((4-methoxybenzyl)thio)phenyl)methanol (**1n**) (100 MHz, DMSO-d_6, 298 K).

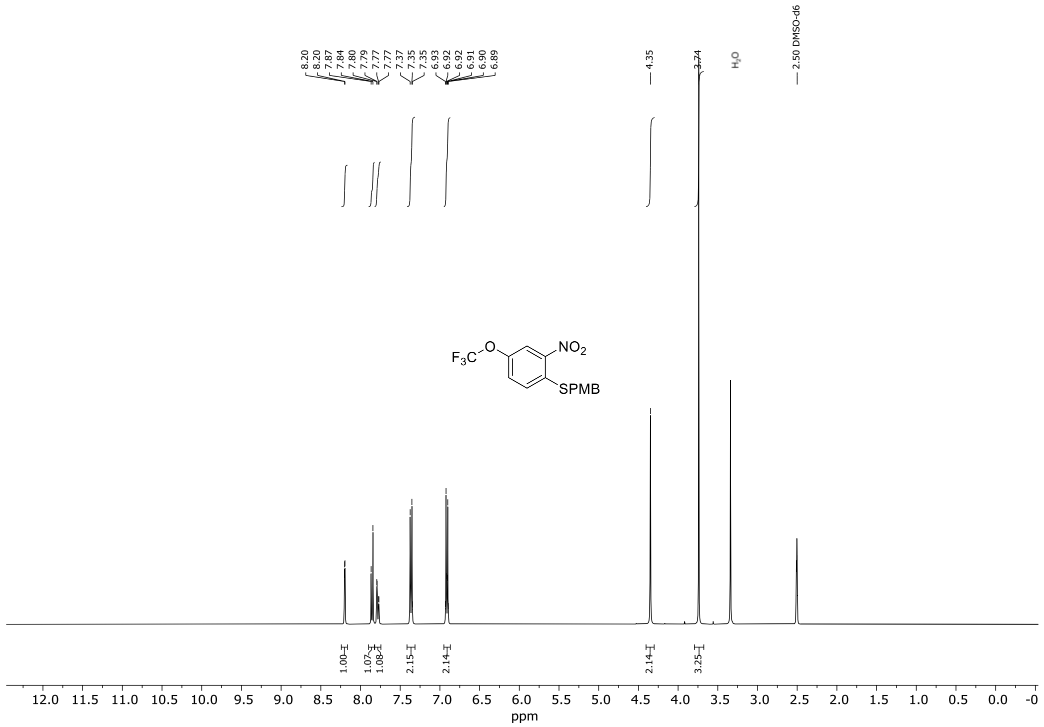

Figure S013: ^{1}H NMR spectrum of (4-methoxybenzyl)(2-nitro-4-(trifluoromethoxy)phenyl)sulfane (400 MHz, DMSO-*d*$_6$, 298 K).

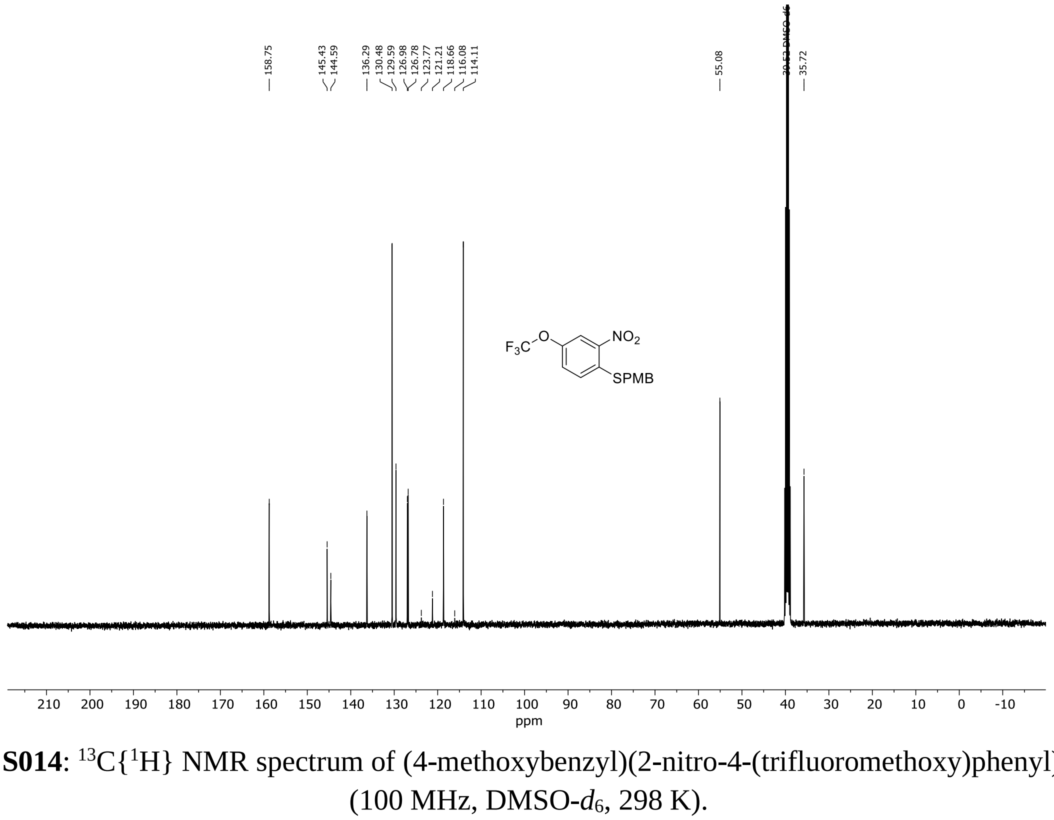

Figure S014: $^{13}C\{^{1}H\}$ NMR spectrum of (4-methoxybenzyl)(2-nitro-4-(trifluoromethoxy)phenyl)sulfane (100 MHz, DMSO-*d*$_6$, 298 K).

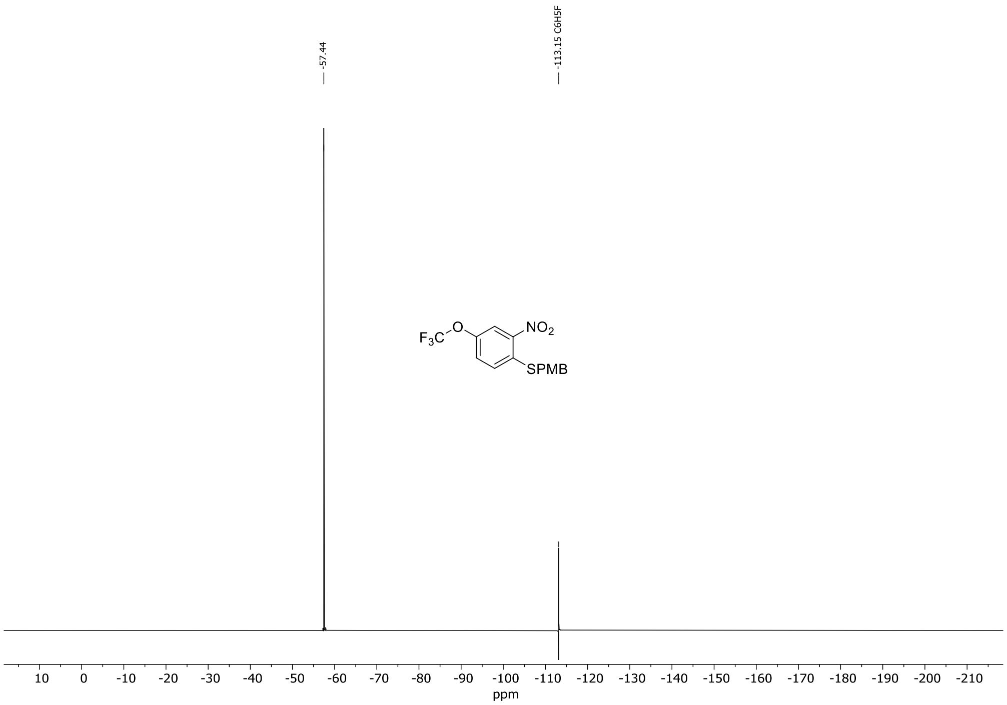

Figure S015: $^{19}F\{^1H\}$ NMR spectrum of (4-methoxybenzyl)(2-nitro-4-(trifluoromethoxy)phenyl)sulfane (376 MHz, DMSO-d_6, 298 K).

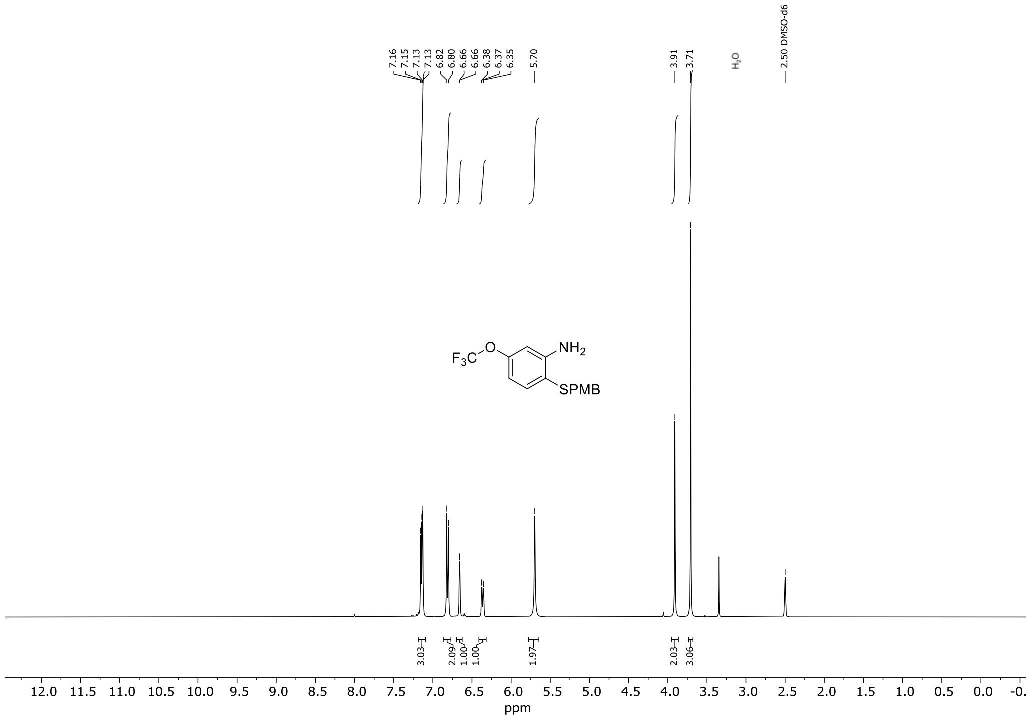

Figure S016: ^1H NMR spectrum of 2-((4-methoxybenzyl)thio)-5-(trifluoromethoxy)aniline (**1o**) (400 MHz, DMSO-d_6, 298 K).

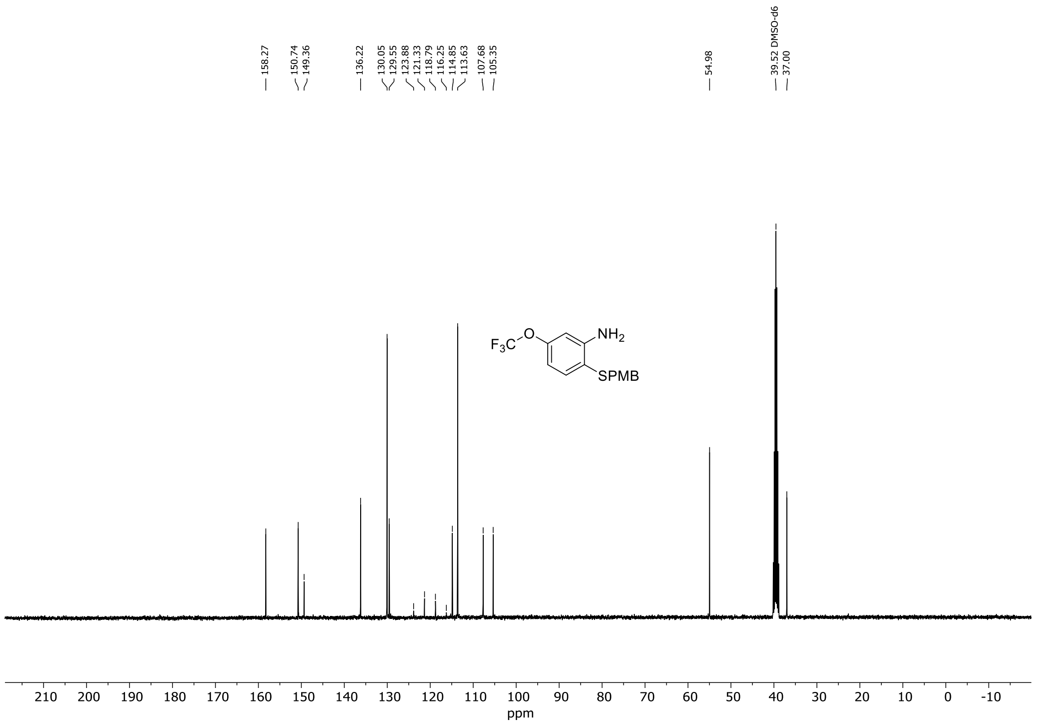

Figure S017: $^{13}C\{^{1}H\}$ NMR spectrum of 2-((4-methoxybenzyl)thio)-5-(trifluoromethoxy)aniline (**1o**) (100 MHz, DMSO-d_6, 298 K).

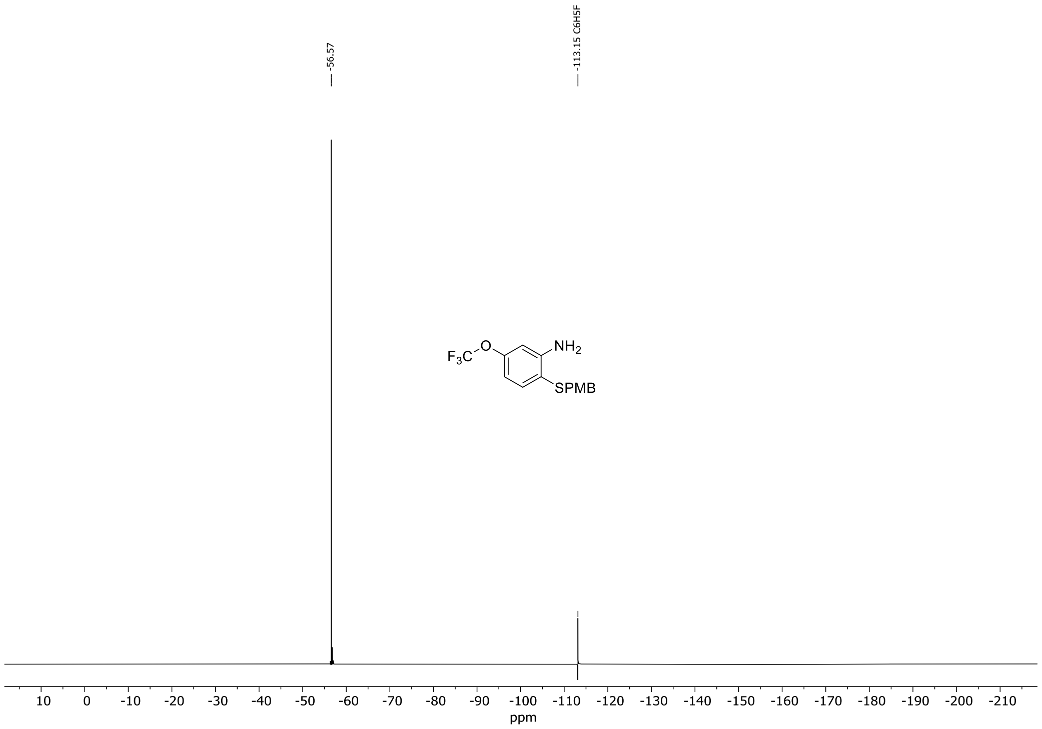

Figure S018: $^{19}F\{^{1}H\}$ NMR spectrum of 2-((4-methoxybenzyl)thio)-5-(trifluoromethoxy)aniline (**1o**) (376 MHz, DMSO-d_6, 298 K).

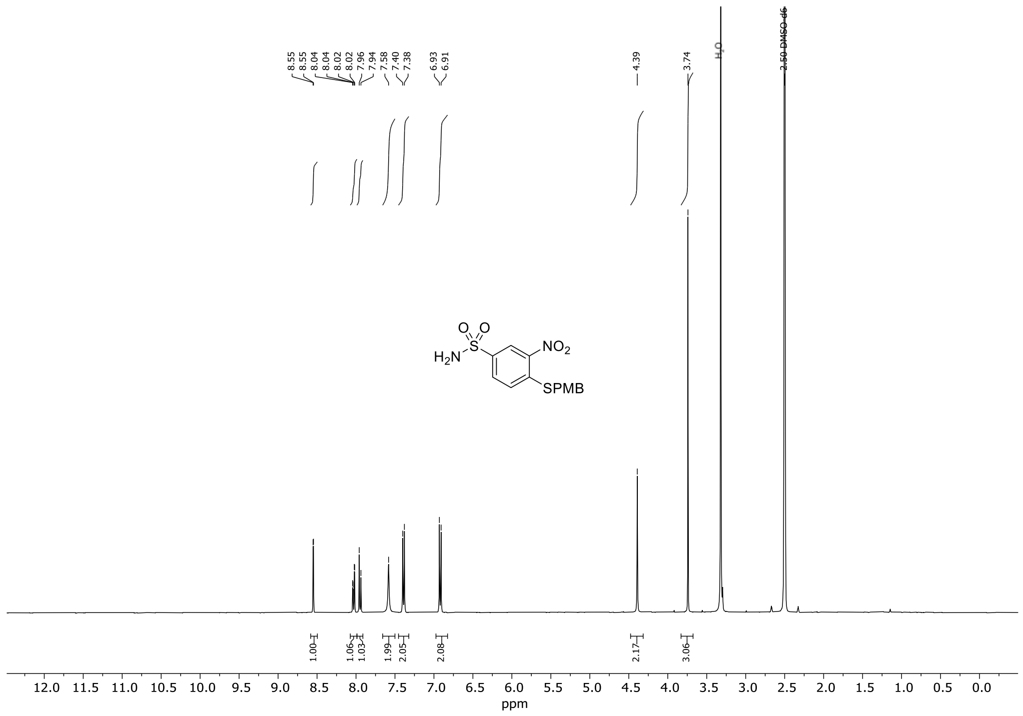

Figure S019: ^{1}H NMR spectrum of 4-((4-methoxybenzyl)thio)-3-nitrobenzenesulfonamide (400 MHz, DMSO-d_6, 298 K).

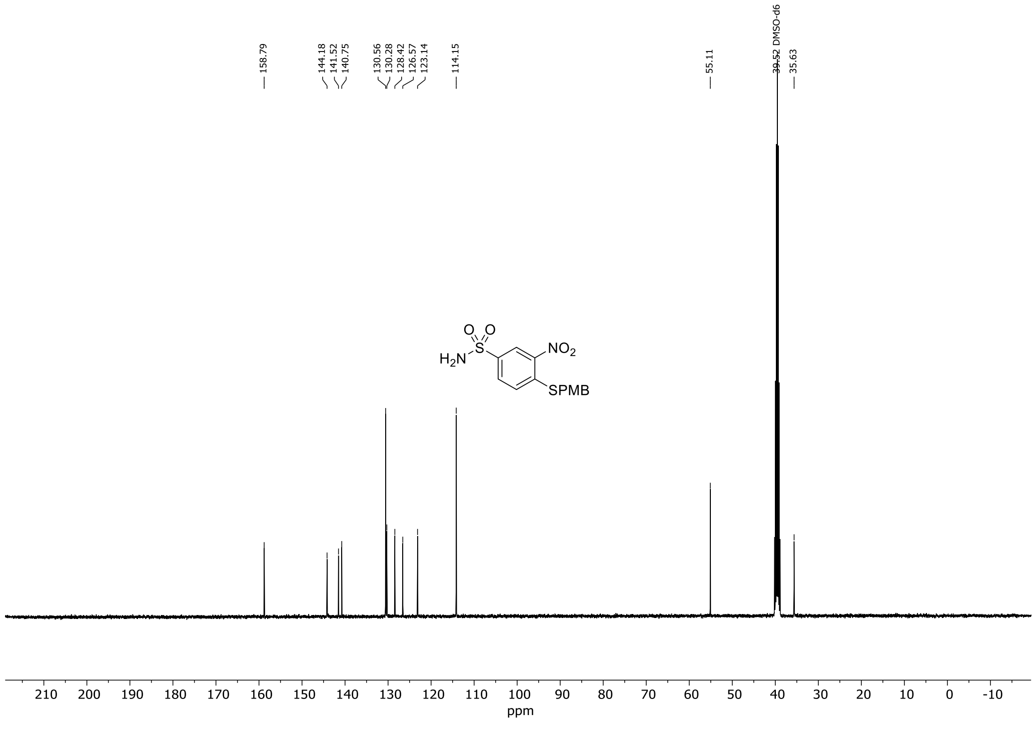

Figure S020: $^{13}C\{^{1}H\}$ NMR spectrum of 4-((4-methoxybenzyl)thio)-3-nitrobenzenesulfonamide (100 MHz, DMSO-d_6, 298 K).

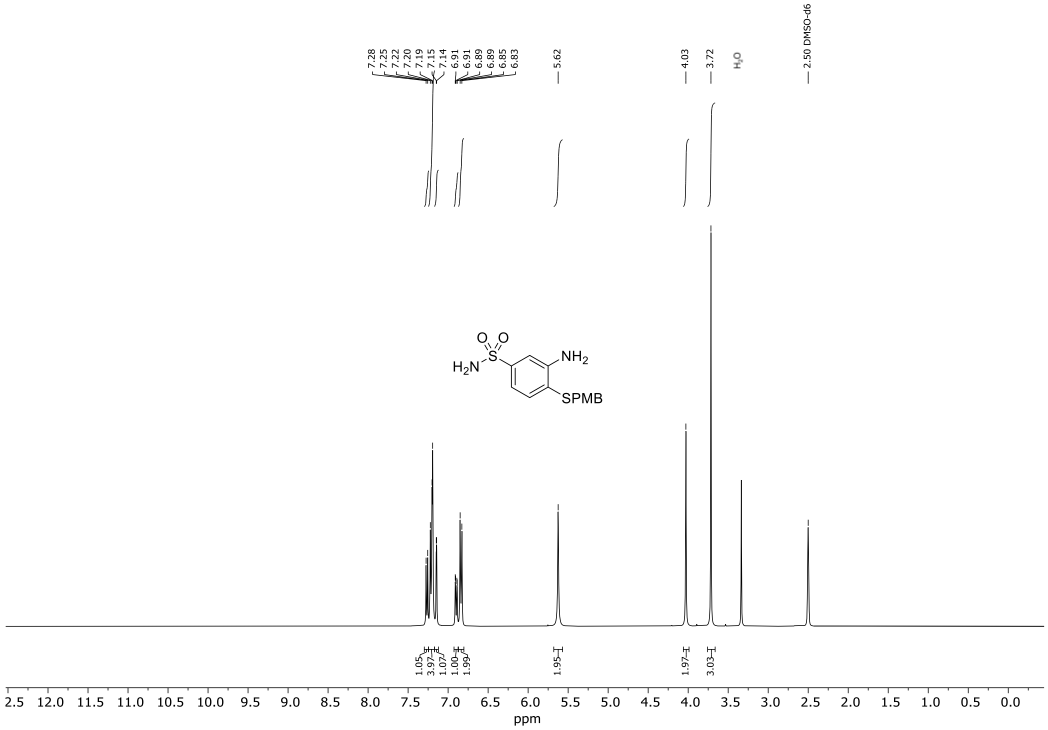

Figure S021: ^{1}H NMR spectrum of 3-amino-4-((4-methoxybenzyl)thio)benzenesulfonamide (**1p**) (400 MHz, DMSO-d_6, 298 K).

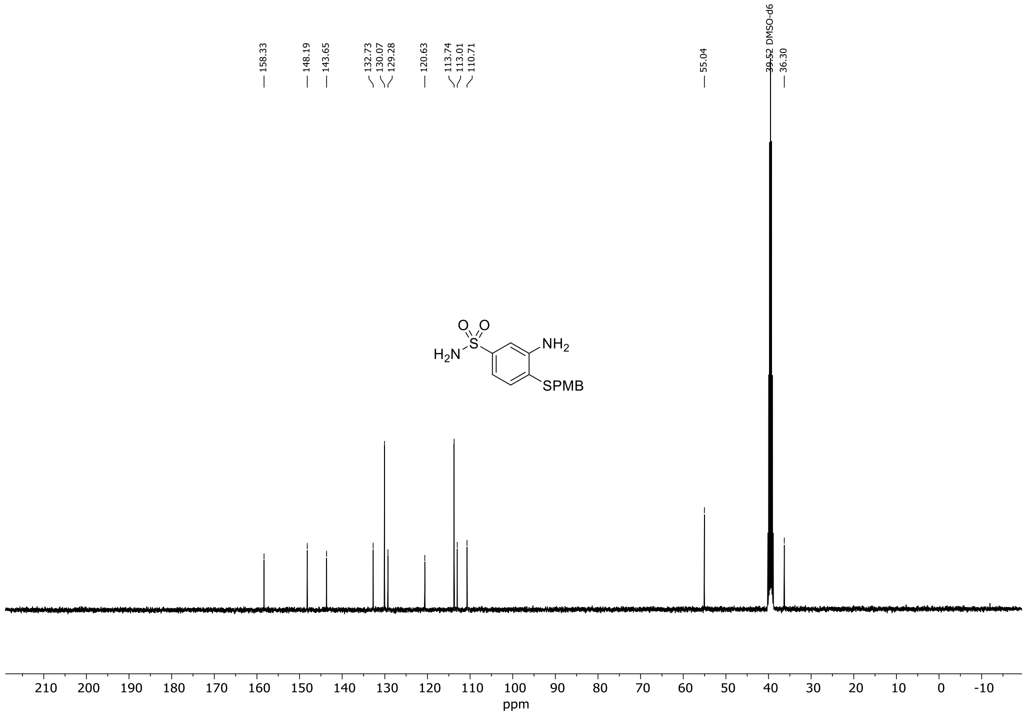

Figure S022: ^{13}C{^{1}H} NMR spectrum of 3-amino-4-((4-methoxybenzyl)thio)benzenesulfonamide (**1p**) (100 MHz, DMSO-d_6, 298 K).

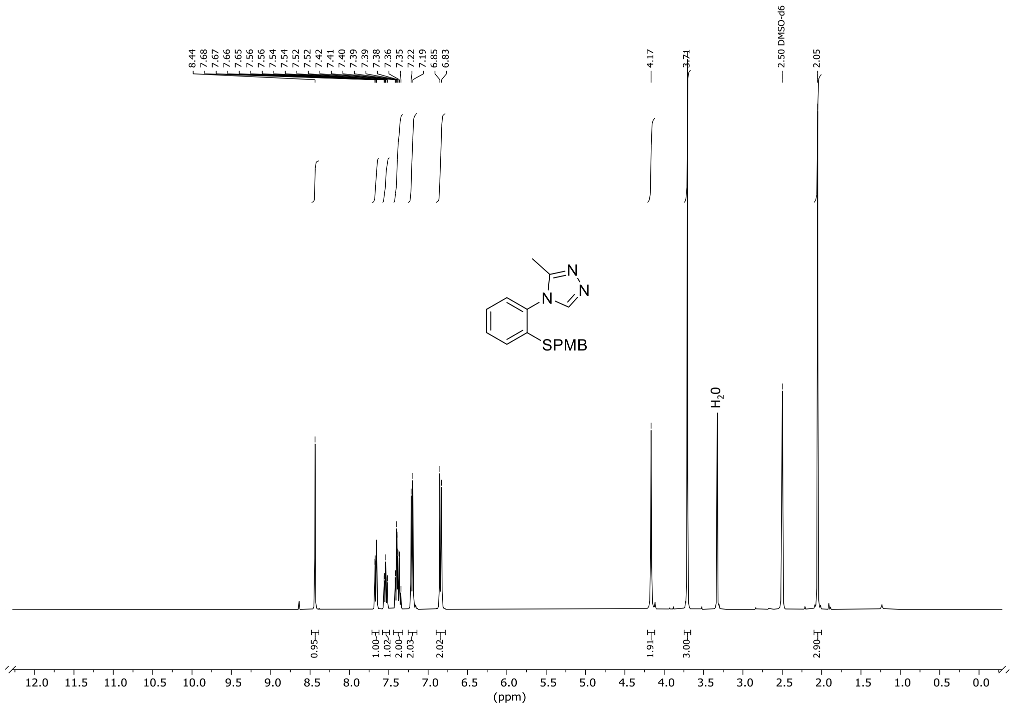

Figure S023: ^{1}H NMR spectrum of 4-(2-((4-methoxybenzyl)thio)phenyl)-3-methyl-4*H*-1,2,4-triazole (**2ab**) (400 MHz, DMSO-d_6, 298 K).

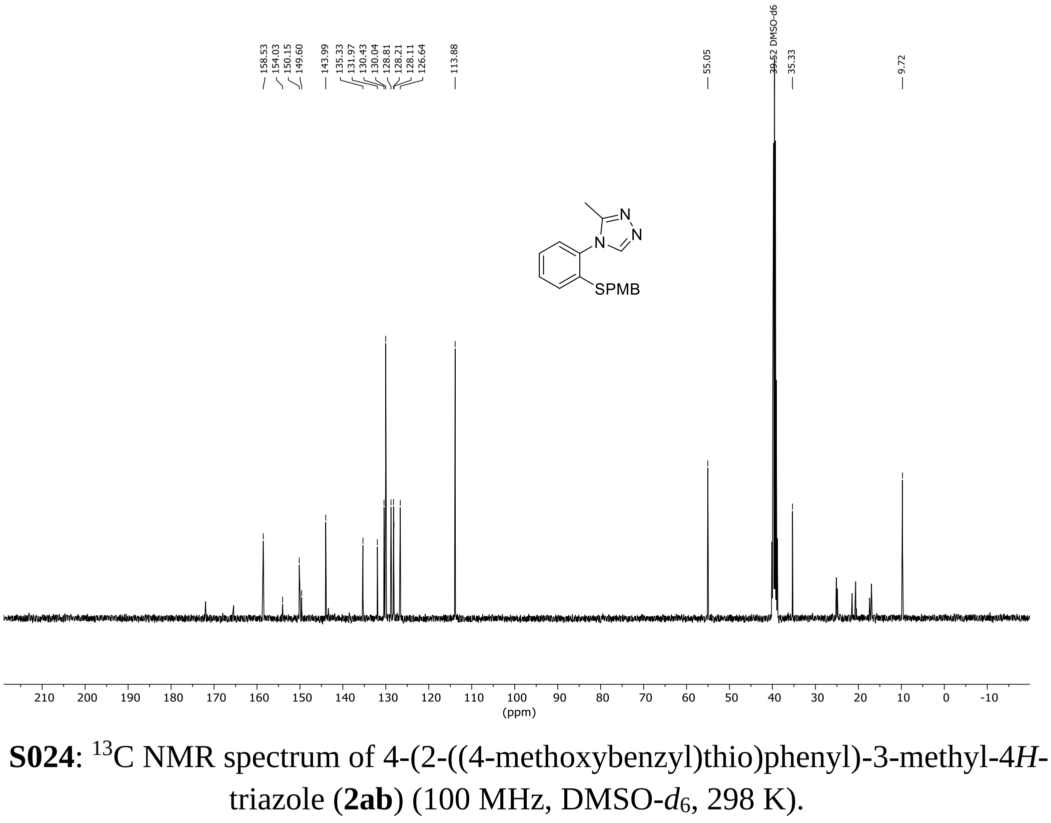

Figure S024: ^{13}C NMR spectrum of 4-(2-((4-methoxybenzyl)thio)phenyl)-3-methyl-4*H*-1,2,4-triazole (**2ab**) (100 MHz, DMSO-d_6, 298 K).

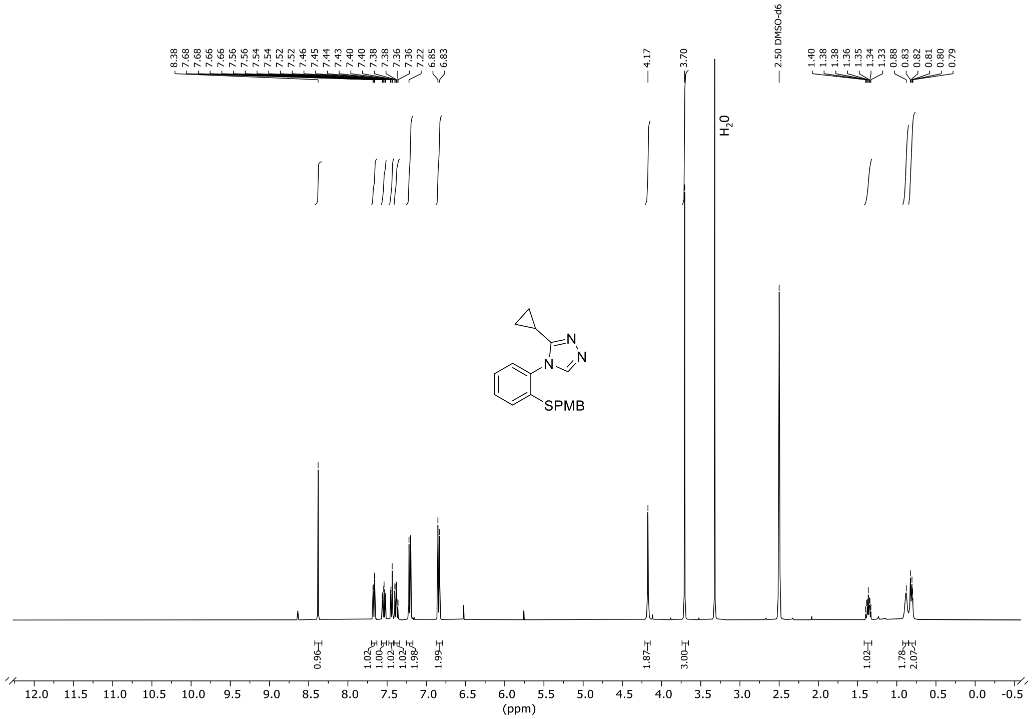

Figure S025: ^{1}H NMR spectrum of 3-cyclopropyl-4-(2-((4-methoxybenzyl)thio)phenyl)-4*H*-1,2,4-triazole (**2ac**) (400 MHz, DMSO-d_6, 298 K).

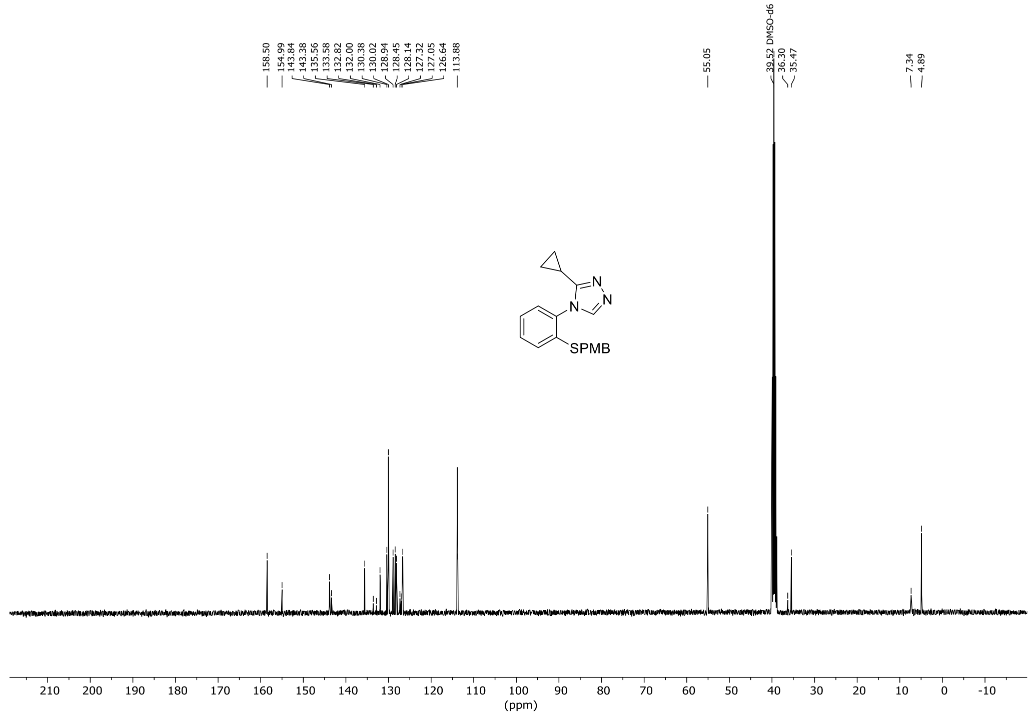

Figure S026: ^{13}C NMR spectrum of 3-cyclopropyl-4-(2-((4-methoxybenzyl)thio)phenyl)-4*H*-1,2,4-triazole (**2ac**) (100 MHz, DMSO-d_6, 298 K).

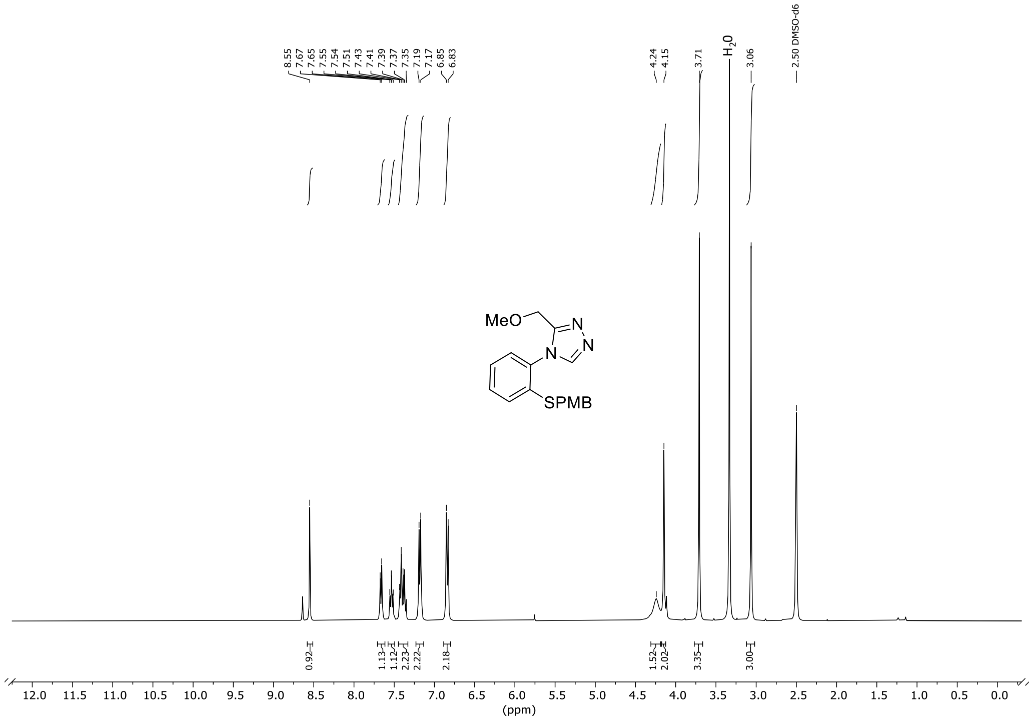

Figure S027: ^{1}H NMR spectrum of 4-(2-((4-methoxybenzyl)thio)phenyl)-3-(methoxymethyl)-4*H*-1,2,4-triazole (**2ad**) (400 MHz, DMSO-d_6, 298 K).

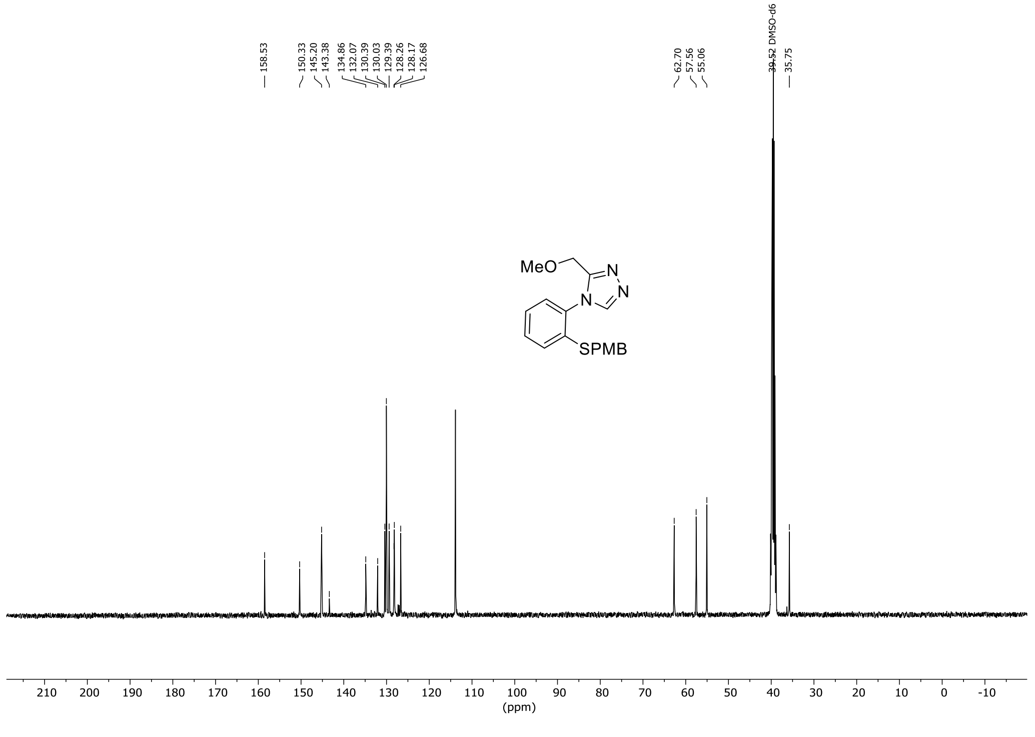

Figure S028: ^{13}C NMR spectrum of 4-(2-((4-methoxybenzyl)thio)phenyl)-3-(methoxymethyl)-4*H*-1,2,4-triazole (**2ad**) (100 MHz, DMSO-d_6, 298 K).

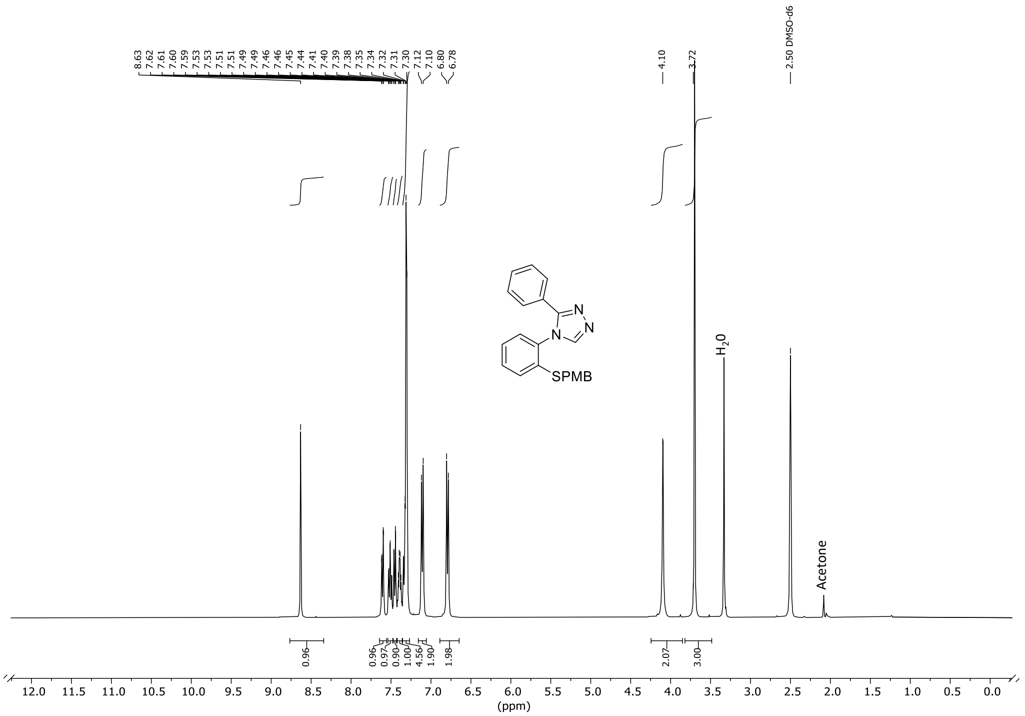

Figure S029: ^{1}H NMR spectrum of 4-(2-((4-methoxybenzyl)thio)phenyl)-3-phenyl-4*H*-1,2,4-triazole (**2ae**) (400 MHz, DMSO-d_6, 298 K).

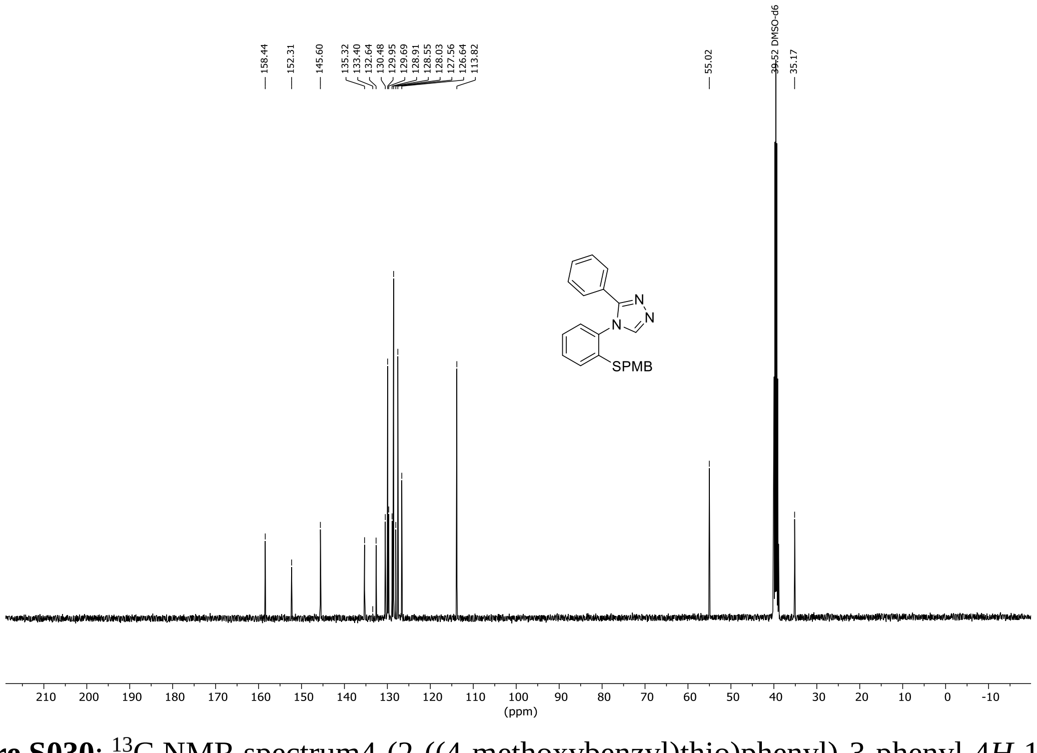

Figure S030: ^{13}C NMR spectrum4-(2-((4-methoxybenzyl)thio)phenyl)-3-phenyl-4*H*-1,2,4-triazole (**2ae**) (100 MHz, DMSO-d_6, 298 K).

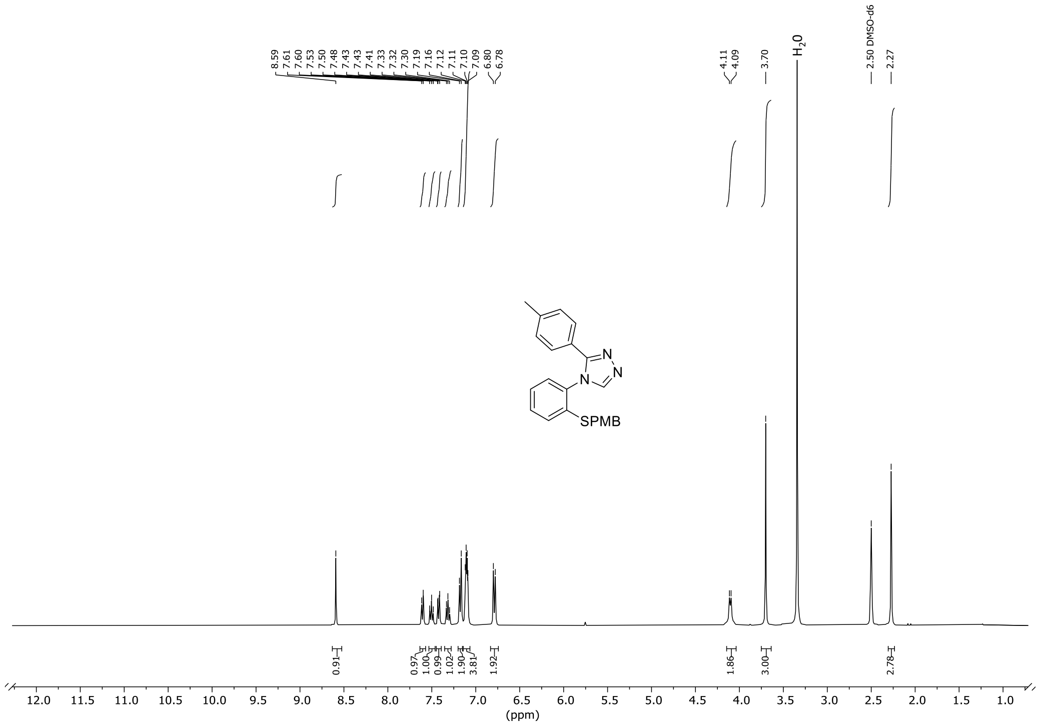

Figure S031: ^{1}H NMR spectrum of 4-(2-((4-methoxybenzyl)thio)phenyl)-3-(p-tolyl)-4*H*-1,2,4-triazole (**2af**) (400 MHz, DMSO-d_6, 298 K).

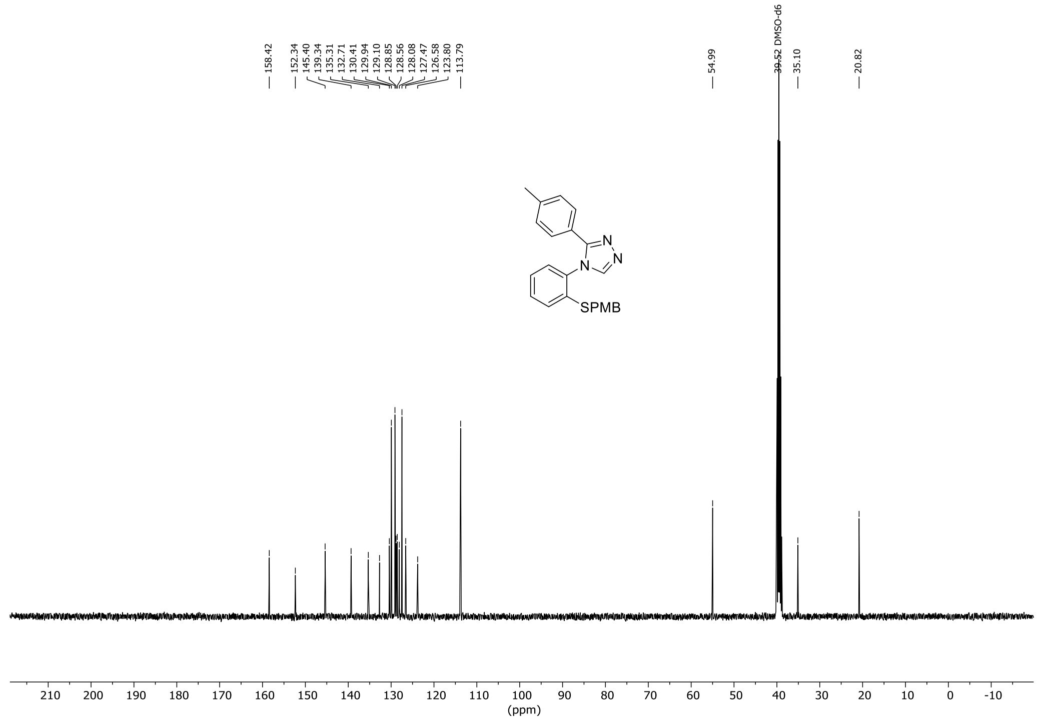

Figure S032: ^{13}C NMR spectrum of 4-(2-((4-methoxybenzyl)thio)phenyl)-3-(p-tolyl)-4*H*-1,2,4-triazole (**2af**) (100 MHz, DMSO-d_6, 298 K).

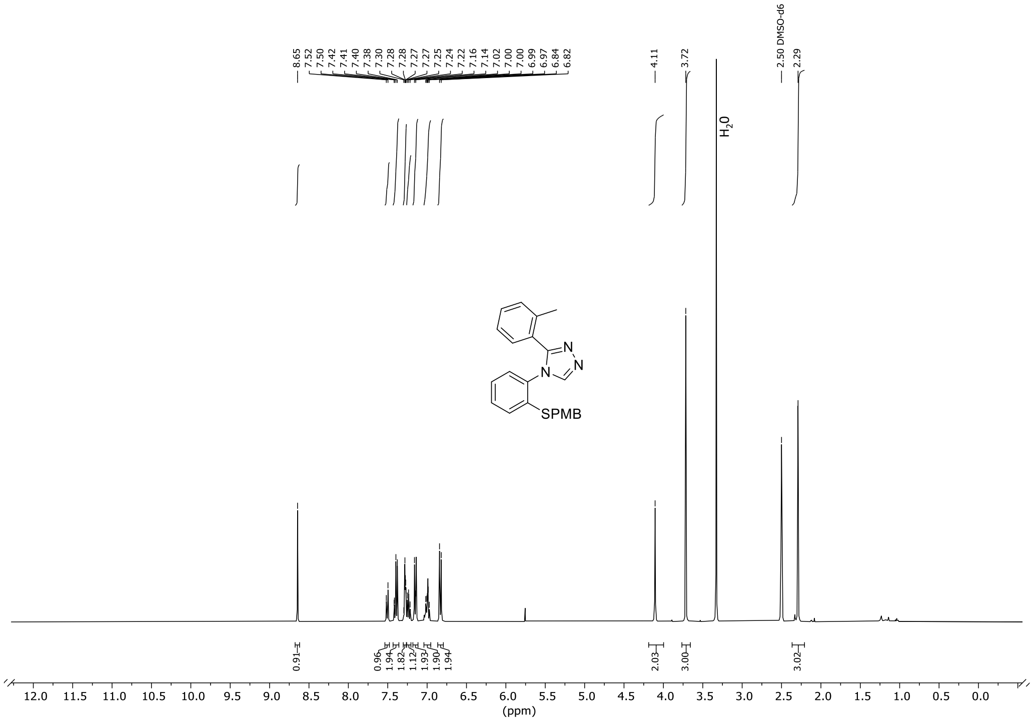

Figure S033: ^{1}H NMR spectrum of 4-(2-((4-methoxybenzyl)thio)phenyl)-3-(o-tolyl)-4*H*-1,2,4-triazole (**2ag**) (400 MHz, DMSO-d_6, 298 K).

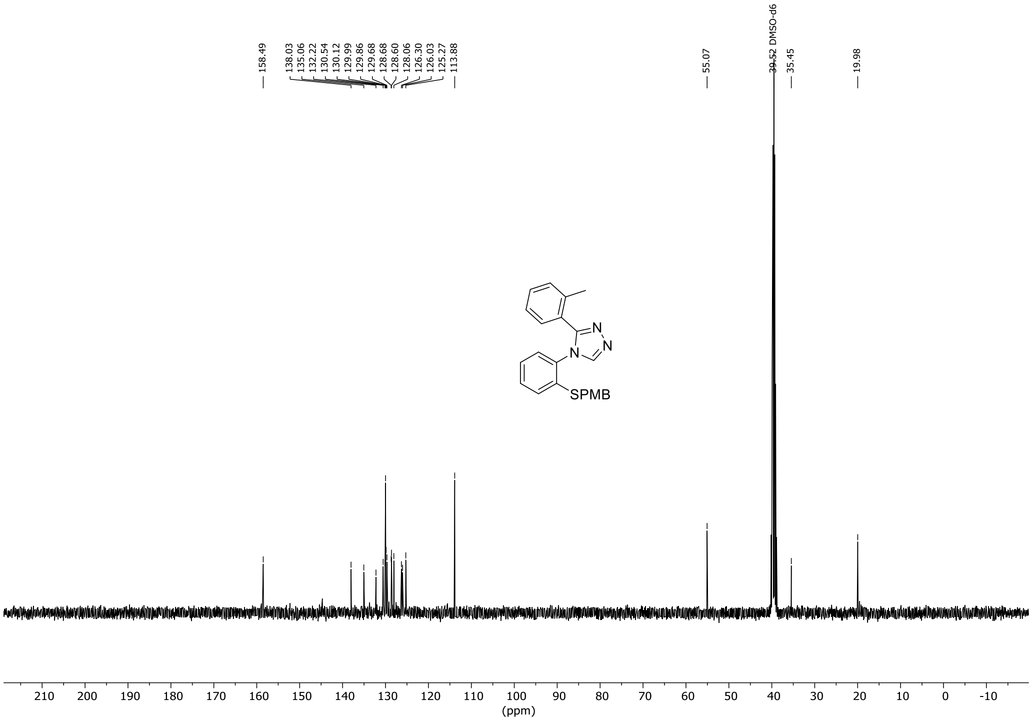

Figure S034: ^{13}C NMR spectrum 4-(2-((4-methoxybenzyl)thio)phenyl)-3-(o-tolyl)-4*H*-1,2,4-triazole (**2ag**) (100 MHz, DMSO-d_6, 298 K).

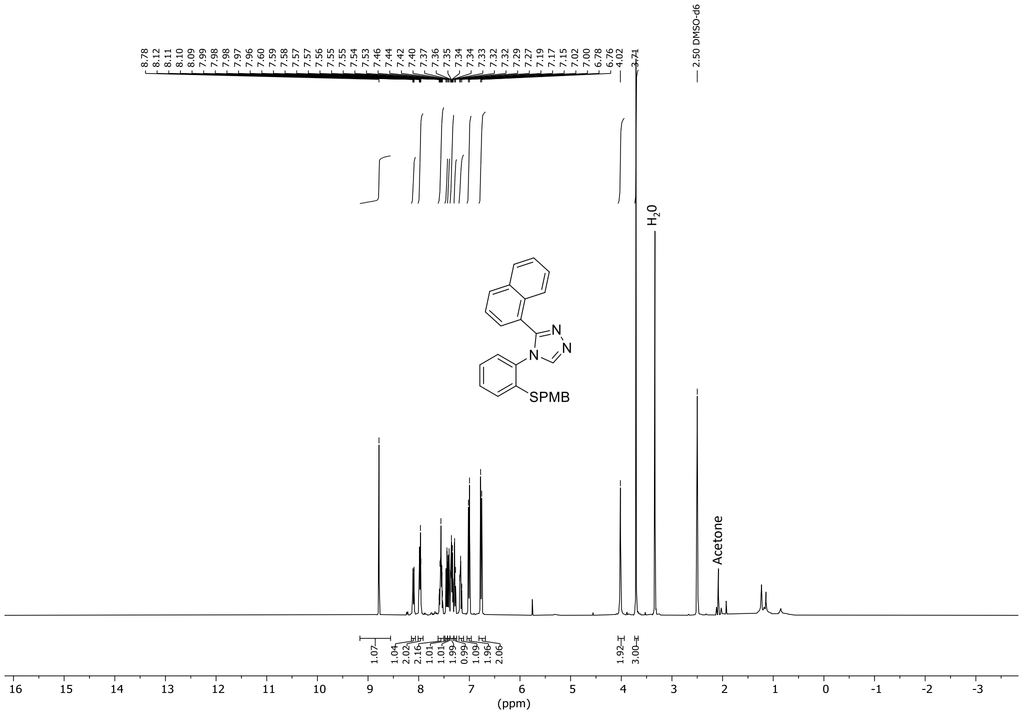

Figure S035: ^{1}H NMR spectrum of 4-(2-((4-methoxybenzyl)thio)phenyl)-3-(naphthalen-1-yl)-4*H*-1,2,4-triazole (**2ah**) (400 MHz, DMSO-d_6, 298 K).

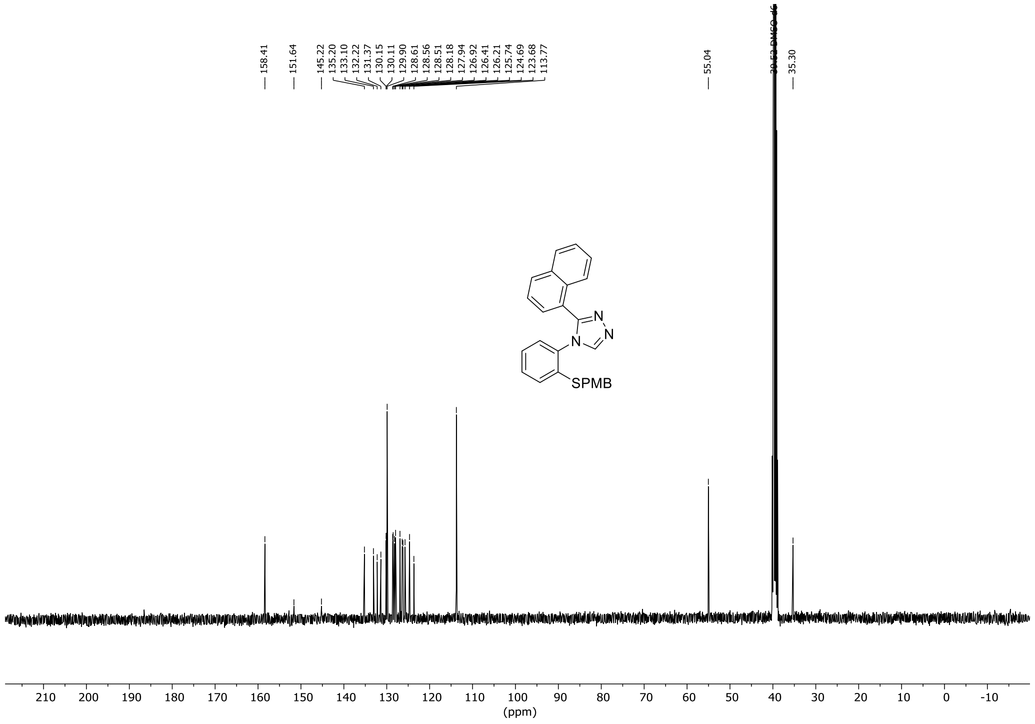

Figure S036: ^{13}C NMR spectrum of 4-(2-((4-methoxybenzyl)thio)phenyl)-3-(naphthalen-1-yl)-4*H*-1,2,4-triazole (**2ah**) (100 MHz, DMSO-d_6, 298 K).

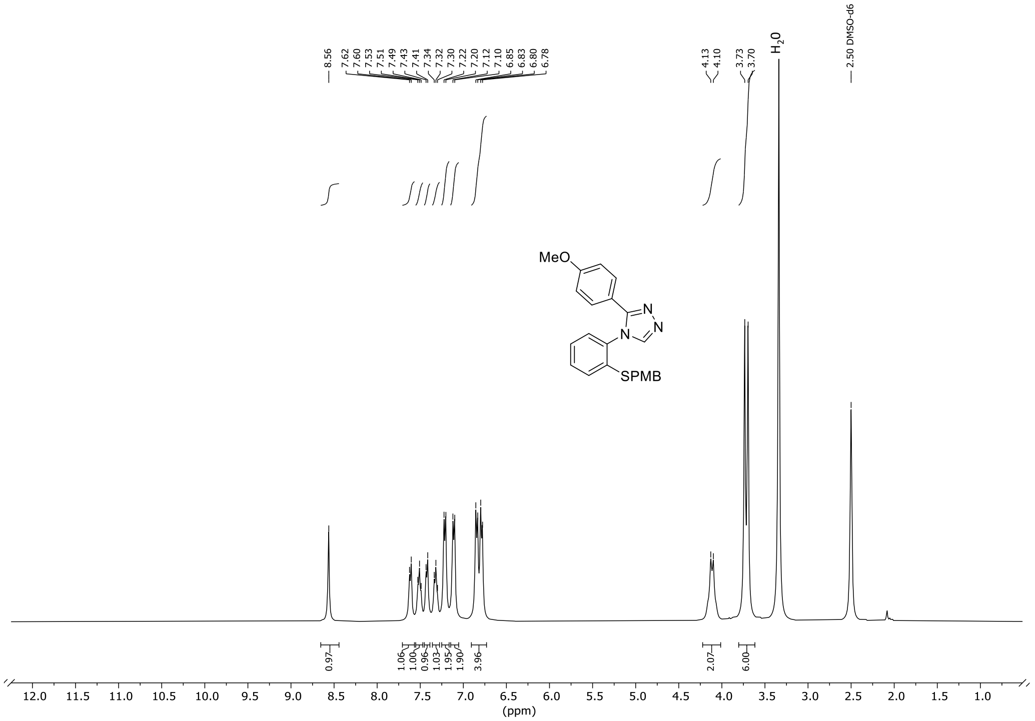

Figure S037: ^{1}H NMR spectrum of4-(2-((4-methoxybenzyl)thio)phenyl)-3-(4-methoxyphenyl)-4*H*-1,2,4-triazole (**2ai**) (400 MHz, DMSO-d_6, 298 K).

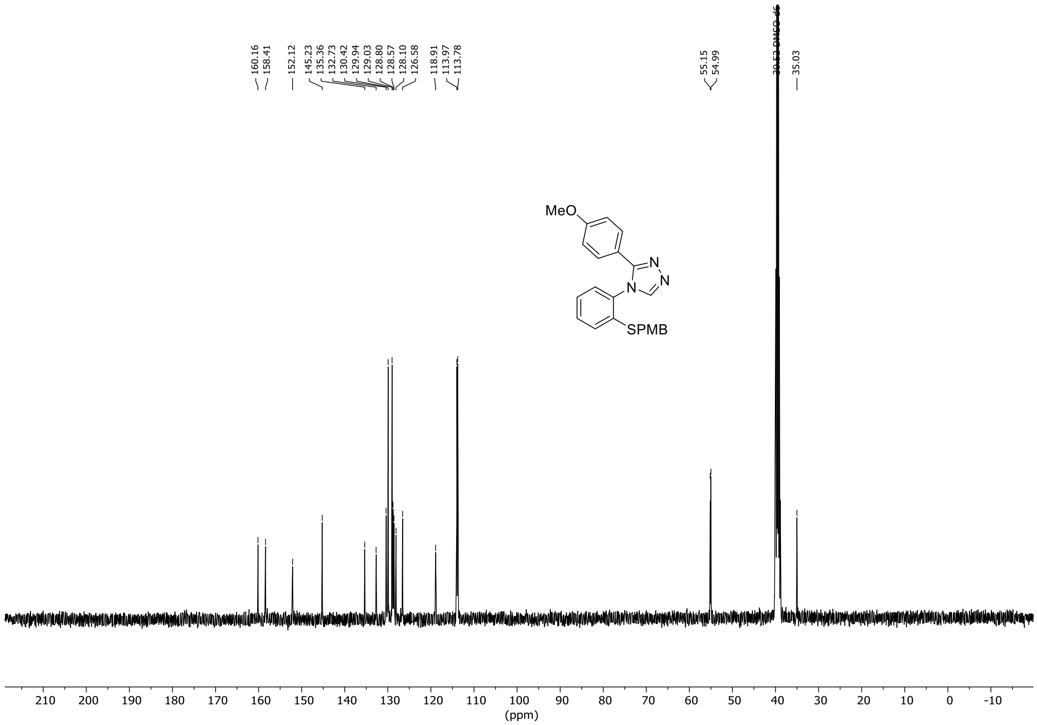

Figure S038: ^{13}C NMR spectrum 4-(2-((4-methoxybenzyl)thio)phenyl)-3-(4-methoxyphenyl)-4*H*-1,2,4-triazole (**2ai**) (100 MHz, DMSO-d_6, 298 K).

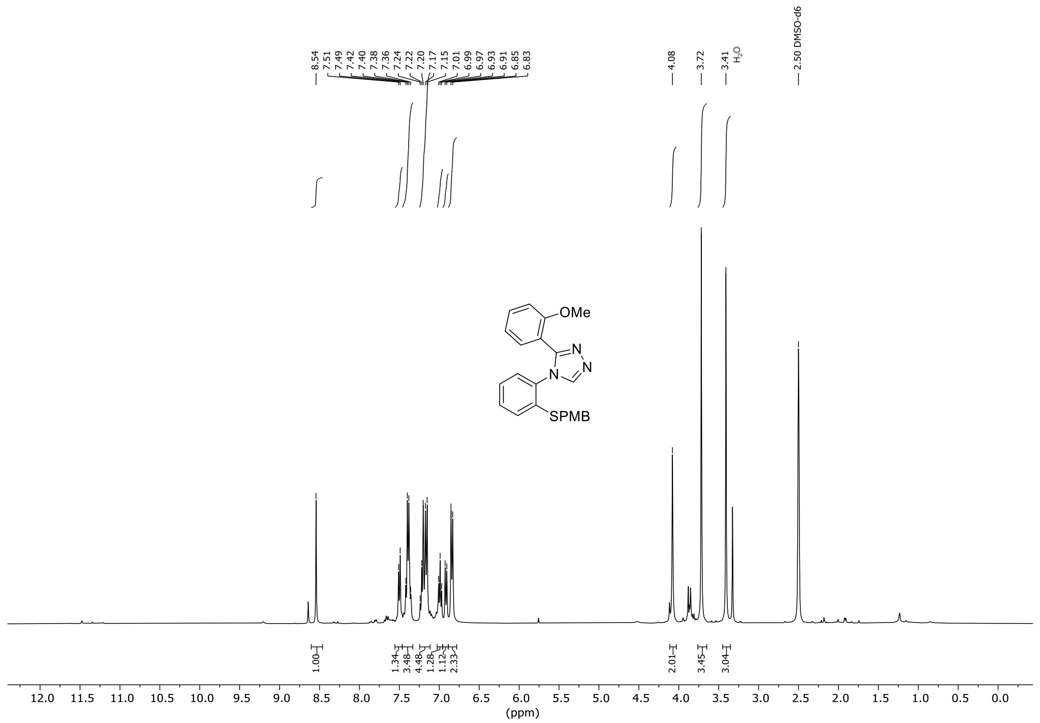

Figure S039: ^{1}H NMR spectrum of 4-(2-((4-methoxybenzyl)thio)phenyl)-3-(2-methoxyphenyl)-4*H*-1,2,4-triazole (**2aj**) (400 MHz, DMSO-d_6, 298 K).

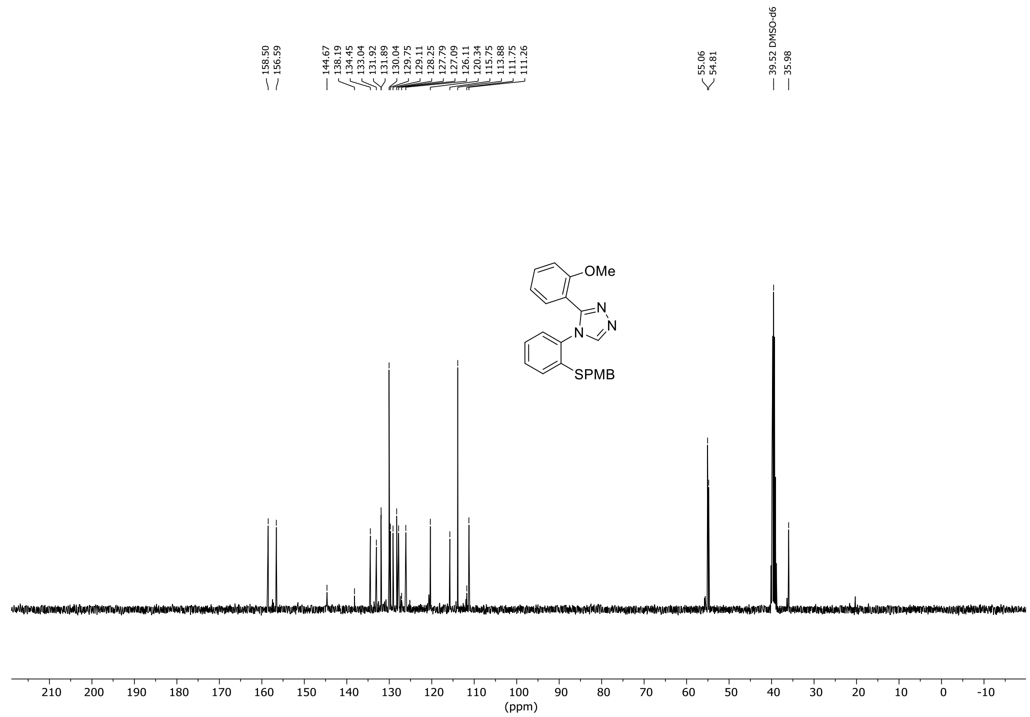

Figure S040 : ^{13}C NMR spectrum 4-(2-((4-methoxybenzyl)thio)phenyl)-3-(2-methoxyphenyl)-4*H*-1,2,4-triazole (**2aj**) (100 MHz, DMSO-d_6, 298 K).

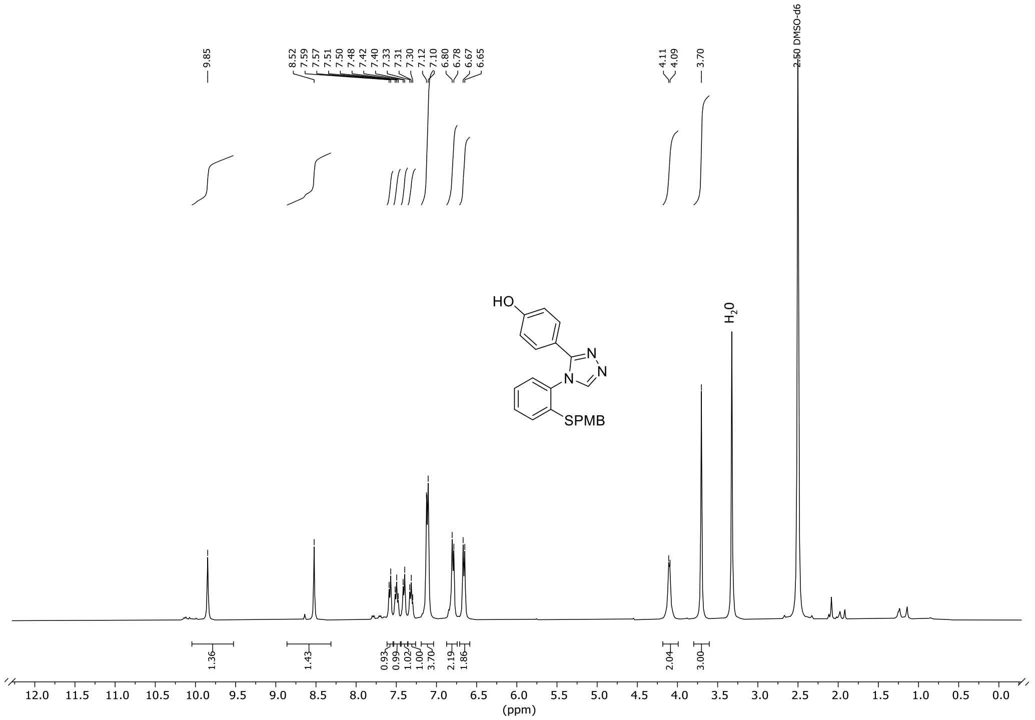

Figure S041: ^{1}H NMR spectrum of4-(4-(2-((4-methoxybenzyl)thio)phenyl)-4*H*-1,2,4-triazol-3-yl)phenol (**2ak**) (400 MHz, DMSO-d_6, 298 K).

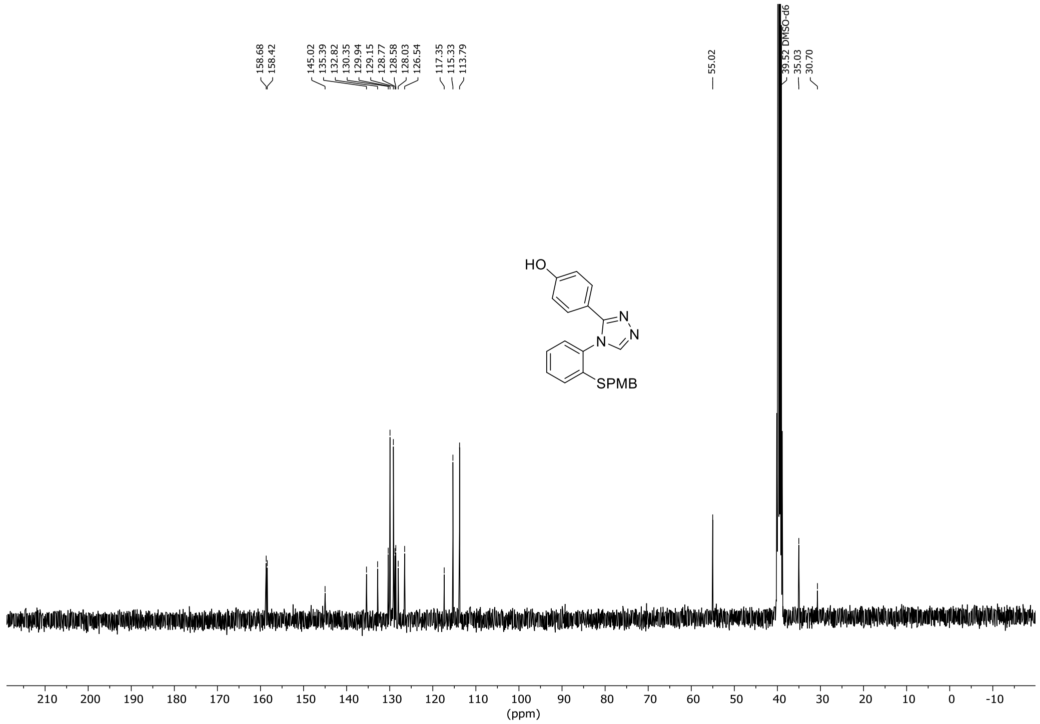

Figure S042: ^{13}C NMR spectrum of -(4-(2-((4-methoxybenzyl)thio)phenyl)-4*H*-1,2,4-triazol-3-yl)phenol (**2ak**) (100 MHz, DMSO-d_6, 298 K).

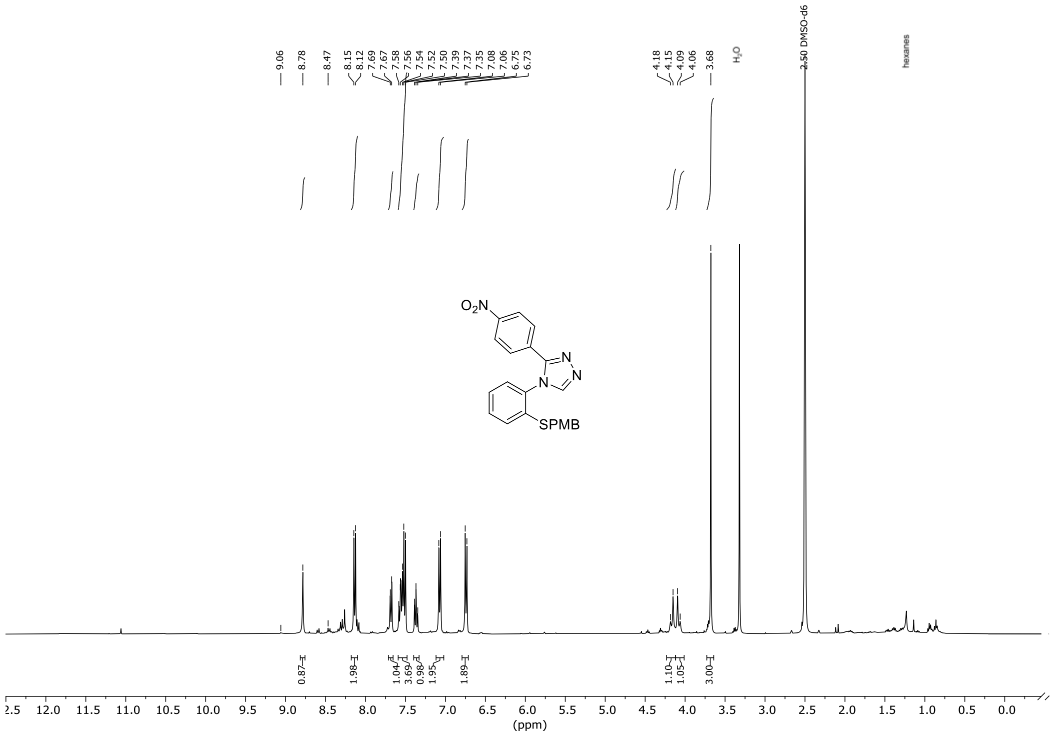

Figure S043: ^{1}H NMR spectrum of 4-(2-((4-methoxybenzyl)thio)phenyl)-3-(4-nitrophenyl)-4*H*-1,2,4-triazole (**2al**) (400 MHz, DMSO-d_6, 298 K).

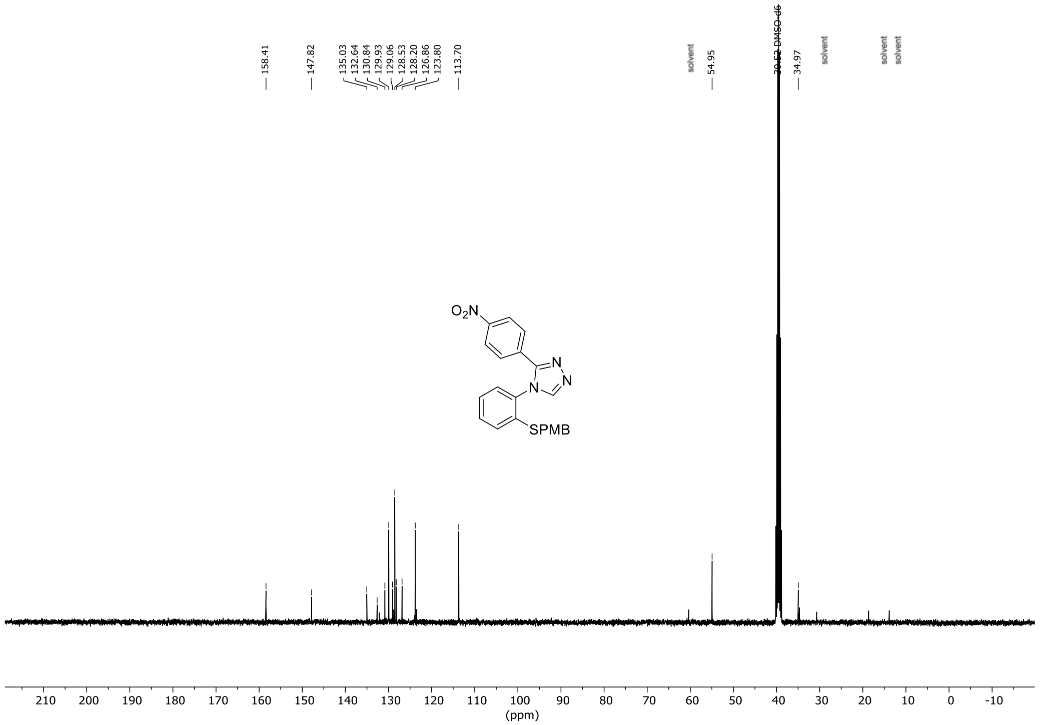

Figure S044: ^{13}C NMR spectrum4-(2-((4-methoxybenzyl)thio)phenyl)-3-(4-nitrophenyl)-4*H*-1,2,4-triazole (**2al**) (100 MHz, DMSO-d_6, 298 K).

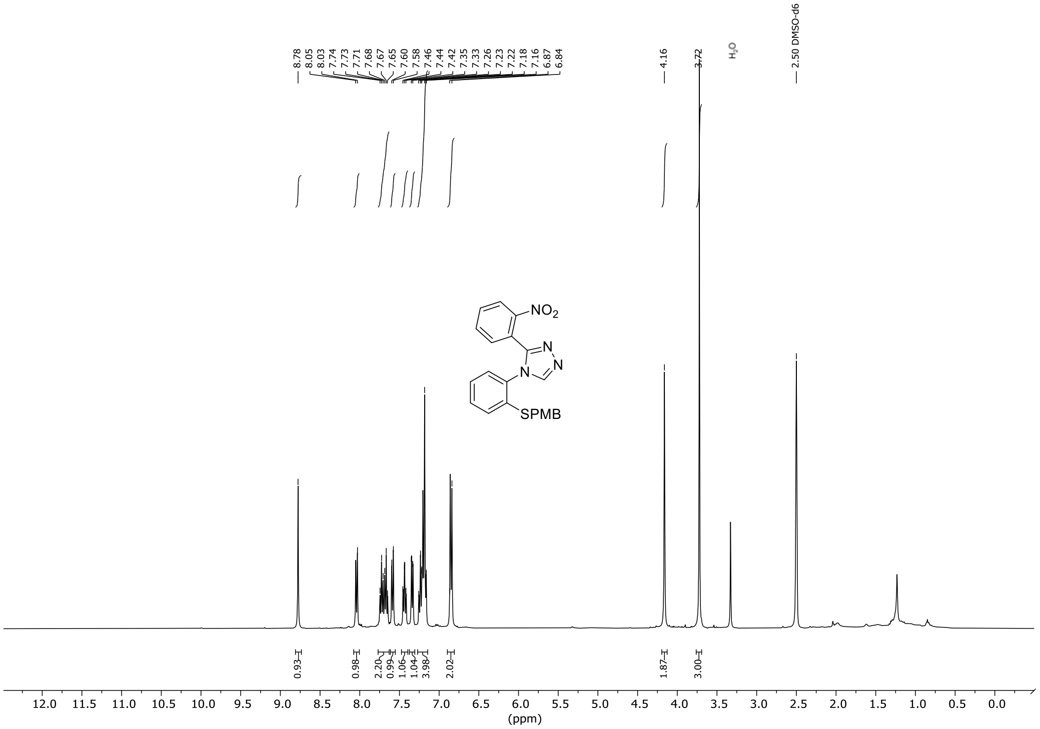

Figure S045: ^{1}H NMR spectrum of 4-(2-((4-methoxybenzyl)thio)phenyl)-3-(2-nitrophenyl)-4*H*-1,2,4-triazole (**2am**) (400 MHz, DMSO-d_6, 298 K).

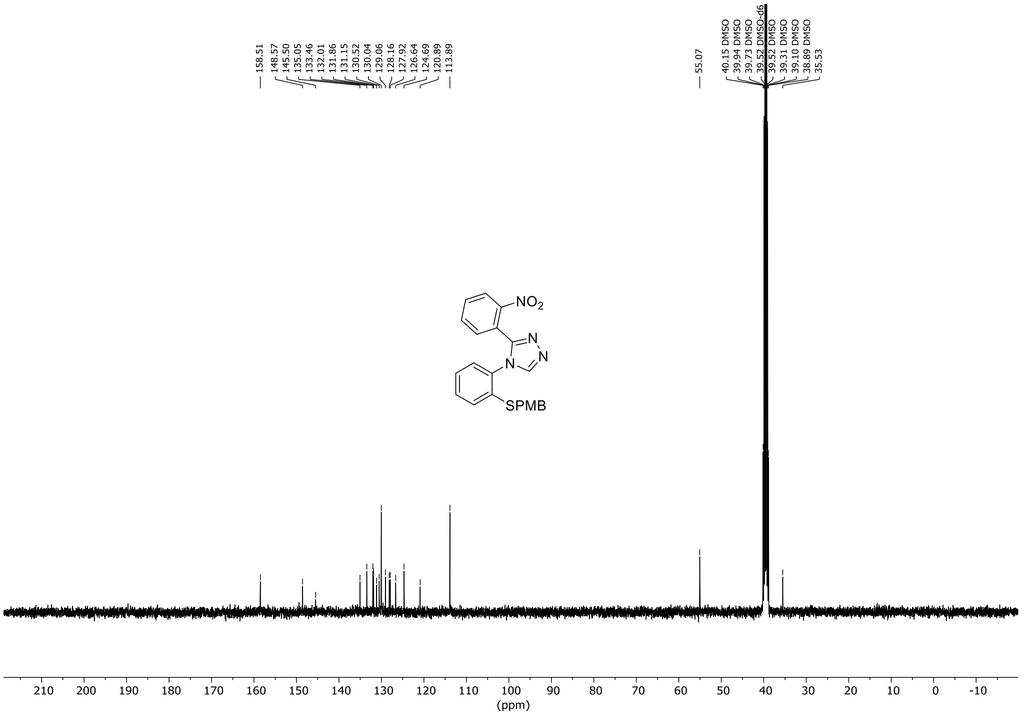

Figure S046: ^{13}C NMR spectrum 4-(2-((4-methoxybenzyl)thio)phenyl)-3-(2-nitrophenyl)-4*H*-1,2,4-triazole (**2am**) (100 MHz, DMSO-d_6, 298 K).

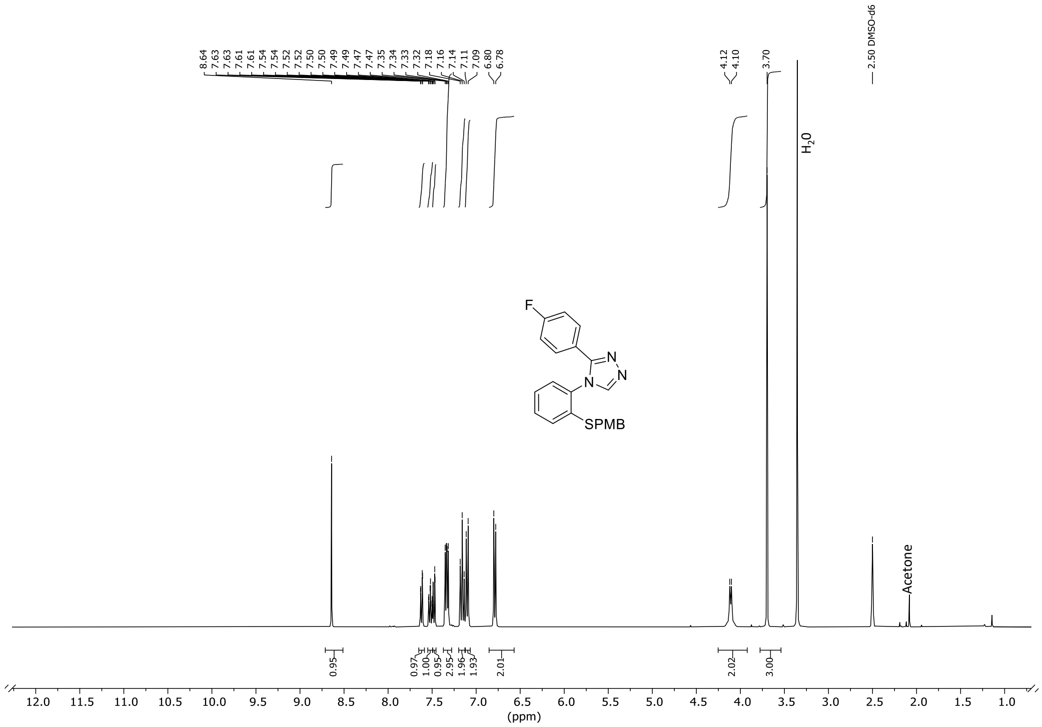

Figure S047: ^{1}H NMR spectrum of 3-(4-fluorophenyl)-4-(2-((4-methoxybenzyl)thio)phenyl)-4*H*-1,2,4-triazole (**2an**) (400 MHz, DMSO-d_6, 298 K).

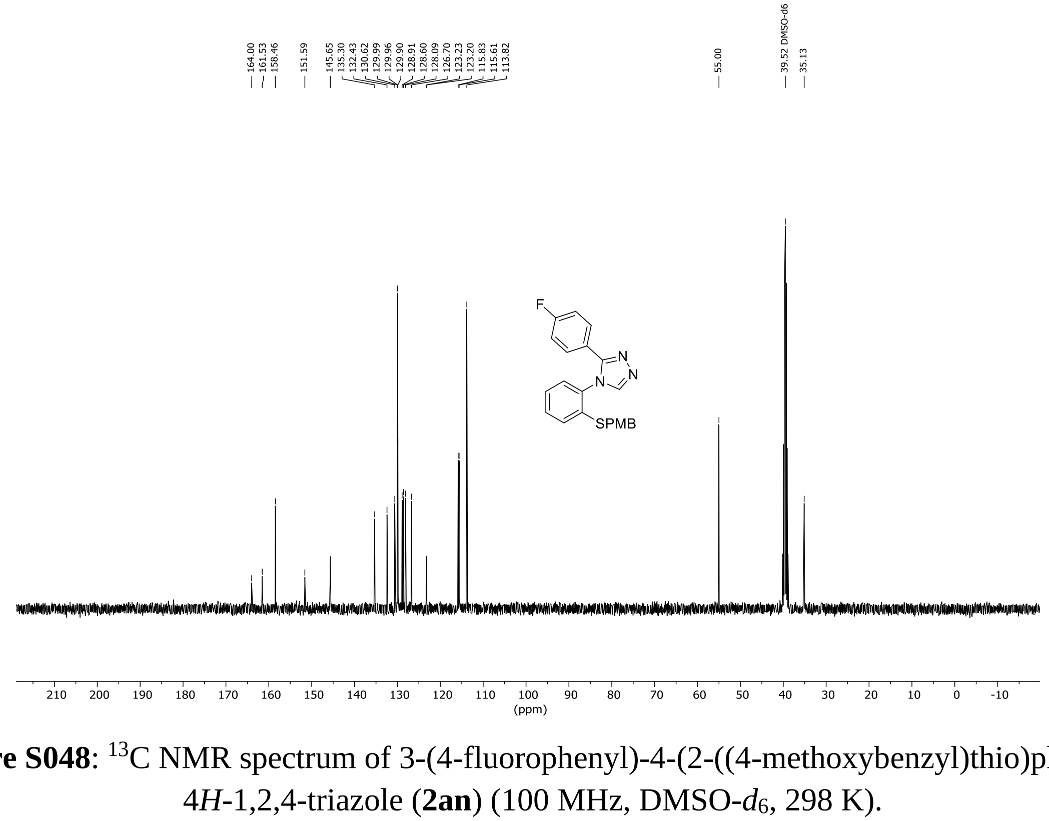

Figure S048: ^{13}C NMR spectrum of 3-(4-fluorophenyl)-4-(2-((4-methoxybenzyl)thio)phenyl)-4*H*-1,2,4-triazole (**2an**) (100 MHz, DMSO-d_6, 298 K).

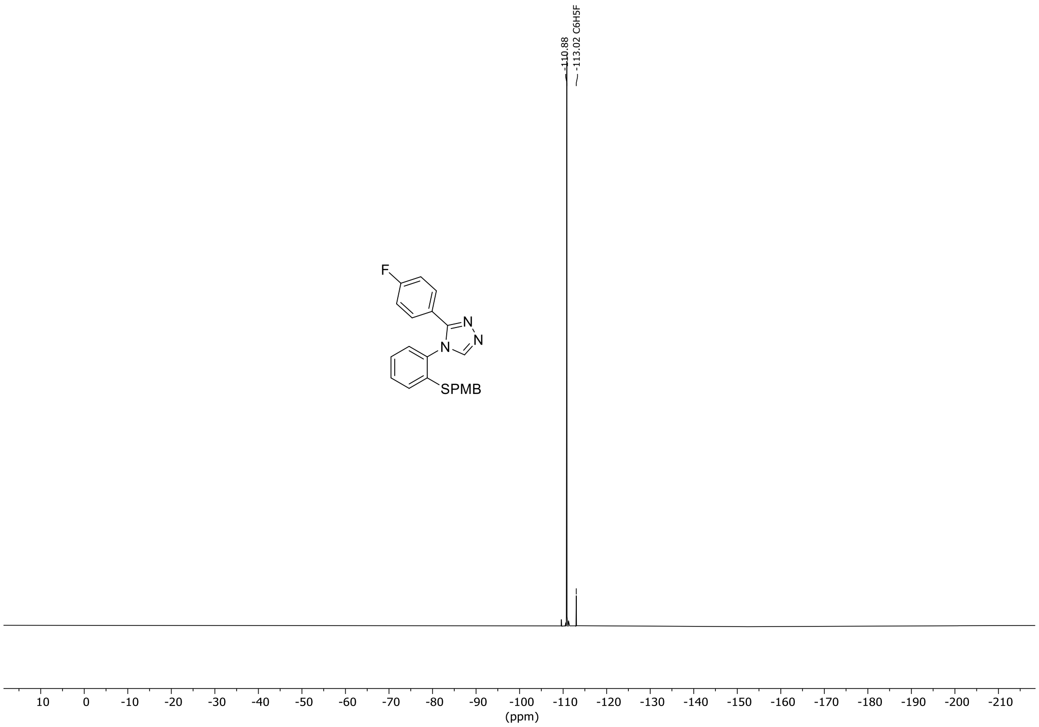

Figure S049: ^{19}F NMR spectrum of 3-(4-fluorophenyl)-4-(2-((4-methoxybenzyl)thio)phenyl)-4*H*-1,2,4-triazole (**2an**) (376 MHz, DMSO-d_6, 298 K).

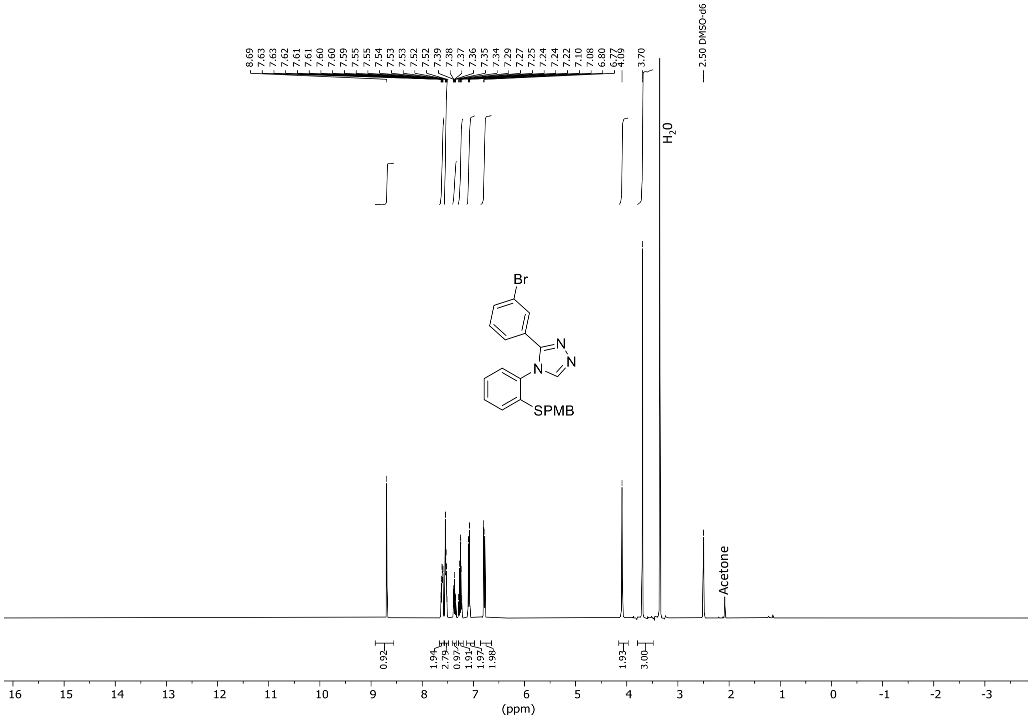

Figure S050: ^{1}H NMR spectrum of 3-(3-bromophenyl)-4-(2-((4-methoxybenzyl)thio)phenyl)-4*H*-1,2,4-triazole (**2ao**) (400 MHz, DMSO-d_6, 298 K).

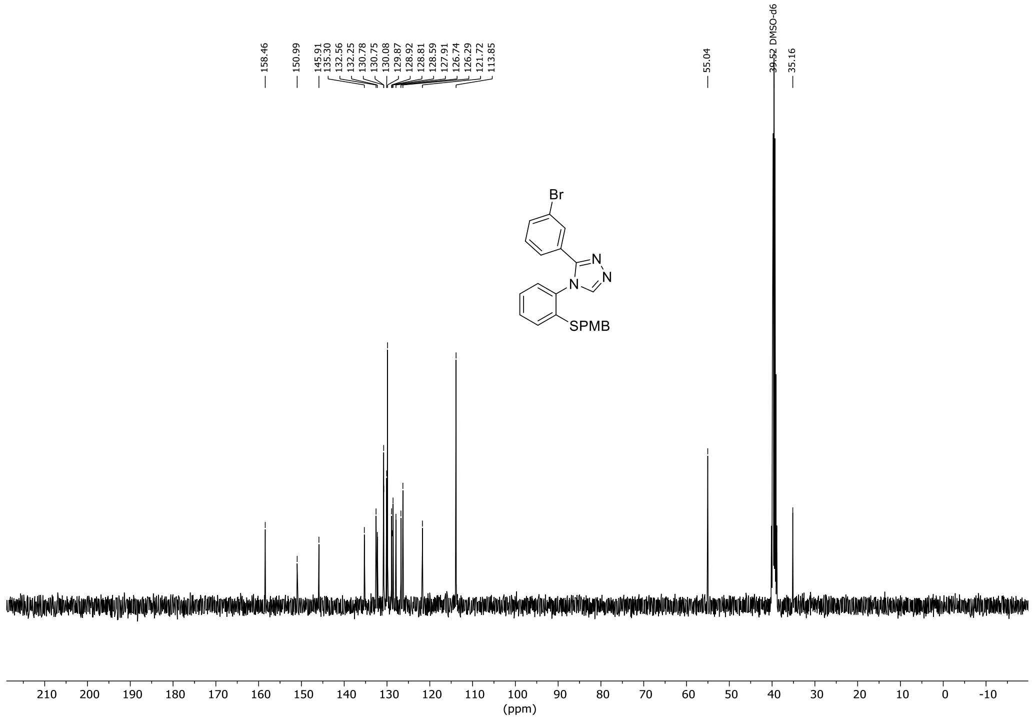

Figure S051: ^{13}C NMR spectrum of 3-(3-bromophenyl)-4-(2-((4-methoxybenzyl)thio)phenyl)-4*H*-1,2,4-triazole (**2ao**) (100 MHz, DMSO-d_6, 298 K).

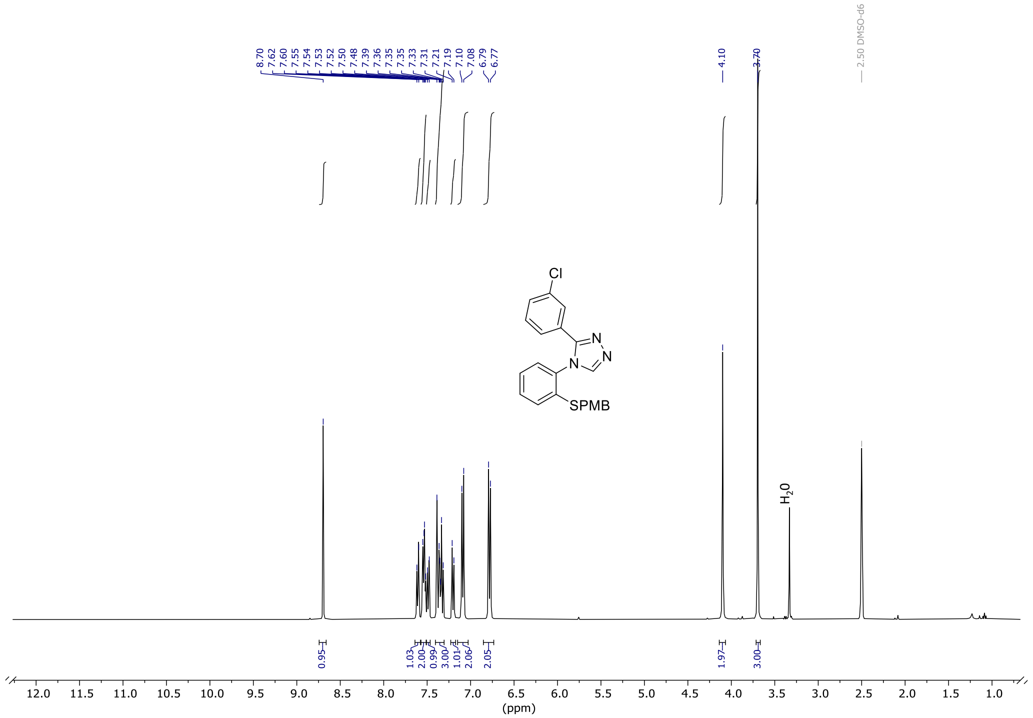

Figure S052: ^{1}H NMR spectrum of 3-(3-chlorophenyl)-4-(2-((4-methoxybenzyl)thio)phenyl)-4*H*-1,2,4-triazole (**2ap**) (400 MHz, DMSO-d_6, 298 K).

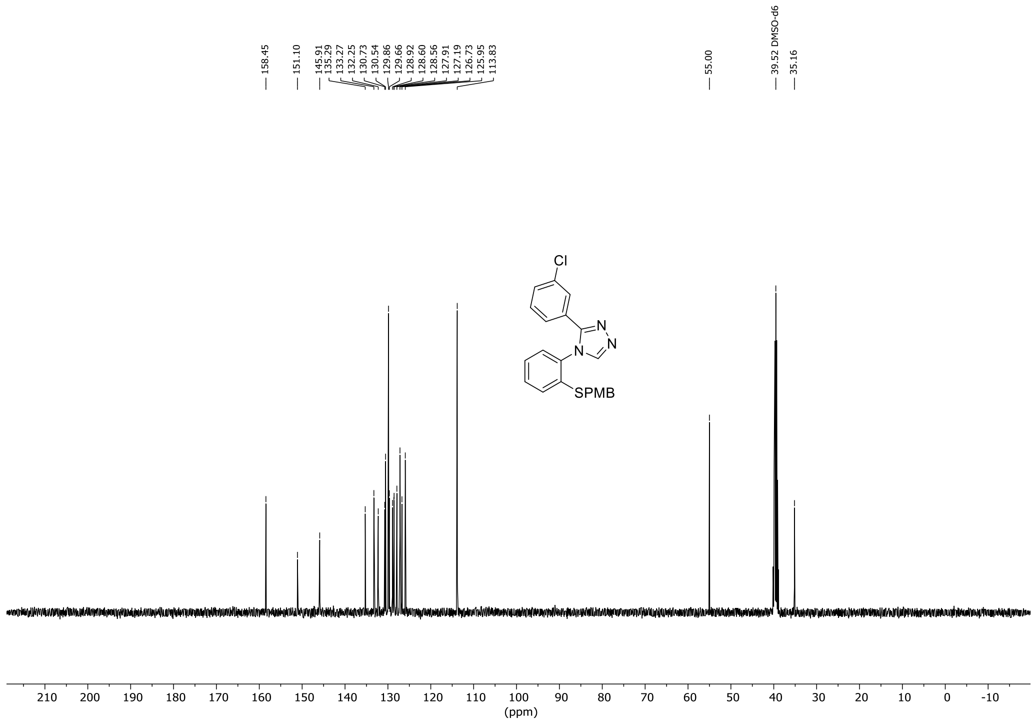

Figure S053: ^{13}C NMR spectrum 3-(3-chlorophenyl)-4-(2-((4-methoxybenzyl)thio)phenyl)-4*H*-1,2,4-triazole (**2ap**) (100 MHz, DMSO-d_6, 298 K).

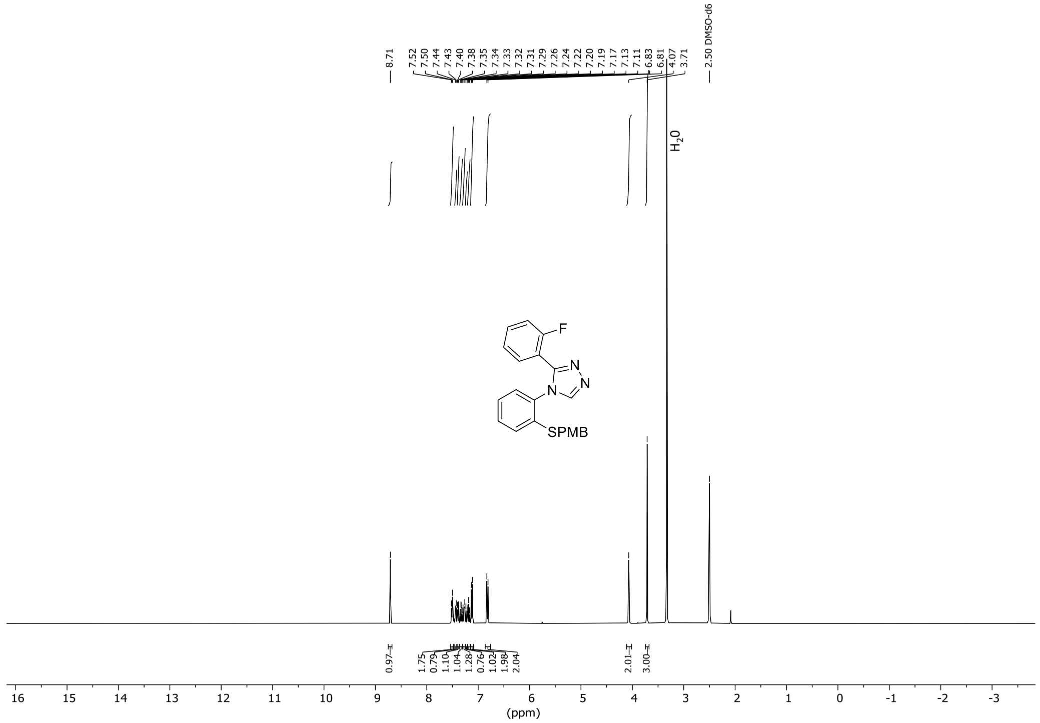

Figure S054: ^{1}H NMR spectrum of 3-(2-fluorophenyl)-4-(2-((4-methoxybenzyl)thio)phenyl)-4*H*-1,2,4-triazole (**2aq**) (400 MHz, DMSO-d_6, 298 K).

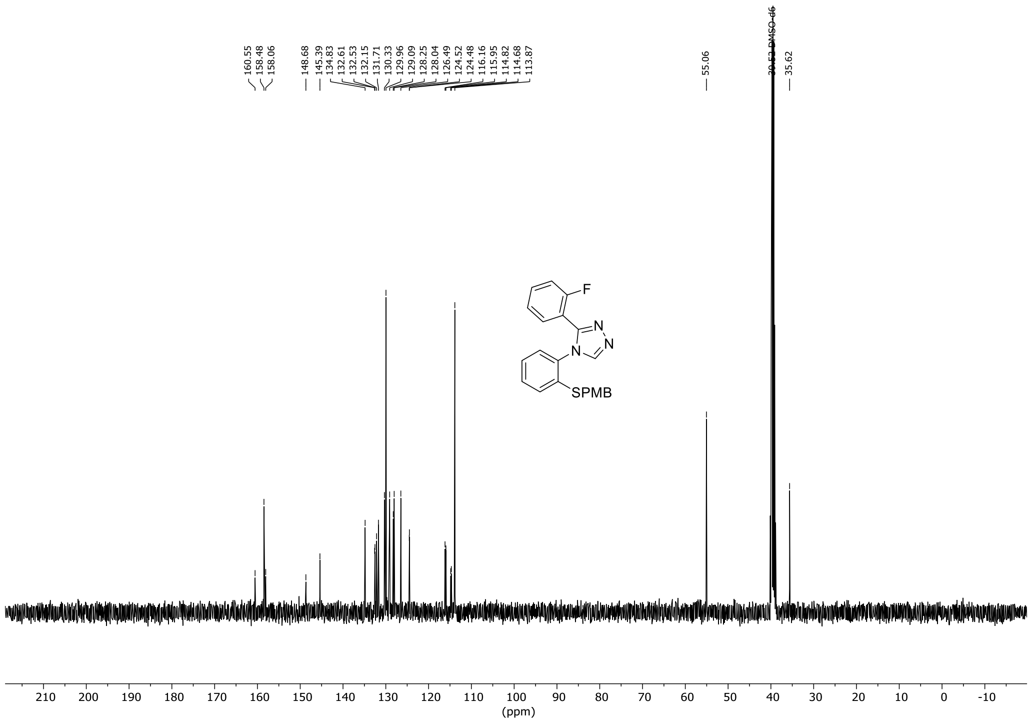

Figure S055: ^{13}C NMR spectrum of of 3-(2-fluorophenyl)-4-(2-((4-methoxybenzyl)thio)phenyl)-4*H*-1,2,4-triazole (**2aq**) (100 MHz, DMSO-d_6, 298 K).

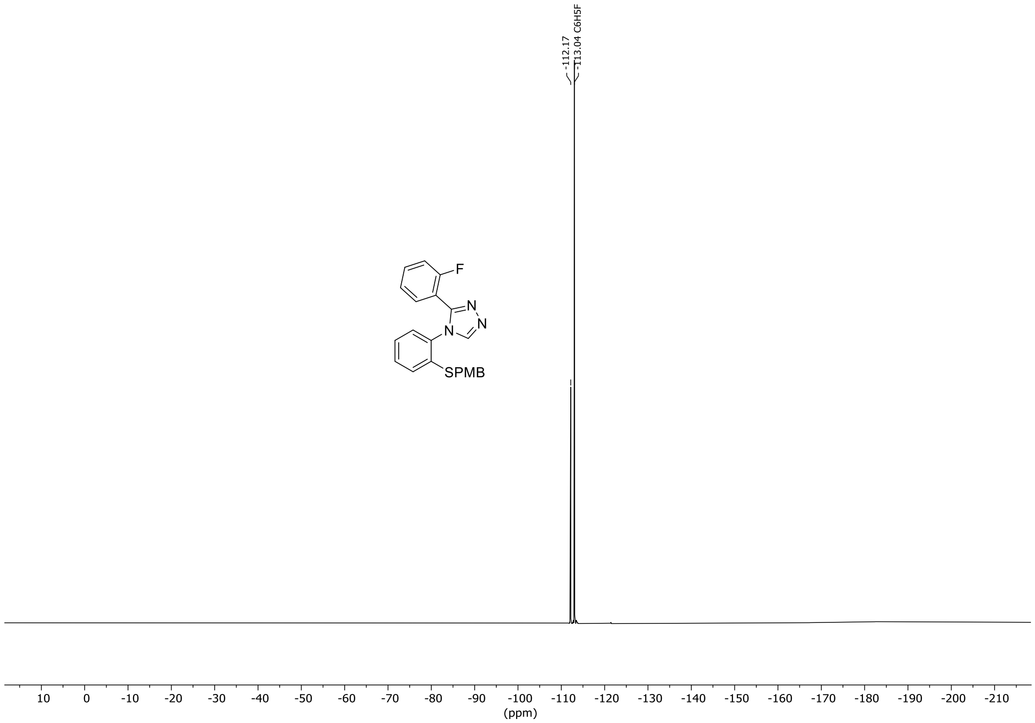

Figure S056: ^{19}F NMR spectrum of 3-(2-fluorophenyl)-4-(2-((4-methoxybenzyl)thio)phenyl)-4*H*-1,2,4-triazole (**2aq**) (376 MHz, DMSO-d_6, 298 K.

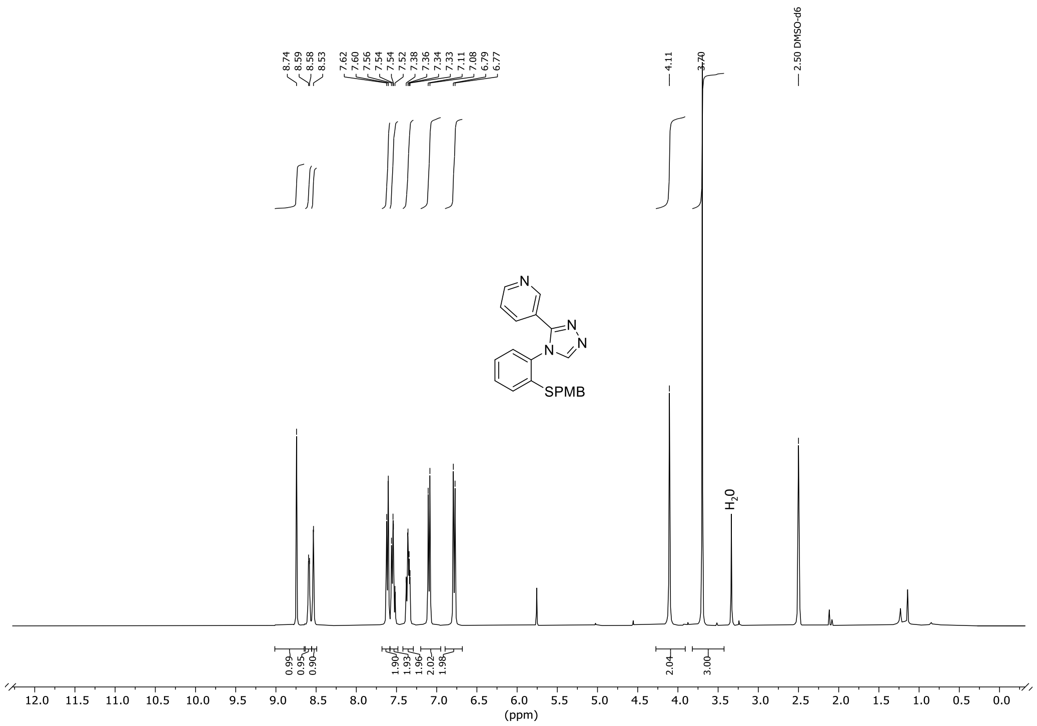

Figure S057: ^{1}H NMR spectrum of 3-(4-(2-((4-methoxybenzyl)thio)phenyl)-4*H*-1,2,4-triazol-3-yl)pyridine (**2ar**) (400 MHz, DMSO-d_6, 298 K).

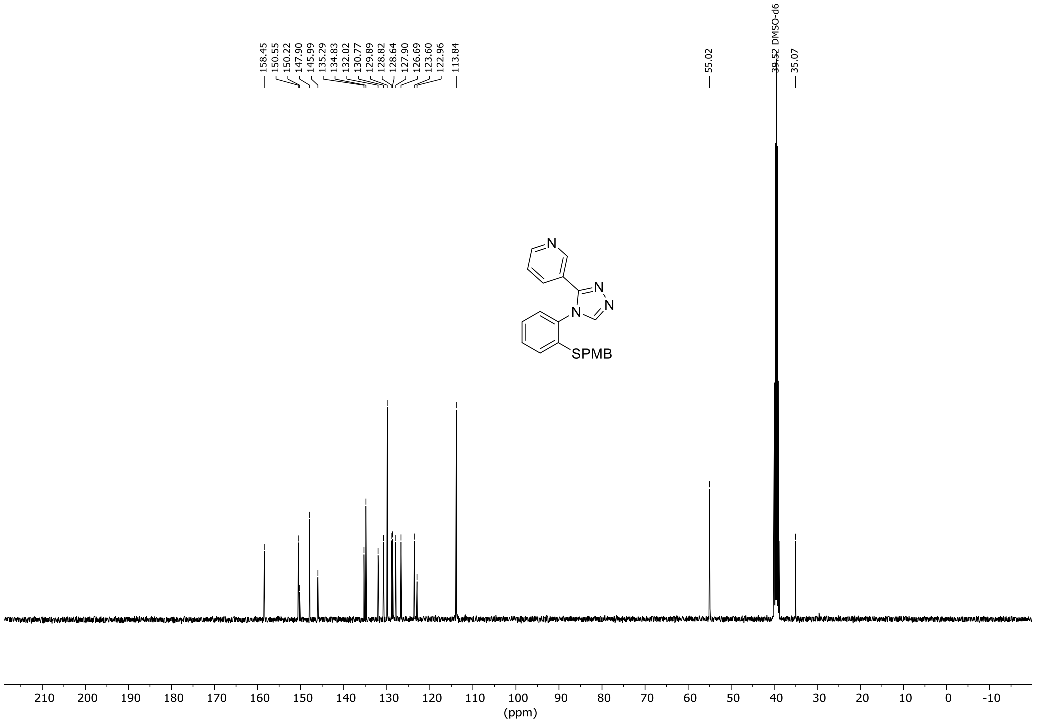

Figure S058: ^{13}C NMR spectrum of 3-(4-(2-((4-methoxybenzyl)thio)phenyl)-4*H*-1,2,4-triazol-3-yl)pyridine (**2ar**) (100 MHz, DMSO-d_6, 298 K).

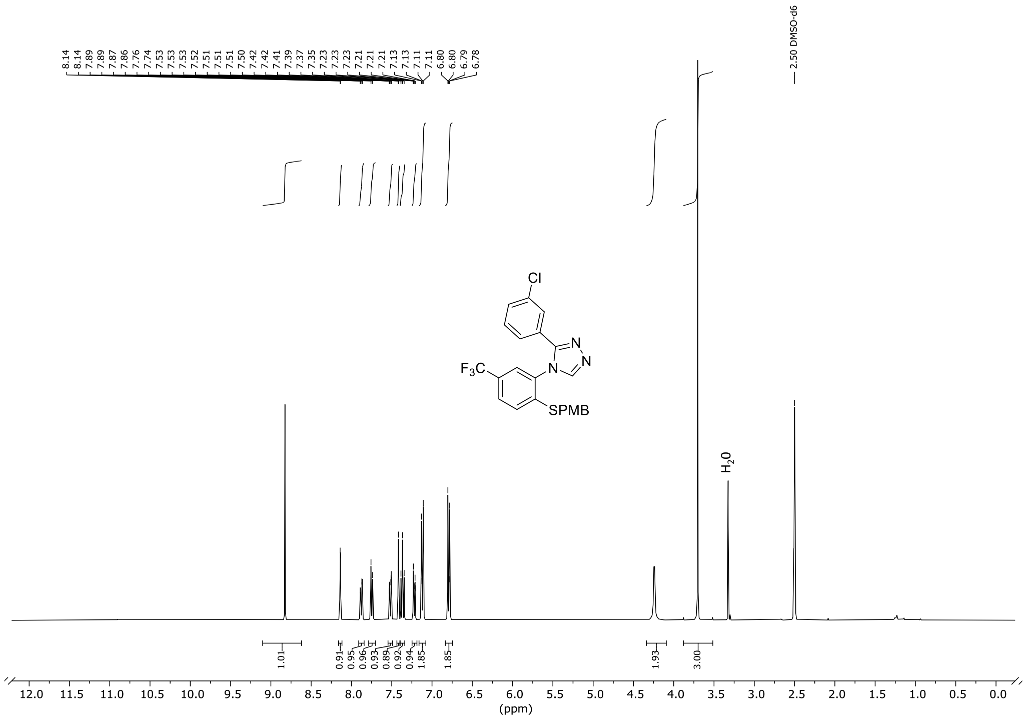

Figure S059: ^{1}H NMR spectrum of 3-(3-chlorophenyl)-4-(2-((4-methoxybenzyl)thio)-5-(trifluoromethyl)phenyl)-4*H*-1,2,4-triazole (**2bp**) (400 MHz, DMSO-d_6, 298 K).

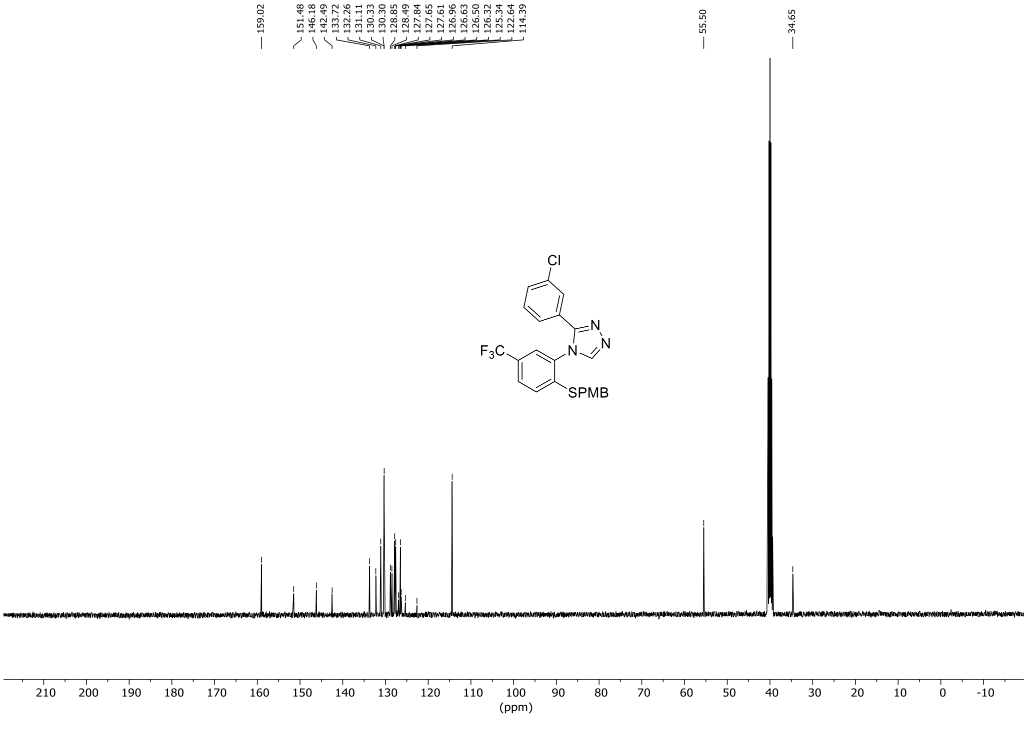

Figure S060: ^{13}C NMR spectrum 3-(3-chlorophenyl)-4-(2-((4-methoxybenzyl)thio)-5-(trifluoromethyl)phenyl)-4*H*-1,2,4-triazole (**2bp**) (100 MHz, DMSO-d_6, 298 K).

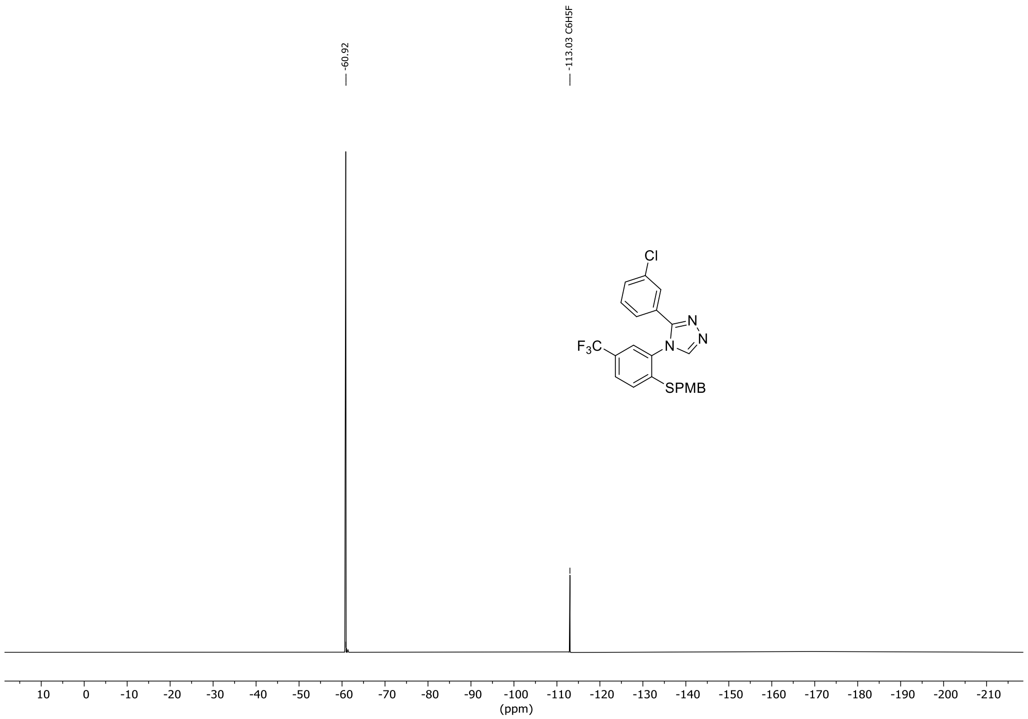

Figure S061: ^{19}F NMR spectrum of 3-(3-chlorophenyl)-4-(2-((4-methoxybenzyl)thio)-5-(trifluoromethyl)phenyl)-4*H*-1,2,4-triazole (**2bp**) (376 MHz, DMSO-d_6, 298 K).

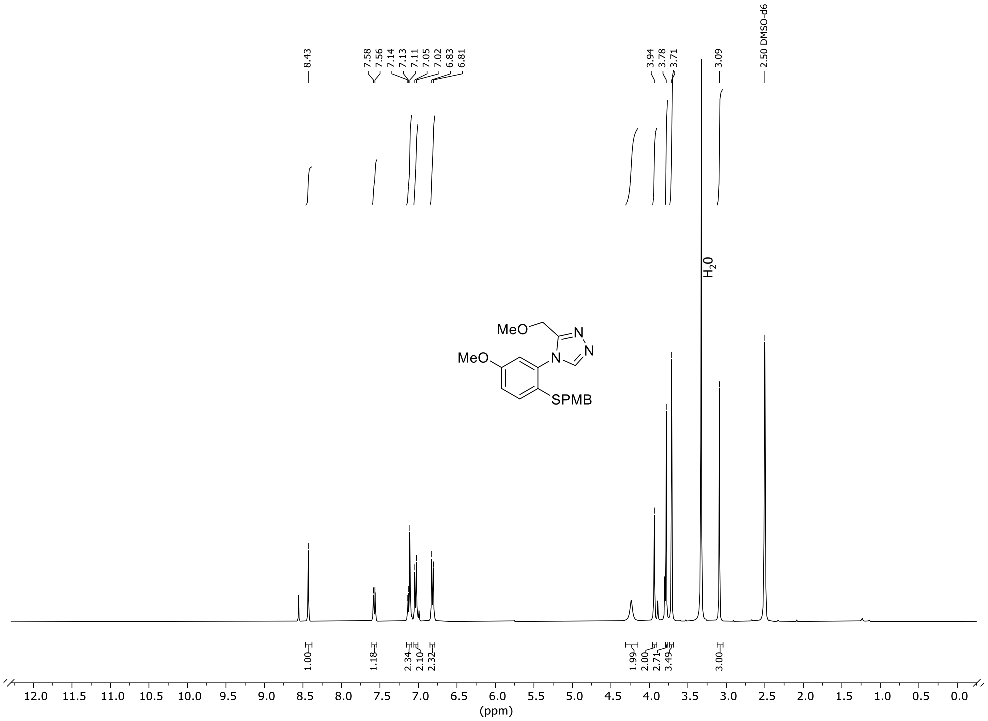

Figure S062: ^{1}H NMR spectrum of 4-(5-methoxy-2-((4-methoxybenzyl)thio)phenyl)-3-(methoxymethyl)-4*H*-1,2,4-triazole (**2cd**) (400 MHz, DMSO-d_6, 298 K).

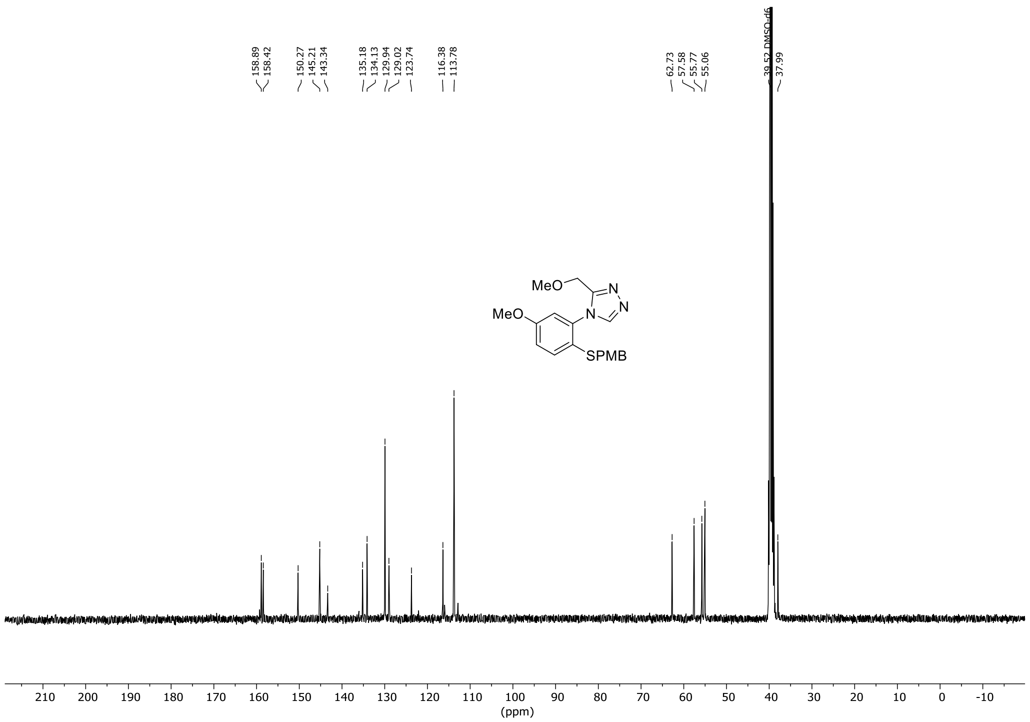

Figure S063: ^{13}C NMR spectrum of 4-(5-methoxy-2-((4-methoxybenzyl)thio)phenyl)-3-(methoxymethyl)-4*H*-1,2,4-triazole (**2cd**) (100 MHz, DMSO-d_6, 298 K).

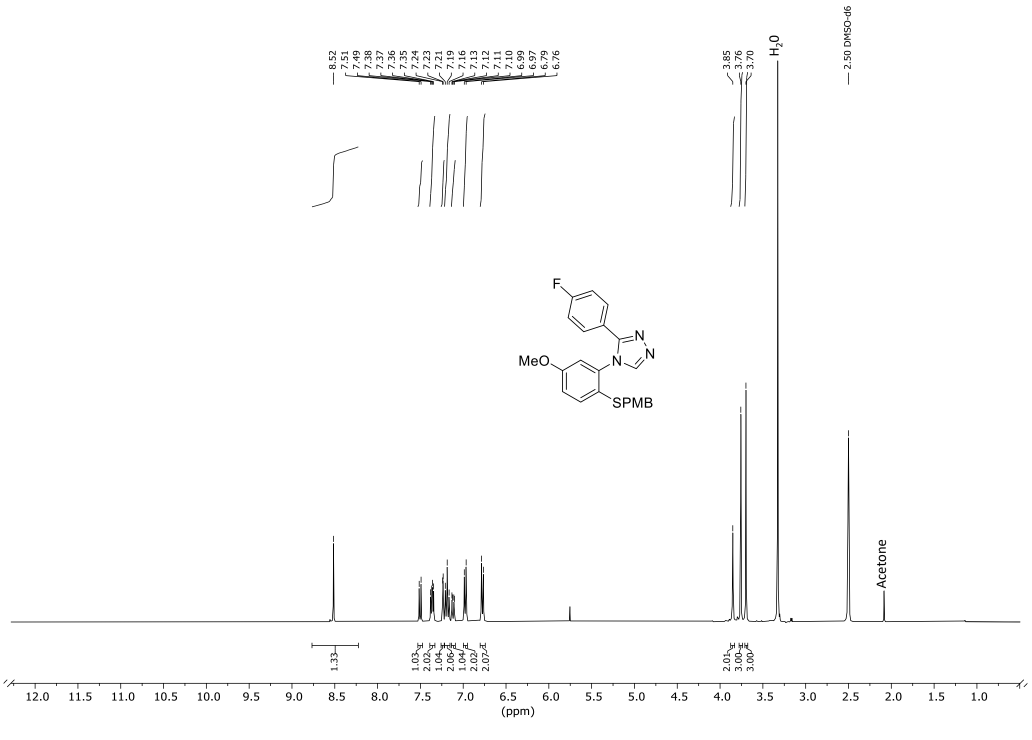

Figure S064: ^{1}H NMR spectrum 3-(4-fluorophenyl)-4-(5-methoxy-2-((4-methoxybenzyl)thio)phenyl)-4*H*-1,2,4-triazole. (**2cn**) (400 MHz, DMSO-d_6, 298 K).

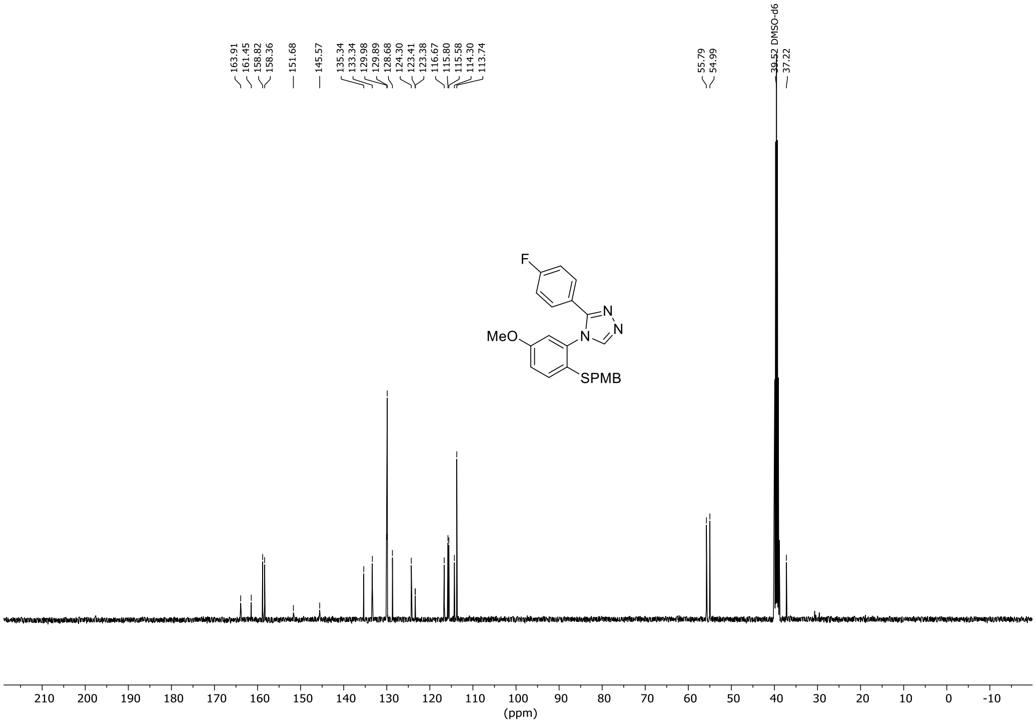

Figure S065: ^{13}C NMR spectrum of 3-(4-fluorophenyl)-4-(5-methoxy-2-((4-methoxybenzyl)thio)phenyl)-4*H*-1,2,4-triazole (**2cn**) (100 MHz, DMSO-d_6, 298 K).

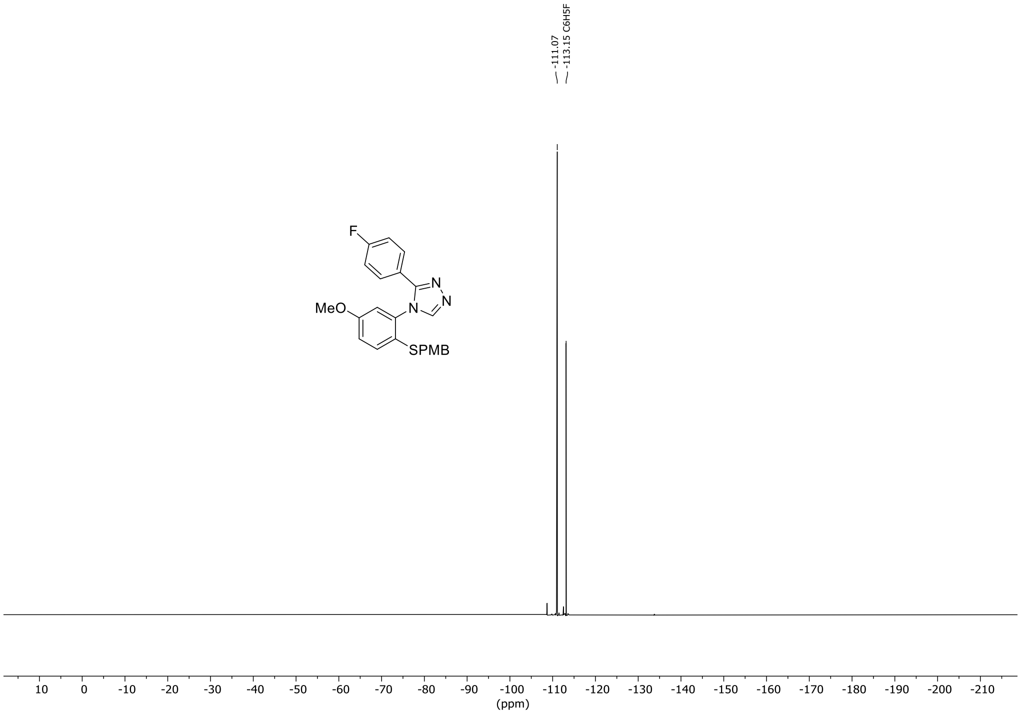

Figure S066: ^{19}F NMR spectrum of 3-(4-fluorophenyl)-4-(5-methoxy-2-((4-methoxybenzyl)thio)phenyl)-4*H*-1,2,4-triazole (**2cn**) (376 MHz, DMSO-d_6, 298 K).

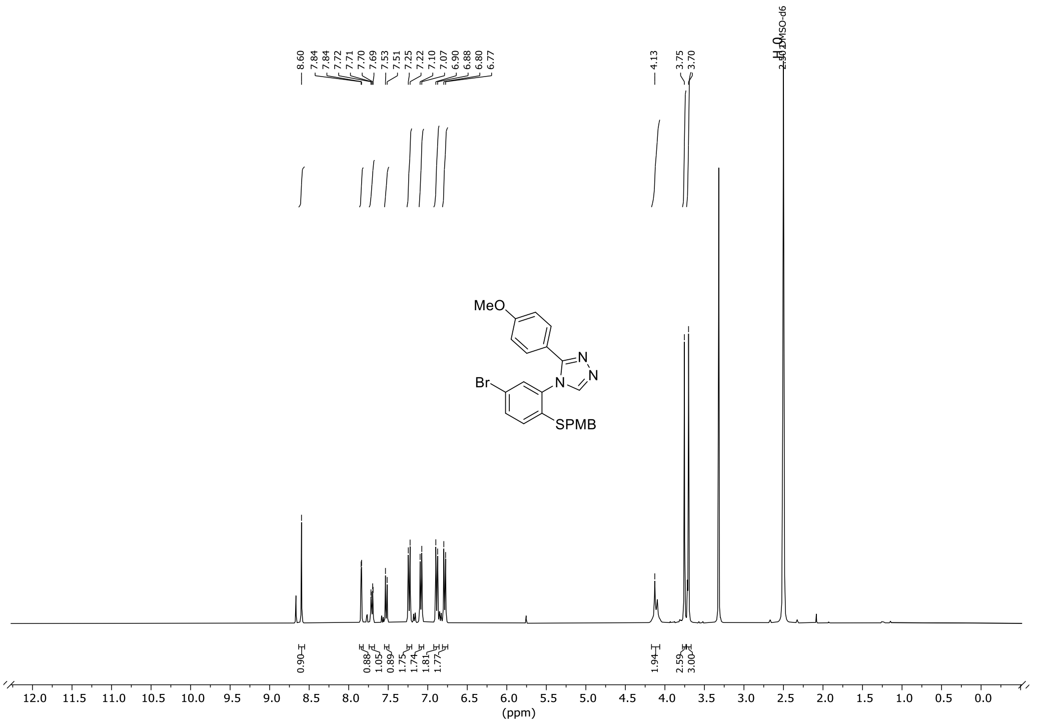

Figure S067: ^{1}H NMR spectrum of 4-(5-bromo-2-((4-methoxybenzyl)thio)phenyl)-3-(4-methoxyphenyl)-4*H*-1,2,4-triazole (**2di**) (400 MHz, DMSO-d_6, 298 K).

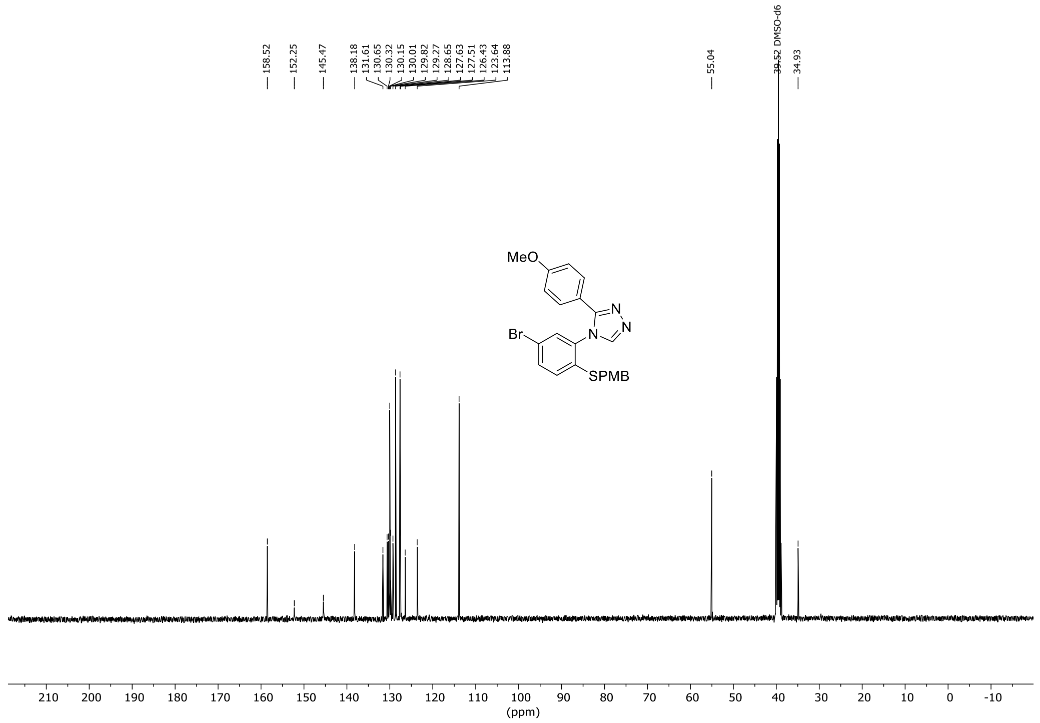

Figure S068: ^{13}C NMR spectrum 4-(5-bromo-2-((4-methoxybenzyl)thio)phenyl)-3-(4-methoxyphenyl)-4*H*-1,2,4-triazole (**2di**) (100 MHz, DMSO-d_6, 298 K).

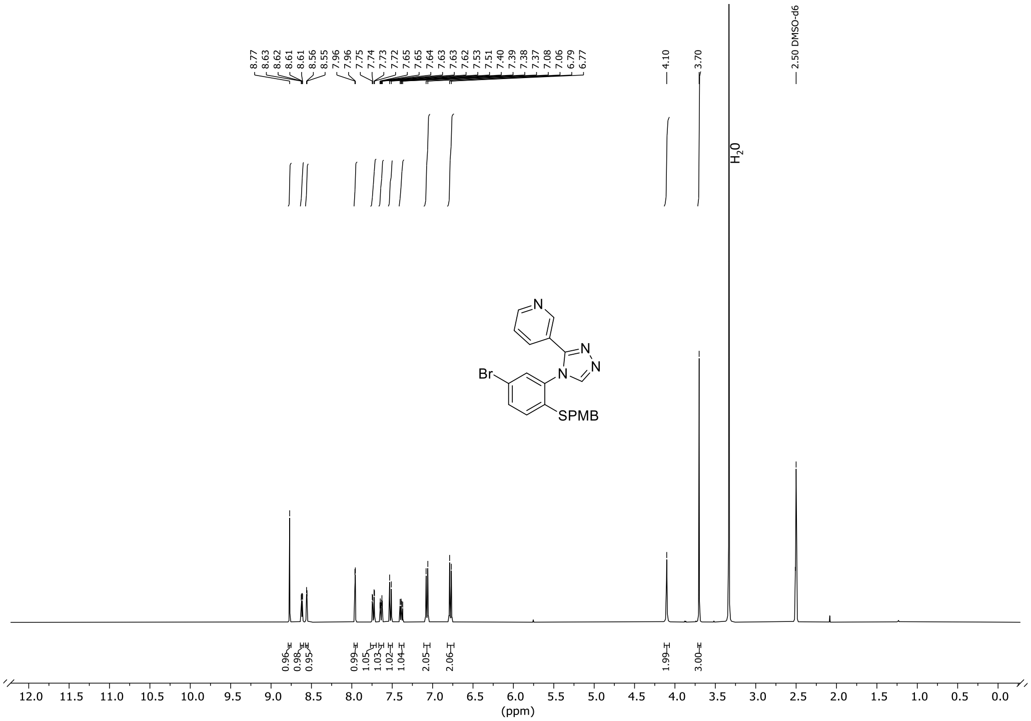

Figure S069: ^{1}H NMR spectrum of 3-(4-(5-bromo-2-((4-methoxybenzyl)thio)phenyl)-4*H*-1,2,4-triazol-3-yl)pyridine (**2dr**) (400 MHz, DMSO-d_6, 298 K).

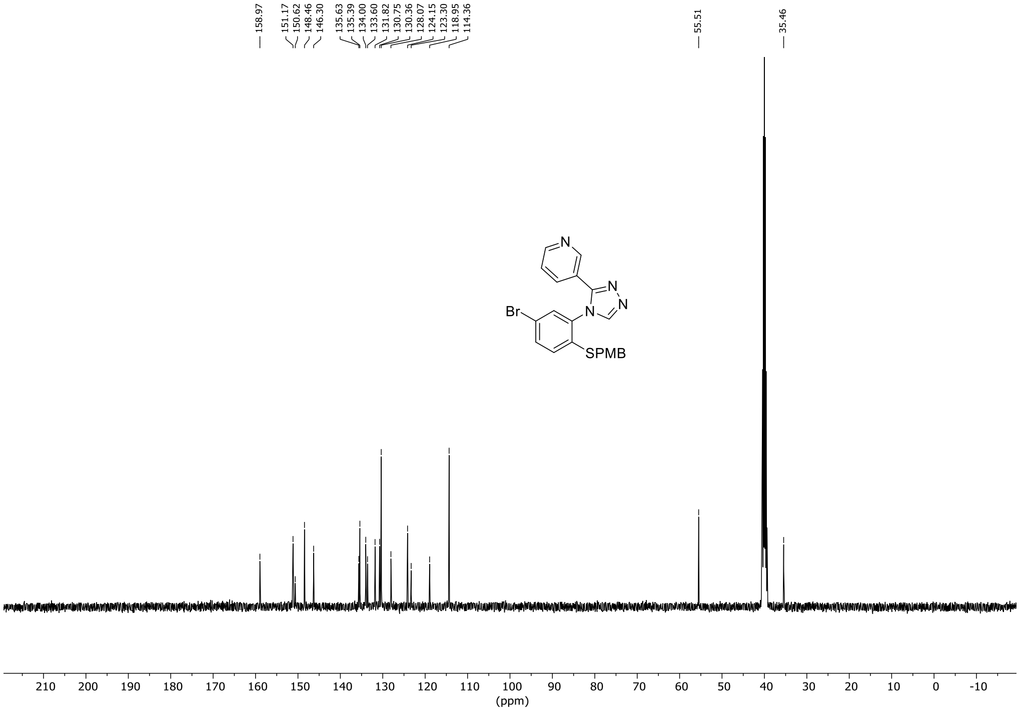

Figure S070: ^{13}C NMR spectrum of 3-(4-(5-bromo-2-((4-methoxybenzyl)thio)phenyl)-4*H*-1,2,4-triazol-3-yl)pyridine (**2dr**) (100 MHz, DMSO-d_6, 298 K).

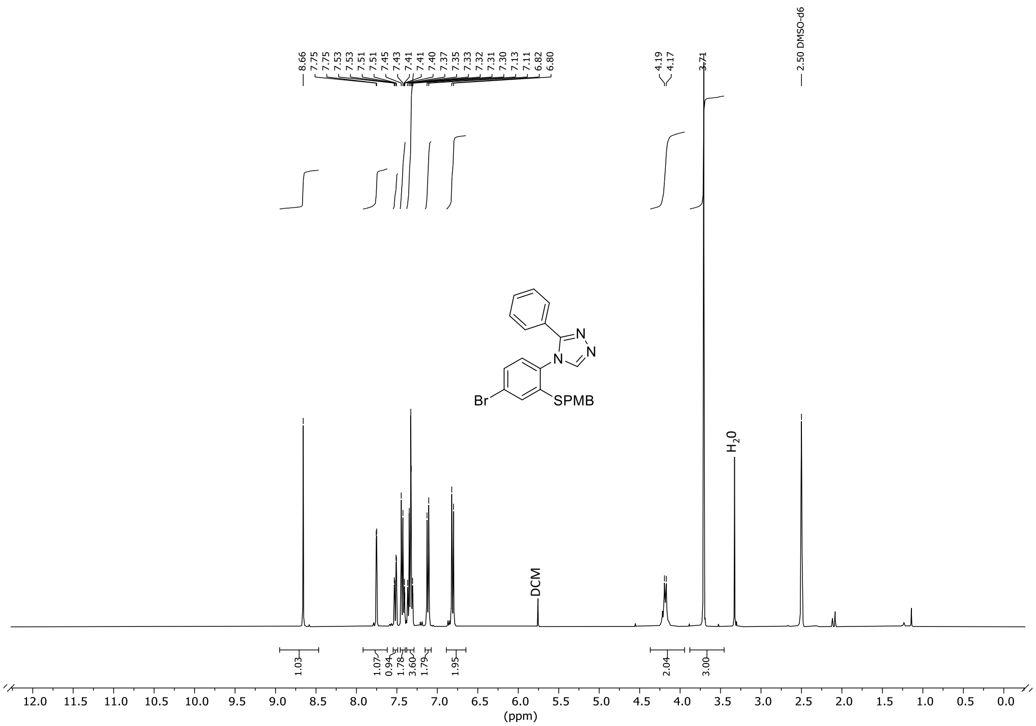

Figure S071: ^{1}H NMR spectrum of 4-(4-bromo-2-((4-methoxybenzyl)thio)phenyl)-3-phenyl-4*H*-1,2,4-triazole (**2ee**) (400 MHz, DMSO-d_6, 298 K).

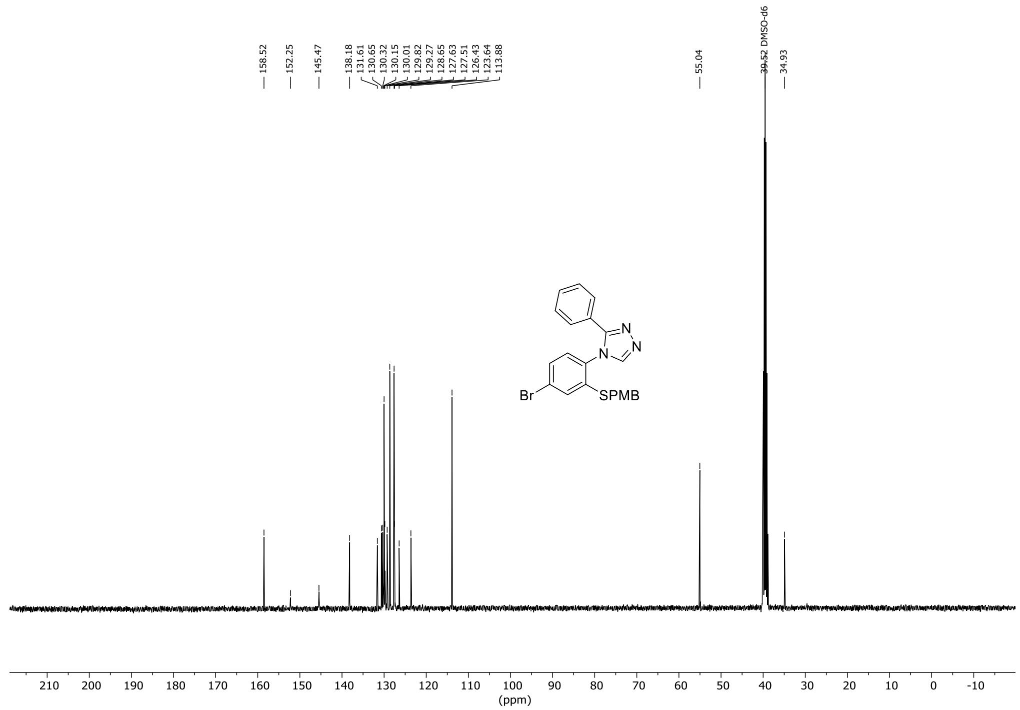

Figure S072: ^{13}C NMR spectrum of 4-(4-bromo-2-((4-methoxybenzyl)thio)phenyl)-3-phenyl-4*H*-1,2,4-triazole (**2ee**) (100 MHz, DMSO-d_6, 298 K).

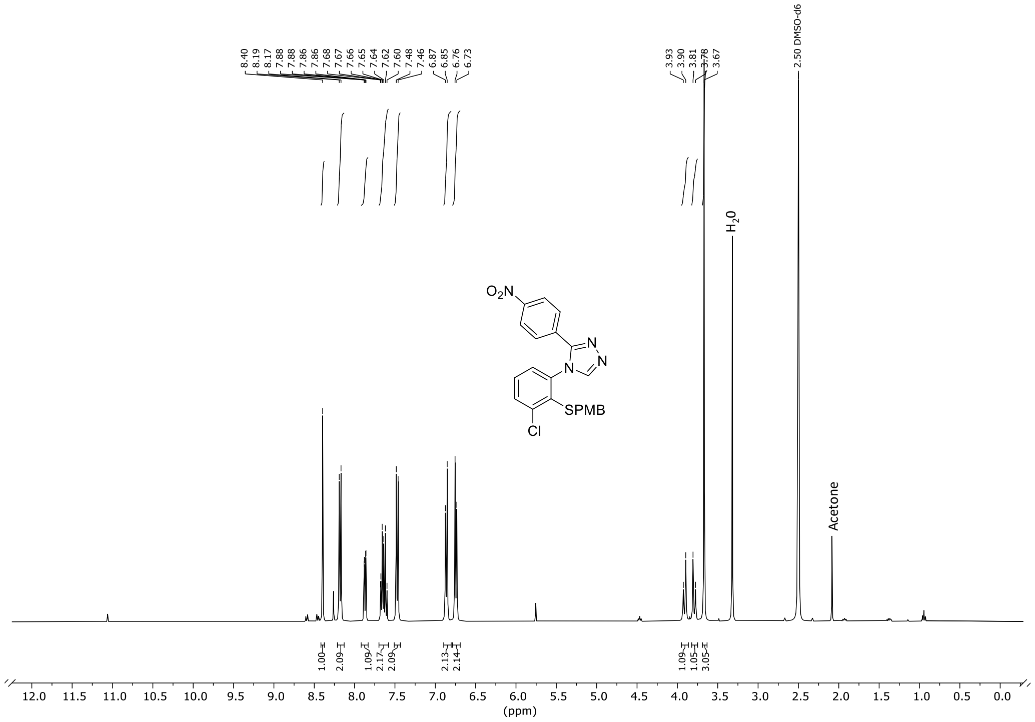

Figure S073: ^{1}H NMR spectrum of 4-(3-chloro-2-((4-methoxybenzyl)thio)phenyl)-3-(4-nitrophenyl)-4*H*-1,2,4-triazole (**2fl**) (400 MHz, DMSO-d_6, 298 K).

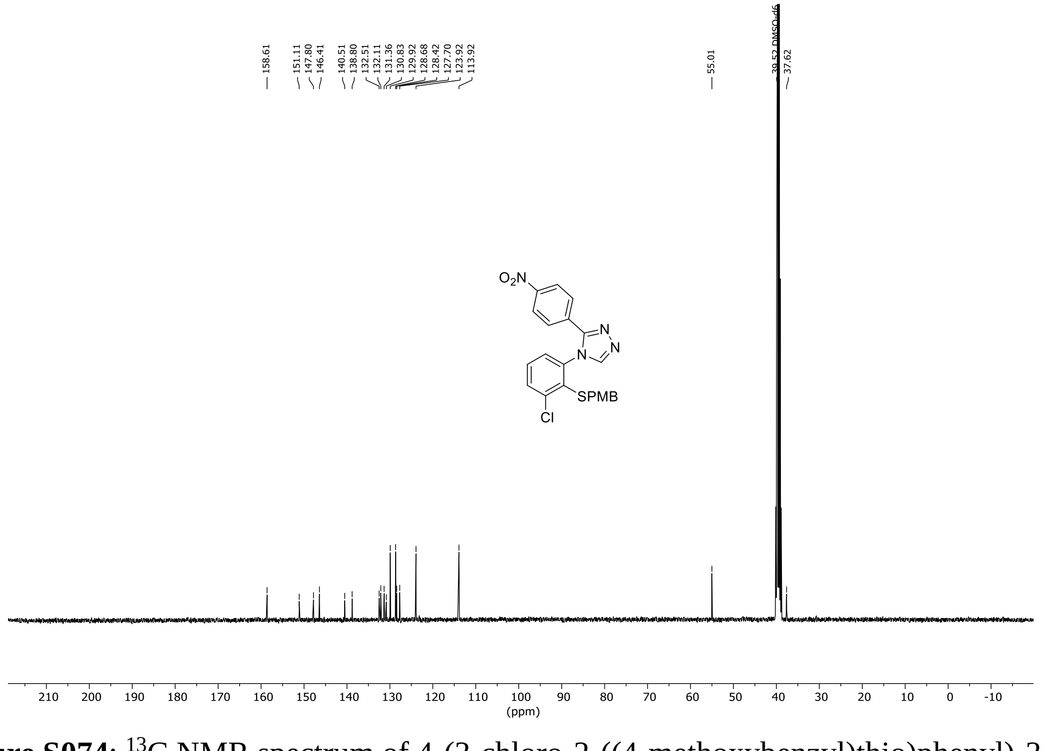

Figure S074: ^{13}C NMR spectrum of 4-(3-chloro-2-((4-methoxybenzyl)thio)phenyl)-3-(4-nitrophenyl)-4*H*-1,2,4-triazole (**2fl**) (100 MHz, DMSO-d_6, 298 K).

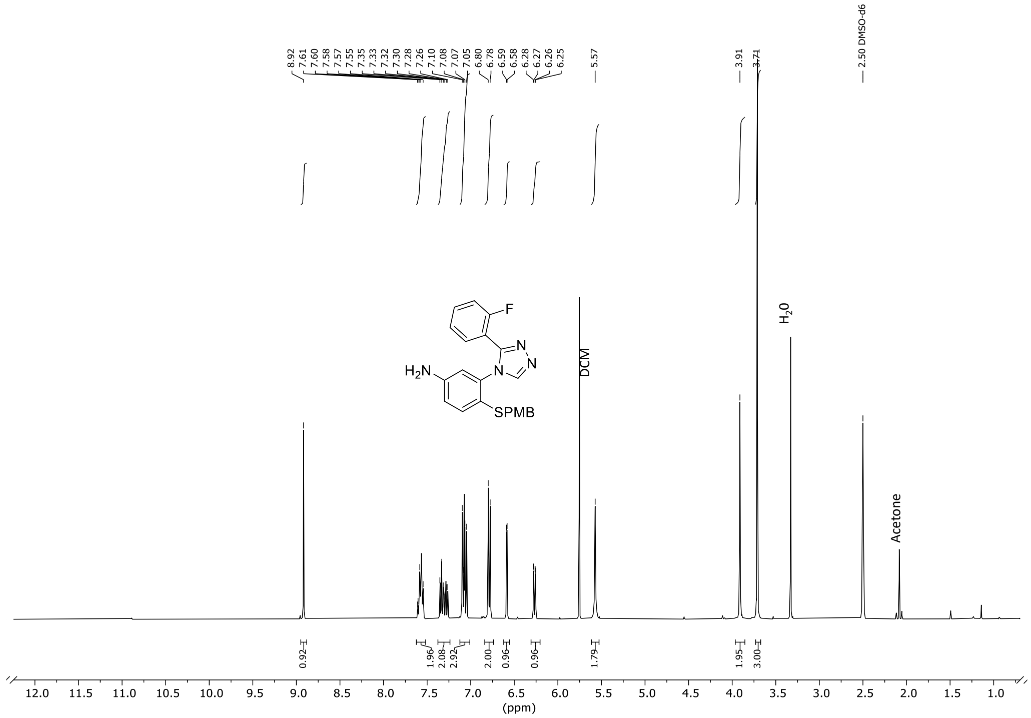

Figure S075: ^{1}H NMR spectrum of 3-(3-(2-fluorophenyl)-4*H*-1,2,4-triazol-4-yl)-4-((4-methoxybenzyl)thio)aniline (**2gq**) (400 MHz, DMSO-d_6, 298 K).

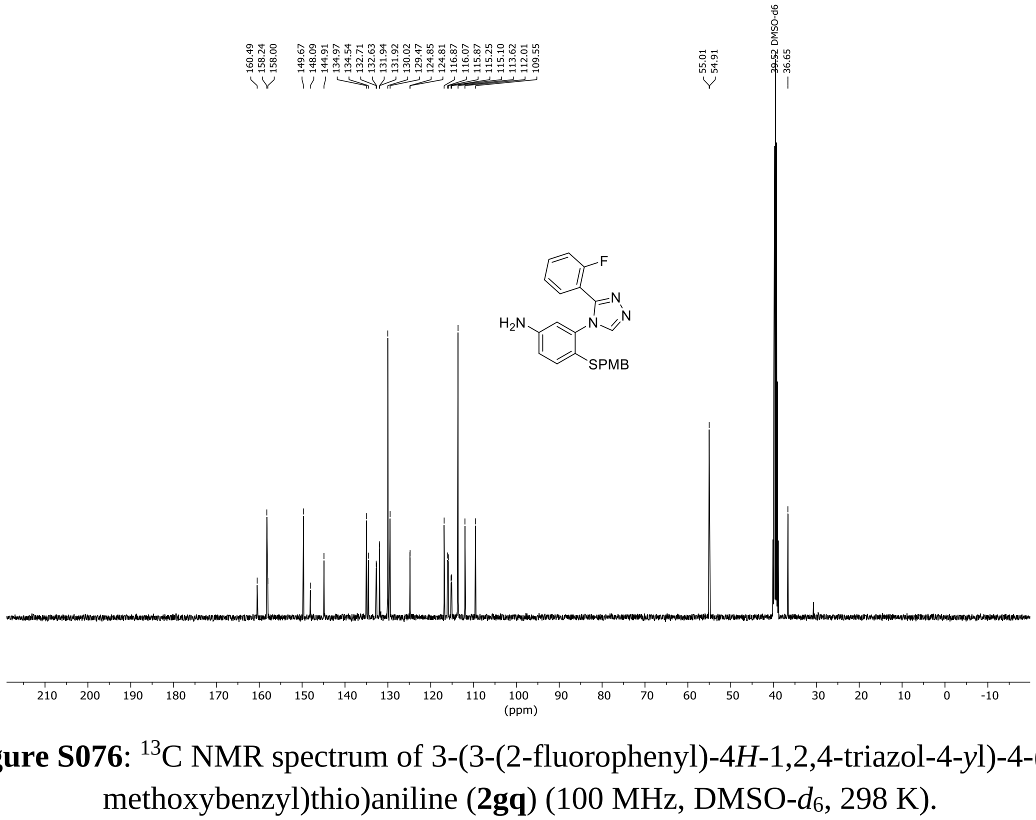

Figure S076: ^{13}C NMR spectrum of 3-(3-(2-fluorophenyl)-4*H*-1,2,4-triazol-4-yl)-4-((4-methoxybenzyl)thio)aniline (**2gq**) (100 MHz, DMSO-d_6, 298 K).

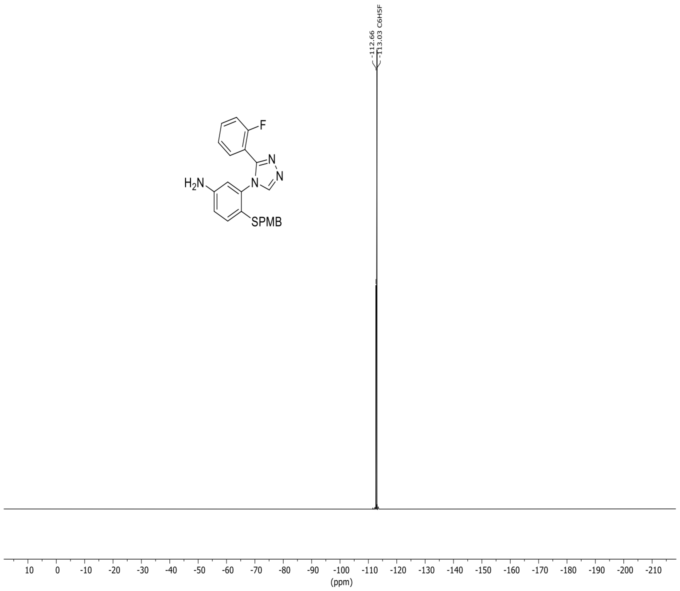

Figure S077: ^{19}F NMR spectrum of 3-(3-(2-fluorophenyl)-4*H*-1,2,4-triazol-4-yl)-4-((4-methoxybenzyl)thio)aniline (**2gq**) (376 MHz, DMSO-d_6, 298 K).

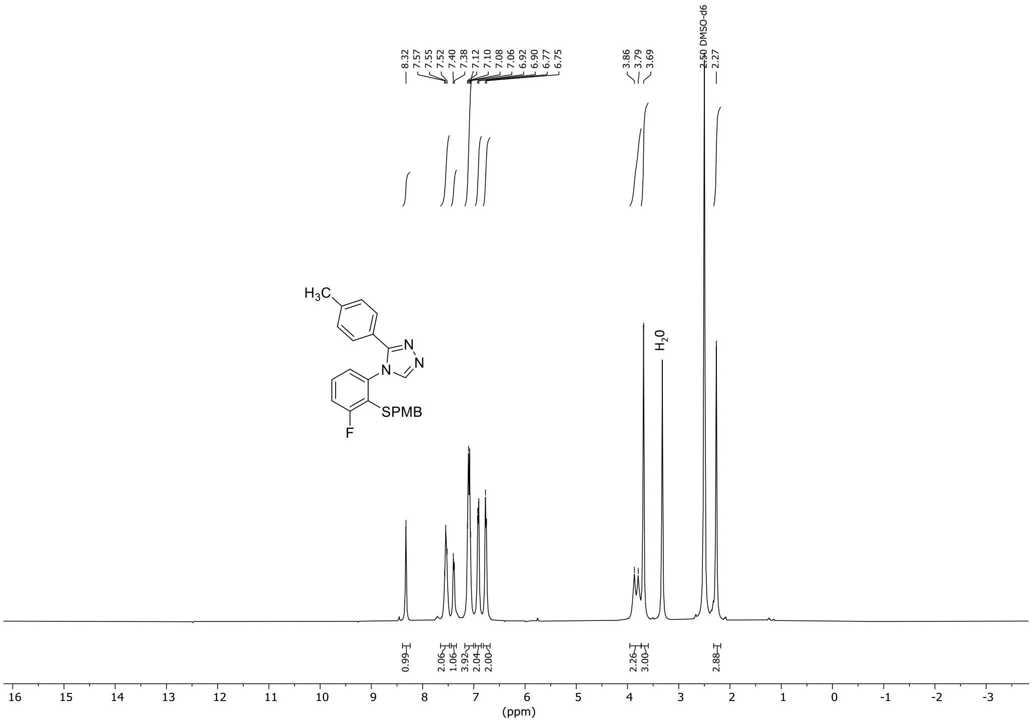

Figure S078: ^{1}H NMR spectrum of 4-(3-fluoro-2-((4-methoxybenzyl)thio)phenyl)-3-(p-tolyl)-4*H*-1,2,4-triazole (**2if**) (400 MHz, DMSO-d_6, 298 K).

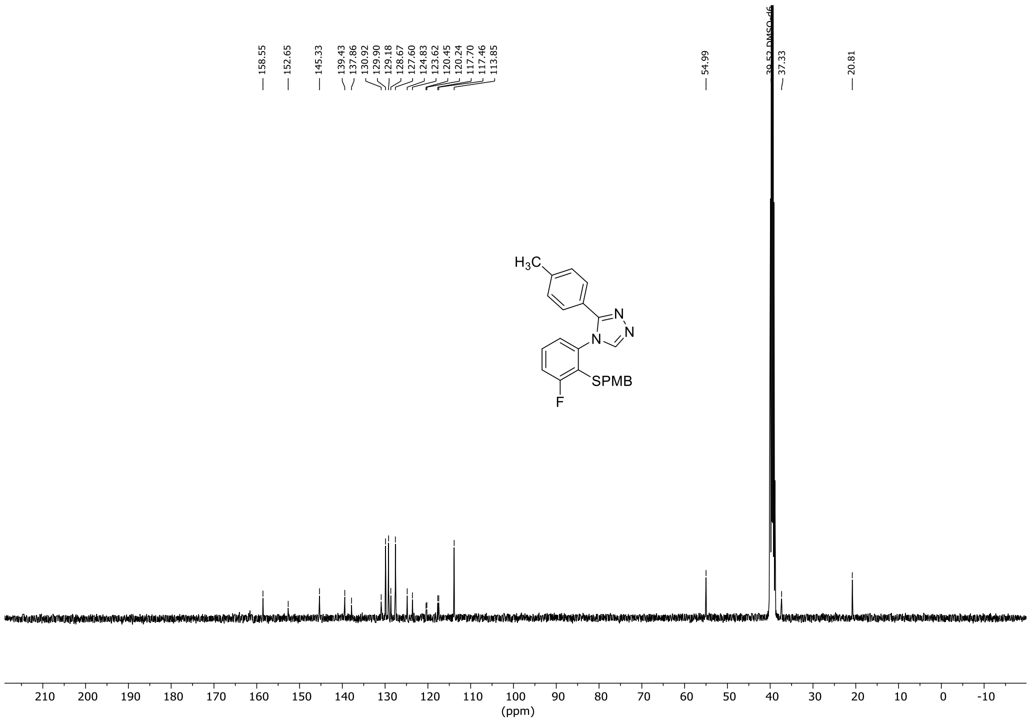

Figure S079: ^{13}C NMR spectrum of 4-(3-fluoro-2-((4-methoxybenzyl)thio)phenyl)-3-(p-tolyl)-4*H*-1,2,4-triazole (**2if**) (100 MHz, DMSO-d_6, 298 K).

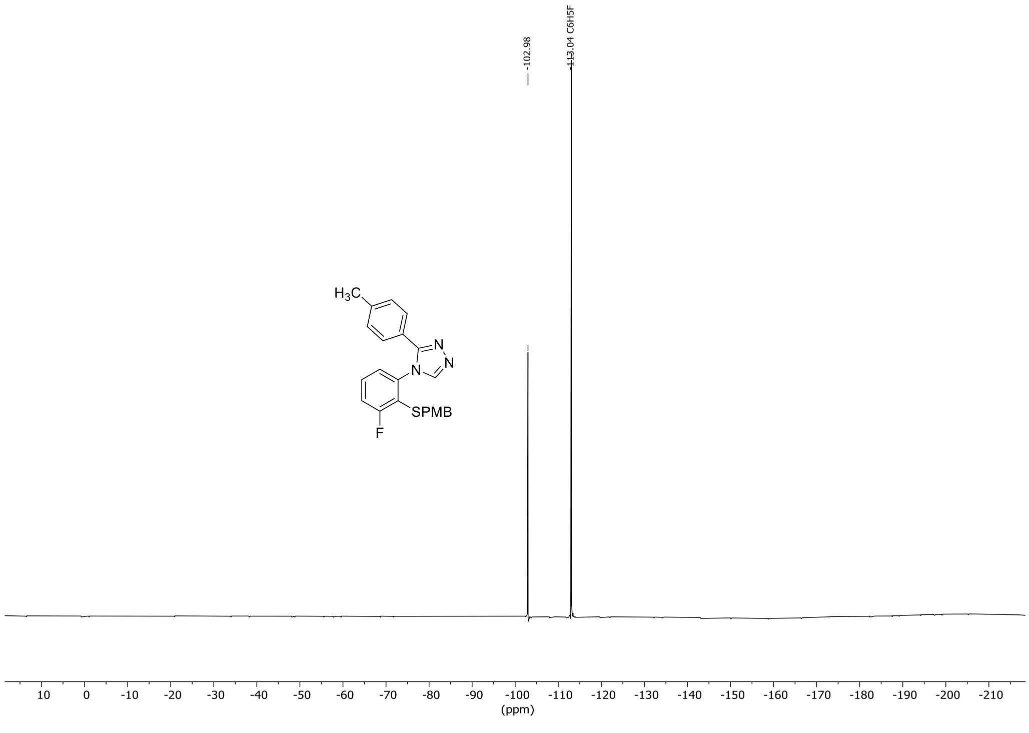

Figure S080: ^{19}F NMR spectrum of 4-(3-fluoro-2-((4-methoxybenzyl)thio)phenyl)-3-(p-tolyl)-4*H*-1,2,4-triazole (**2if**) (376 MHz, DMSO-d_6, 298 K).

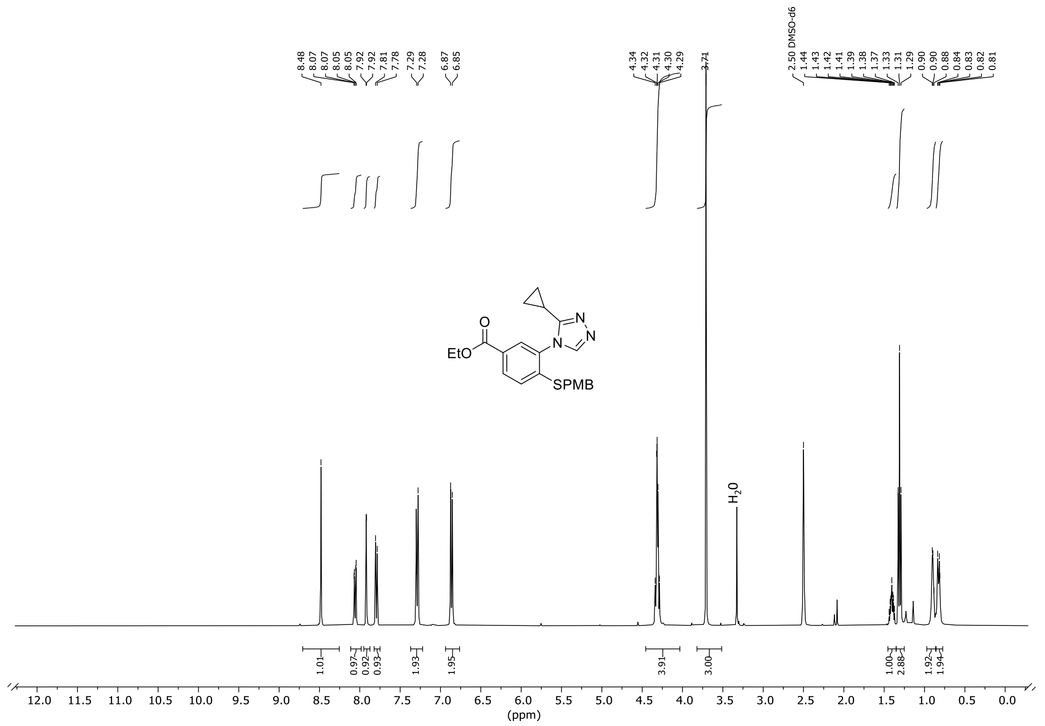

Figure S081: ^{1}H NMR spectrum of Ethyl 3-(3-cyclopropyl-4*H*-1,2,4-triazol-4-yl)-4-((4-methoxybenzyl)thio)benzoate (**2jc**) (400 MHz, DMSO-d_6, 298 K).

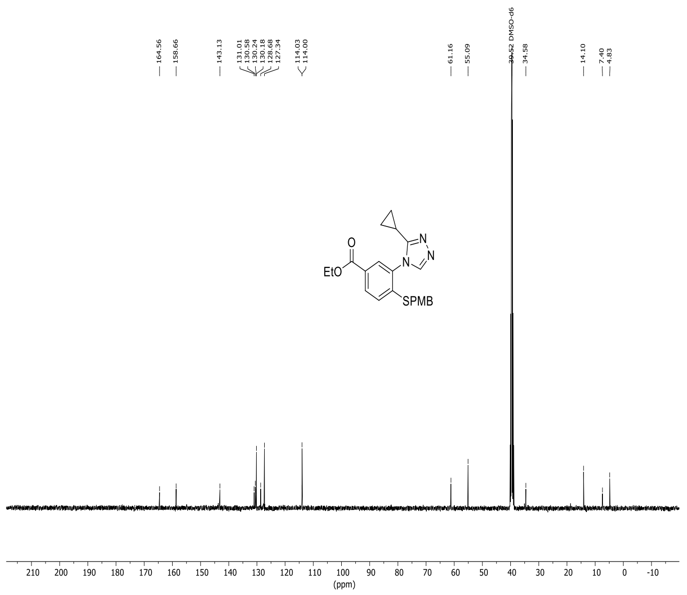

Figure S082: ^{13}C NMR spectrum of Ethyl 3-(3-cyclopropyl-4*H*-1,2,4-triazol-4-yl)-4-((4-methoxybenzyl)thio)benzoate (**2jc**) (100 MHz, DMSO-d_6, 298 K).

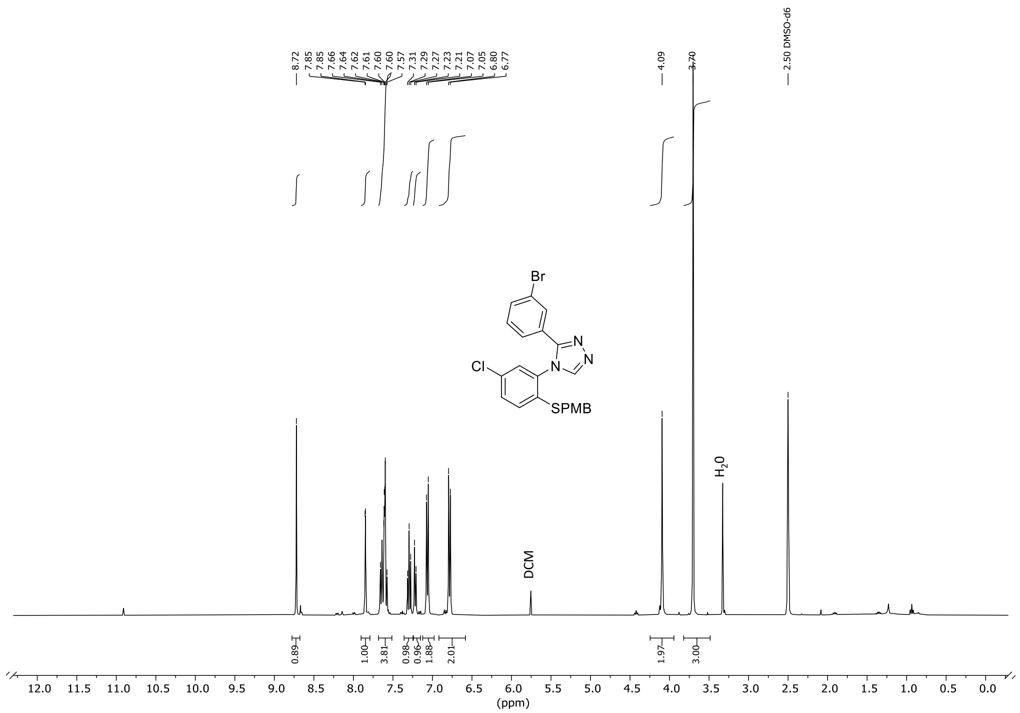

Figure S083 : ^{1}H NMR spectrum of 3-(3-bromophenyl)-4-(5-chloro-2-((4-methoxybenzyl)thio)phenyl)-4*H*-1,2,4-triazole (**2ko**) (400 MHz, DMSO-d_6, 298 K).

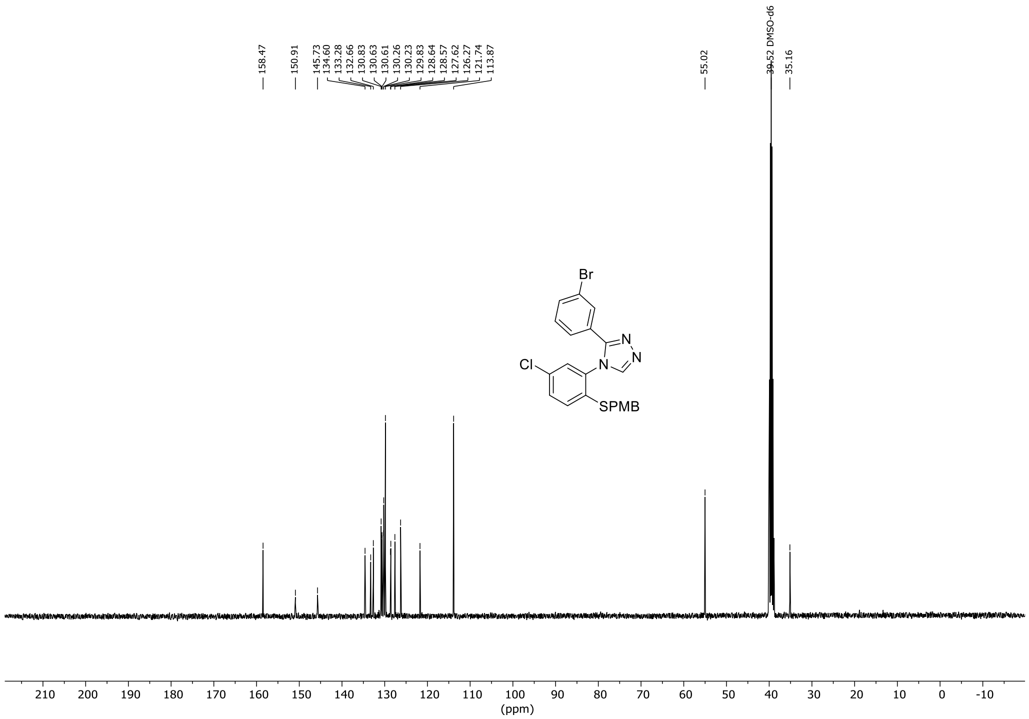

Figure S084: ^{13}C NMR spectrum 3-(3-bromophenyl)-4-(5-chloro-2-((4-methoxybenzyl)thio)phenyl)-4*H*-1,2,4-triazole (**2ko**) (100 MHz, DMSO-d_6, 298 K).

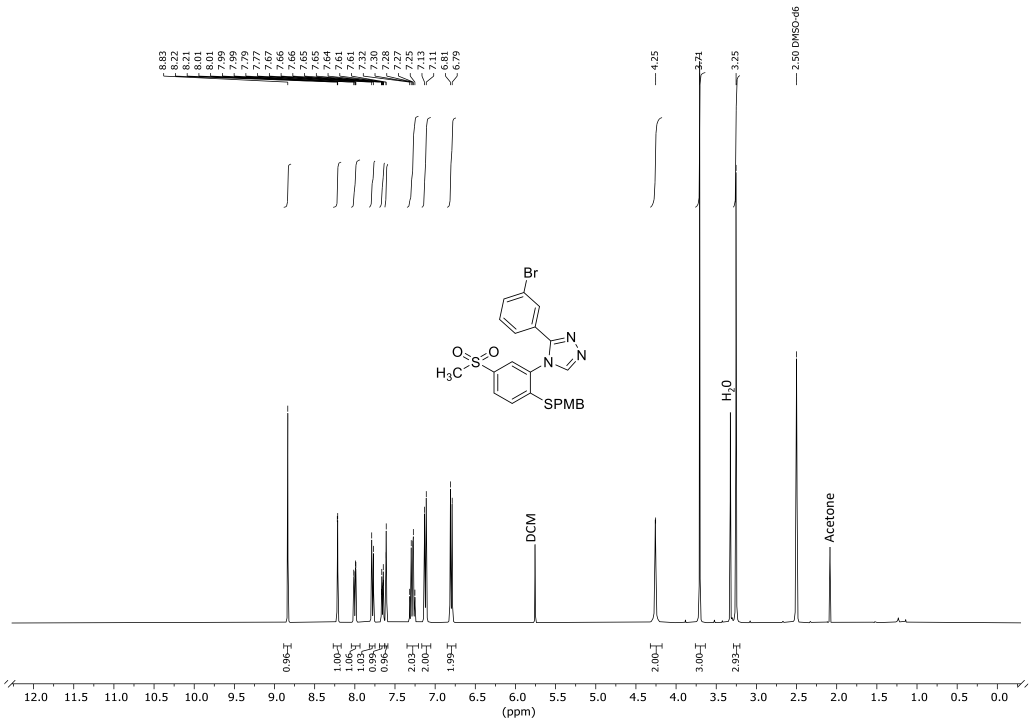

Figure S085: ^{1}H NMR spectrum of 3-(3-bromophenyl)-4-(2-((4-methoxybenzyl)thio)-5-(methylsulfonyl)phenyl)-4*H*-1,2,4-triazole (**2mo**) (400 MHz, DMSO-d_6, 298 K).

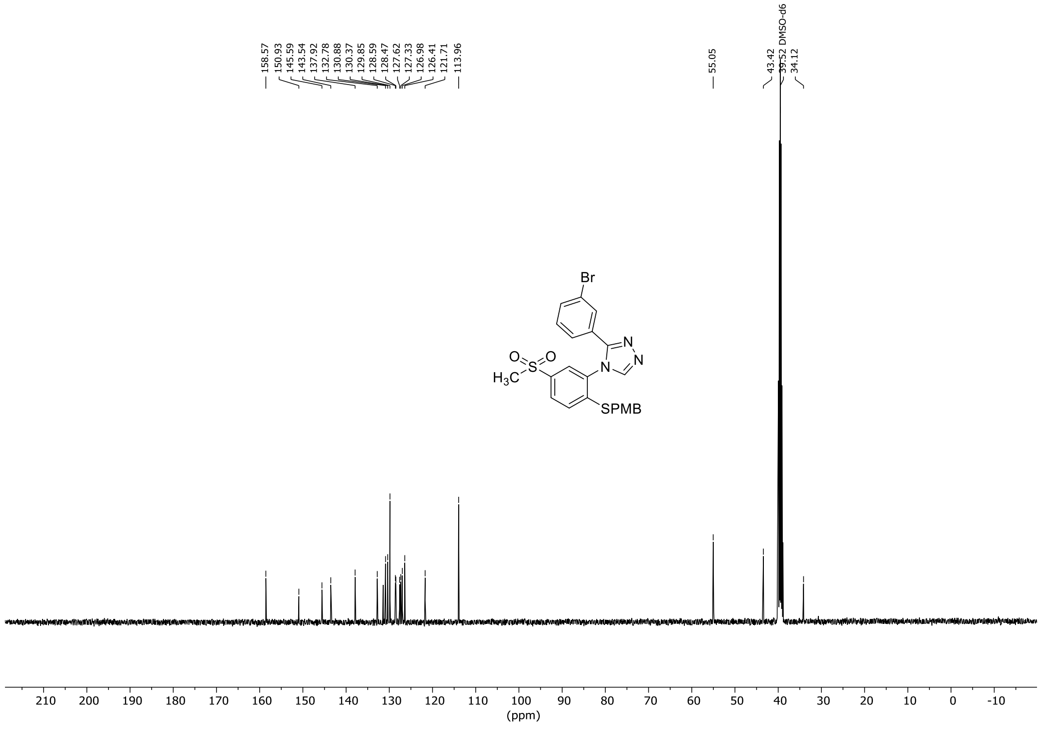

Figure S086: ^{13}C NMR spectrum of 3-(3-bromophenyl)-4-(2-((4-methoxybenzyl)thio)-5-(methylsulfonyl)phenyl)-4*H*-1,2,4-triazole (**2mo**) (100 MHz, DMSO-d_6, 298 K).

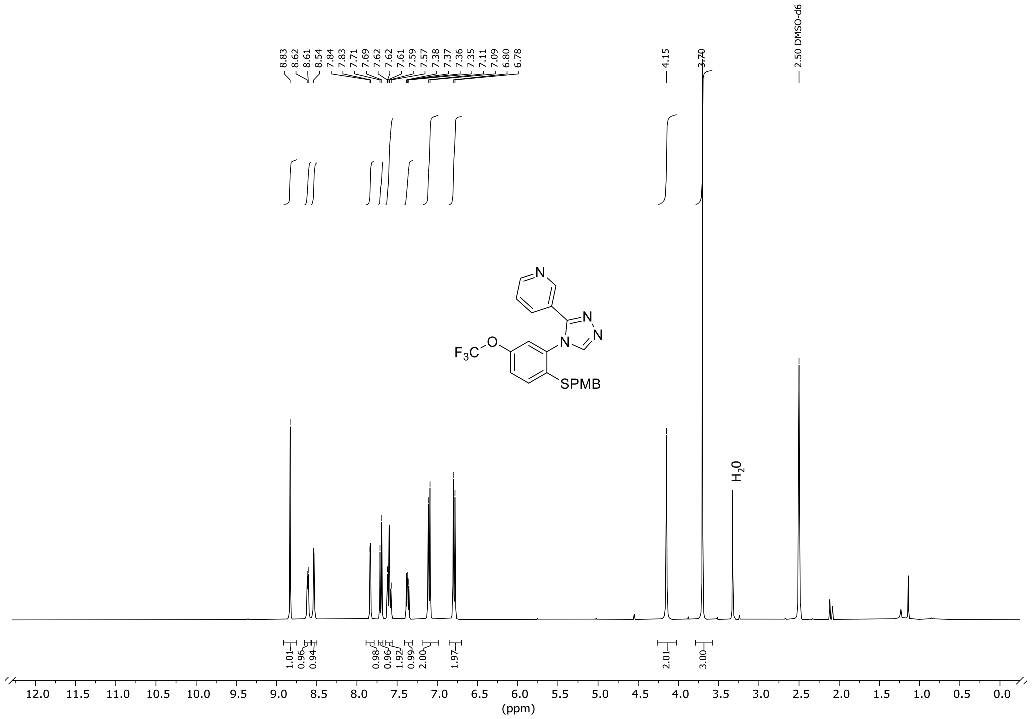

Figure S087: ^{1}H NMR spectrum of 3-(4-(2-((4-methoxybenzyl)thio)-5-(trifluoromethoxy)phenyl)-4*H*-1,2,4-triazol-3-yl)pyridine (**2or**) (400 MHz, DMSO-d_6, 298 K).

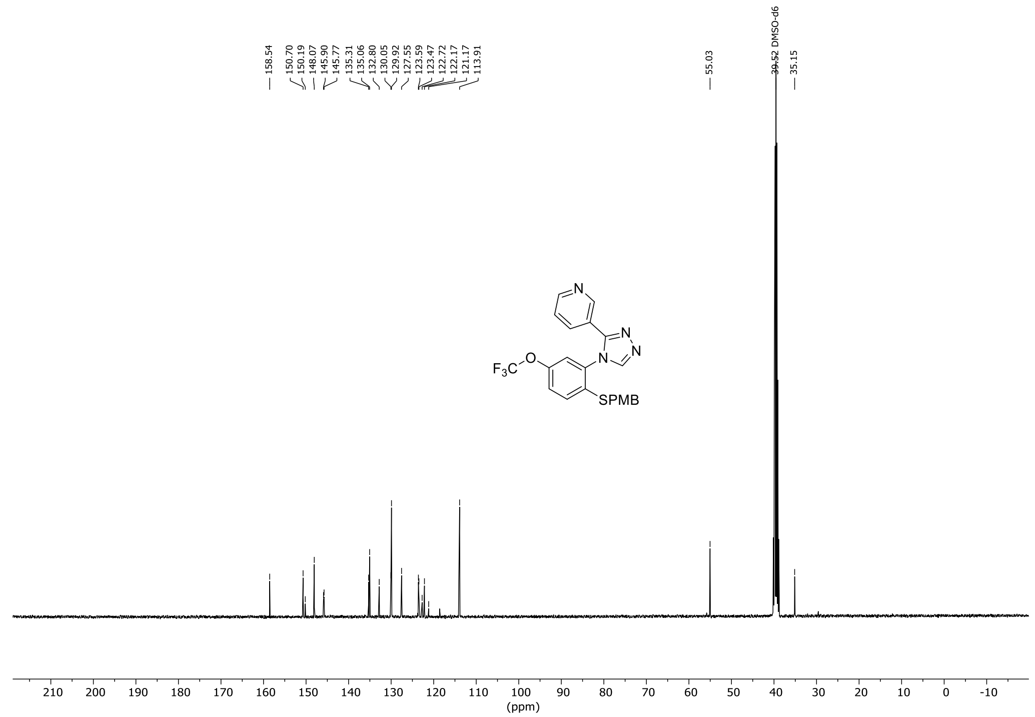

Figure S088: ^{13}C NMR spectrum of 3-(4-(2-((4-methoxybenzyl)thio)-5-(trifluoromethoxy)phenyl)-4*H*-1,2,4-triazol-3-yl)pyridine (**2or**) (100 MHz, DMSO-d_6, 298 K).

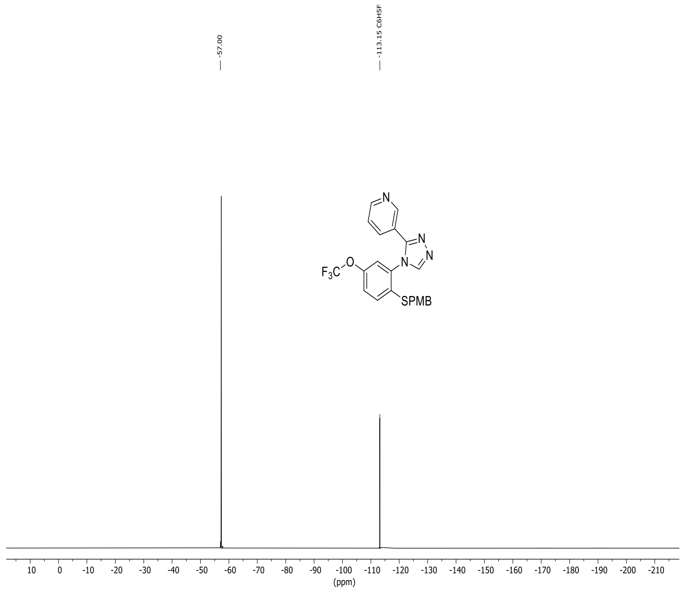

Figure S089: ^{19}F NMR spectrum of of 3-(4-(2-((4-methoxybenzyl)thio)-5-(trifluoromethoxy)phenyl)-4*H*-1,2,4-triazol-3-yl)pyridine (**2or**) (376 MHz, DMSO-d_6, 298 K).

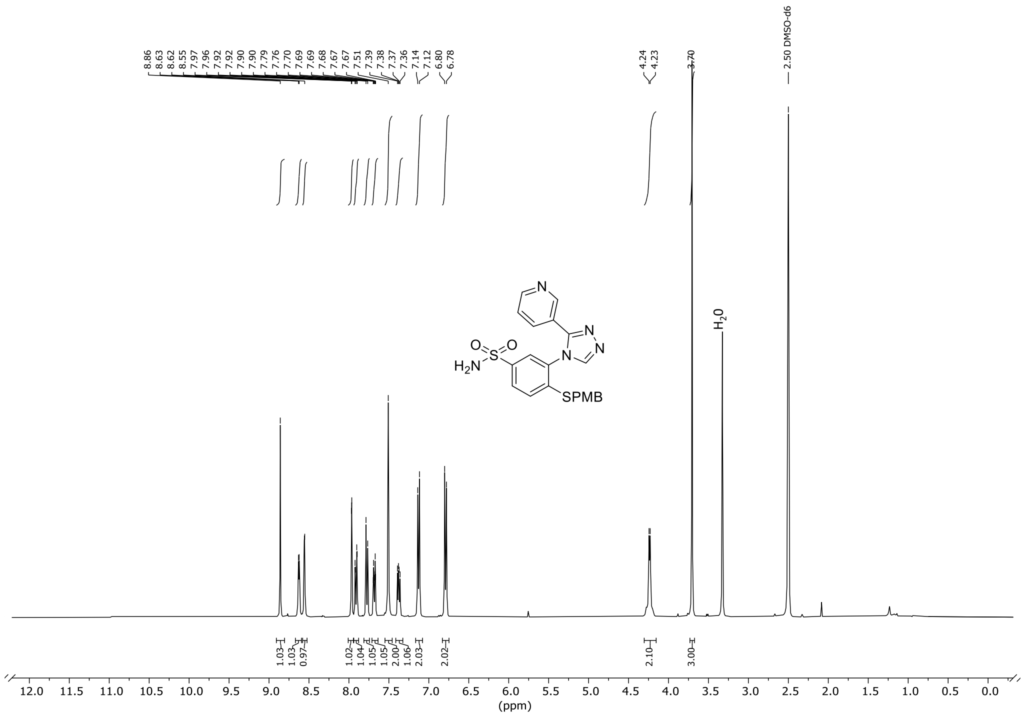

Figure S090: ^{1}H NMR spectrum of 4-((4-methoxybenzyl)thio)-3-(3-(pyridin-2-yl)-4*H*-1,2,4-triazol-4-yl)benzenesulfonamide (**2pr**) (400 MHz, DMSO-d_6, 298 K).

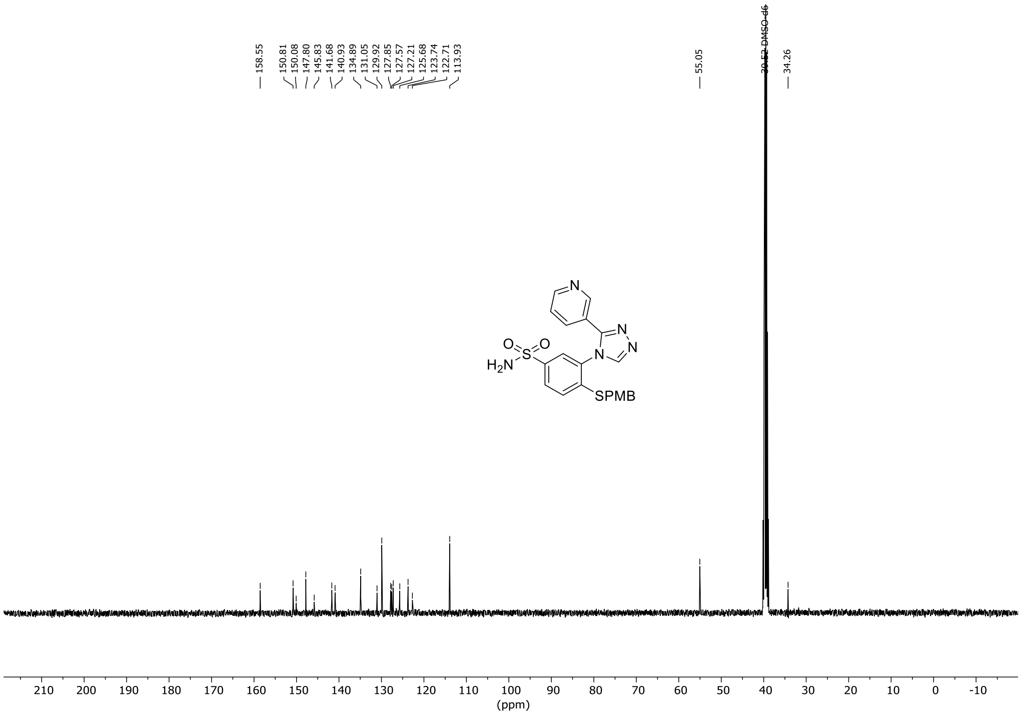

Figure S091: ^{13}C NMR spectrum of 4-((4-methoxybenzyl)thio)-3-(3-(pyridin-2-yl)-4*H*-1,2,4-triazol-4-yl)benzenesulfonamide (**2pr**) (100 MHz, DMSO-d_6, 298 K).

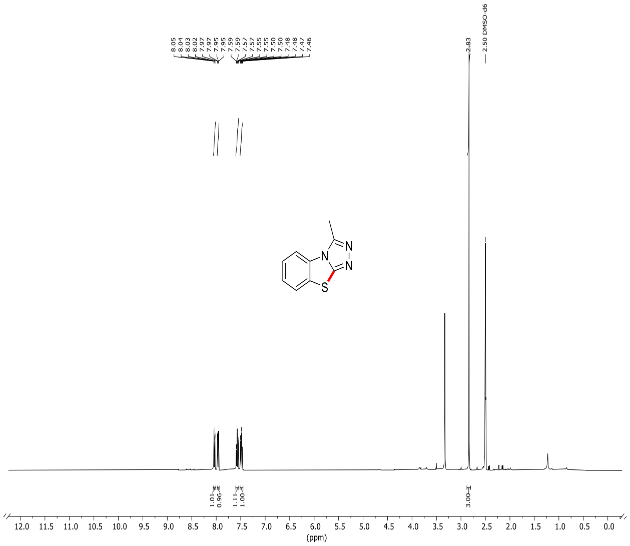

Figure S092: ^{1}H NMR spectrum of 3-Methylbenzo[4,5]thiazolo[2,3-*c*][1,2,4]triazole **(4ab)** (400 MHz, DMSO-d_6, 298 K)

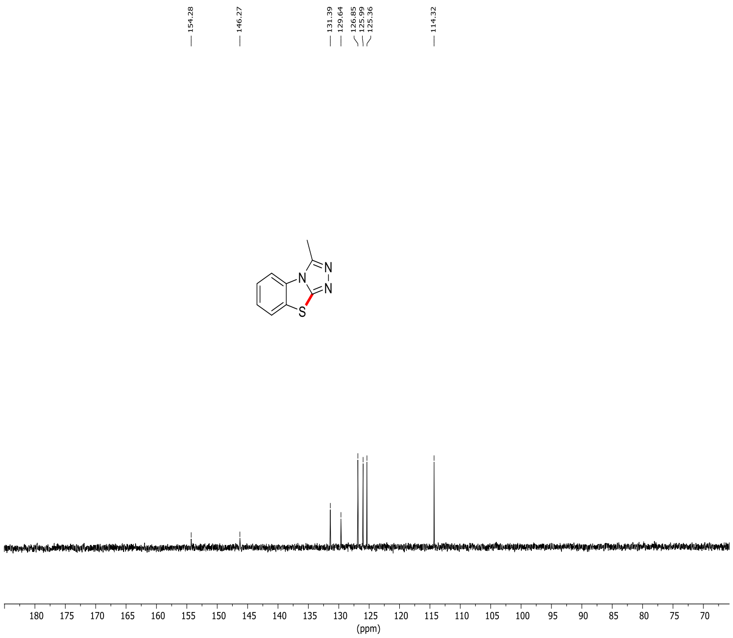

Figure S093: ^{13}C NMR spectrum of 3-Methylbenzo[4,5]thiazolo[2,3-*c*][1,2,4]triazole **(4ab)** (100 MHz, DMSO-d_6, 298 K)

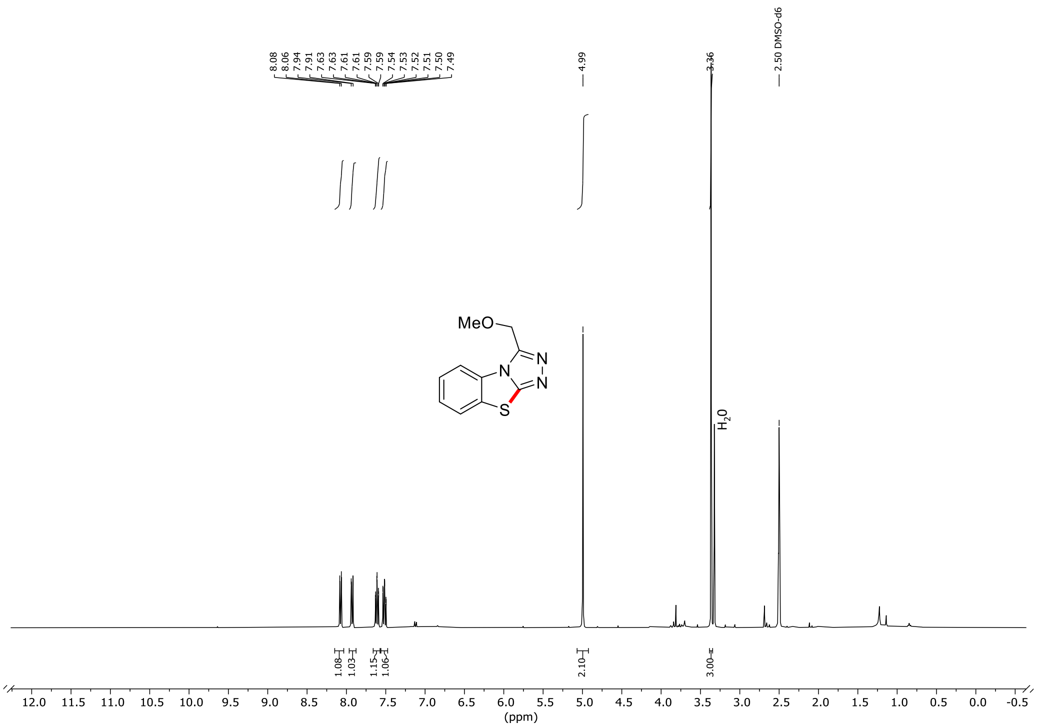

Figure S094: ^{1}H NMR spectrum of 3-(methoxymethyl)benzo[4,5]thiazolo[2,3-*c*][1,2,4]triazole (**4ad**) (400 MHz, DMSO-d_6, 298 K).

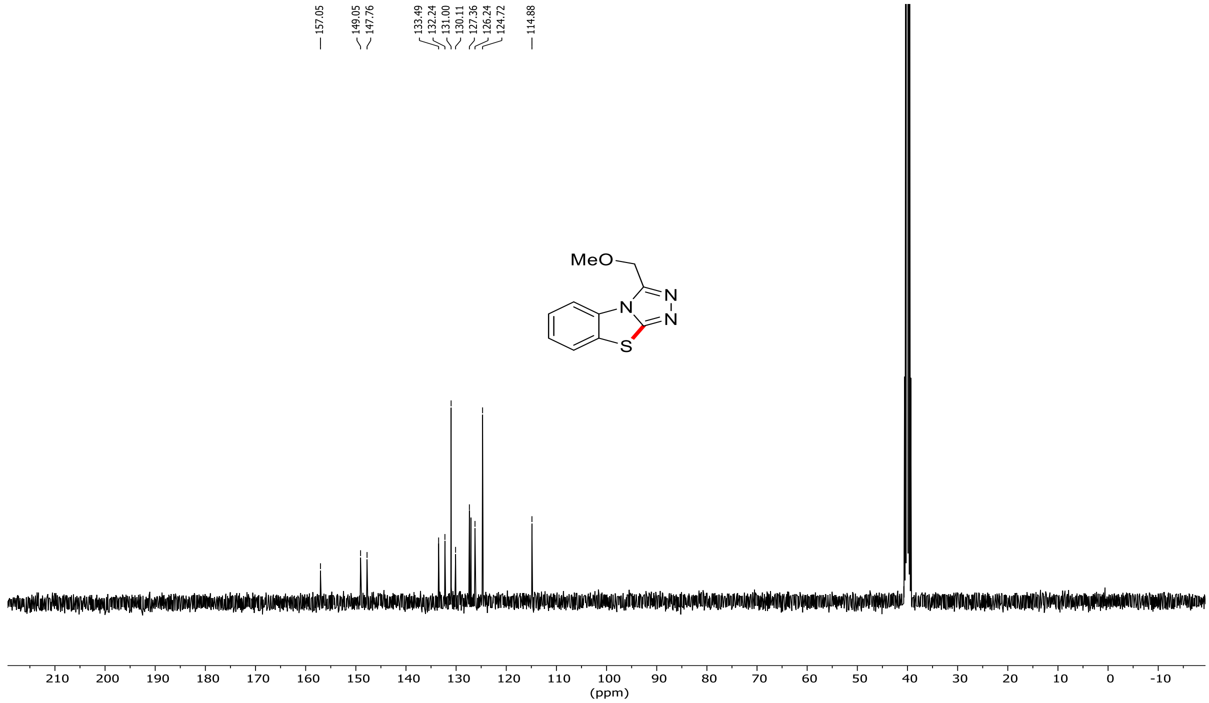

Figure S095: ^{13}C NMR spectrum of (3-(methoxymethyl)benzo[4,5]thiazolo[2,3-*c*][1,2,4]triazole (**4ad**) (100 MHz, DMSO-d_6, 298 K

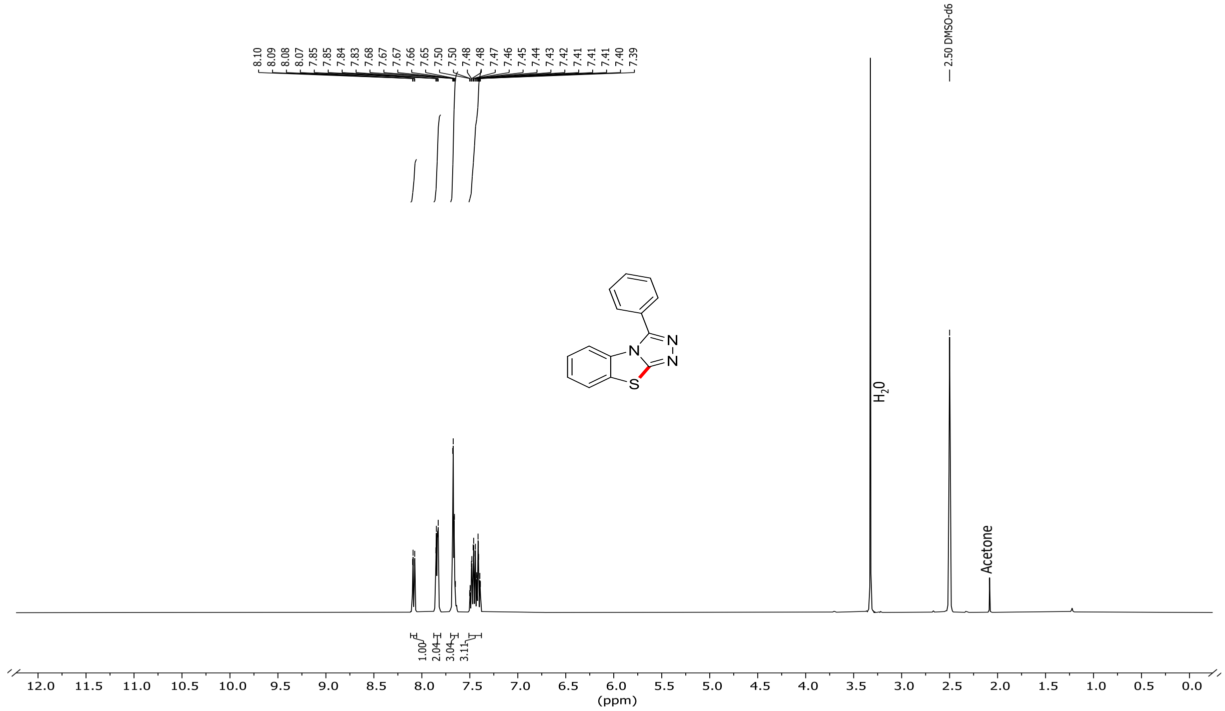

Figure S096: ^{1}H NMR spectrum of 3-phenylbenzo[4,5]thiazolo[2,3-*c*][1,2,4]triazole (**4ae**) (400 MHz, DMSO-d_6, 298 K).

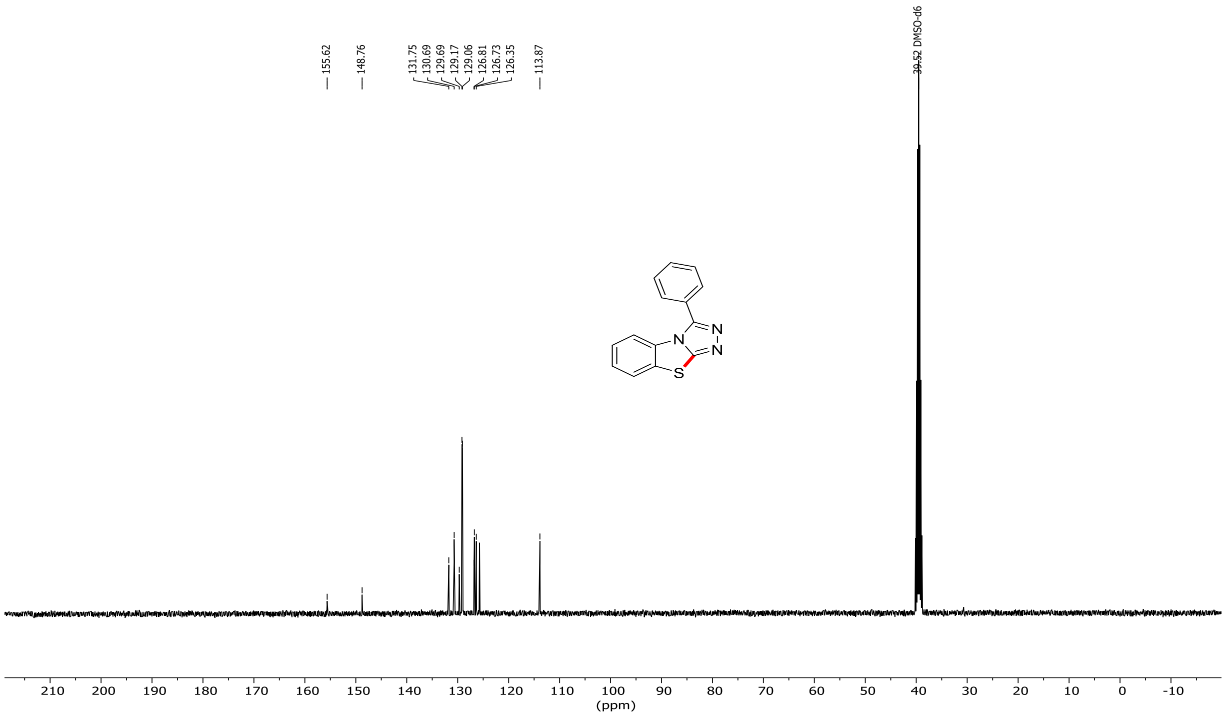

Figure S097: ^{13}C NMR spectrum of 3-phenylbenzo[4,5]thiazolo[2,3-*c*][1,2,4]triazole (**4ae**) (100 MHz, DMSO-d_6, 298 K).

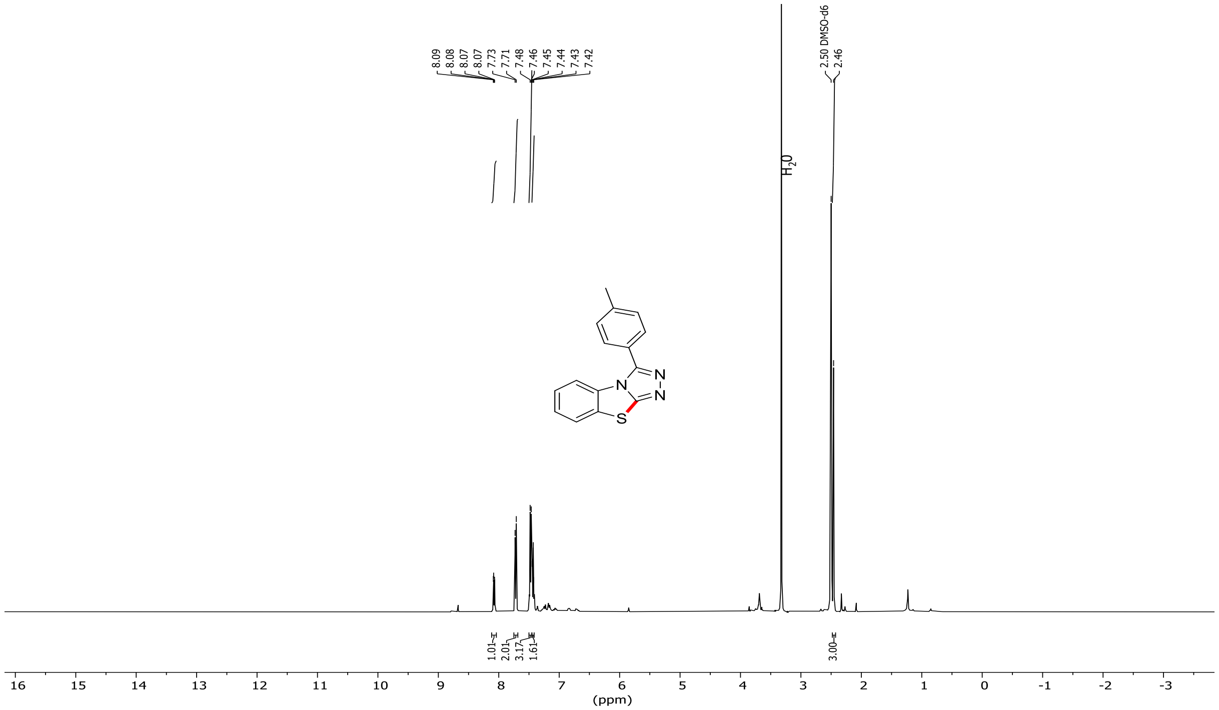

Figure S098: ^{1}H NMR spectrum of 3-(p-tolyl)benzo[4,5]thiazolo[2,3-*c*][1,2,4]triazole (**4af**) (400 MHz, DMSO-d_6, 298 K).

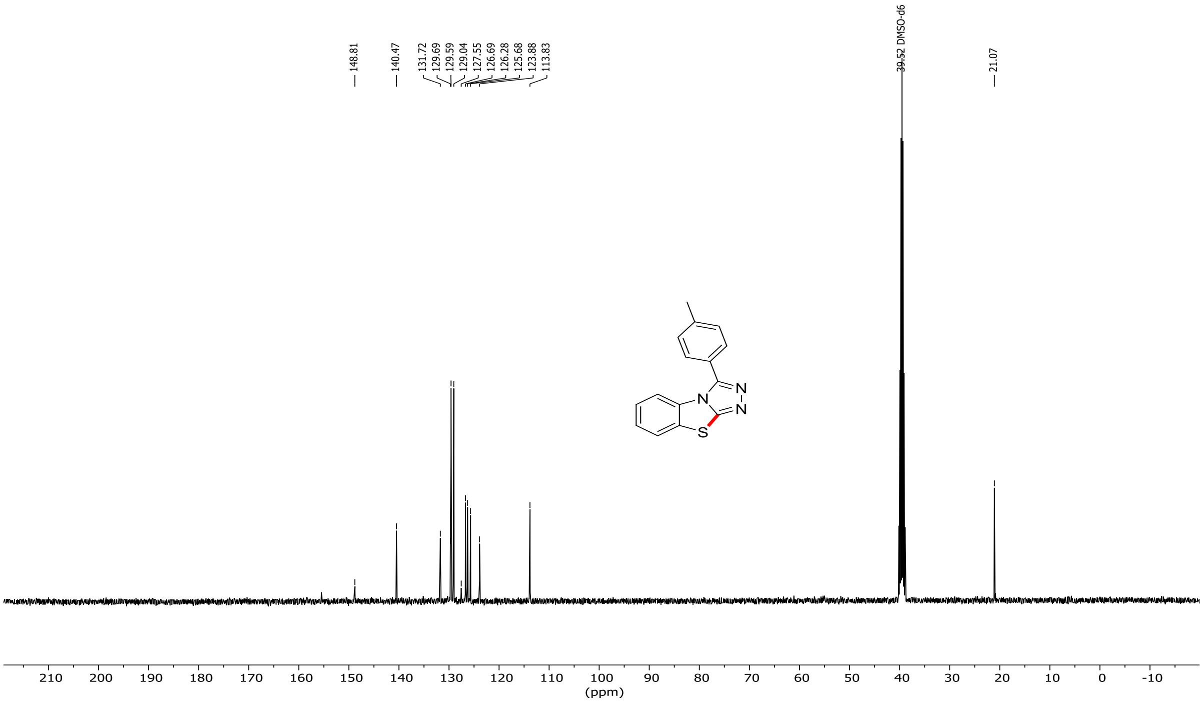

Figure S099: ^{13}C NMR spectrum of 3-(p-tolyl)benzo[4,5]thiazolo[2,3-*c*][1,2,4]triazole (**4af**) (100 MHz, DMSO-d_6, 298 K).

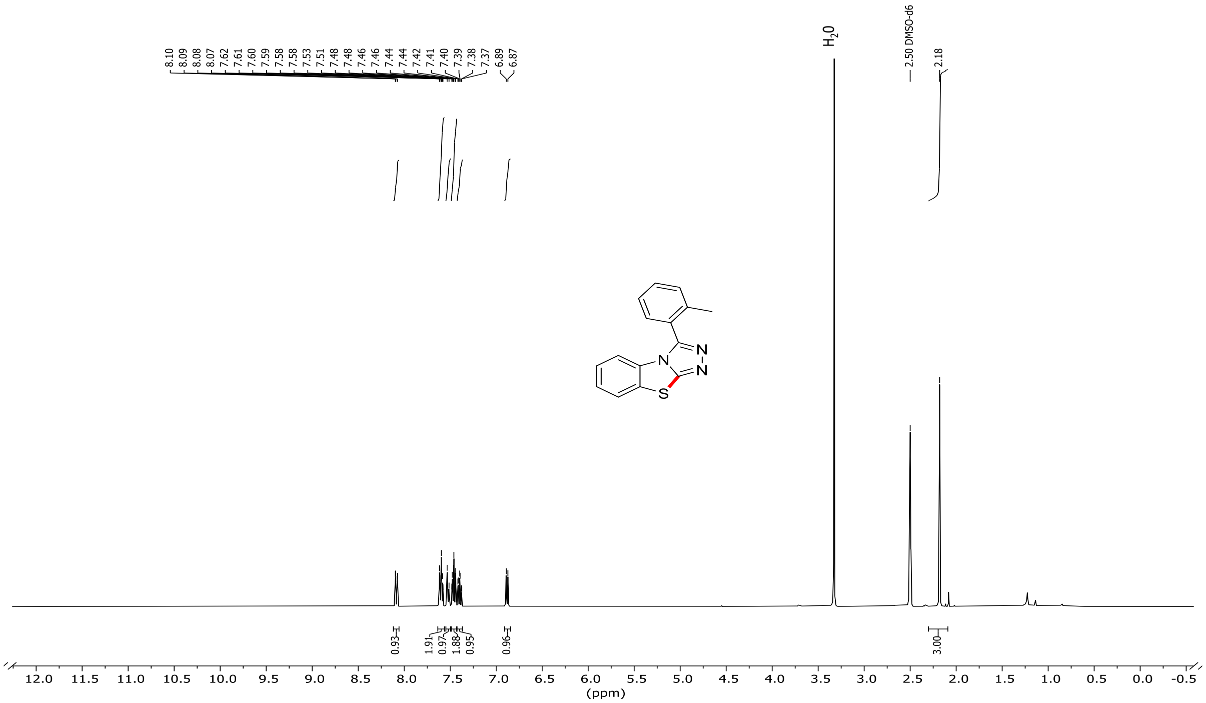

Figure S100: ^{1}H NMR spectrum of 3-(o-tolyl)benzo[4,5]thiazolo[2,3-*c*][1,2,4]triazole (**4ag**) (400 MHz, DMSO-d_6, 298 K).

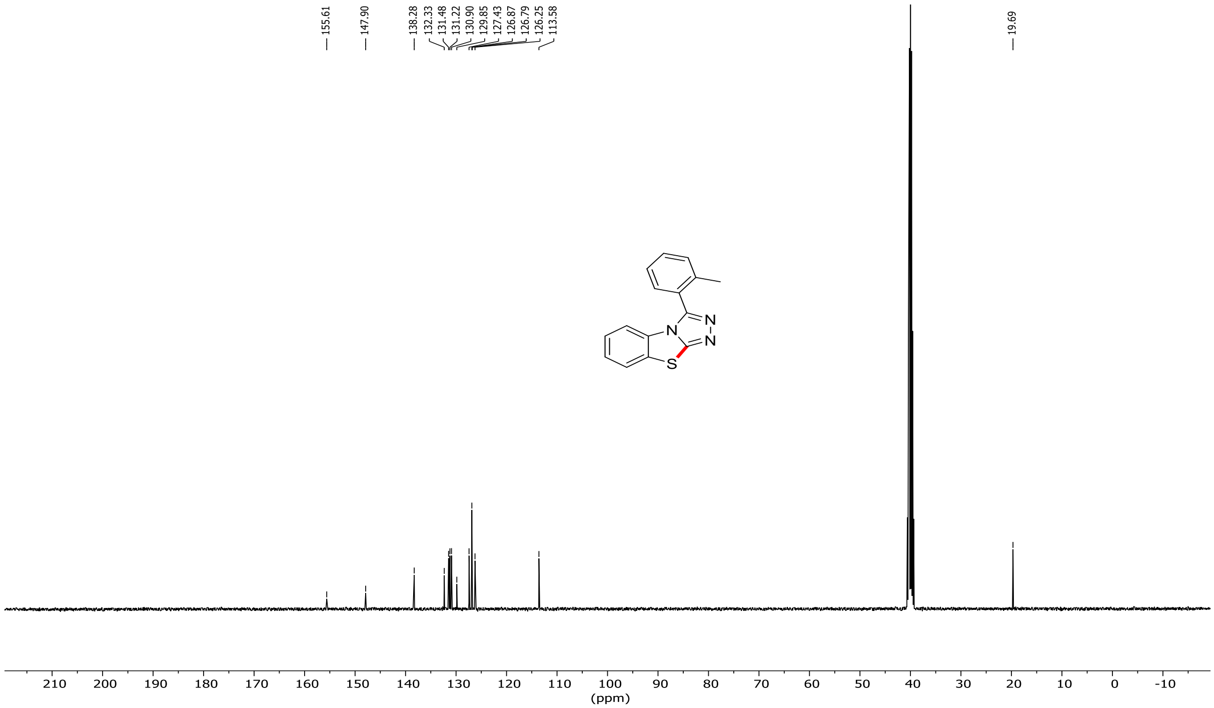

Figure S101: ^{13}C NMR spectrum of 3-(o-tolyl)benzo[4,5]thiazolo[2,3-*c*][1,2,4]triazole (**4ag**) (100 MHz, DMSO-d_6, 298 K).

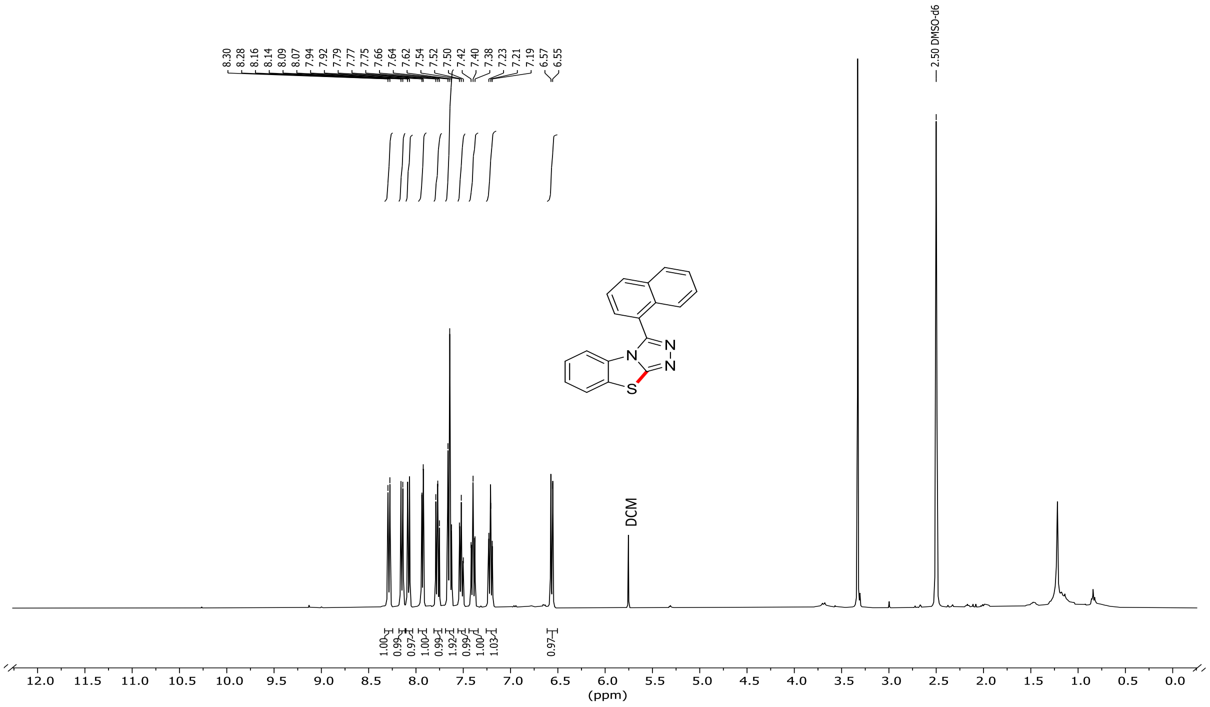

Figure S102: ^{1}H NMR spectrum of 3-(naphthalen-1-yl)benzo[4,5]thiazolo[2,3-*c*][1,2,4]triazole (**4ah**) (400 MHz, DMSO-d_6, 298 K).

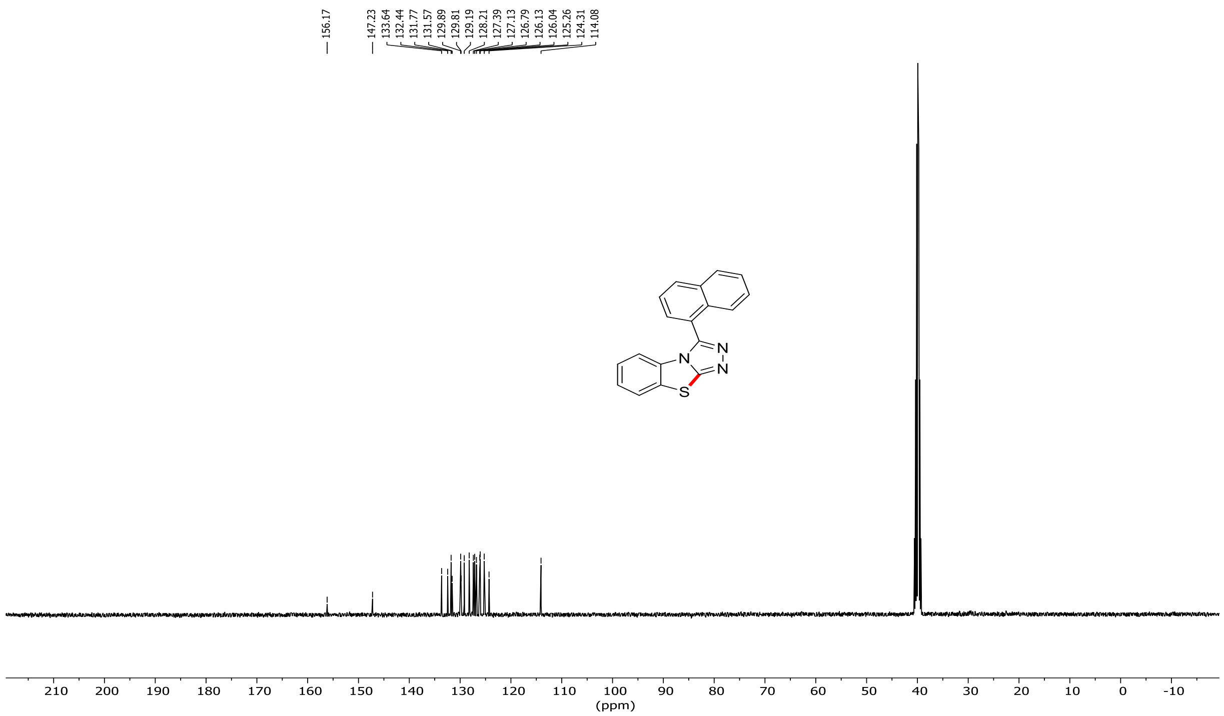

Figure S103: ^{13}C NMR spectrum of 3-(naphthalen-1-yl)benzo[4,5]thiazolo[2,3-*c*][1,2,4]triazole (**4ah**) (100 MHz, DMSO-d_6, 298 K).

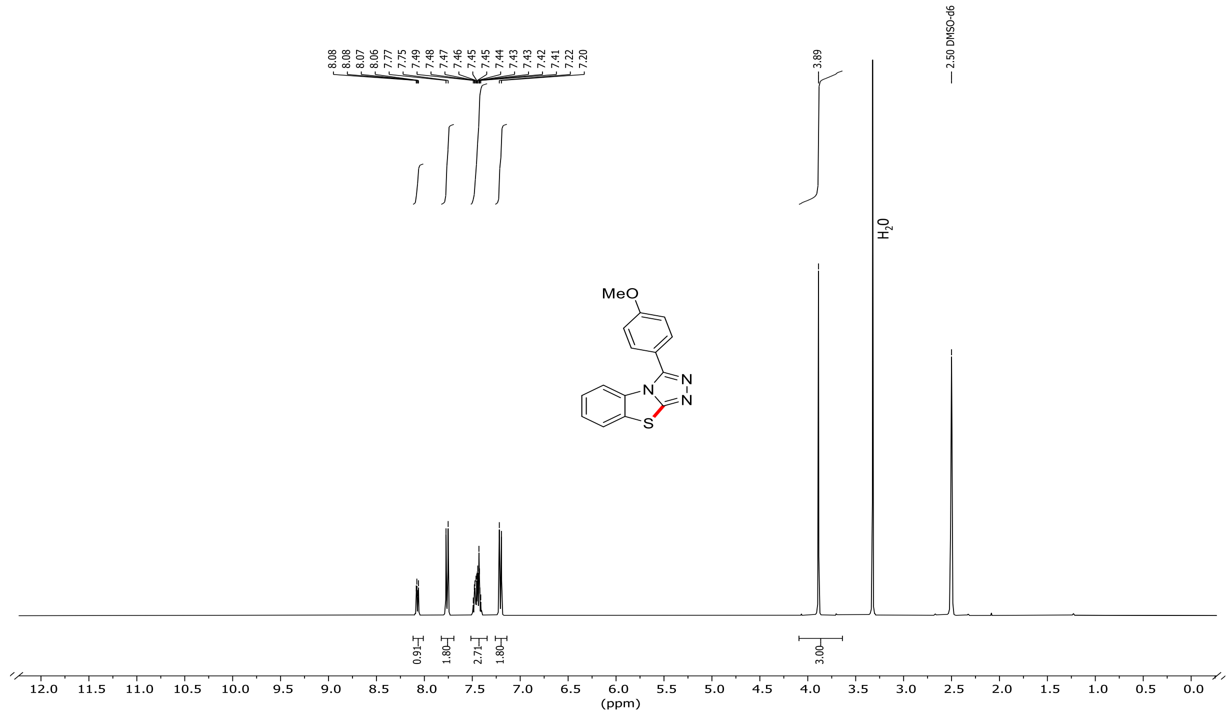

Figure S104: ^{1}H NMR spectrum of 3-(4-methoxyphenyl)benzo[4,5]thiazolo[2,3-*c*][1,2,4]triazole (**4ai**) (400 MHz, DMSO-d_6, 298 K).

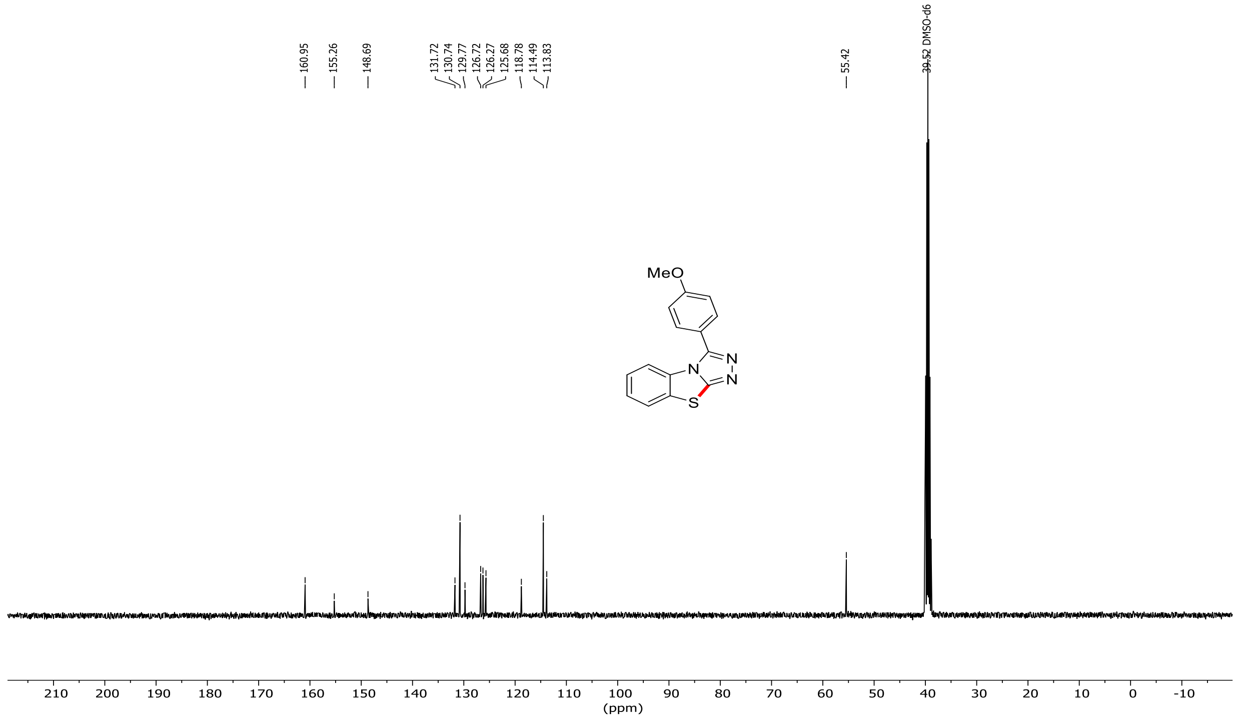

Figure S105: ^{13}C NMR spectrum of 3-(4-methoxyphenyl)benzo[4,5]thiazolo[2,3-*c*][1,2,4]triazole (**4ai**) (100 MHz, DMSO-d_6, 298 K).

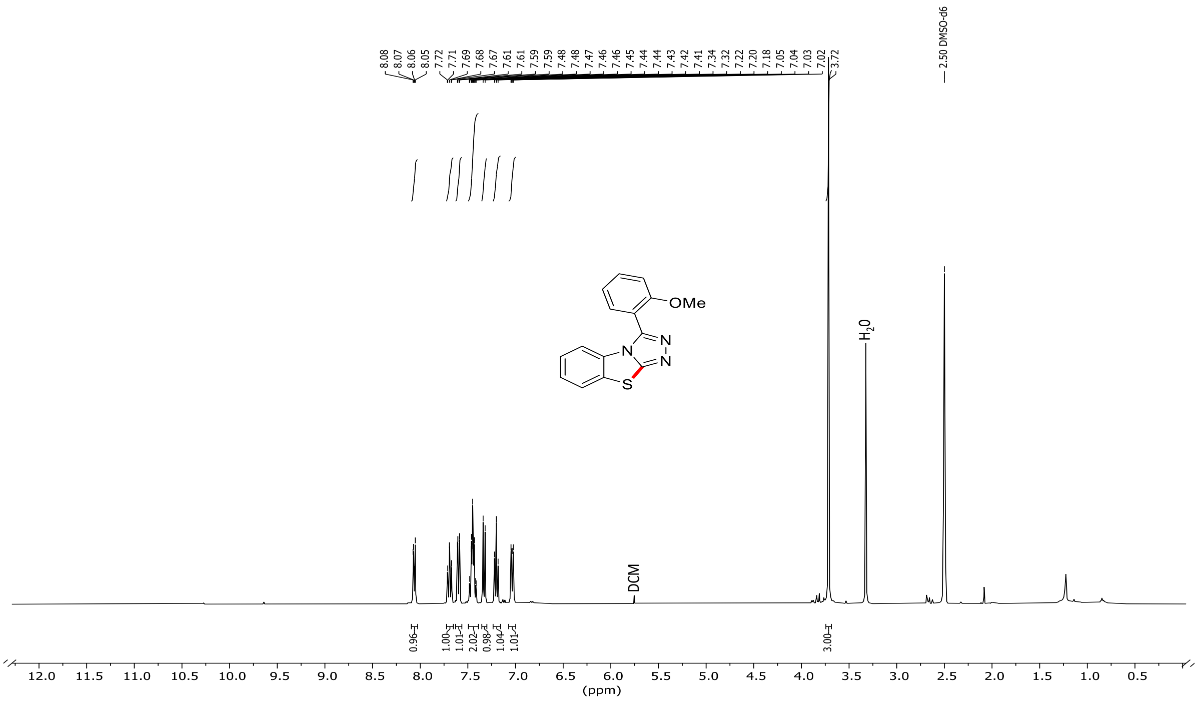

Figure S106: ^{1}H NMR spectrum of 3-(2-methoxyphenyl)benzo[4,5]thiazolo[2,3-*c*][1,2,4]triazole (**4aj**) (400 MHz, DMSO-d_6, 298 K).

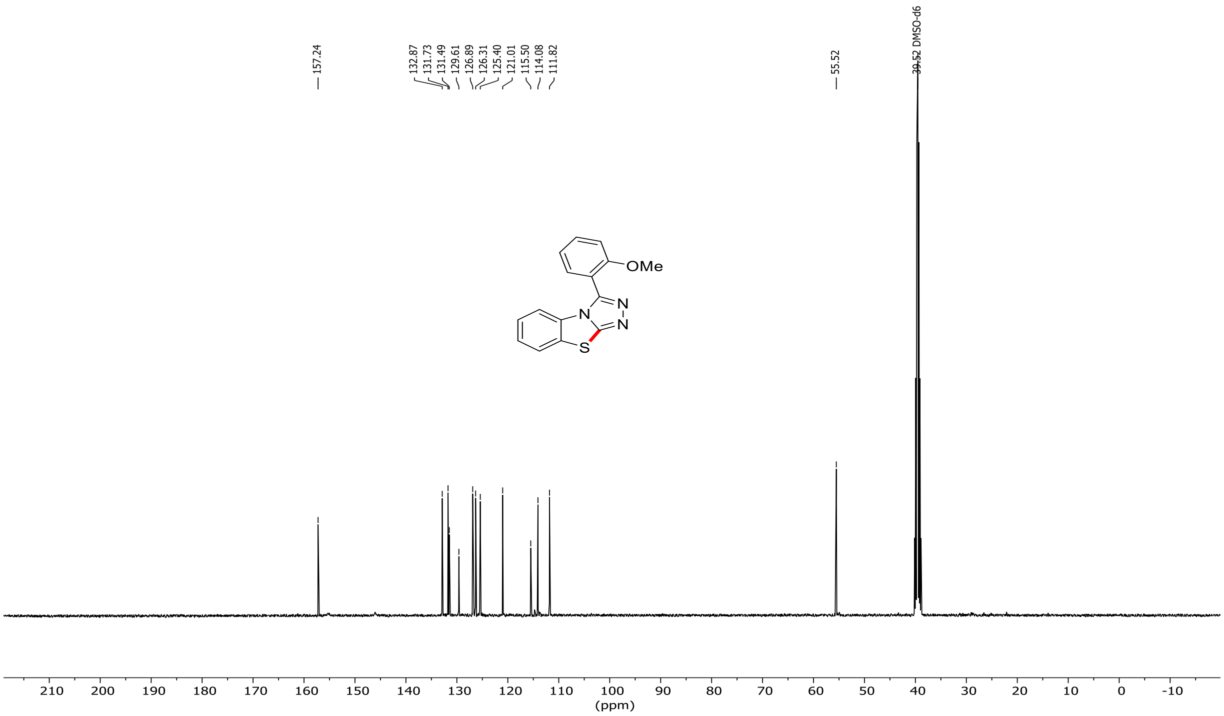

Figure S107: ^{13}C NMR spectrum of 3-(2-methoxyphenyl)benzo[4,5]thiazolo[2,3-*c*][1,2,4]triazole (**4aj**) (100 MHz, DMSO-d_6, 298 K).

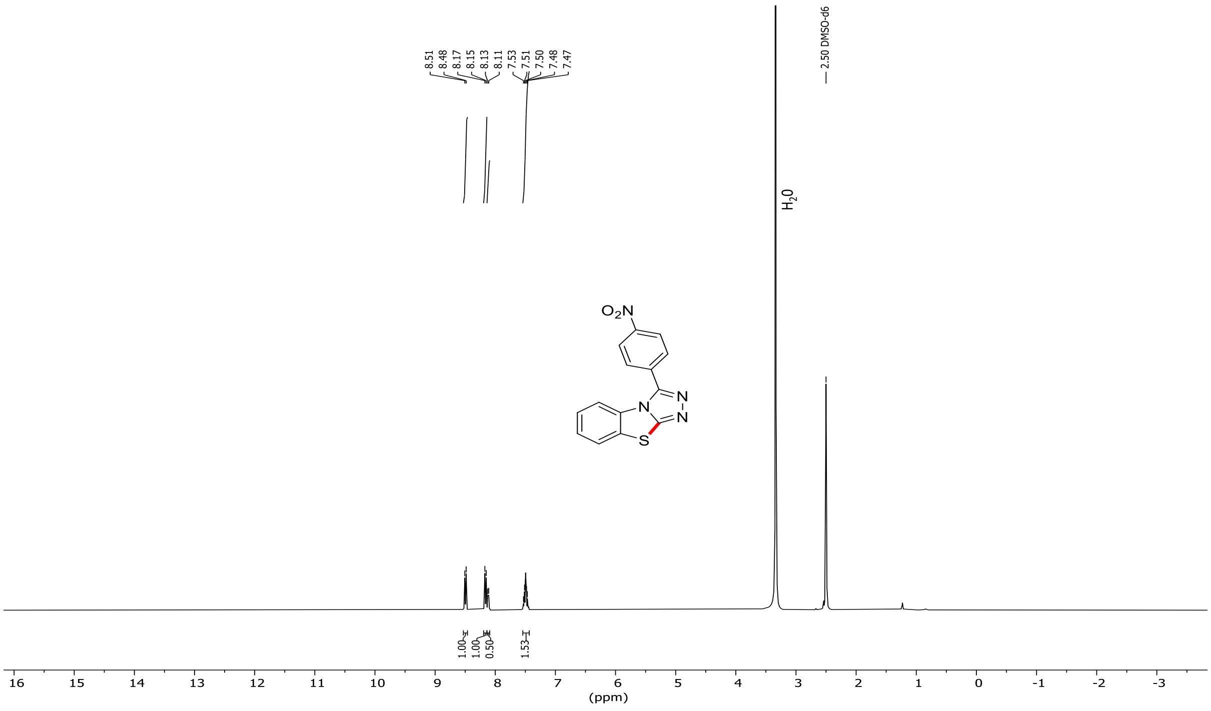

Figure S108: ^{1}H NMR spectrum of 3-(4-nitrophenyl)benzo[4,5]thiazolo[2,3-*c*][1,2,4]triazole (**4al**) (400 MHz, DMSO-d_6, 298 K).

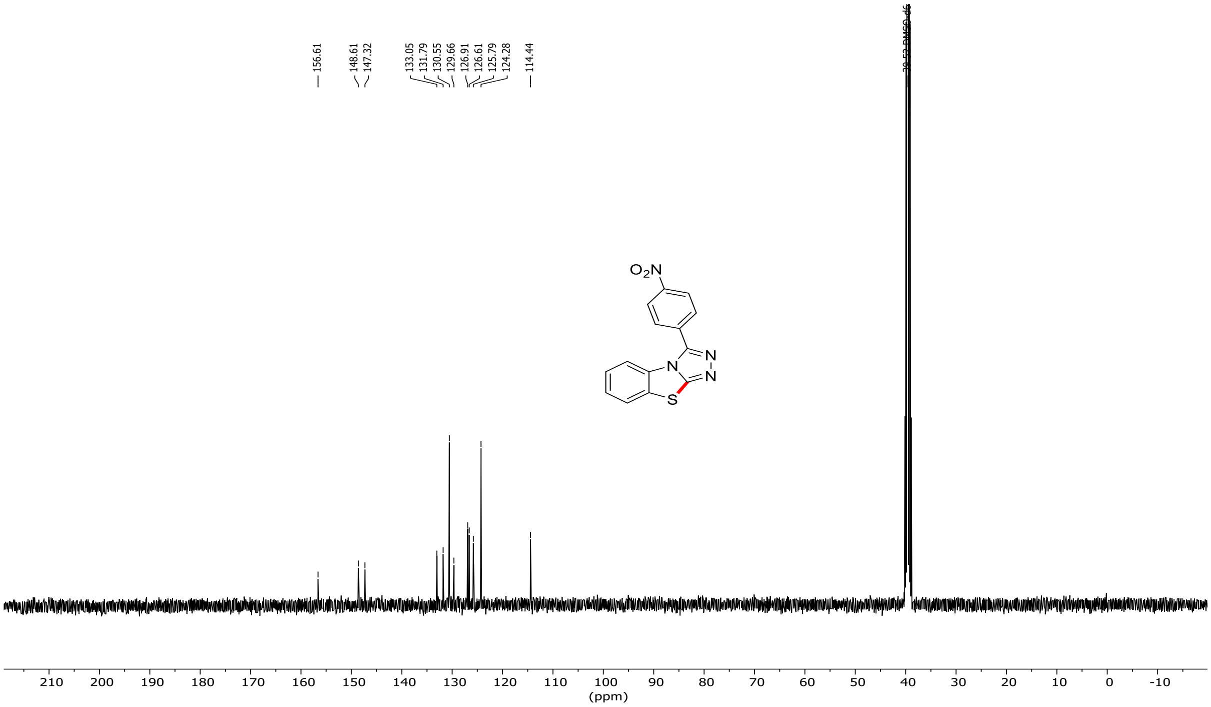

Figure S109: ^{13}C NMR spectrum of 3-(4-nitrophenyl)benzo[4,5]thiazolo[2,3-*c*][1,2,4]triazole (**4al**) (100 MHz, DMSO-d_6, 298 K).

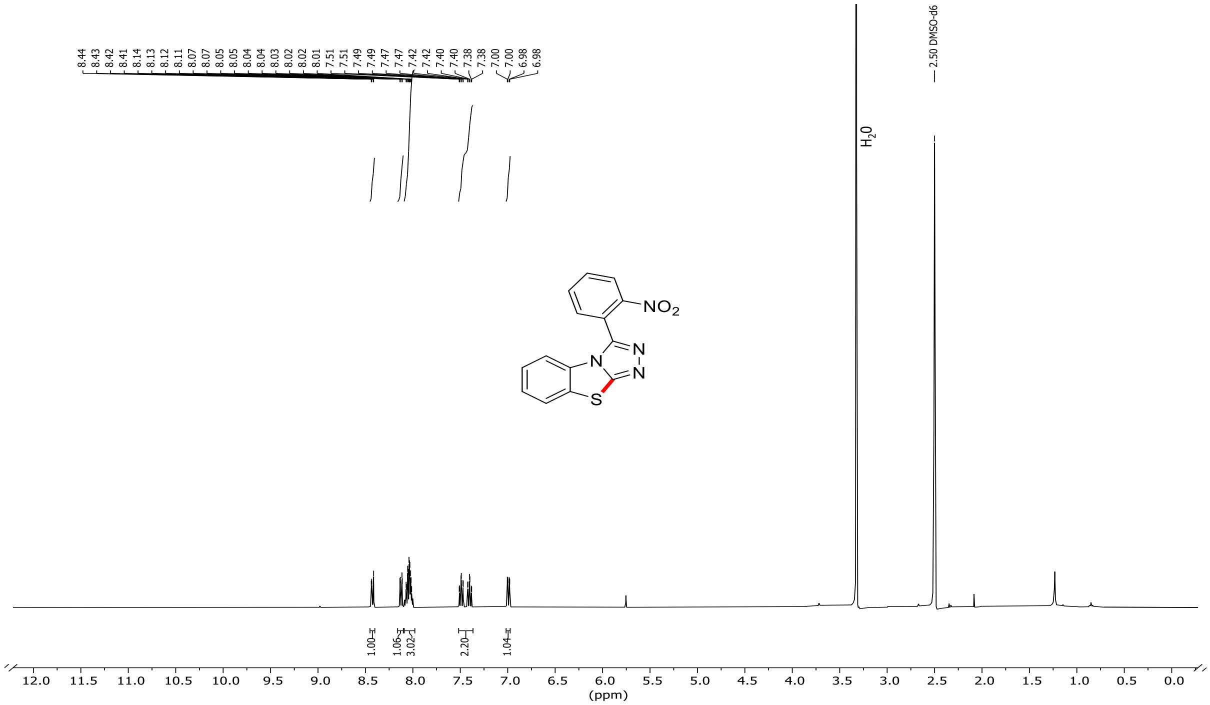

Figure S110: ^{1}H NMR spectrum of 3-(2-nitrophenyl)benzo[4,5]thiazolo[2,3-*c*][1,2,4]triazole. (**4am**) (400 MHz, DMSO-d_6, 298 K).

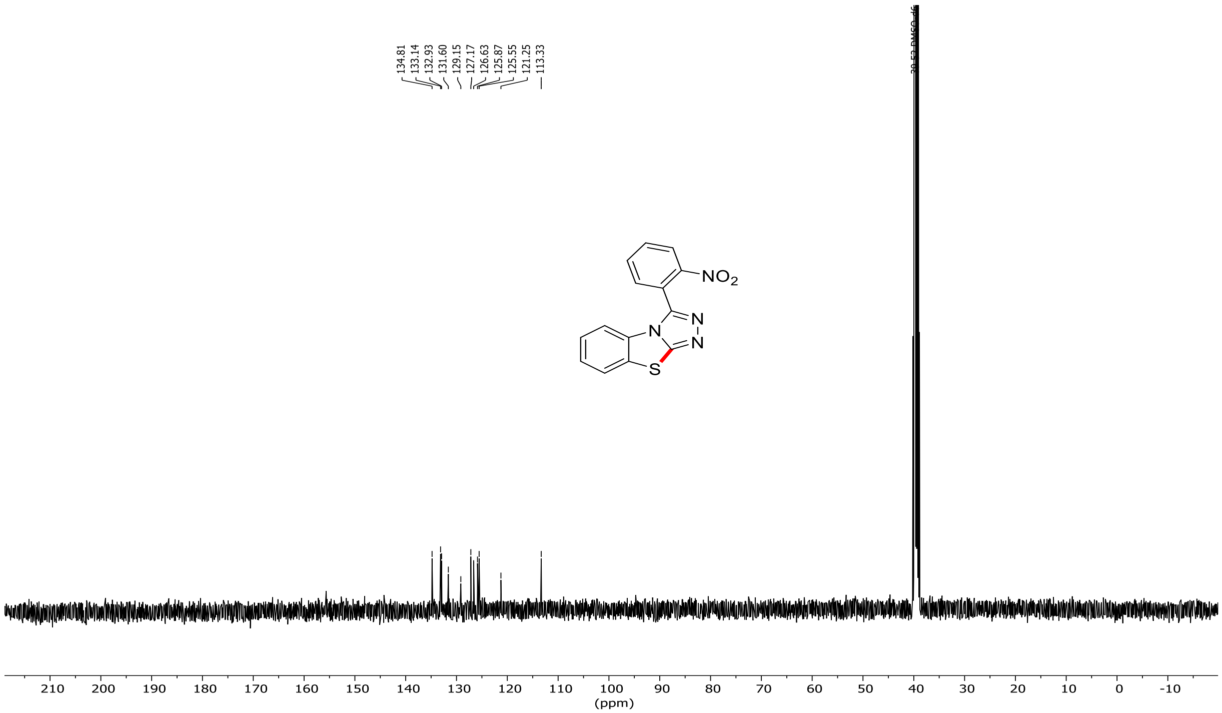

Figure S111: ^{13}C NMR spectrum of 3-(2-nitrophenyl)benzo[4,5]thiazolo[2,3-*c*][1,2,4]triazole (**4am**) (100 MHz, DMSO-d_6, 298 K).

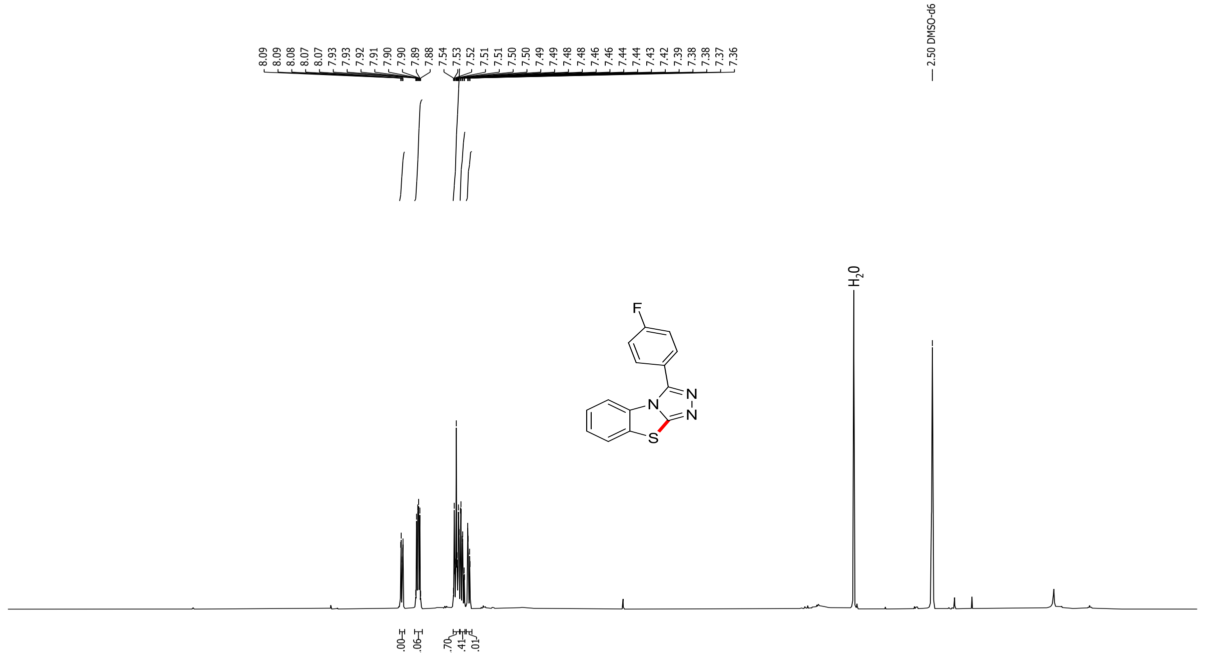

Figure S112: ^{1}H NMR spectrum of 3-(4-fluorophenyl)benzo[4,5]thiazolo[2,3-*c*][1,2,4]triazole (**4an**) (400 MHz, DMSO-d_6, 298 K).

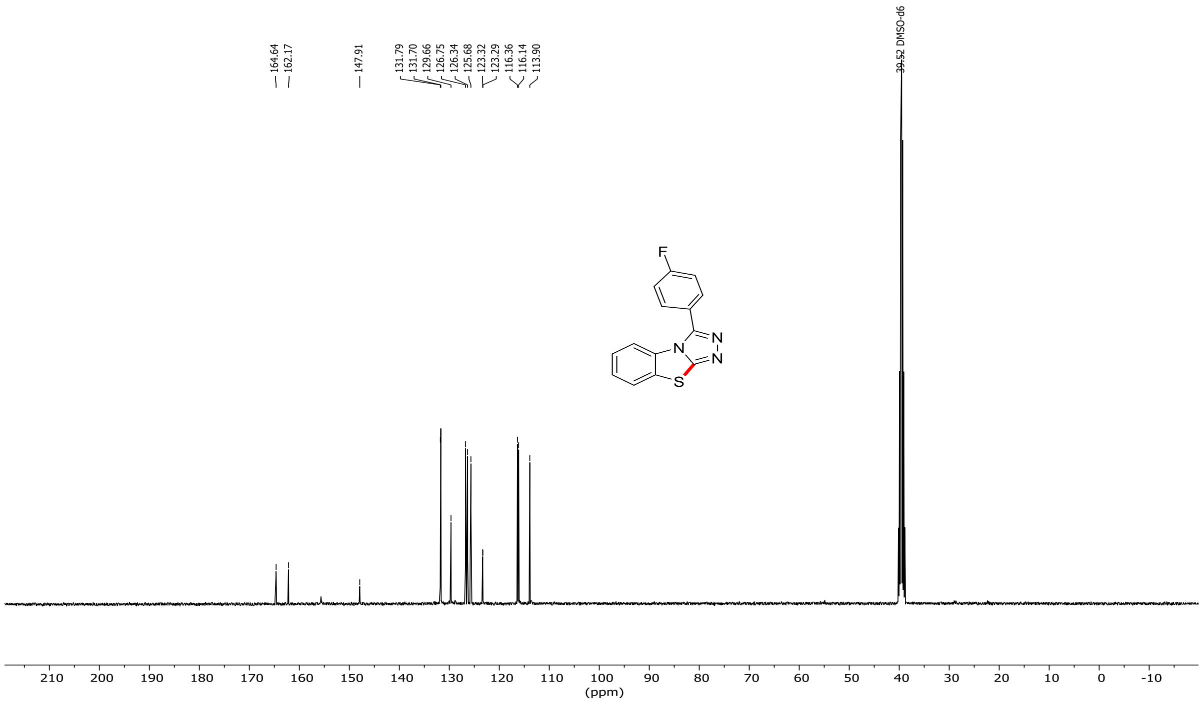

Figure S113: ^{13}C NMR spectrum of 3-(4-fluorophenyl)benzo[4,5]thiazolo[2,3-*c*][1,2,4]triazole (**4an**) (100 MHz, DMSO-d_6, 298 K).

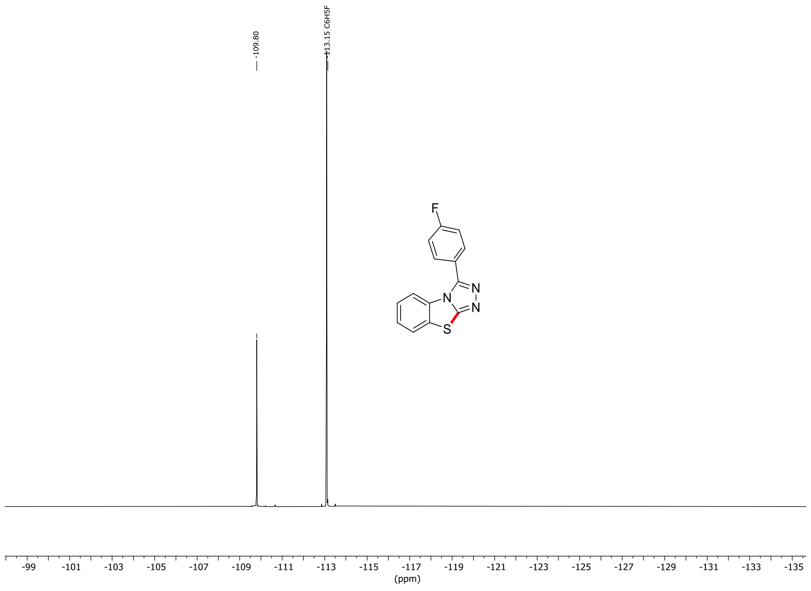

Figure S114: ^{19}F NMR spectrum of 3-(4-fluorophenyl)benzo[4,5]thiazolo[2,3-*c*][1,2,4]triazole (**4an**) (376 MHz, DMSO-d_6, 298 K).

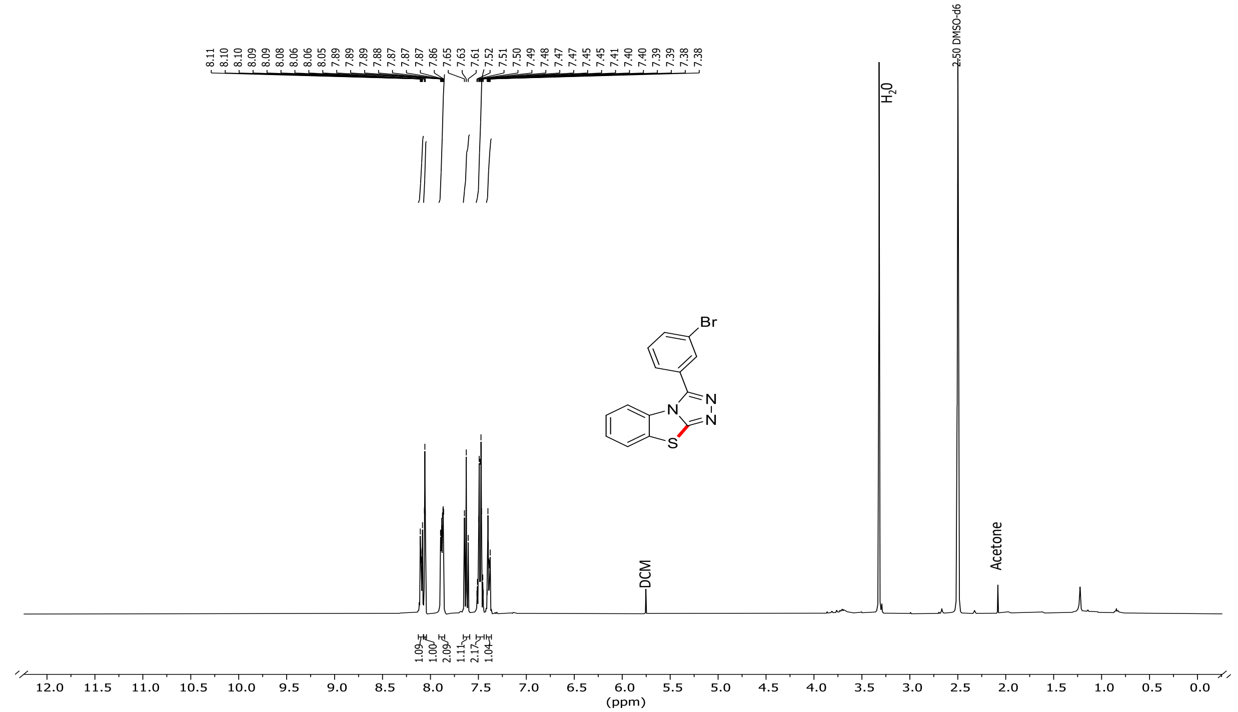

Figure S115: ^{1}H NMR spectrum of 3-(3-bromophenyl)benzo[4,5]thiazolo[2,3-*c*][1,2,4]triazole (**4ao**) (400 MHz, DMSO-d_6, 298 K).

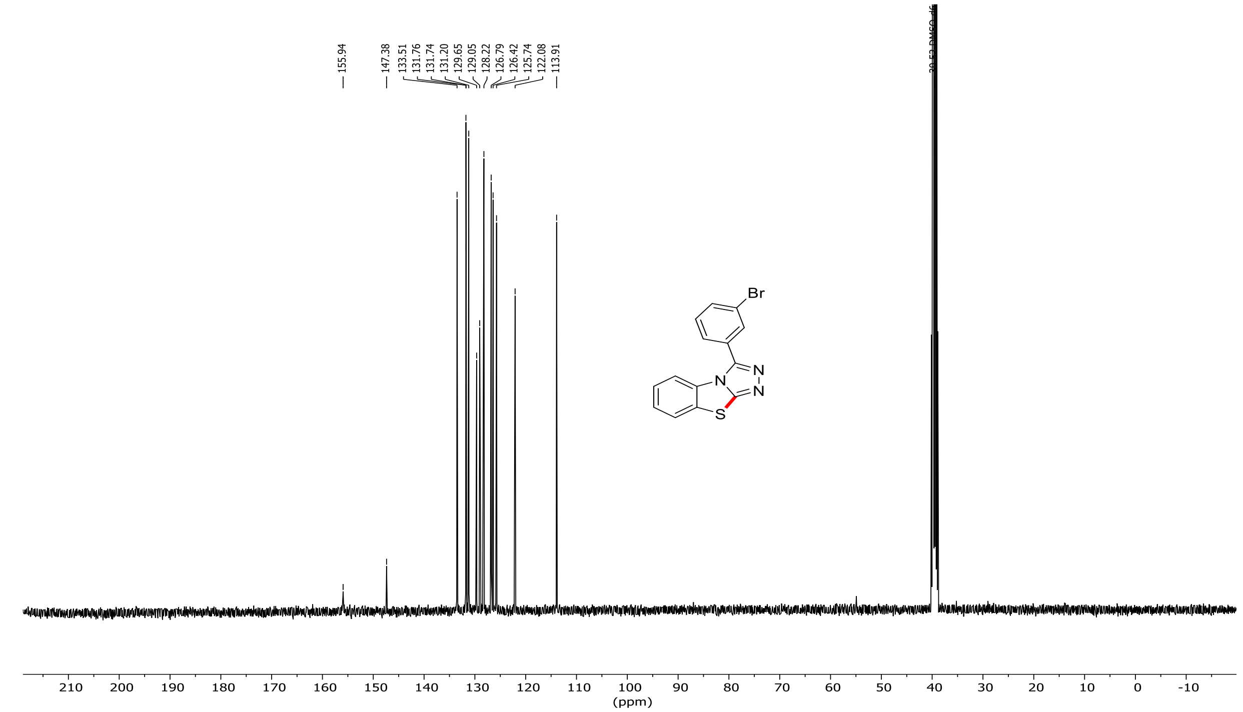

Figure S116: ^{13}C NMR spectrum of 3-(3-bromophenyl)benzo[4,5]thiazolo[2,3-*c*][1,2,4]triazole (**4ao**) (100 MHz, DMSO-d_6, 298 K).

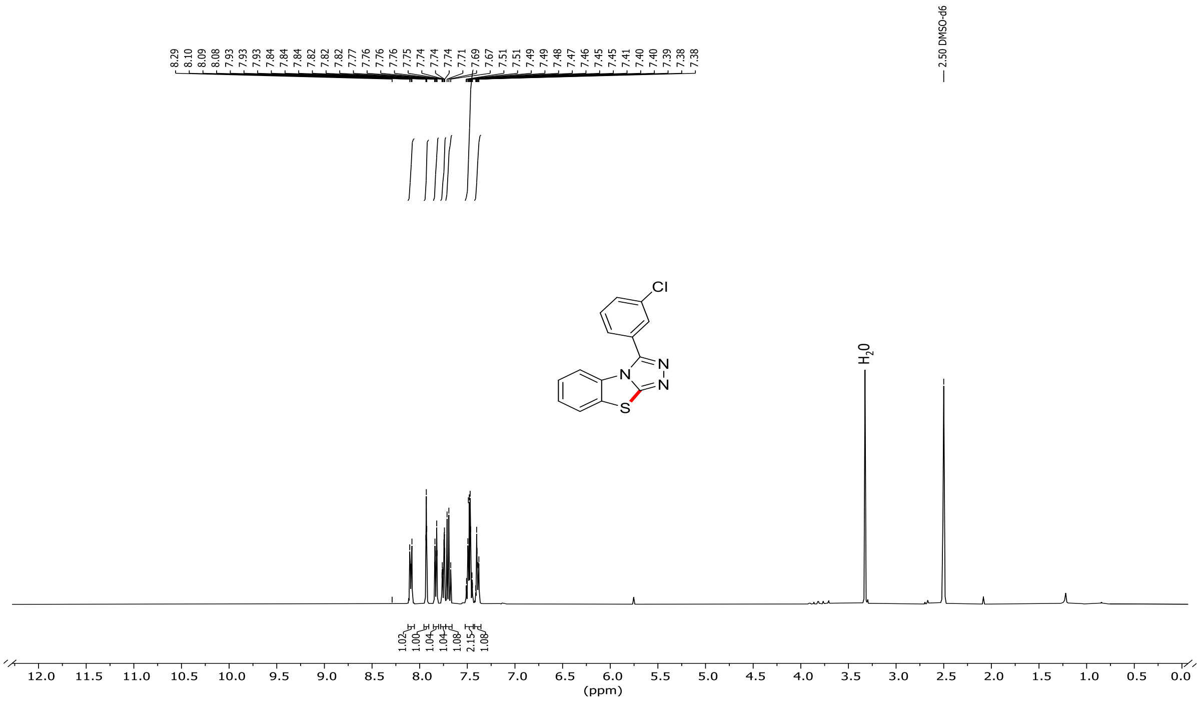

Figure S117: ^{1}H NMR spectrum of 3-(3-chlorophenyl)benzo[4,5]thiazolo[2,3-*c*][1,2,4]triazole. (**4ap**) (400 MHz, DMSO-d_6, 298 K).

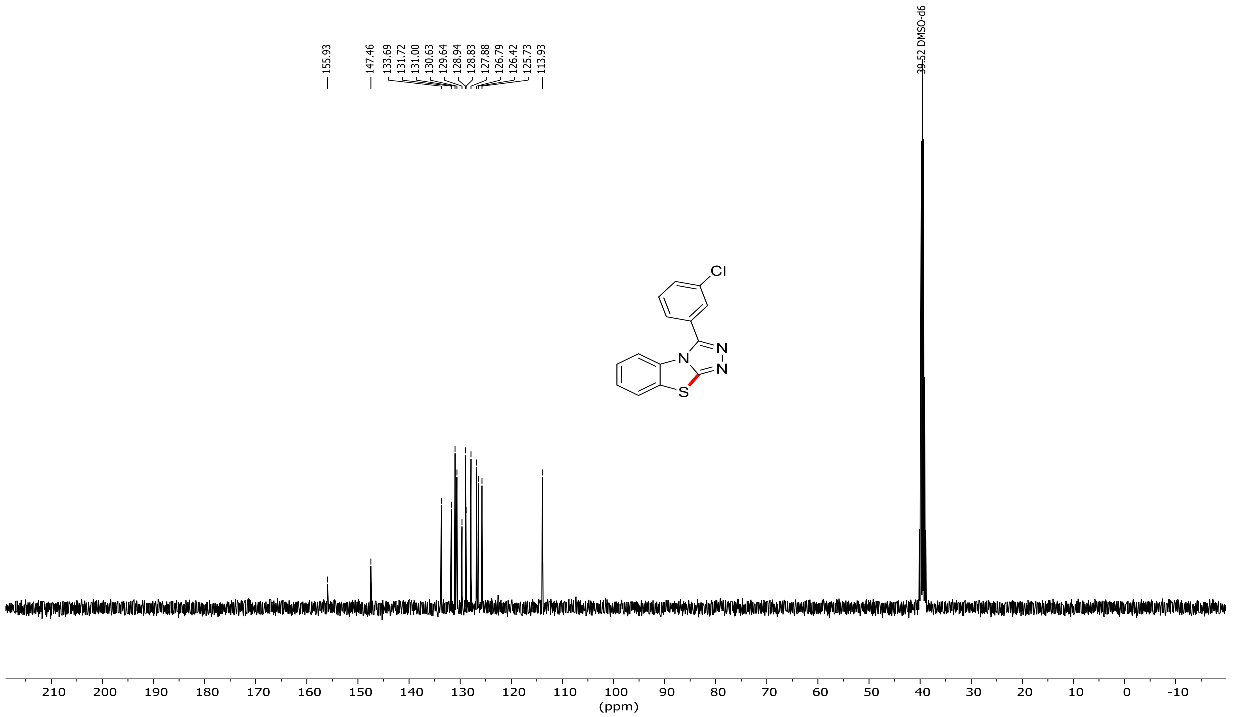

Figure S118: ^{13}C NMR spectrum of 3-(3-chlorophenyl)benzo[4,5]thiazolo[2,3-*c*][1,2,4]triazole. (**4ap**) (100 MHz, DMSO-d_6, 298 K).

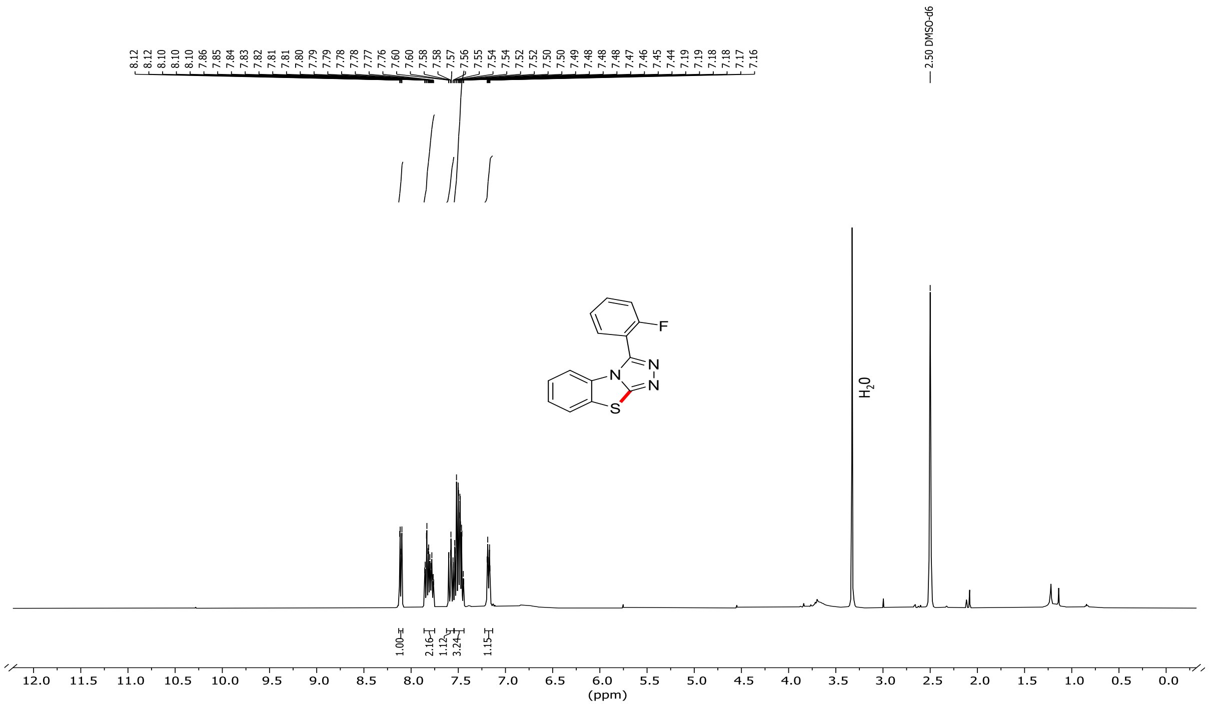

Figure S119: ^{1}H NMR spectrum of 3-(2-fluorophenyl)benzo[4,5]thiazolo[2,3-*c*][1,2,4]triazole (**4aq**) (400 MHz, DMSO-d_6, 298 K).

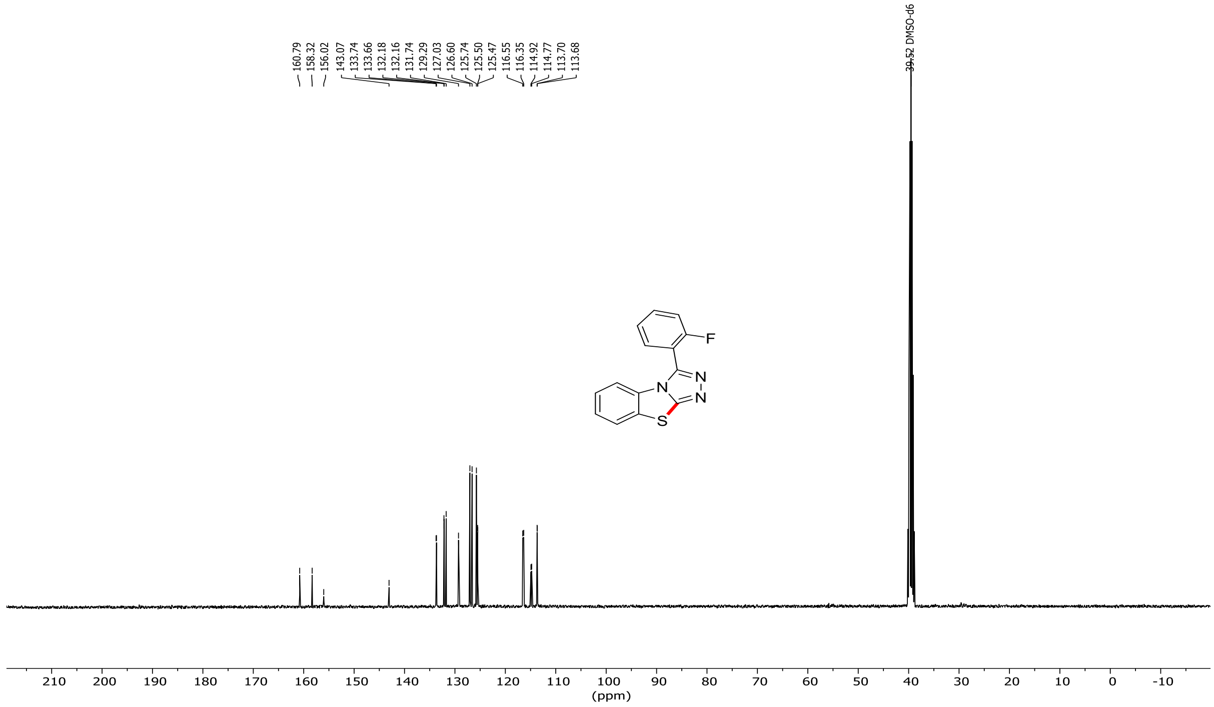

Figure S120: ^{13}C NMR spectrum of 3-(2-fluorophenyl)benzo[4,5]thiazolo[2,3-*c*][1,2,4]triazole (**4aq**) (100 MHz, DMSO-d_6, 298 K).

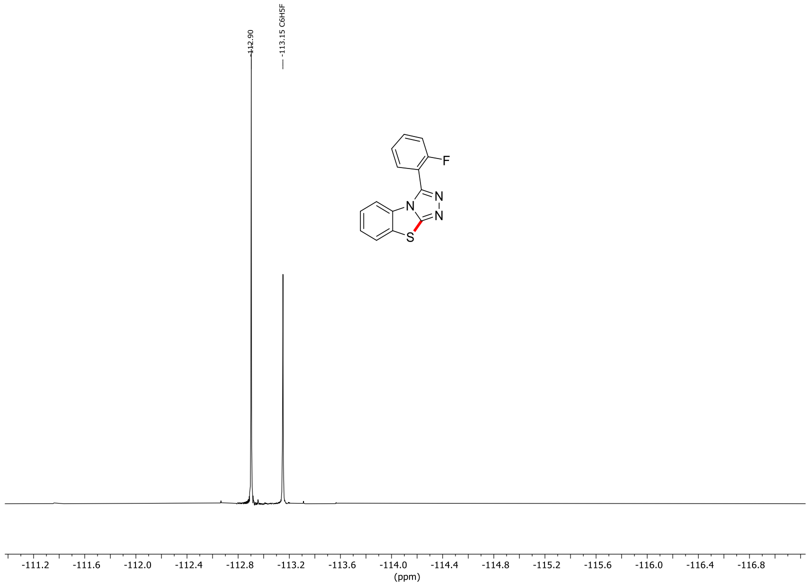

Figure S121: ^{19}F NMR spectrum of 3-(2-fluorophenyl)benzo[4,5]thiazolo[2,3-*c*][1,2,4]triazole (**4aq**) (376 MHz, DMSO-d_6, 298 K).

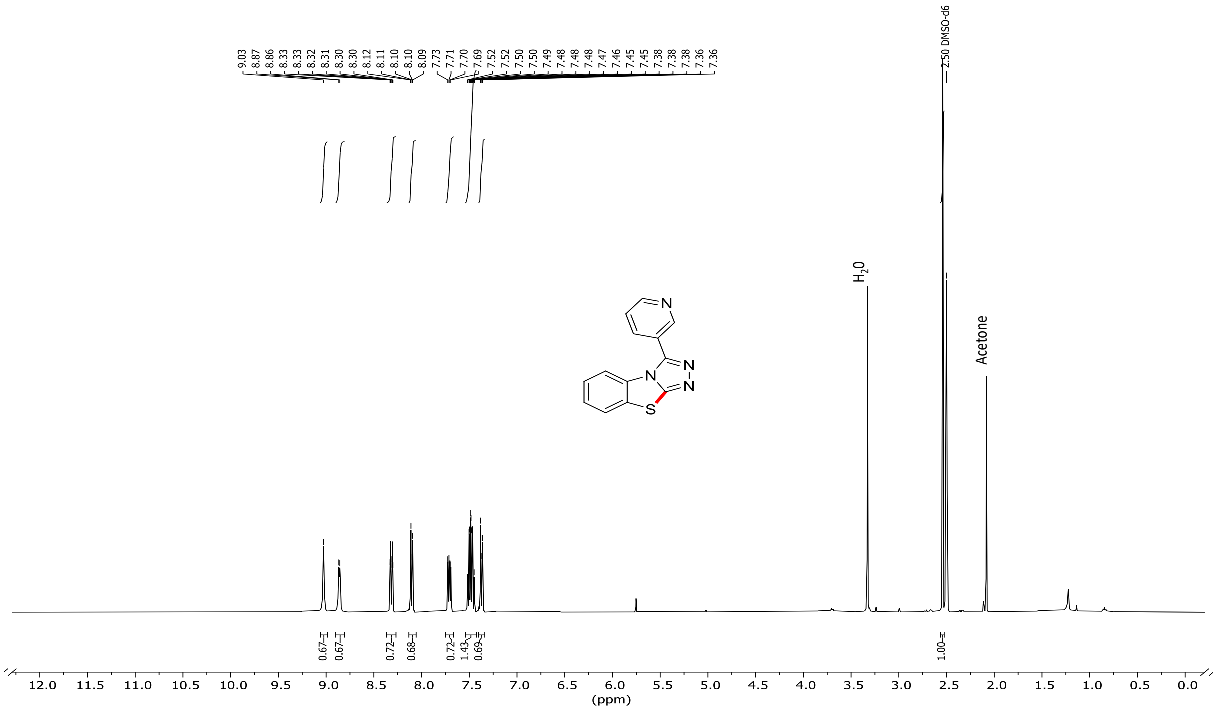

Figure S122: ^{1}H NMR spectrum of 3-(pyridin-2-yl)benzo[4,5]thiazolo[2,3-*c*][1,2,4]triazole (**4ar**) (400 MHz, DMSO-d_6, 298 K).

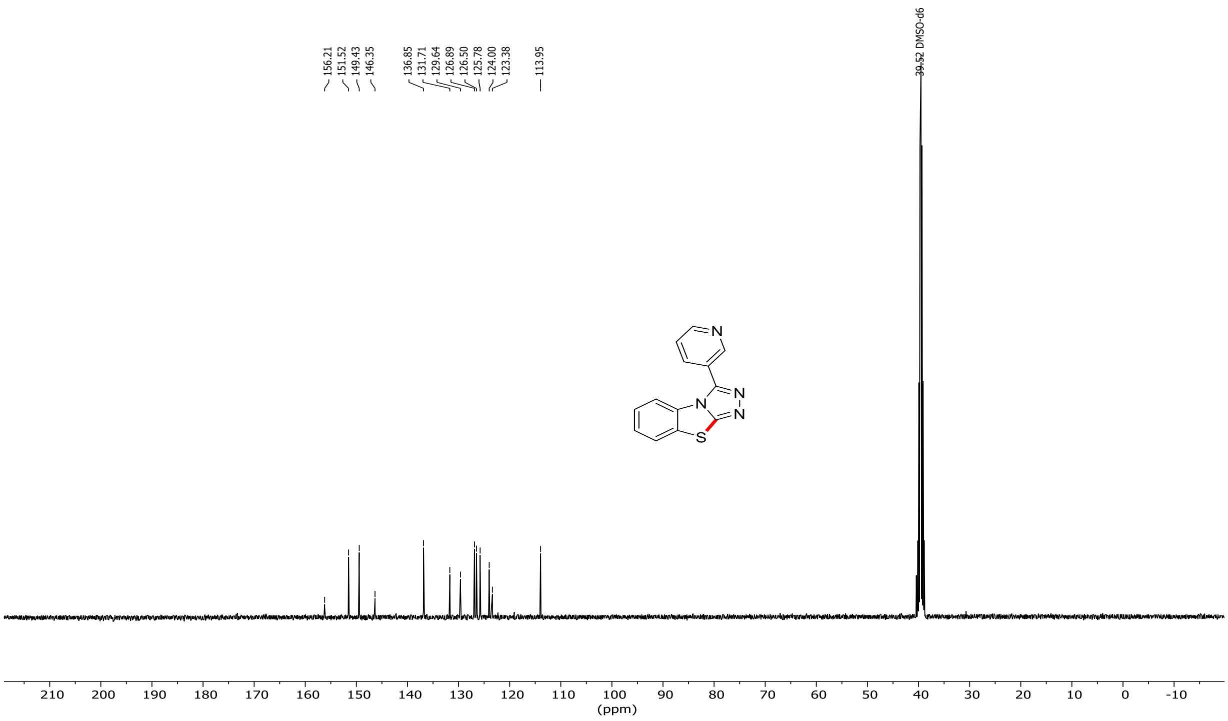

Figure S123: ^{13}C NMR spectrum of 3-(pyridin-2-yl)benzo[4,5]thiazolo[2,3-*c*][1,2,4]triazole (**4ar**) (100 MHz, DMSO-d_6, 298 K).

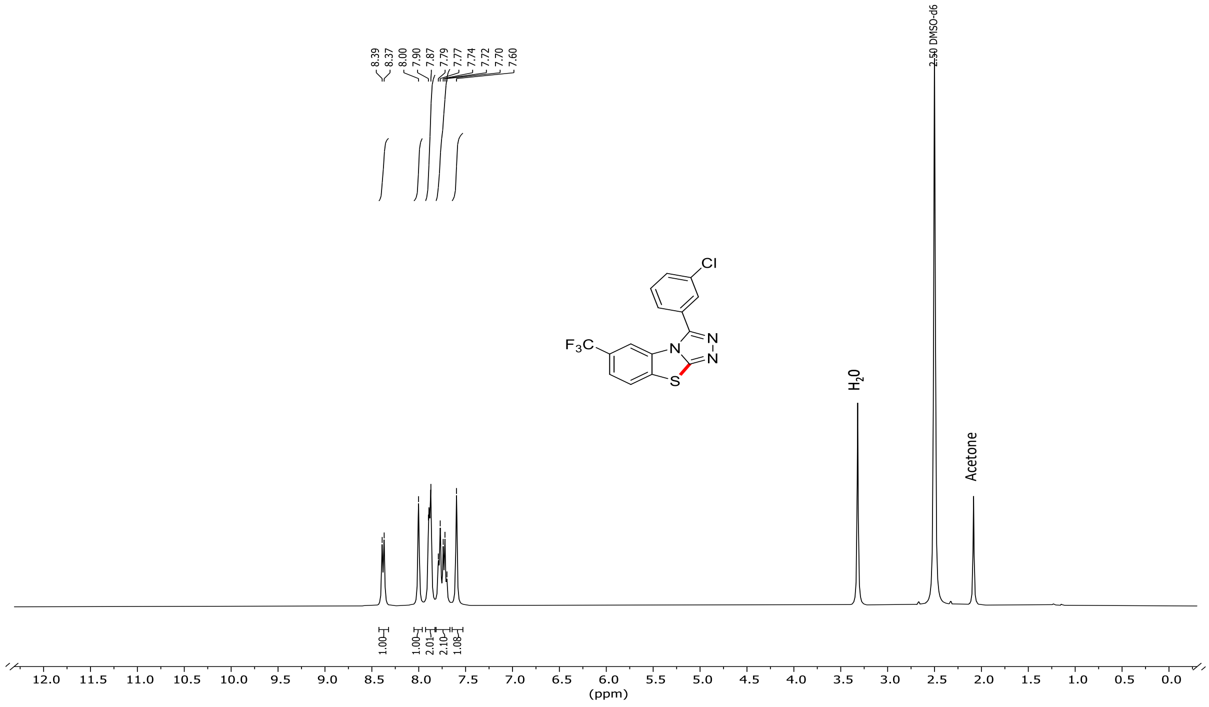

Figure S124: ^{1}H NMR spectrum of 3-(3-chlorophenyl)-6-(trifluoromethyl)benzo[4,5]thiazolo[2,3-*c*][1,2,4]triazole (**4bp**) (400 MHz, DMSO-d_6, 298 K).

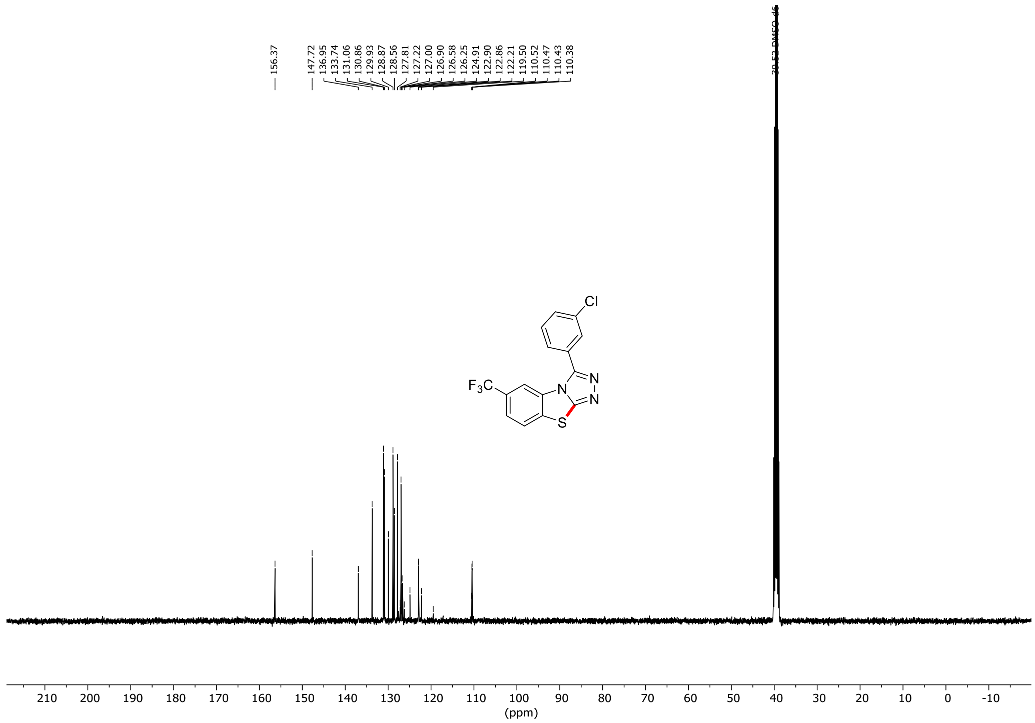

Figure S125: ^{13}C NMR spectrum of 3-(3-chlorophenyl)-6-(trifluoromethyl)benzo[4,5]thiazolo[2,3-*c*][1,2,4]triazole (**4bp**) (100 MHz, DMSO-d_6, 298 K).

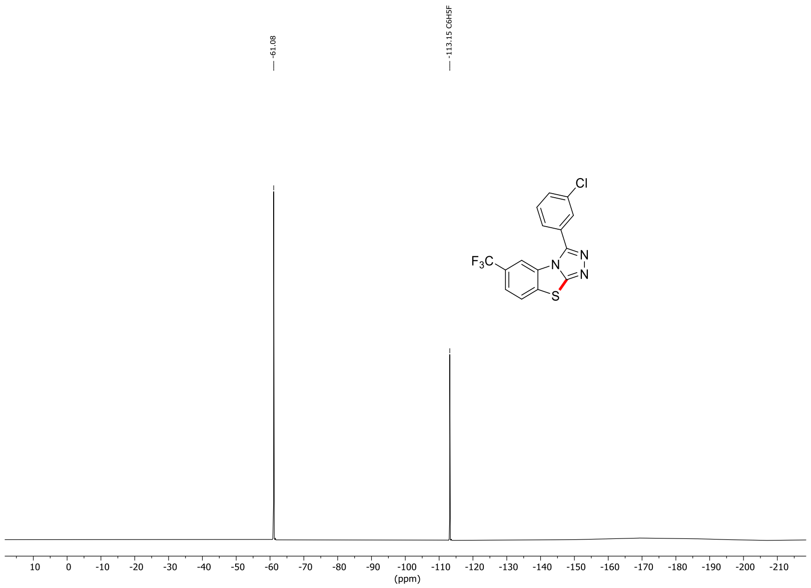

Figure S126: ^{19}F NMR spectrum of 3-(3-chlorophenyl)-6-(trifluoromethyl)benzo[4,5]thiazolo[2,3-*c*][1,2,4]triazole (**4bp**) (376 MHz, DMSO-d_6, 298 K).

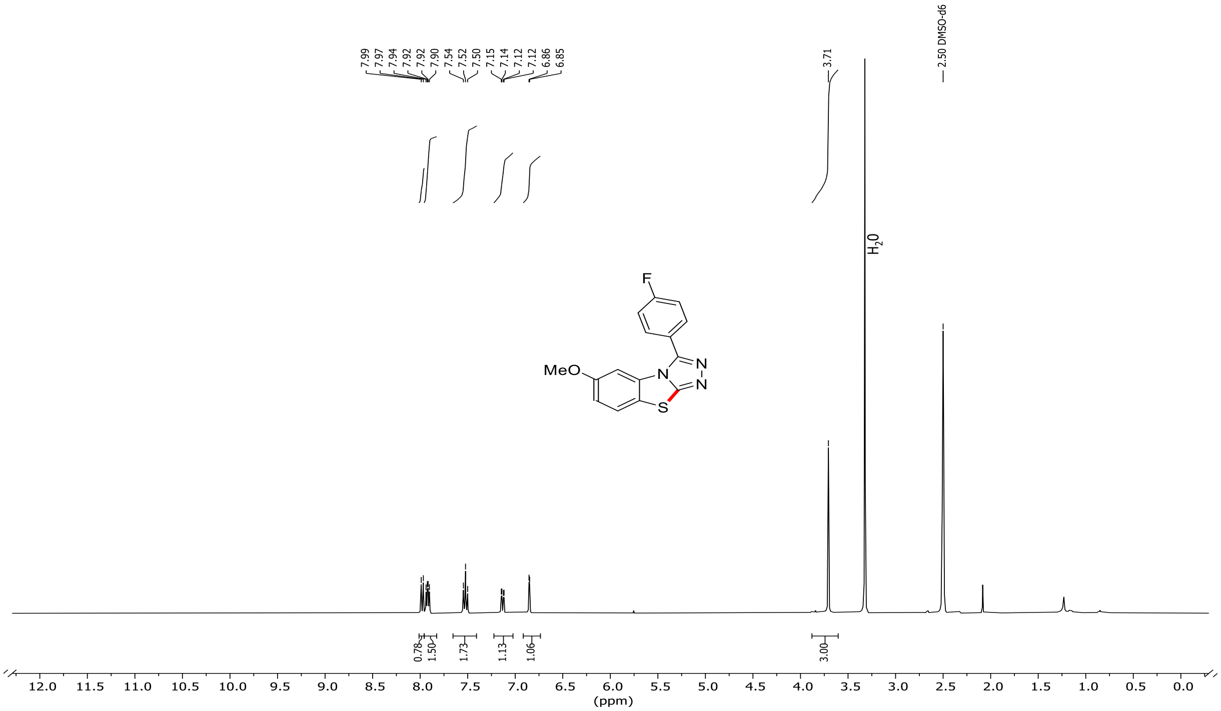

Figure S127: ^{1}H NMR spectrum of 3-(4-fluorophenyl)-6-methoxybenzo[4,5]thiazolo[2,3-*c*][1,2,4]triazole (**4cn**) (400 MHz, DMSO-d_6, 298 K).

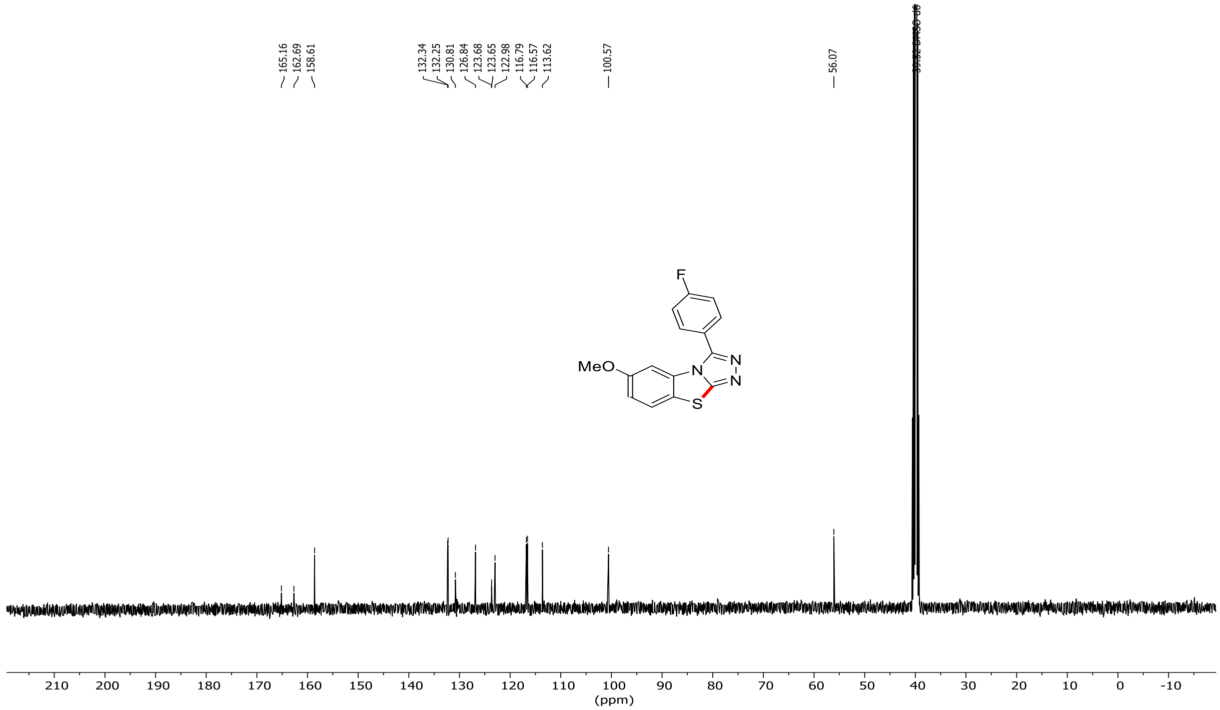

Figure S128: ^{13}C NMR spectrum 3-(4-fluorophenyl)-6-methoxybenzo[4,5]thiazolo[2,3-*c*][1,2,4]triazole (**4cn**) (100 MHz, DMSO-d_6, 298 K).

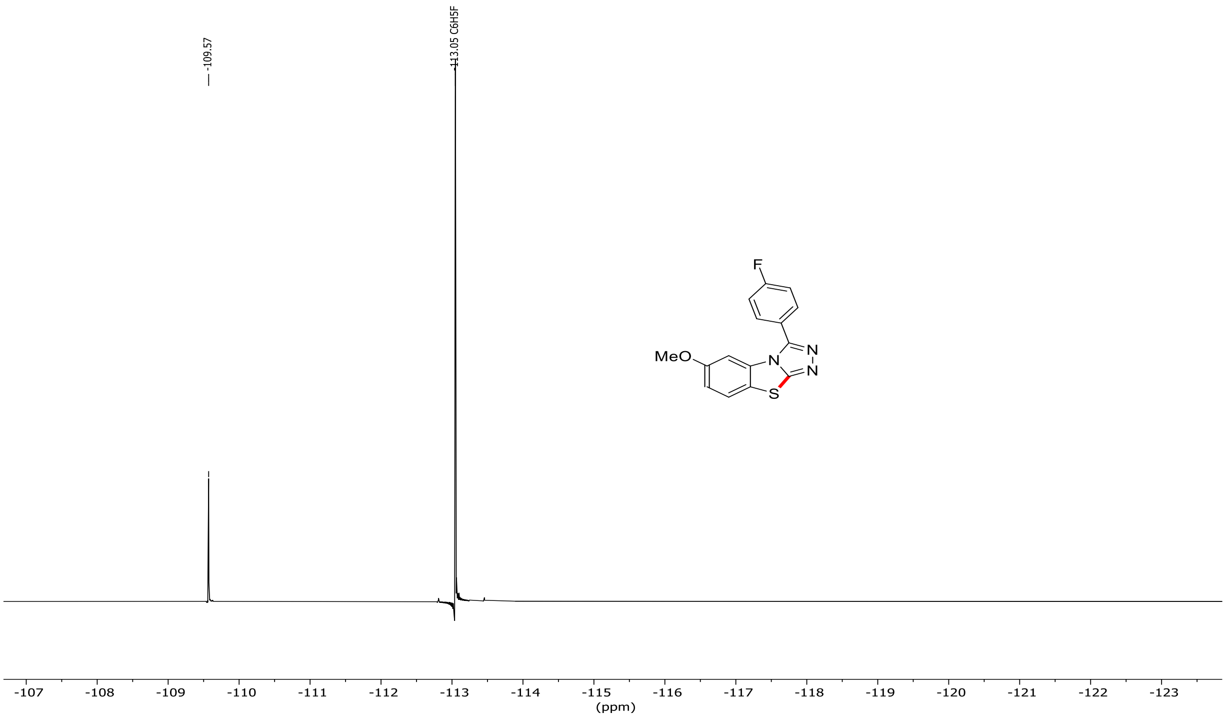

Figure S129: ^{19}F NMR spectrum of 3-(4-fluorophenyl)-6-methoxybenzo[4,5]thiazolo[2,3-*c*][1,2,4]triazole (**4cn**) (376 MHz, DMSO-d_6, 298 K).

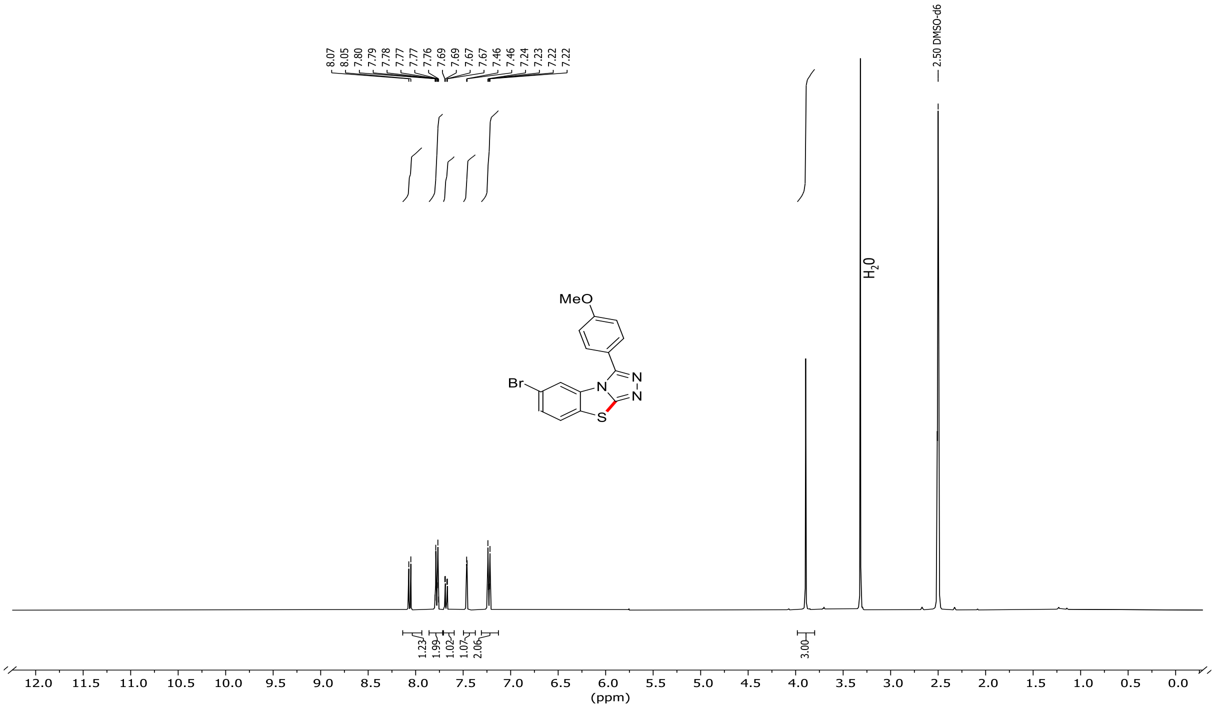

Figure S130: ^{1}H NMR spectrum of 6-bromo-3-(4-methoxyphenyl)benzo[4,5]thiazolo[2,3-*c*][1,2,4]triazole (**4di**) (400 MHz, DMSO-d_6, 298 K).

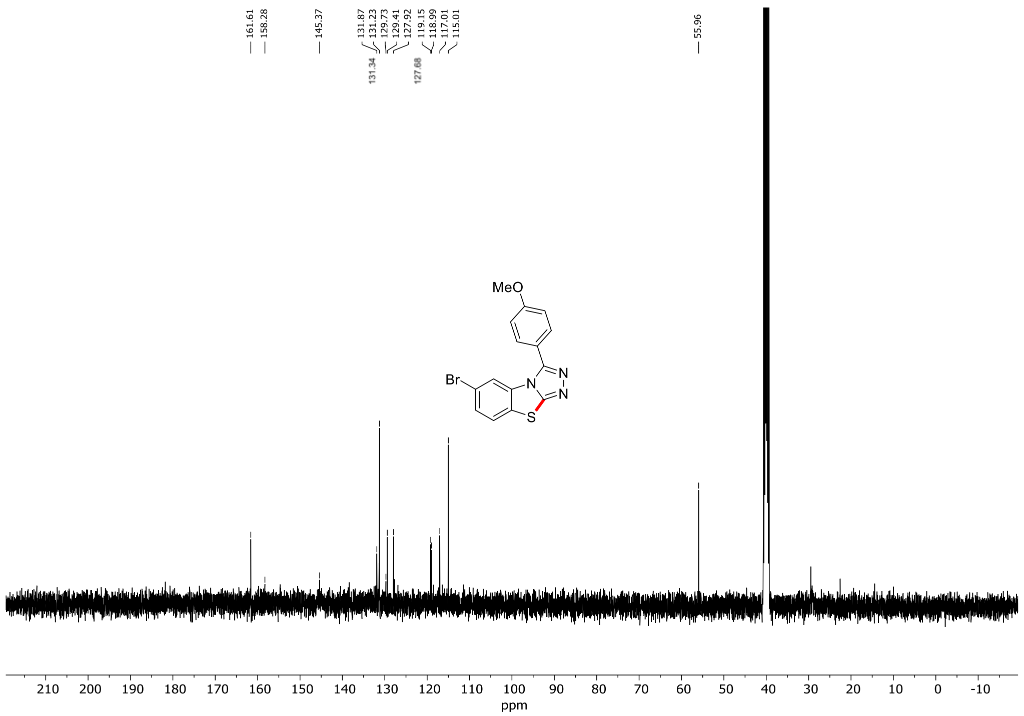

Figure S131: ^{13}C NMR spectrum of 6-bromo-3-(4-methoxyphenyl)benzo[4,5]thiazolo[2,3-*c*][1,2,4]triazole (**4di**) (100 MHz, DMSO-d_6, 298 K).

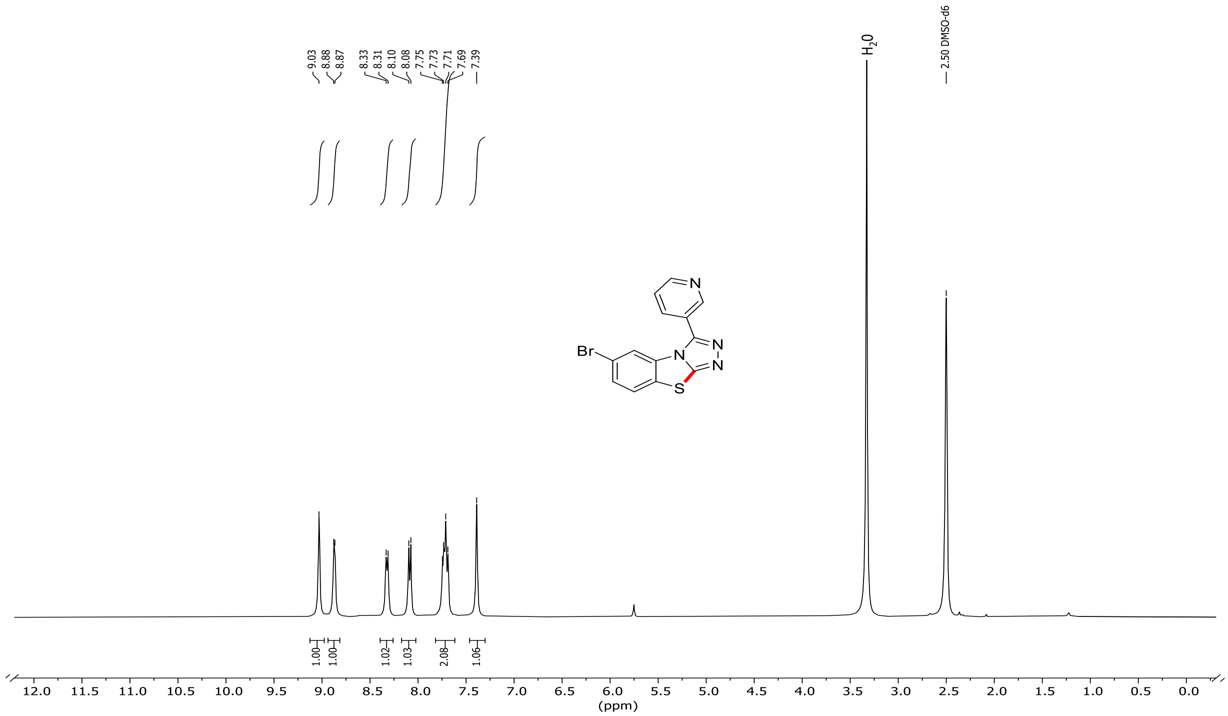

Figure S132: ^{1}H NMR spectrum of 6-bromo-3-(pyridin-3-yl)benzo[4,5]thiazolo[2,3-*c*][1,2,4]triazole (**4dr**) (400 MHz, DMSO-d_6, 298 K).

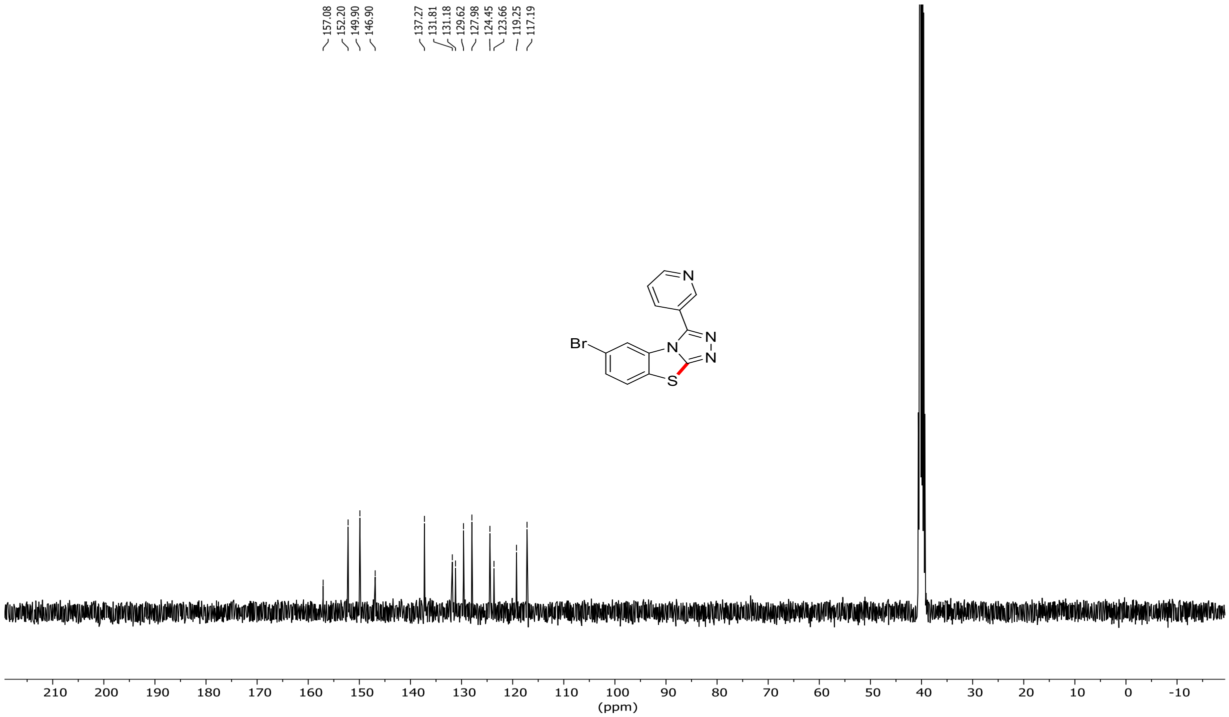

Figure S133: ^{13}C NMR spectrum 6-bromo-3-(pyridin-3-yl)benzo[4,5]thiazolo[2,3-*c*][1,2,4]triazole (**4dr**) (100 MHz, DMSO-d_6, 298 K).

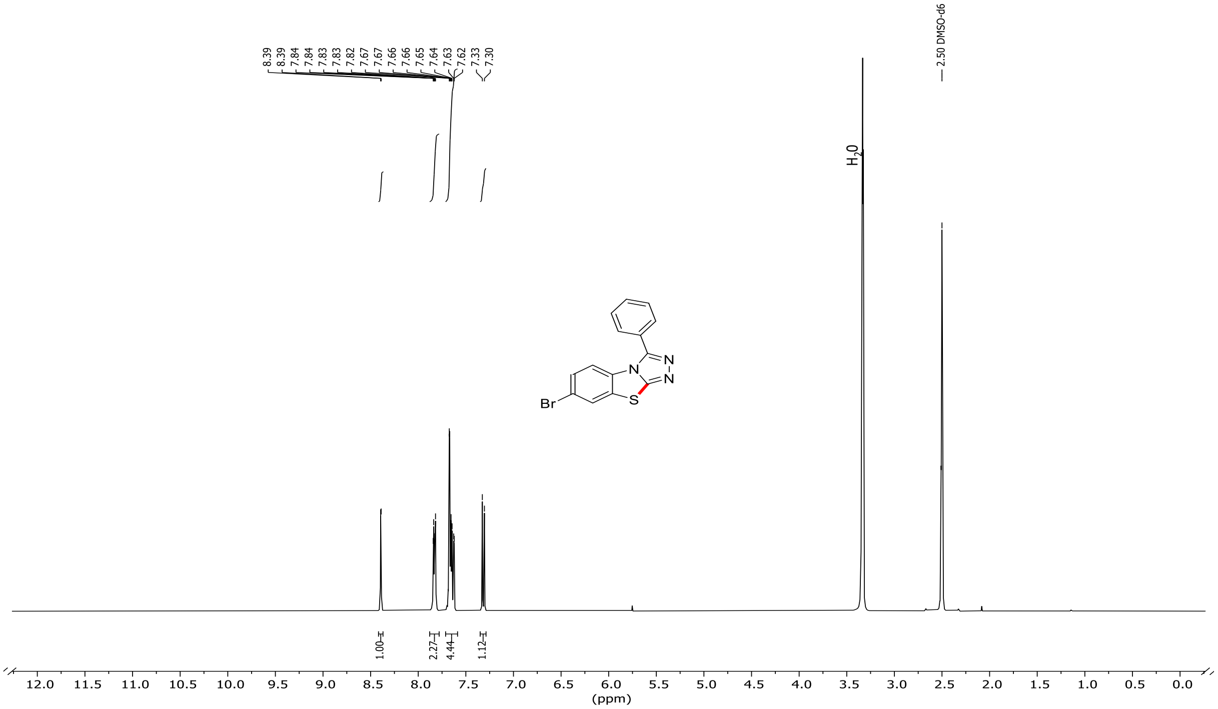

Figure S134: ^{1}H NMR spectrum of 7-bromo-3-phenylbenzo[4,5]thiazolo[2,3-*c*][1,2,4]triazole (**4ee**) (400 MHz, DMSO-d_6, 298 K).

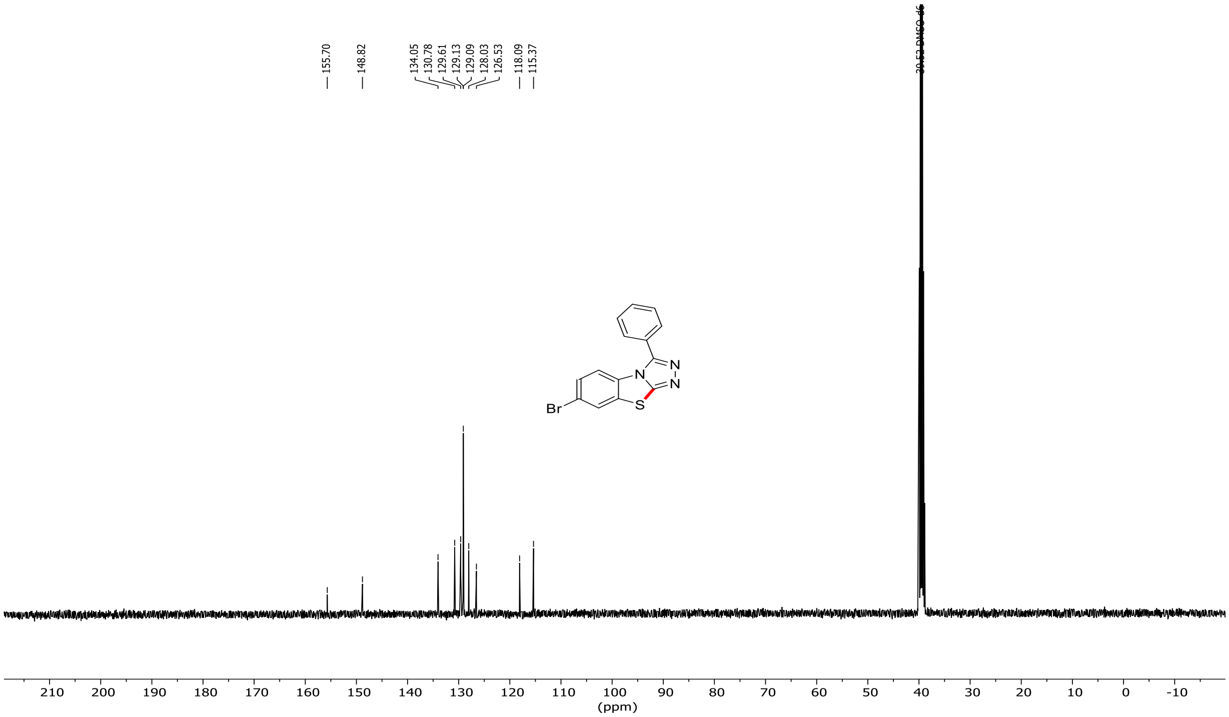

Figure S135: ^{13}C NMR spectrum of 7-bromo-3-phenylbenzo[4,5]thiazolo[2,3-*c*][1,2,4]triazole (**4ee**) (100 MHz, DMSO-d_6, 298 K).

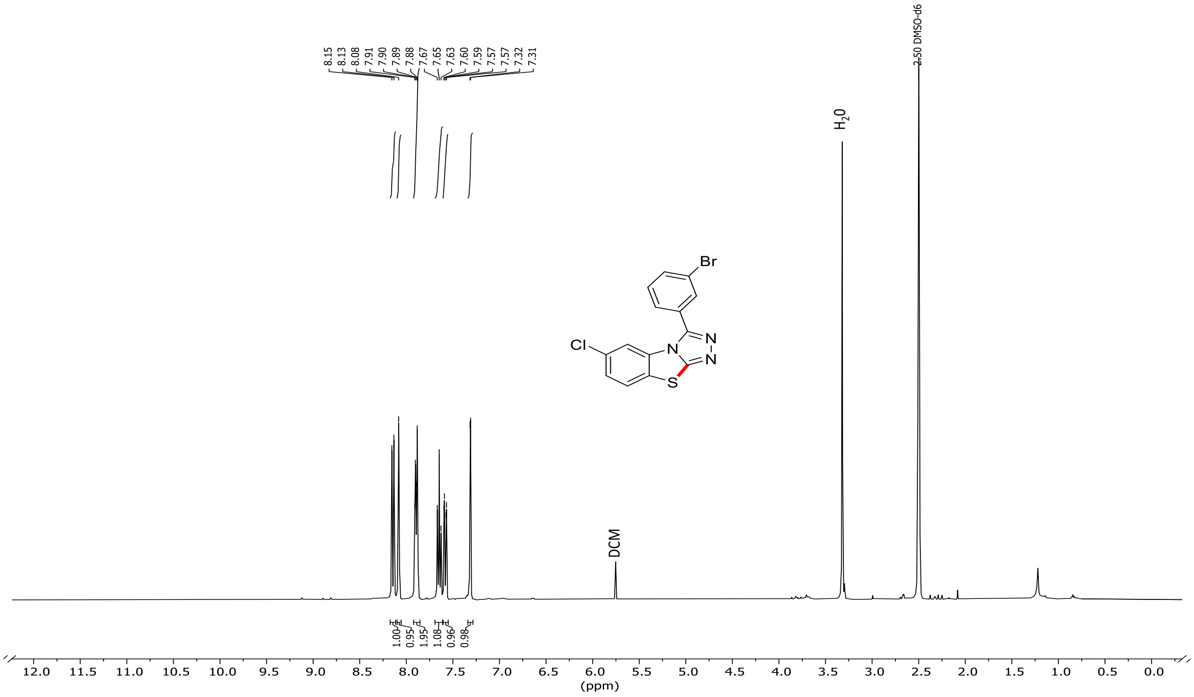

Figure S136: ^{1}H NMR spectrum of 3-(3-bromophenyl)-6-chlorobenzo[4,5]thiazolo[2,3-*c*][1,2,4]triazole (**4ko**) (400 MHz, DMSO-d_6, 298 K).

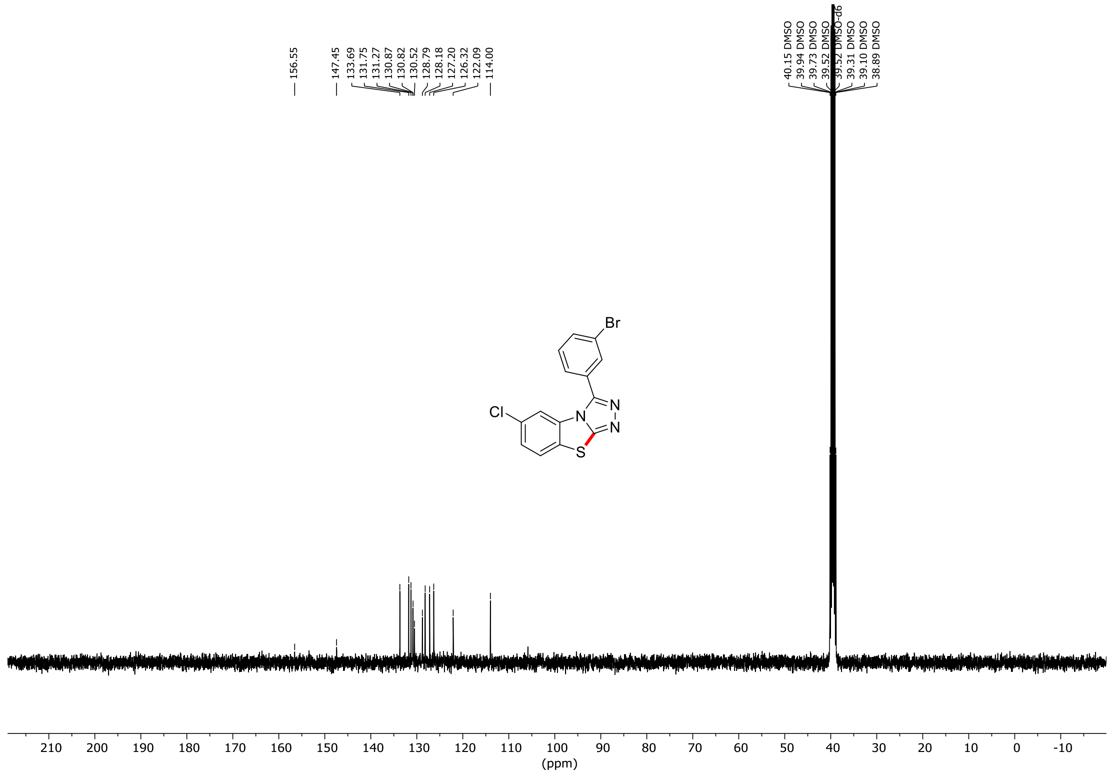

Figure S137: ^{13}C NMR spectrum of 3-(3-bromophenyl)-6-chlorobenzo[4,5]thiazolo[2,3-*c*][1,2,4]triazole (**4ko**) (100 MHz, DMSO-d_6, 298 K).

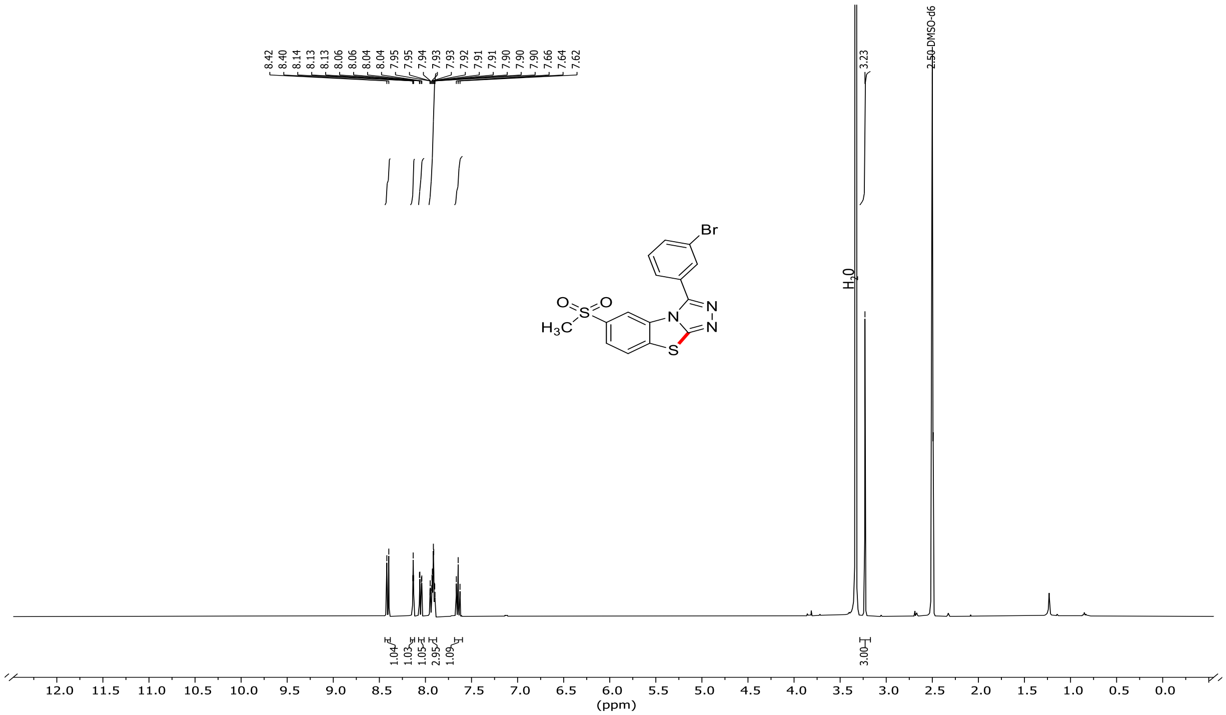

Figure S138: ^{1}H NMR spectrum of 3-(3-bromophenyl)-6-(methylsulfonyl)benzo[4,5]thiazolo[2,3-*c*][1,2,4]triazole (**4mo**) (400 MHz, DMSO-d_6, 298 K).

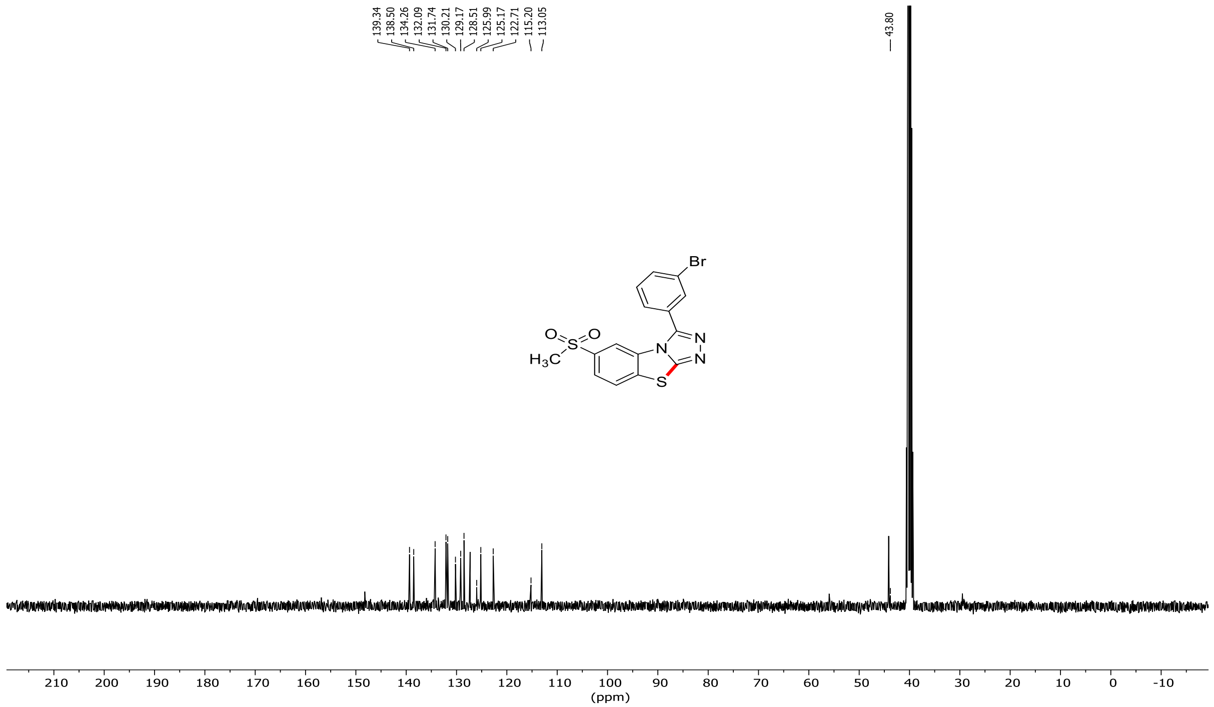

Figure S139: ^{13}C NMR spectrum of 3-(3-bromophenyl)-6-(methylsulfonyl)benzo[4,5]thiazolo[2,3-*c*][1,2,4]triazole (**4mo**) (100 MHz, DMSO-d_6, 298).

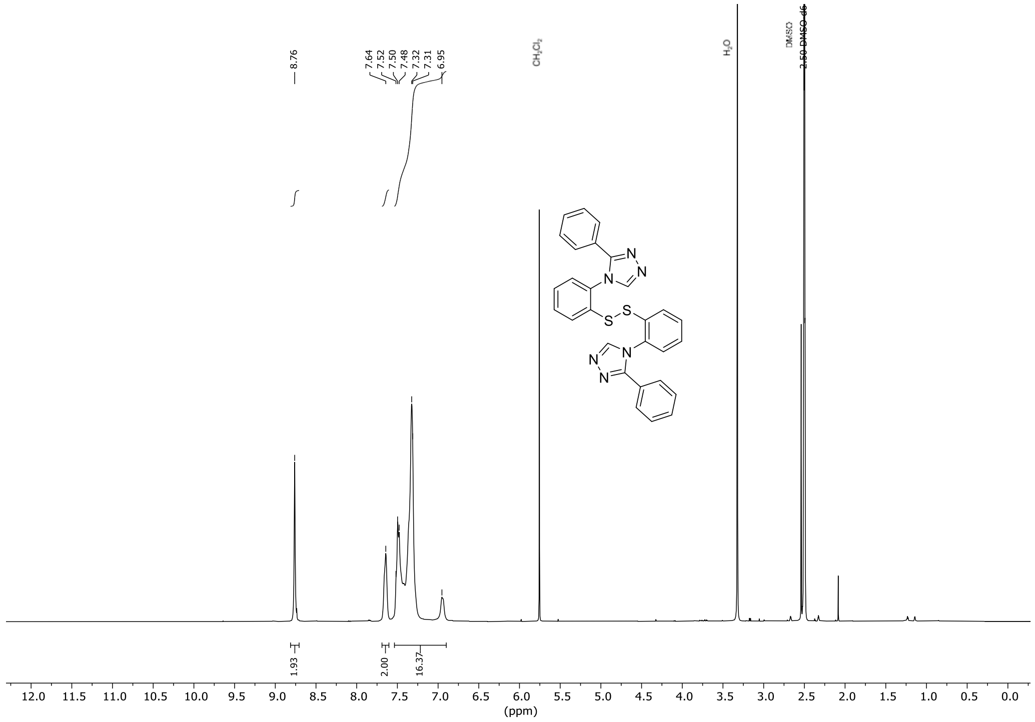

Figure S140: ^{1}H NMR spectrum of 1,2-bis(2-(3-phenyl-4H-1,2,4-triazol-4-yl)phenyl)disulfane (**5ae**) (400 MHz, DMSO-d_6, 298 K).

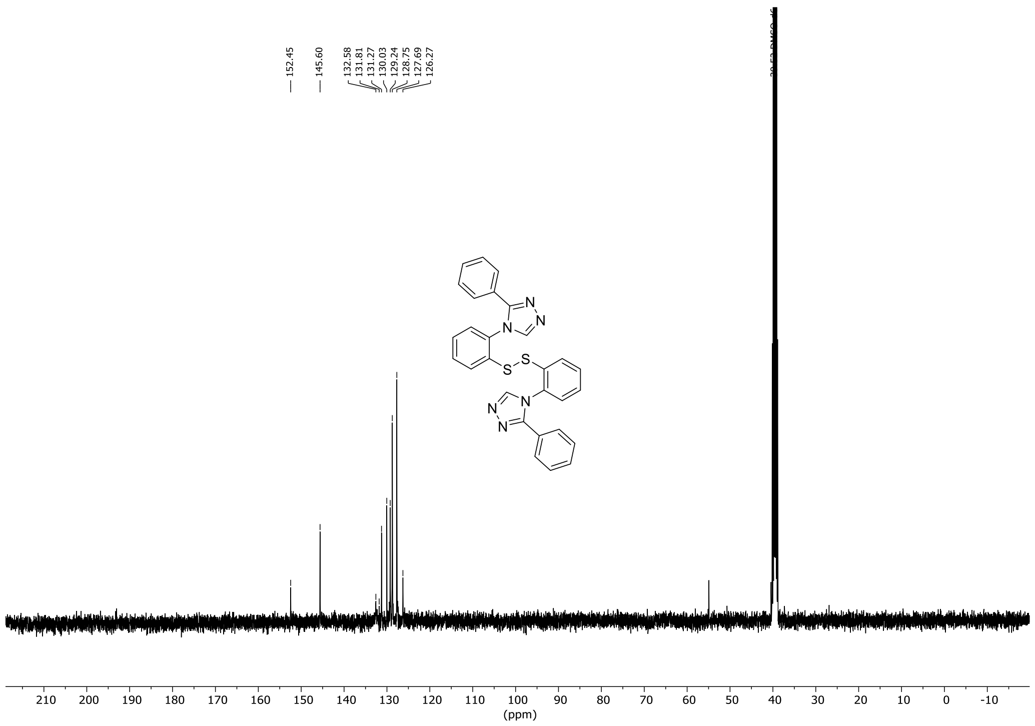

Figure S141: ^{13}C NMR spectrum of 1,2-bis(2-(3-phenyl-4H-1,2,4-triazol-4-yl)phenyl)disulfane (**5ae**) (100 MHz, DMSO-d_6, 298).

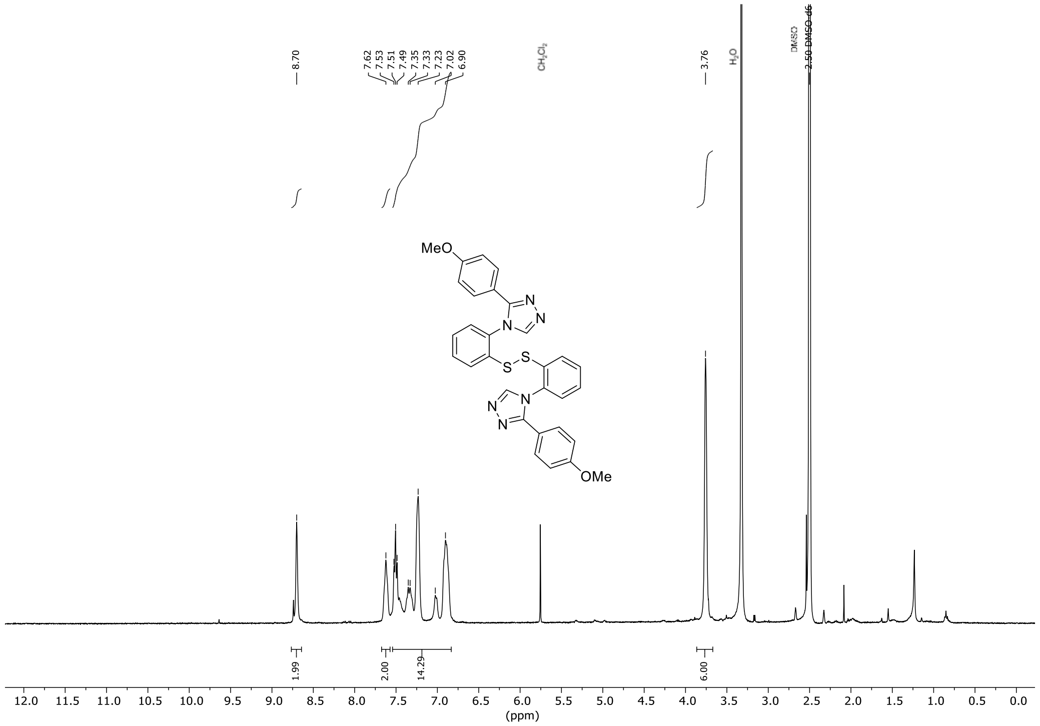

Figure S142: ^{1}H NMR spectrum of 1,2-bis(2-(3-(4-methoxyphenyl)-4H-1,2,4-triazol-4-yl)phenyl)disulfane (**5ai**) (400 MHz, DMSO-d_6, 298 K).

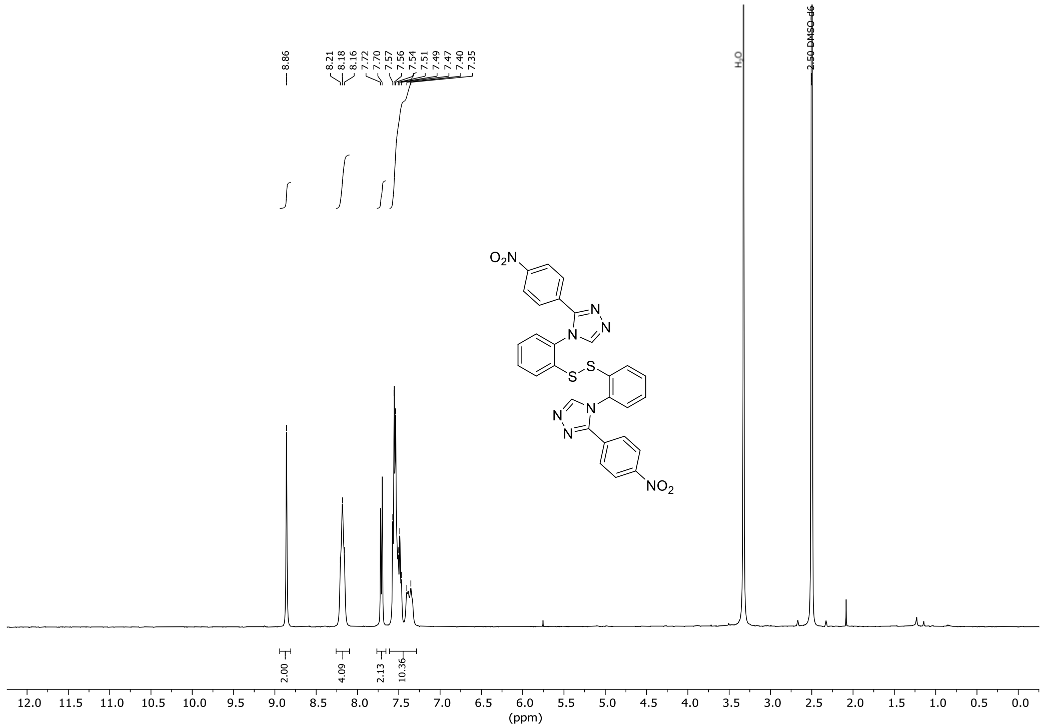

Figure S143: ^{1}H NMR spectrum of 1,2-bis(2-(3-(4-nitrophenyl)-4H-1,2,4-triazol-4-yl)phenyl)disulfane (**5al**) (400 MHz, DMSO-d_6, 298 K).

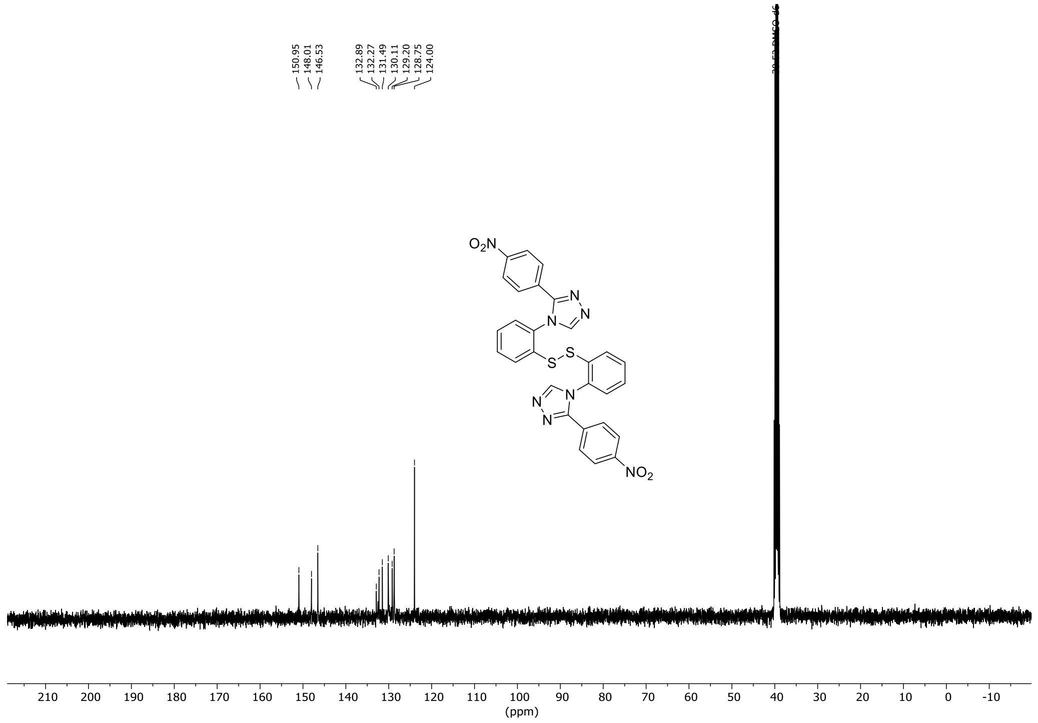

Figure S144: ^{13}C NMR spectrum 1,2-bis(2-(3-(4-nitrophenyl)-4H-1,2,4-triazol-4-yl)phenyl)disulfane (**5al**) (100 MHz, DMSO-d_6, 298).
